# Copper-catalyzed condensation of imines and α-diazo-β-dicarbonyl compounds: modular and regiocontrolled synthesis of multisubstituted pyrroles†
†Electronic supplementary information (ESI) available: CCDC 1040843 and 1063222. For ESI and crystallographic data in CIF or other electronic format see DOI: 10.1039/c5sc02322j


**DOI:** 10.1039/c5sc02322j

**Published:** 2015-08-03

**Authors:** Wei Wen Tan, Naohiko Yoshikai

**Affiliations:** a Division of Chemistry and Biological Chemistry , School of Physical and Mathematical Sciences , Nanyang Technological University , Singapore 637371 , Singapore . Email: nyoshikai@ntu.edu.sg.

## Abstract

In the presence of a copper(ii) catalyst, enolizable imines bearing various *N*-substituents and α-diazo-β-ketoesters undergo denitrogenative and dehydrative condensation to afford highly substituted pyrroles in moderate to good yields with exclusive regioselectivity. The reaction likely involves nucleophilic addition of the imine nitrogen to a copper carbenoid, tautomerization of the resulting azomethine ylide to an α-enaminoketone, and a subsequent enamine–ketone cyclocondensation. With Yb(OTf)_3_ as a unique cocatalyst, α-diazo-β-diketones also participate in the same condensation reaction. The present reaction is applicable to acyclic, exocyclic, and endocyclic imines with tolerance of a broad range of functional groups and heterocyclic moieties, thus opening a new convenient route for the synthesis of the lamellarin family of natural products.

## Abstract 2

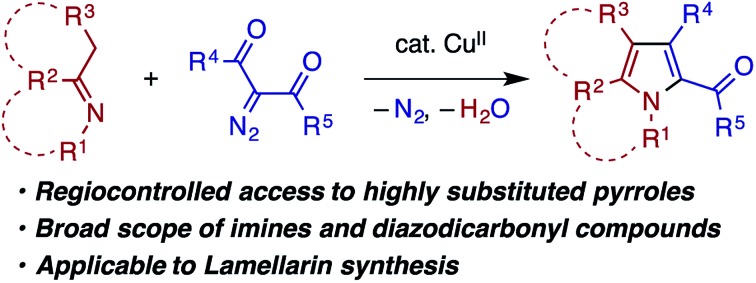
The Cu-catalyzed condensation of ketimines and diazodicarbonyl compounds enables the rapid and regiocontrolled synthesis of pharmaceutically relevant multisubstituted pyrroles.

## Introduction

**Scheme 1:**
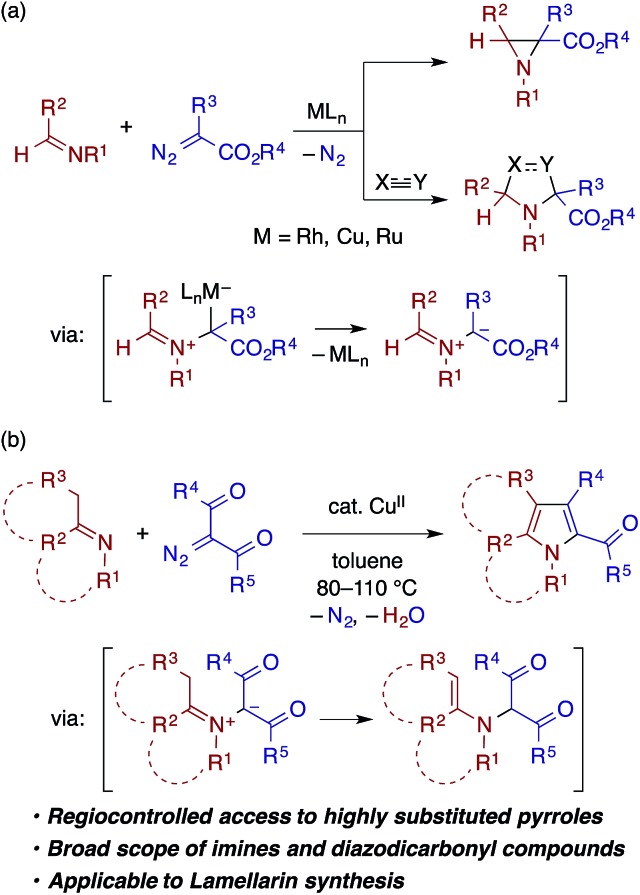
Transition metal-catalyzed condensation of imines and diazocarbonyl compounds.

Transition metal carbenoids, generated through the decomposition of diazocarbonyl compounds, have been proven to serve as extremely versatile electrophilic intermediates for organic synthesis.^1^ Among the various carbenoid transformations, reactions using aldimines bearing *N*-aryl or *N*-alkyl substituents as nucleophilic reaction partners have been extensively explored as they offer useful methods for the synthesis of nitrogen-containing heterocycles (Scheme 1a). In a prototypical reaction pattern, the nucleophilic addition of the aldimine nitrogen atom to the carbenoid gives rise to a metal-bound or a free azomethine ylide.^2^ The azomethine ylide then undergoes intramolecular cyclization to afford an aziridine derivative—a process that can be made enantioselective using a chiral metal catalyst.^3^ The azomethine ylide can also be intercepted by an appropriate dipolarophile, such as an electron-deficient alkene or alkyne, thus affording a pyrrolidine or related heterocycle through [3 + 2] cycloaddition.^4^,^5^

In contrast to the extensive studies on the carbenoid reactions with aldimines, reports on transition metal-catalyzed reactions of ketimines with diazocarbonyl compounds are very rare. While isatin-derived ketimines were reported to react with a rhodium carbenoid derived from diazomalonate to generate azomethine ylides for [3 + 2] cycloaddition,^6^ in other examples, ketimines do not directly react with a metal carbenoid but undergo [2 + 2] cycloaddition with a ketene generated through a Wolff rearrangement of the carbenoid.^7^ To our knowledge, reactions of other types of ketimines, including those bearing α-protons, with metal carbenoids have not been documented in the literature. In pursuit of heterocycle synthesis through the transition metal catalysis of ketimines,^8^ we have found that a ketimine derived from an enolizable ketone participates in the reaction with an α-diazo-β-ketoester (or diketone) under copper catalysis to afford a multisubstituted pyrrole with the concomitant loss of dinitrogen and water (Scheme 1b), which is reported herein. The reaction is considered to involve the nucleophilic addition of the ketimine nitrogen to a copper carbenoid and the tautomerization of the resulting azomethine ylide to an α-enamino-β-dicarbonyl intermediate, which then undergoes dehydrative cyclocondensation to give the pyrrole product. The reaction is applicable to ketimines with various skeletons and *N*-substituents, and features a simple catalytic system and operation.

**Chart 1:**
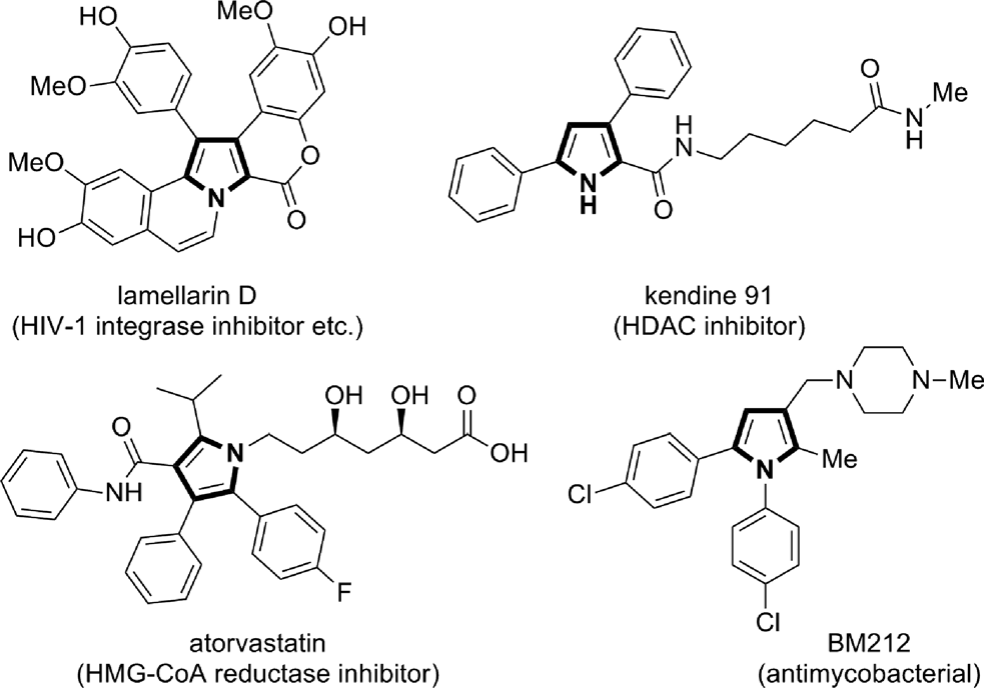
Examples of pyrrole-containing bioactive molecules.

Multisubstituted pyrroles are present in many natural products, pharmaceutically relevant compounds, and other functional molecules (Chart 1).^9^ While a number of new synthetic methods for multisubstituted pyrroles, those catalyzed by transition metals in particular, have been developed in the last decades,^10^,^11^ the demand for simple, robust, and sustainable methods remains high. Notably, in many of the recent transition metal-catalyzed methods, the catalyst plays a key role in the formation of linear intermediates such as α-enaminoketone,^12^ γ-ketoimine (or its tautomers),^13^ and 1,4-diimine,^14^ which undergo dehydrative or deaminative cyclocondensation to afford the pyrrole products. In this context, the present reaction represents a useful addition to the synthetic repertoire for pyrroles, because the substitution patterns of the key α-enamino-β-dicarbonyl intermediates are otherwise not readily accessible.^15^ As such, the present reaction enables the modular and expeditious preparation of dozens of new multisubstituted pyrroles and also opens a new alternative route for the synthesis of the lamellarin family of natural products.

## Results and discussion

The present study commenced with attempts on the condensation of imine **1a**, derived from acetophenone and *p*-anisidine, with α-diazo-β-ketoester **2a**, derived from ethyl isobutyrylacetate (Table 1). Upon the screening of various reaction conditions, the desired condensation was found to proceed efficiently in the presence of catalytic copper(ii) trifluoroacetylacetonate (Cu(tfacac)_2_, 10 mol%) and 4 Å molecular sieves (MS) in toluene at 110 °C to afford the tetrasubstituted pyrrole **3aa** as the exclusive regioisomer in 88% yield (entry 1). The regiochemistry of **3aa** was confirmed by two-dimensional NMR (HMQC and HMBC) analysis. While copper(ii) hexafluoroacetylacetonate (Cu(hfacac)_2_) showed a comparable catalytic activity to Cu(tfacac)_2_ (entry 2), other copper salts such as Cu(acac)_2_ and Cu(OAc)_2_ were much less effective (entries 3 and 4). The use of Rh_2_(OAc)_4_ resulted in the formation of an intractable mixture of products, in which the desired product **3aa** was not detected (entry 5). The yield of **3aa** diminished substantially in the absence of the 4 Å MS (entry 6). The reduction of the catalyst loading (to 5 mol%) or the reaction temperature (to 90 °C) resulted in slightly lower yields (entries 7 and 8). 1,2-Dichloroethane (DCE) can be used as an alternative solvent (entry 9), while the reaction was completely shut down in DMSO (entry 10). Note that the formation of an aziridine product was not observed during the optimization study.3a–e

**Table 1:** Screening of the reaction conditions^*a*^

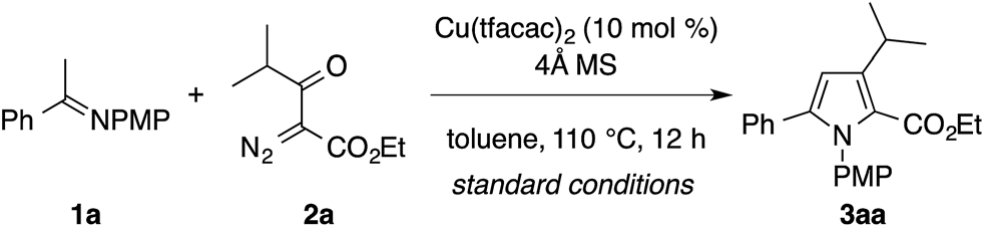		
Entry	Deviation from the standard conditions	Yield^*b*^ (%)
1	None	88^*c*^
2	Cu(hfacac)_2_ instead of Cu(tfacac)_2_	87
3	Cu(acac)_2_ instead of Cu(tfacac)_2_	7
4	Cu(OAc)_2_ instead of Cu(tfacac)_2_	21
5	Rh_2_(OAc)_4_ instead of Cu(tfacac)_2_	0
6	Without 4 Å MS	52
7	5 mol% of Cu(tfacac)_2_	76
8	*T* = 90 °C	82
9	DCE instead of toluene	81
10	DMSO instead of toluene	0

^*a*^The reaction was performed using 0.2 mmol of **1a** and 0.3 mmol of **2a**. PMP = *p*-methoxyphenyl. Cu(tfacac)_2_ = copper(ii) trifluoroacetylacetonate. Cu(hfacac)_2_ = copper(ii) hexafluoroacetylacetonate.

^*b*^Determined by GC.

^*c*^Isolated yield.

To explore the scope of the present pyrrole synthesis, we first subjected various *N*-PMP ketimines **1a–1u** to the reaction with the diazoester **2a** (Table 2). The imines **1a–1o** derived from a wide variety of (hetero)aryl methyl ketones could be condensed with **2a**, affording the tetrasubstituted pyrroles **3aa–3oa** in moderate to good yields. The X-ray diffraction analysis of single crystals of **3ja** unambiguously confirmed its regiochemistry. The reaction of the parent acetophenone imine **1a** could be performed on a gram (5 mmol) scale without problem (see **3aa**). Various functional groups, such as halogen (F, Cl, Br, I), trifluoromethyl, cyano, and nitro groups, as well as heteroaryl moieties such as furyl, benzofuryl, thienyl, and indolyl groups, were tolerated. The imines **1p–1t** derived from propiophenone, 2-phenylacetophenone, cyclopropyl methyl ketone, and cycloalkanones also participated in the reaction with **2a** to afford the corresponding penta- or tetrasubstituted pyrroles **3pa–3ta** in moderate to good yields.

**Table 2:** Condensation of various *N*-PMP imines with diazoketoester **2a**^*a*^

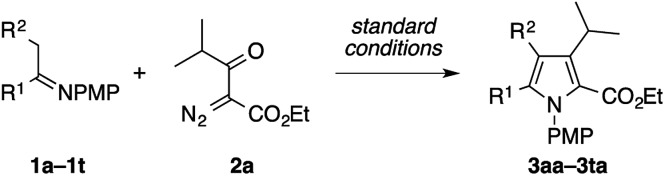
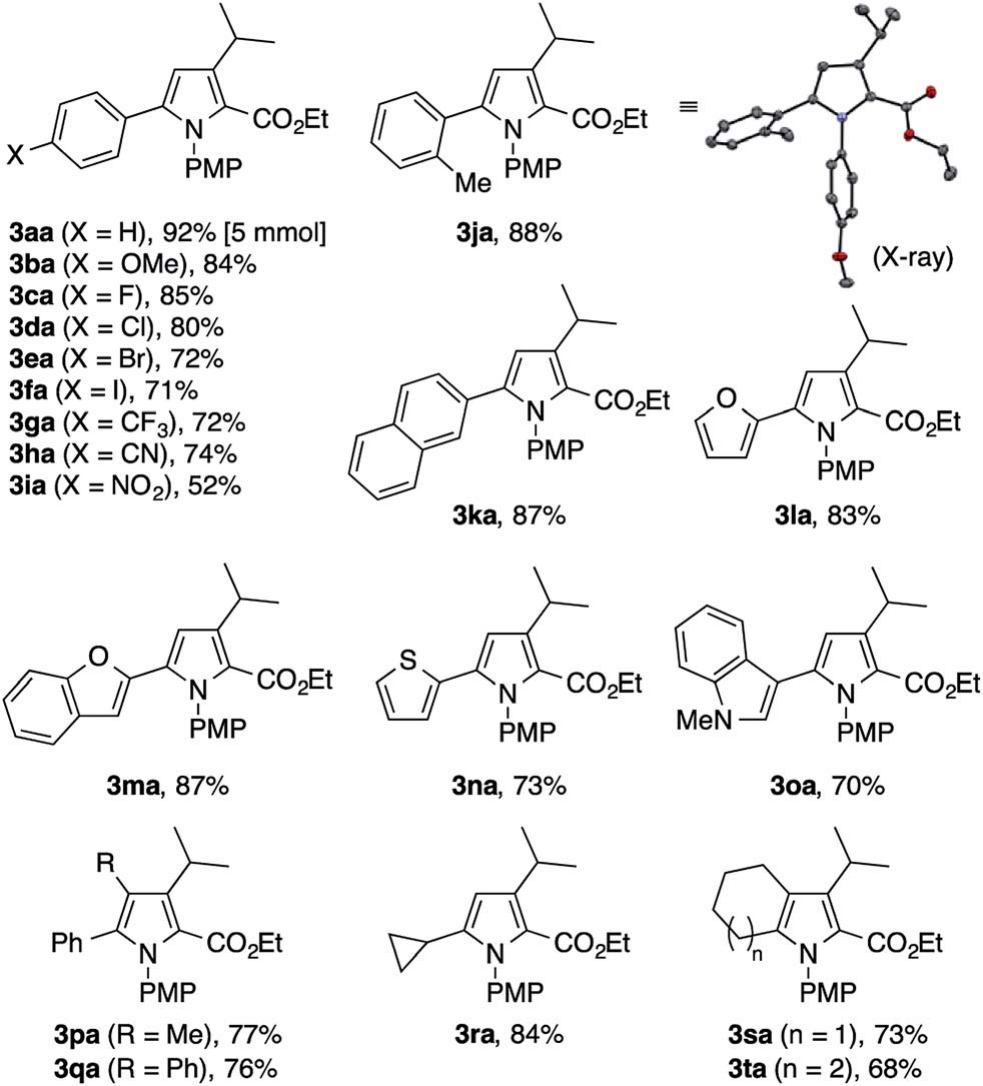

^*a*^The reaction was performed on a 0.2 mmol scale according to the standard conditions described in Table 1.

The present condensation reaction is applicable to imines bearing *N*-substituents other than the PMP group, as illustrated in Table 3. The reaction of the acetophenone imine bearing the *N*-4-chlorophenyl group (**1u**) with **2a** afforded the desired product **3ua** in 42% yield, which was markedly lower than that obtained with the *N*-PMP imine **1a**. This suggests the relevance of the electron-richness of the nitrogen atom to the reactivity of the imine. The imines **1v–1z** bearing removable benzyl, 4-methoxybenzyl (PMB), and allyl groups smoothly participated in the reaction with **2a** to afford the pyrroles **3va–3za** in respectable yields of 57–73%, thus making the preparation of N–H pyrroles feasible. For example, the removal of the PMB group of **3ya** was achieved in 90% yield with the aid of trifluoroacetic acid and anisole. The methyl-substituted dihydroisoquinoline **1aa**, readily prepared by the Bischler–Napieralski reaction, was also amenable to the condensation with **2a** to afford a 5,6-dihydropyrrolo[2,1-*a*]isoquinoline derivative **3aaa**, implying the potential applicability of the present method to the synthesis of the lamellarin family of natural products (*vide infra*).9b–c Interestingly, the benzyl-substituted dihydroisoquinoline **1ab** afforded a mixture of 5,6-dihydropyrrolo[2,1-*a*]isoquinoline **3aba** and an unexpected dihydrobenz[*g*]indolizinone derivative **3aba′**.^16^ The latter product features the migration of the phenyl group of **1ab** from the β-position of the nitrogen atom to the α-position, as well as the concomitant oxidation of the β-position. We consider that these structural changes occur on the reaction pathway leading to the pyrrole product **3aba**, rather than after the formation of **3aba**, because the ratio of **3aba** and **3aba′** was not affected by the reaction time (see Scheme S1 in the ESI† for a possible mechanism).

**Table 3:** Condensation of other imines with diazoketoester **2a**^*a*^

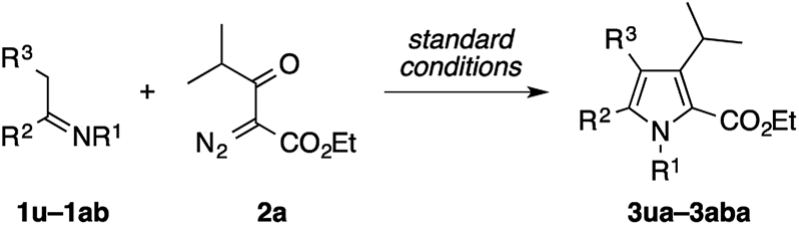
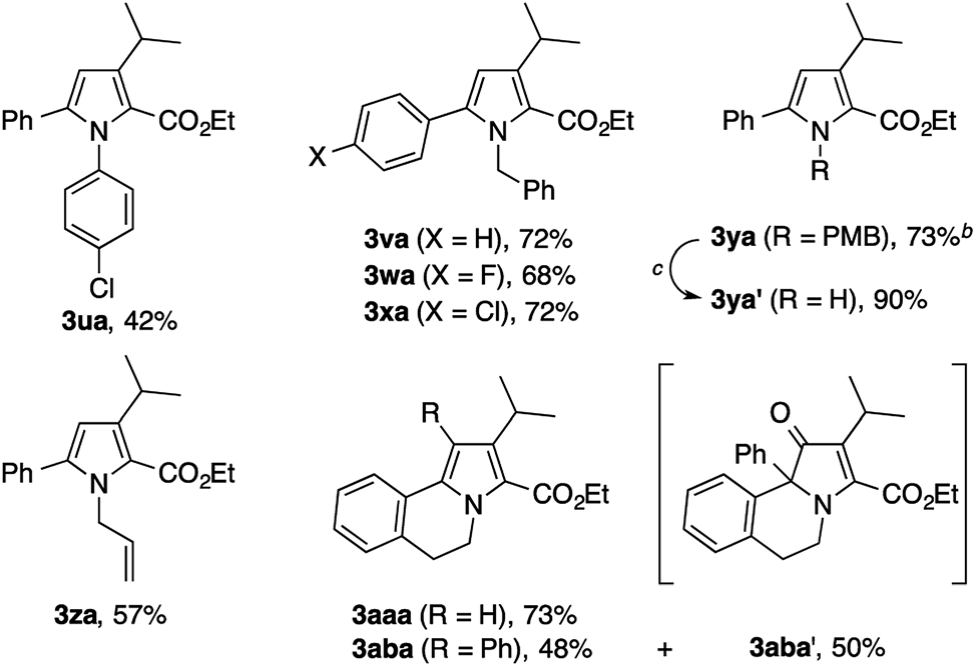

^*a*^The reaction was performed on a 0.2 mmol scale according to the standard conditions described in Table 1.

^*b*^Performed on a 0.5 mmol scale.

^*c*^TFA, anisole, CH_2_Cl_2_, 37 °C, 40 h.

**Scheme 2:**
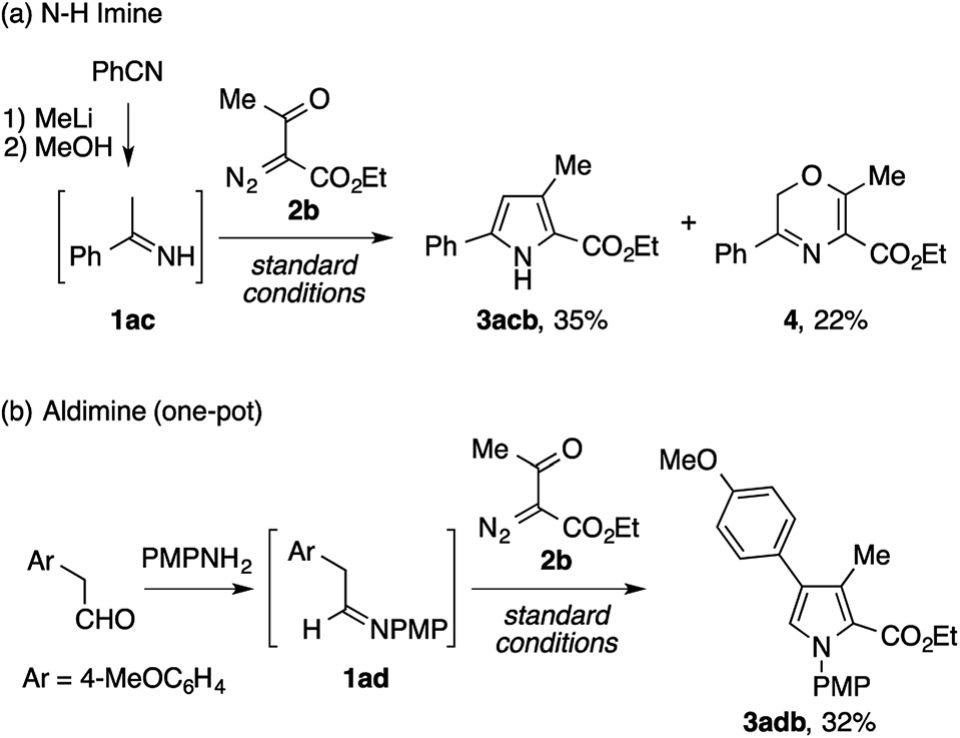
Condensation of unstable imines with diazoketoester **2b**.

Besides the isolable imines described above, we also examined the viability of unstable imines as reactants for the present pyrrole synthesis (Scheme 2). First, an N–H imine of acetophenone **1ac**, prepared from benzonitrile and methyllithium with minimal workup, was subjected to the reaction with ethyl acetyldiazoacetate **2b**, which afforded the desired N–H pyrrole **3acb** in a modest yield (Scheme 4a). Interestingly, the reaction was accompanied by the formation of a 2*H*-1,4-oxazine derivative **4** as a byproduct. Next, the condensation of 4-methoxyphenylacetaldehyde and *p*-anisidine and the subsequent reaction of the resulting aldimine **1ad** and **2b** were performed in a one-pot manner. Although the first condensation step was inevitably complicated by side reactions such as the enamine aldol reaction, the desired tetrasubstituted pyrrole **3adb** was obtained in a modest yield (Scheme 4b).

**Scheme 3:**
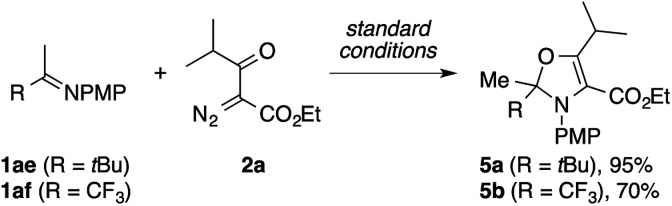
Formation of dihydrooxazole derivatives.

During the exploration of the scope of the imines, we encountered a few cases of an unexpected mode of cyclization (Scheme 3). The reaction of the pinacolone-derived imine **1ae** with **2a** cleanly furnished a 2,3-dihydrooxazole derivative **5a**, rather than the expected pyrrole. The same reaction was also observed using the trifluoroacetone-derived imine **1af**.

We next explored the reaction of imine **1a** with various α-diazo-β-ketoesters **2b–2p** (Table 4). The reaction allowed the facile preparation of tetrasubstituted pyrroles bearing alkyl (entries 1–3), benzyl (entry 4), and (hetero)aryl (entries 5–12) groups in moderate to good yields, with tolerance of functional groups such as bromo and nitro groups (entries 7, 8, and 10). Diazo compounds derived from diethyl 2-oxosuccinate and ethyl 4,4,4-trifluoroacetoacetate could also be condensed with **1a**, thus furnishing 2,3-diethoxycarbonylpyrrole **3an** and 2-ethoxycarbonyl-3-trifluoromethylpyrrole **3ao**, respectively (entries 13 and 14). In addition to the 1,2,3,5-tetrasubstituted pyrroles **3ab–3ao**, the 1,2,5-trisubstituted pyrrole **3ap** was also successfully prepared in a moderate yield using ethyl formyldiazoacetate **2p** as the reactant (entry 15).

**Table 4:** Condensation of the imine **1a** with various α-diazo-β-ketoesters^*a*^

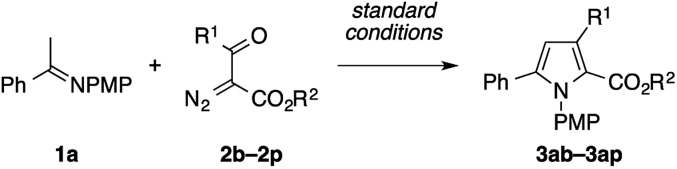				
Entry	R^1^	R^2^	Product	Yield (%)
1	Me	Et	**3ab**	81
2	*c*-C_3_H_5_	Et	**3ac**	80
3	*c*-C_6_H_11_	Me	**3ad**	88
4	Bn	Me	**3ae**	94
5	Ph	Et	**3af**	77
6	4-MeOC_6_H_4_	Et	**3ag**	77
7	4-BrC_6_H_4_	Et	**3ah**	86
8	4-NO_2_C_6_H_4_	Et	**3ai**	80
9	2-MeC_6_H_4_	Et	**3aj**	67
10	2-BrC_6_H_4_	Et	**3ak**	60
11	1-Naphthyl	Me	**3al**	74
12	2-Furyl	Me	**3am**	70
13	CO_2_Et	Et	**3an**	79
14	CF_3_	Et	**3ao**	41
15	H	Et	**3ap**	47

^*a*^The reaction was performed on a 0.2 mmol scale according to the standard conditions described in Table 1.

**Scheme 4:**
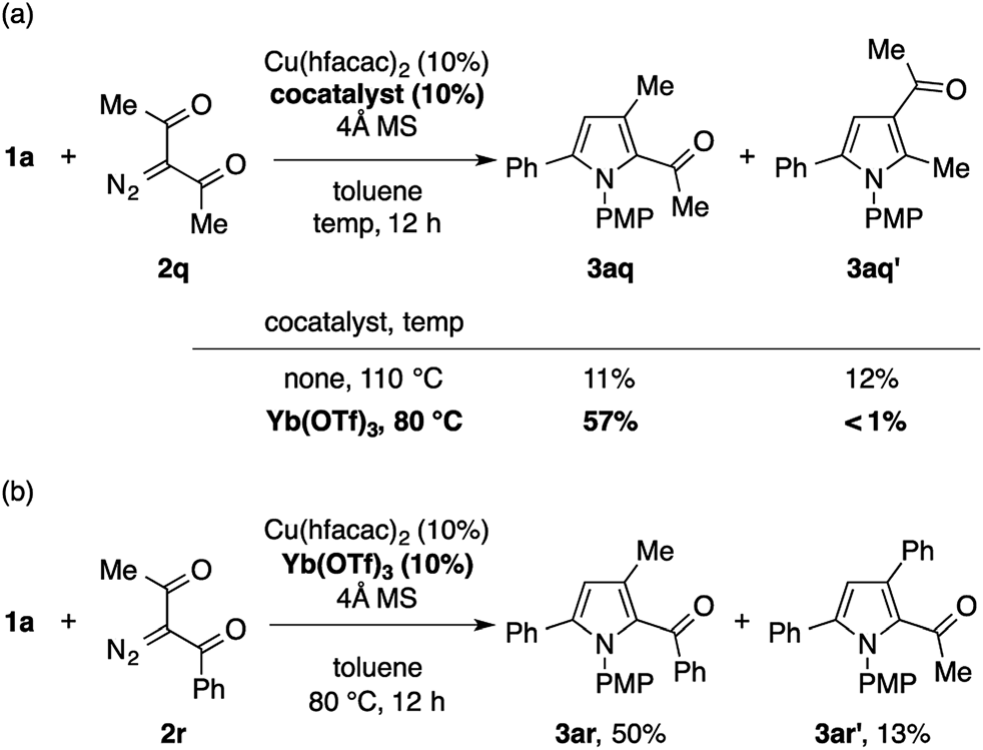
Condensation of **1a** with α-diazo-β-diketones using a Yb(OTf)_3_ cocatalyst.

The reaction of **1a** with 3-diazopentane-2,4-dione **2q** under the standard conditions with Cu(tfacac)_2_ did not give the desired pyrrole product. The use of Cu(hfacac)_2_ instead of Cu(tfacac)_2_ promoted the reaction, but produced a *ca.* 1 : 1 mixture of the expected pyrrole **3aq** and its positional isomer **3aq′** in a low overall yield (Scheme 4a). The latter isomer would be formed through the formal C–H insertion of a copper carbenoid into the α-position of **1a**. Interestingly, the efficiency and the chemoselectivity of this reaction were substantially improved by adding Yb(OTf)_3_ (10 mol%) and lowering the temperature to 80 °C.^17^ Thus, the 2-acetylpyrrole isomer **3aq** was obtained almost exclusively in a respectable yield of 57%. The Cu/Yb cocatalytic system also effected the condensation of **1a** with 1-diazo-1-benzoylacetone **2r** in the same mode of cyclization, in favor of the 2-benzoylpyrrole isomer **3ar** over the 2-acetylpyrrole isomer **3ar′** (Scheme 4b). Note that the same reaction in the absence of Yb(OTf)_3_ produced a mixture of four isomers, including **3ar** and **3ar′** in a low overall yield, as suggested by the GC analysis of the crude product. While the role of Yb(OTf)_3_ is not clear, we speculate that it serves as a Lewis acid to the diketo moiety to enhance the electrophilicity of the copper carbenoid. Note that the α-diazoketones, such as 2-diazo-1,2-diphenylethanone, decomposed too quickly not only under the standard conditions, but also under conditions employing the less reactive Cu(acac)_2_ at a lower temperature, hence producing none of the desired pyrrole product.

Having established the scope and limitation with respect to imines and diazo compounds, we became interested in the applicability of the present condensation reaction to the synthesis of bioactive natural or unnatural products. In this connection, the successful reactions of the dihydroisoquinolines **1aa** and **1ab** (Table 3) turned our attention to the lamellarin family of natural products, many members of which contain a (5,6-dihydro)pyrrolo[2,1-*a*]isoquinoline skeleton. Since the early studies of Steglich, Ishibashi/Iwao, and Banwell,^18^ lamellarins have been popular synthetic targets due to the broad spectrum of their biological activities, and have also served as touchstones for new methods for the construction and the functionalization of pyrrole rings.9b–c,^19^ As some of the completed lamellarin syntheses involve 3-alkoxycarbonyl-5,6-dihydropyrrolo[2,1-*a*]isoquinoline derivatives as key intermediates, we wondered whether the present reaction offers an alternative and efficient route to such intermediates.

**Scheme 5:**
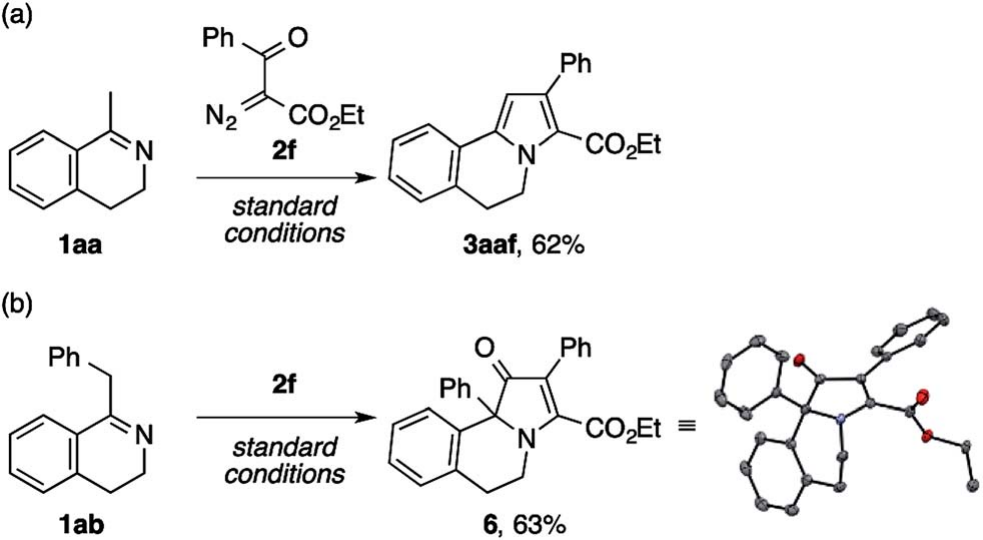
Model reactions of the dihydroisoquinolines **1aa** and **1ab** with ethyl benzoyldiazoacetate **2f**.

Aiming at a straightforward access to the polyarylated pyrrole structure of lamellarins, we first tested model reactions of methyl- and benzyldihydroisoquinolines **1aa** and **1ab** with ethyl benzoyldiazoacetate **2f** (Scheme 5). The reaction of the former imine successfully furnished the desired pyrrole scaffold **3aaf** in 62% yield (Scheme 5a). By contrast, the latter exclusively afforded a dihydrobenz[*g*]indolizinone derivative **6**,^16^ the structure of which was unambiguously confirmed by X-ray crystallographic analysis (Scheme 5b).

**Scheme 6:**
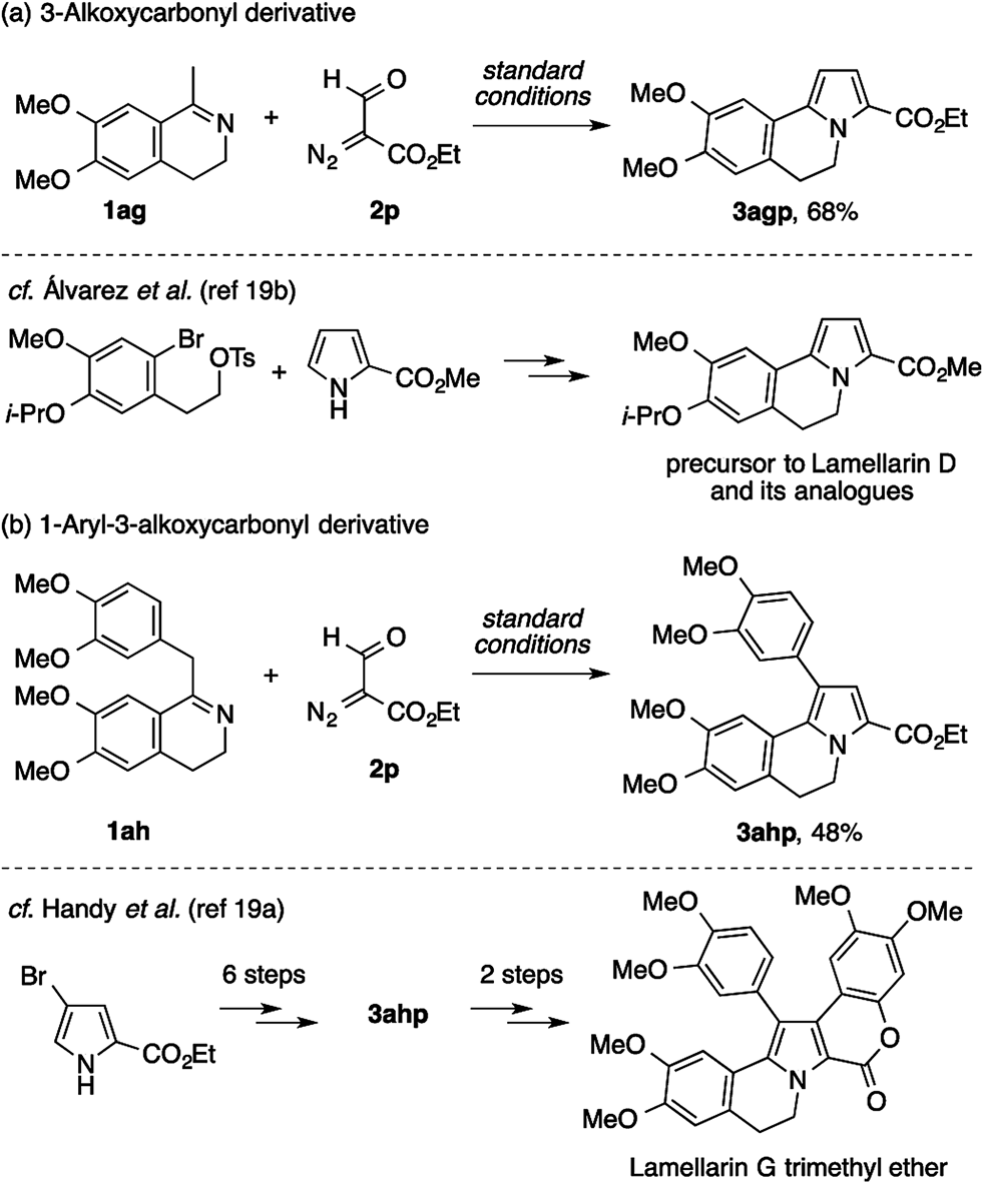
Construction of the 5,6-dihydropyrrolo[2,1-*a*]isoquinoline scaffolds for lamellarin synthesis.

While the above results suggest that the present method may not be suitable for the direct assembly of the pentasubstituted pyrrole core of lamellarins, attempts using ethyl formyldiazoacetate **2p** have proven its utility in the preparation of building blocks for the modular synthesis of lamellarins (Scheme 6). The reaction of methyldihydroisoquinoline with two methoxy groups (**1ag**) and **2p** cleanly afforded the 3-ethoxycarbonyl-5,6-dihydropyrrolo[2,1-*a*]isoquinoline derivative **3agp** (Scheme 6a). An analogous compound of **3agp** was previously synthesized through a sequence of pyrrole *N*-alkylation and an intramolecular Heck reaction, and used as an intermediate to lamellarin D and its analogues.19b,^20^ Furthermore, benzyldihydroisoquinoline bearing four methoxy groups (**1ah**) also underwent condensation with **2p** to afford the 1-aryl-3-ethoxycarbonyl-5,6-dihydropyrrolo[2,1-*a*]isoquinoline derivative **3ahp** (Scheme 6b). In a previous study by Handy *et al.*, **3ahp** was prepared through the six-step manipulation of a pyrrole-based starting material, and was then converted to lamellarin G trimethyl ether in two steps.19*a*

**Scheme 7:**
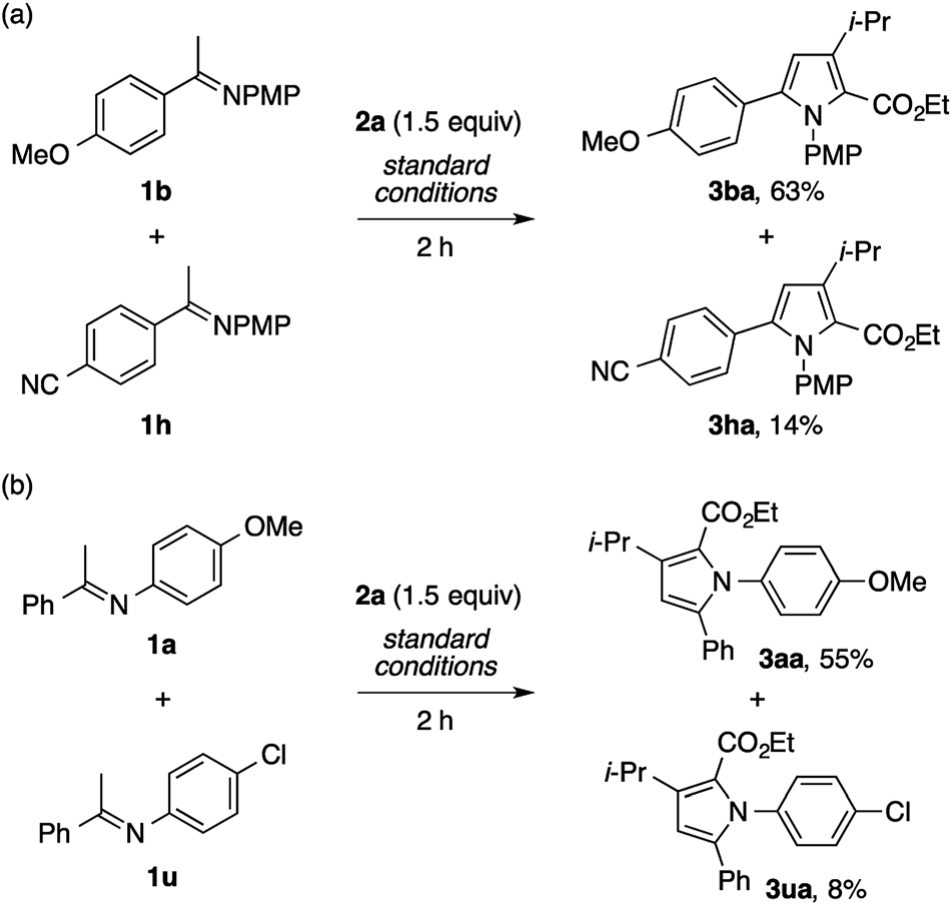
Competition experiments.

On the basis of the regiochemistry of the pyrrole products as well as common reaction patterns in the transition metal catalysis of diazo compounds, the present reaction is considered to involve the addition of the imine nitrogen to a copper carbenoid.^2^,^3^,4g To probe the nature of this putative step, competition experiments using imines bearing different substituents were performed. The reaction of a mixture of the imines **1b** and **1h**, derived from electron-rich and electron-poor acetophenones, respectively, with **2a** afforded the product of the former (**3ba**) as the dominant product (Scheme 7a). Likewise, the reaction of a mixture of the imines **1a** and **1u**, derived from electron-rich and electron-poor anilines, respectively, preferentially produced the product of the former (Scheme 7b). Thus, the nucleophilicity of the imine nitrogen atom would play a critical role in its reaction with the copper carbenoid.

**Scheme 8:**
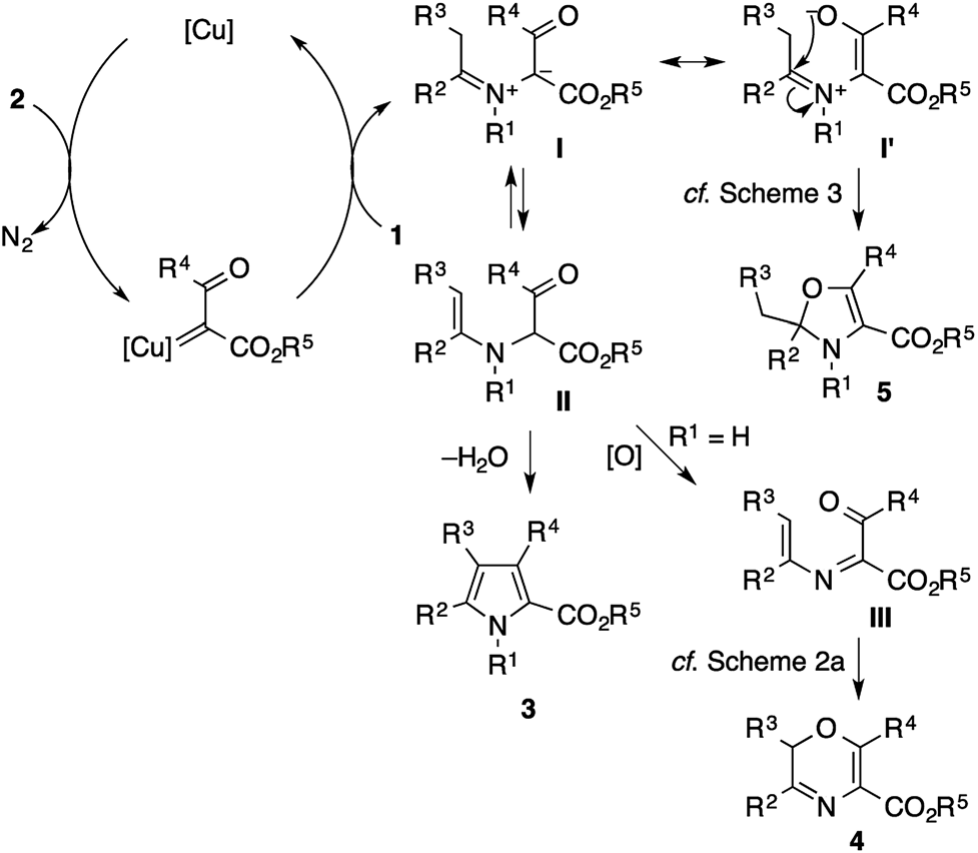
Plausible reaction pathways leading to pyrrole and other byproducts.

Scheme 8 shows plausible reaction pathways of the present pyrrole synthesis. The decomposition of the diazo compound **2** with the copper catalyst generates an electrophilic copper carbenoid. Nucleophilic attack of the imine **1** to the carbenoid gives azomethine ylide **I**^2^–^6^ and regenerates the copper catalyst. The tautomerization of **I** to α-enaminoketone **II***via* proton transfer is followed by cyclocondensation to afford the pyrrole product **3**. Attempts to intercept the azomethine ylide **I** with typical dipolarophiles such as dimethyl acetylenedicarboxylate, dimethyl maleate, and *N*-phenylphthalimide failed to give the corresponding [3 + 2] cycloadducts, but exclusively afforded the pyrrole product, presumably because of the rapid tautomerization of **I** to **II**. Nevertheless, the intermediacy of **I** and **II** rationalizes not only the formation of the pyrrole **3**, but also the side reactions observed in the present study. In the reaction of the N–H imine (Scheme 2), the intermediate **II** may be oxidized to the 1-acyl-2-azadiene **III**, which would then undergo a 6π electrocyclization to afford a 2*H*-1,4-oxazine derivative **4**.^21^ The azomethine ylide **I** may also behave as an enolate/iminium bifunctional species **I′**, which undergoes an intramolecular attack of the enolate oxygen to the iminium moiety to afford the 2,3-dihydrooxazole **5**. This side reaction would be operative in the cases shown in Scheme 3, possibly because of the lower acidity of the α-proton of the iminium moiety (for **1ae**) or the increased electrophilicity of the iminium moiety (for **1af**).

## Conclusions

In conclusion, we have developed a copper-catalyzed condensation reaction of imines and α-diazo-β-dicarbonyl compounds to afford multisubstituted pyrroles in a regiocontrolled manner. The reaction features a broad scope and the ready availability of starting materials and a simple operation, thus enabling the modular and quick preparation of a variety of densely functionalized pyrroles. Given the extensive previous studies on carbenoid reactions with imines,^2^–^6^ it is rather surprising that the present condensation of enolizable imines has not been documented. The reaction opens concise preparative routes to lamellarin scaffolds, and may find further applications in the synthesis of pyrrole alkaloids. The cooperative effect of the carbene transfer catalyst (Cu(hfacac)_2_) and the Lewis acid catalyst (Yb(OTf)_3_), observed in the reaction of α-diazo-β-diketones, also deserves mechanistic and synthetic explorations. Further studies on heterocycle synthesis using imines as starting materials^8^ and/or based on carbenoid chemistry are currently in progress in our laboratory.

## Supplementary Material

Supplementary information

Crystal structure data
