# Supplementary material for: Copper-catalyzed condensation of imines and α-diazo-β-dicarbonyl compounds: modular and regiocontrolled synthesis of multisubstituted pyrroles
Source: Chem Sci. 2015 Aug 3;6(11):6448–55. doi: 10.1039/c5sc02322j (PMC6054072; doi:10.1039/c5sc02322j)

Electronic Supplementary Material (ESI) for Chemical Science.
This journal is © The Royal Society of Chemistry 2015

Electronic Supplementary Information

Copper-Catalyzed Condensation of Imines and α-Diazo-β-dicarbonyl Compounds: Modular and Regiocontrolled Synthesis of Multisubstituted Pyrroles

Wei Wen Tan and Naohiko Yoshikai

Division of Chemistry and Biological Chemistry, School of Physical and Mathematical Sciences, Nanyang Technological University, Singapore 637371

Contents

Materials and Methods....S2

Preparation of Starting Materials....S3

Cu-Catalyzed Condensation of Imines and α-Diazo-β-ketoesters....S6

Cu/Yb-Catalyzed Condensation of Imine with α-Diazo-β-diketone....S30

Application to Lamellarin Scaffolds....S33

References....S35

^{1}H and ^{13}C NMR Spectra....S37

Materials and Methods

General. All reactions dealing with air- and moisture-sensitive compounds were carried out in oven dried reaction vessels under a nitrogen atmosphere. Analytical thin-layer chromatography (TLC) was performed on Merck 60 F254 silica gel plates. ^{1}H and ^{13}C nuclear magnetic resonance (NMR) spectra were recorded on Bruker 400 MHz NMR spectrometers. ^{1}H and ^{13}C NMR spectra are reported in parts per million (ppm) downfield from an internal standard, tetramethylsilane (0.00 ppm) and $CHCl_3$ (77.26 ppm), respectively. Chemical shifts are reported in ppm and the multiplicities are reflected by s (singlet), d (doublet), q (quartet), sept (septet) and m (multiplet). Coupling constants are represented by *J* in hertz (Hz). Gas chromatographic (GC) analysis was performed on a Shimadzu GC-2010 system equipped with FID detector and a capillary column, DB-5 (Agilent J&W, 0.25 mm i.d. x 30 m, 0.25 μm film thickness). High-resolution mass spectra (HRMS) were obtained with a Q-Tof Premier LC HR mass spectrometer. Melting points were determined using a capillary melting point apparatus and are uncorrected.

Materials. Unless otherwise noted, materials were purchased from Aldrich, Alfa Aesar, and other commercial suppliers and were used as received. Copper(II) trifluoroacetylacetonate and copper(II) hexafluoroacetylacetonate were purchased from Alfa Aesar and Strem Chemicals, respectively, and were used as received. Anhydrous toluene used was distilled over calcium hydride (CaH_2) and stored under N_2.

Preparation of Starting Materials

Synthesis of Imines

All imines except for **1aa, 1ab, 1ac, 1ag, 1ah** were synthesized by condensation of the corresponding anilines and ketones according to the literature procedures,[1] and purified by recrystallization from EtOAc/hexane or distillation under reduced pressure. Below is the summarized characterization data for newly synthesized imine. ^{1}H and ^{13}C NMR spectral data for the rest of the imines showed good agreement with literature data.[2] Imines **1aa, 1ab, 1ac, 1ad, 1ag, 1ah** were prepared according to the literature procedures.[3,4]

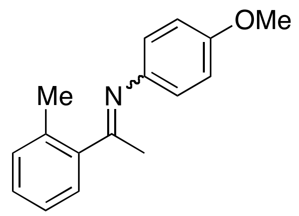

4-Methoxy-*N*-(1-(*o*-tolyl)ethylidene)aniline (1j): Red oil (68% yield, *E*/*Z* = 3:2); **^{1}H NMR** (400 MHz, $CDCl_3$, *E*-isomer): δ 7.37–7.35 (m, 1H), 7.26–7.22 (m, 2H), 7.06–6.99 (m, 1H), 6.91 (d, *J* = 8.9 Hz, 2H), 6.80 (d, *J* = 8.9 Hz, 2H), 3.80 (s, 3H), 2.48 (s, 3H), 2.16 (s, 3H); **^{13}C NMR** (100 MHz, $CDCl_3$, *E*-isomer): δ 170.3, 156.2, 144.5, 142.0, 135.0, 131.2, 128.7, 127.3, 125.95, 120.8, 114.5, 55.6, 21.3, 20.3; **HRMS** (ESI) Calcd for $C_{16}H_{18}NO$ $[M + H]^+$ 240.1388, found 240.1383.

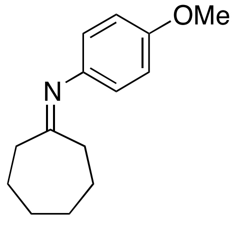

***N*-Cycloheptylidene-4-methoxyaniline (1t):** Orange oil (66% yield); **^{1}H NMR** (400 MHz, $CDCl_3$) δ 6.84 (d, *J* = 8.8 Hz, 2H), 6.63 (d, *J* = 8.8 Hz, 2H), 3.77 (s, 3H), 2.63–2.60 (m, 2H), 2.39–2.23 (m, 2H), 1.78–1.74 (m, 2H), 1.67–1.54 (m, 6H); **^{13}C NMR** (100 MHz, $CDCl_3$) δ 178.5, 155.6, 144.7, 120.4, 114.3, 55.5, 40.7, 33.4, 30.3, 30.0, 26.9, 25.5; **HRMS** (ESI) Calcd for $C_{14}H_{20}NO$ $[M + H]^+$ 218.1545, found 218.1544.

Synthesis of Diazocarbonyl Compounds

General Method. To a 100 mL round bottom flask charged with ketoester (10 mmol) and 4-acetamidobenzenesulfonyl azide (2.64 g, 11 mmol) in acetonitrile (40 mL) was added

triethylamine (4.2 mL, 30 mmol) dropwise at 0 °C. Upon stirring at room temperature for 14 h, the reaction mixture was concentrated under reduced pressure. The residual solid was triturated with ether/light petroleum ether. The mixture was filtered through a pad of Celite, and the filtrate was concentrated under reduced pressure. Purification by flash chromatography afforded the corresponding diazocarbonyl compound.

Below are summarized characterization data for newly synthesized diazocarbonyl compounds. ^{1}H and ^{13}C NMR spectral data for the rest of the diazocarbonyl compound showed good agreement with literature data.[5]

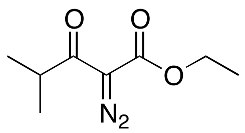

Ethyl 2-diazo-4-methyl-3-oxopentanoate (2a): Yellow oil (96% yield, eluent = hexane/EtOAc (90:10)); **^{1}H NMR** (400 MHz, $CDCl_3$) δ 4.30 (q, *J* = 7.1 Hz, 2H), 3.58 (sept, *J* = 6.8 Hz, 1H), 1.34 (t, *J* = 7.1 Hz, 3H), 1.13 (d, *J* = 6.8 Hz, 6H); **^{13}C NMR** (100 MHz, $CDCl_3$) δ 197.2, 161.4, 61.5, 37.0, 18.7, 14.5; **HRMS** (ESI) Calcd for $C_8H_{13}N_2O_3$ $[M + H]^+$ 185.0926, found 185.0924.

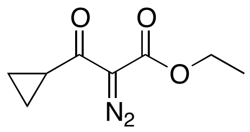

Ethyl 3-cyclopropyl-2-diazo-3-oxopropanoate (2c): Yellow oil (95% yield, eluent = hexane/EtOAc (90:10)); ^{1}H NMR (400 MHz, $CDCl_3$) δ 4.33 (q, *J* = 7.1 Hz, 2H), 3.10 (tt, *J* = 7.9, 4.6 Hz, 1H), 1.35 (t, *J* = 7.1 Hz, 3H), 1.25–1.14 (m, 2H), 1.08–0.92 (m, 2H); **^{13}C NMR** (100 MHz, $CDCl_3$) δ 192.6, 162.0, 61.6, 17.9, 14.5, 12.0; **HRMS** (ESI) Calcd for $C_8H_{11}N_2O_3$ $[M + H]^+$ 183.0770, found 183.0772.

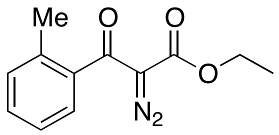

Ethyl 2-diazo-3-oxo-3-(*o*-tolyl)propanoate (2j): Yellow oil (64% yield, eluent = hexane/EtOAc (90:10)); **^{1}H NMR** (400 MHz, $CDCl_3$): δ 7.39–7.29 (m, 1H), 7.28–7.18 (m, 3H), 4.17 (q, *J* = 7.1 Hz, 2H), 2.35 (s, 3H), 1.17 (t, *J* = 7.1 Hz, 3H); **^{13}C NMR** (100 MHz,

$CDCl_3$): δ 188.9, 160.6, 138.0, 135.1, 130.5, 130.3, 126.6, 125.3, 61.6, 19.2, 14.1; **HRMS** (ESI) Calcd for $C_{12}H_{13}N_2O_3$ $[M + H]^+$ 233.0926, found 233.0921.

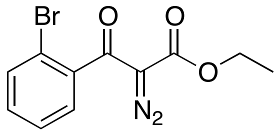

Ethyl 3-(2-bromophenyl)-2-diazo-3-oxopropanoate (2k): Yellow oil (72% yield, eluent = hexane/EtOAc (90:10)); **^{1}H NMR** (400 MHz, $CDCl_3$): δ 7.58 (dd, J = 7.9, 1.0 Hz, 1H), 7.39 (td, J = 7.5, 1.1 Hz, 1H), 7.32 (td, J = 7.7, 1.8 Hz, 1H), 7.27 (dd, J = 7.5, 1.7 Hz, 1H), 4.18 (q, J = 7.1 Hz, 2H), 1.16 (t, J = 7.1 Hz, 3H); **^{13}C NMR** (100 MHz, $CDCl_3$): δ 186.7, 160.32, 140.0, 132.5, 131.2, 127.8, 127.3, 119.0, 61.7, 14.0; **HRMS** (ESI) Calcd for $C_{11}H_{10}BrN_2O_3$ $[M + H]^+$ 296.9875, found 296.9877.

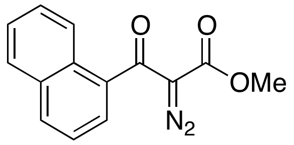

Methyl 2-diazo-3-(naphthalen-1-yl)-3-oxopropanoate (2l): Yellow oil (74% yield, eluent = hexane/EtOAc (90:10)); **^{1}H NMR** (400 MHz, $CDCl_3$): δ 8.06–7.98 (m, 1H), 7.92 (d, J = 8.0 Hz, 1H), 7.85 (dd, J = 6.7, 2.6 Hz, 1H), 7.57–7.38 (m, 4H), 3.63 (s, 3H); **^{13}C NMR** (100 MHz, $CDCl_3$): δ 187.7, 160.9, 135.2, 133.5, 131.3, 129.9, 128.6, 127.4, 126.4, 125.8, 124.5, 124.4, 52.4; **HRMS** (ESI) Calcd for $C_{14}H_{11}N_2O_3$ $[M + H]^+$ 255.0770, found 255.0764.

Cu-Catalyzed Condensation of Imines and α-Diazo-β-ketoesters

General Procedure: A 10 mL Schlenk tube equipped with a stirrer bar was charged with imine (0.20 mmol), $Cu(tfacac)_2$ (7.4 mg, 0.020 mmol, 10 mol%), 4Å molecular sieves (300 mg), and diazocarbonyl compound (0.30 mmol), followed by the addition of toluene (0.5 mL). The resulting mixture was stirred at 110 °C for 12 h. Upon cooling to room temperature, the reaction mixture was diluted with ethyl acetate (5 mL), followed by filtration through a pad of silica gel with ethyl acetate (20 mL) as an eluent. The filtrate was concentrated under reduced pressure, and the residue was purified by flash chromatography on silica gel to afford the pyrrole product.

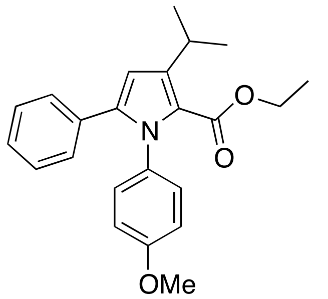

Ethyl 3-isopropyl-1-(4-methoxyphenyl)-5-phenyl-1*H*-pyrrole-2-carboxylate (3aa): The reaction was perfromed on a 5 mmol scale in a 100 mL 2-necked flask, following the same reaction stoichiometry and conditions as that of the general procedure. Yellow solid (92% yield, eluent = hexane/EtOAc (95:5)); The regiochemistry was confirmed by 2D NMR (HMQC and HMBC) analysis (see the attached spectra); Mp = 108–109 °C; **^{1}H NMR** (400 MHz, $CDCl_3$) δ 7.16–7.14 (m, 3H), 7.11–7.06 (m, 4H), 6.83–6.78 (m, 2H), 6.35 (s, 1H), 4.11 (q, J = 7.1 Hz, 2H), 3.79 (s, 3H), 3.61 (sept, J = 6.8 Hz, 1H), 1.30 (d, J = 6.9 Hz, 6H), 1.12 (t, J = 7.1 Hz, 3H); **^{13}C NMR** (100 MHz, $CDCl_3$) δ 161.7, 159.0, 141.9, 140.1, 133.2, 132.7, 129.8, 129.1, 128.2, 127.3, 121.4, 113.6, 108.5, 59.7, 55.6, 26.4, 24.2, 14.3; **HRMS** (ESI) Calcd for $C_{23}H_{26}NO_3$ $[M + H]^+$ 364.1913, found 364.1908.

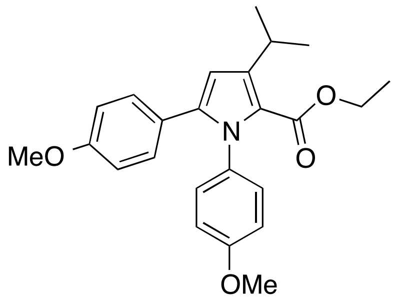

Ethyl 3-isopropyl-1,5-bis(4-methoxyphenyl)-1*H*-pyrrole-2-carboxylate (3ba): Brown solid (84% yield, eluent = hexane/EtOAc (95:5)); Mp = 95–97 °C; **^{1}H NMR** (400 MHz, $CDCl_3$): δ 7.12–7.04 (m, 2H), 7.04–6.99 (m, 2H), 6.85–6.78 (m, 2H), 6.73–6.66 (m, 2H), 6.29 (s, 1H), 4.10 (q, J = 7.1 Hz, 2H), 3.80 (s, 3H), 3.74 (s, 3H), 3.61 (sept, J = 6.8 Hz, 1H), 1.29 (d, J = 6.9 Hz, 6H), 1.12 (t, J = 7.1 Hz, 3H); **^{13}C NMR** (100 MHz, $CDCl_3$): δ 161.7, 158.9, 141.9, 140.1, 133.4, 130.4, 129.9, 125.2, 121.0, 113.7, 113.6, 107.9, 59.7, 55.6, 55.4, 26.4, 24.2, 14.3; **HRMS** (ESI) Calcd for $C_{24}H_{28}NO_4$ $[M + H]^+$ 394.2018, found 394.2015.

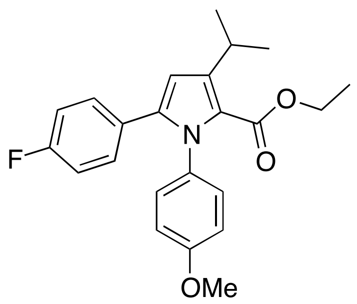

Ethyl 5-(4-fluorophenyl)-3-isopropyl-1-(4-methoxyphenyl)-1*H*-pyrrole-2-carboxylate (3ca): Yellow solid (85% yield, eluent = hexane/EtOAc (95:5)); Mp = 117–118 °C; **^{1}H NMR** (400 MHz, $CDCl_3$): δ 7.12–6.99 (m, 4H), 6.95–6.76 (m, 4H), 6.31 (s, 1H), 4.11 (q, J = 7.1 Hz, 2H), 3.79 (s, 3H), 3.61 (sept, J = 6.9 Hz, 1H), 1.29 (d, J = 6.9 Hz, 6H), 1.12 (t, J = 7.1 Hz, 3H); **^{13}C NMR** (100 MHz, $CDCl_3$): δ 162.1 (d, $J_{C\text{-}F}$ = 245.8 Hz), 161.6, 159.0, 141.8, 139.1, 133.0, 130.8 (d, $J_{C\text{-}F}$ = 8.0 Hz), 129.8, 128.8 (d, $J_{C\text{-}F}$ = 3.4 Hz), 121.4, 115.2 (d, $J_{C\text{-}F}$ = 21.5 Hz), 113.7, 108.4, 59.8, 55.6, 26.3, 24.1, 14.3; **HRMS** (ESI) Calcd for $C_{23}H_{25}FNO_3$ $[M + H]^+$ 382.1818, found 382.1815.

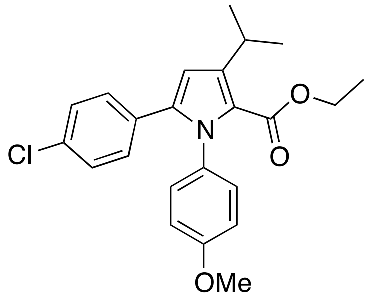

Ethyl 5-(4-chlorophenyl)-3-isopropyl-1-(4-methoxyphenyl)-1*H*-pyrrole-2-carboxylate (3da): Yellow solid (80% yield, eluent = hexane/EtOAc (95:5)); Mp = 119–120 °C; **^{1}H NMR** (400 MHz, $CDCl_3$) δ 7.13 (d, J = 8.6 Hz, 2H), 7.07 (d, J = 8.8 Hz, 2H), 7.01 (d, J = 8.5 Hz, 2H), 6.82 (d, J = 8.8 Hz, 2H), 6.34 (s, 1H), 4.11 (q, J = 7.1 Hz, 2H), 3.80 (s, 3H), 3.60 (sept, J = 6.9 Hz, 1H), 1.29 (d, J = 6.9 Hz, 6H), 1.12 (t, J = 7.1 Hz, 3H); **^{13}C NMR** (100 MHz,

$CDCl_3$) δ 161.6, 159.1, 141.8, 138.8, 133.3, 132.9, 131.1, 130.3, 129.8, 128.4, 121.8, 113.7, 108.6, 59.8, 55.6, 26.3, 24.2, 14.3; **HRMS** (ESI) Calcd for $C_{23}H_{25}ClNO_3$ $[M + H]^+$ 398.1523, found 398.1526.

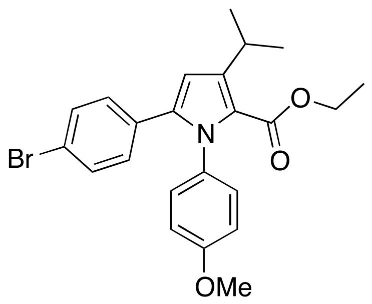

Ethyl 5-(4-bromophenyl)-3-isopropyl-1-(4-methoxyphenyl)-1*H*-pyrrole-2-carboxylate (3ea): Yellow solid (72% yield, eluent = hexane/EtOAc (95:5)); Mp = 115–117 °C; **^{1}H NMR** (400 MHz, $CDCl_3$): δ 7.28 (d, *J* = 8.5 Hz, 2H), 7.06 (d, *J* = 8.8 Hz, 2H), 6.95 (d, *J* = 8.5 Hz, 2H), 6.82 (d, *J* = 8.8 Hz, 2H), 6.34 (s, 1H), 4.10 (q, *J* = 7.1 Hz, 2H), 3.80 (s, 3H), 3.60 (sept, *J* = 6.8 Hz, 1H), 1.29 (d, *J* = 6.9 Hz, 6H), 1.12 (t, *J* = 7.1 Hz, 3H); **^{13}C NMR** (100 MHz, $CDCl_3$): δ 161.6, 159.1, 141.8, 138.7, 132.9, 131.6, 131.4, 130.5, 129.8, 121.8, 121.5, 113.7, 108.6, 59.8, 55.6, 26.3, 24.1, 14.3; **HRMS** (ESI) Calcd for $C_{23}H_{25}BrNO_3$ $[M + H]^+$ 442.1018, found 442.1023.

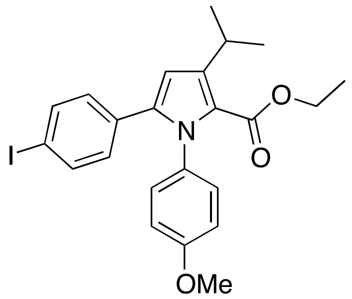

Ethyl 5-(4-iodophenyl)-3-isopropyl-1-(4-methoxyphenyl)-1*H*-pyrrole-2-carboxylate (3fa): Yellow solid (71% yield, eluent = hexane/EtOAc (95:5)); Mp = 103–104 °C; **^{1}H NMR** (400 MHz, $CDCl_3$) δ 7.48 (d, *J* = 8.0 Hz, 2H), 7.07 (d, *J* = 8.3 Hz, 2H), 6.83–6.81 (m, 4H), 6.34 (s, 1H), 4.10 (q, *J* = 7.1 Hz, 2H), 3.81 (s, 3H), 3.59 (sept, *J* = 6.5 Hz, 1H), 1.29 (d, *J* = 6.8 Hz, 6H), 1.12 (t, *J* = 7.1 Hz, 3H); **^{13}C NMR** (100 MHz, $CDCl_3$): δ 161.6, 159.1, 141.8, 138.8, 137.3, 132.9, 132.2, 130.7, 129.8, 121.9, 113.8, 108.6, 93.2, 59.9, 55.6, 26.3, 24.2, 14.3; **HRMS** (ESI) Calcd for $C_{23}H_{25}INO_3$ $[M + H]^+$ 490.0879, found 490.0877.

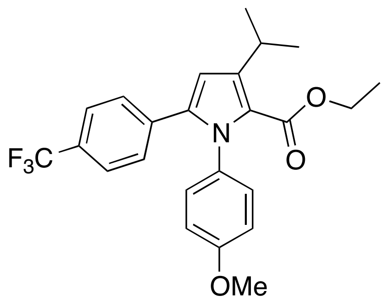

Ethyl 3-isopropyl-1-(4-methoxyphenyl)-5-(4-(trifluoromethyl)phenyl)-1*H*-pyrrole-2-carboxylate (3ga): Yellow solid (72% yield, eluent = hexane/EtOAc (97:3)); Mp = 94–95 °C; **^{1}H NMR** (400 MHz, $CDCl_3$): δ 7.41 (d, J = 8.3 Hz, 2H), 7.19 (d, J = 8.2 Hz, 2H), 7.08 (d, J = 8.8 Hz, 2H), 6.83 (d, J = 8.8 Hz, 2H), 6.41 (s, 1H), 4.12 (q, J = 7.1 Hz, 2H), 3.82 (s, 3H), 3.60 (sept, J = 6.8 Hz, 1H), 1.30 (d, J = 6.9 Hz, 6H), 1.13 (t, J = 7.1 Hz, 3H); **^{13}C NMR** (100 MHz, $CDCl_3$): δ 161.6, 159.2, 141.8, 138.2, 136.2, 132.7, 129.8, 129.02, 129.02 (q, $J_{C\text{-}F}$ = 33.0 Hz), 125.1 (q, $J_{C\text{-}F}$ = 4.0 Hz), 124.3 (q, $J_{C\text{-}F}$ = 270.0 Hz), 122.4, 113.8, 109.3, 60.0, 55.6, 26.3, 24.1, 14.3; **HRMS** (ESI) Calcd for $C_{24}H_{25}F_3NO_3$ $[M + H]^+$ 432.1787, found 432.1782.

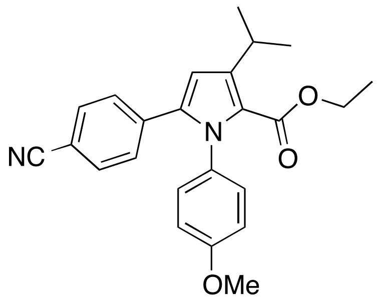

Ethyl 5-(4-cyanophenyl)-3-isopropyl-1-(4-methoxyphenyl)-1*H*-pyrrole-2-carboxylate (3ha): White solid (74% yield, eluent = hexane/EtOAc (90:10)); Mp = 121–123 °C; **^{1}H NMR** (400 MHz, $CDCl_3$): δ 7.44 (d, J = 8.4 Hz, 2H), 7.17 (d, J = 8.4 Hz, 2H), 7.08 (d, J = 8.8 Hz, 2H), 6.84 (d, J = 8.8 Hz, 2H), 6.45 (s, 1H), 4.12 (q, J = 7.1 Hz, 2H), 3.82 (s, 3H), 3.59 (sept, J = 6.8 Hz, 1H), 1.30 (d, J = 6.9 Hz, 6H), 1.13 (t, J = 7.1 Hz, 3H); **^{13}C NMR** (100 MHz, $CDCl_3$): δ 161.5, 159.4, 141.7, 137.6, 137.1, 132.5, 132.0, 129.7, 129.1, 122.9, 119.0, 113.9, 110.5, 109.8, 60.1, 55.6, 26.3, 24.1, 14.2; **HRMS** (ESI) Calcd for $C_{24}H_{25}N_2O_3$ $[M + H]^+$ 389.1865, found 389.1861.

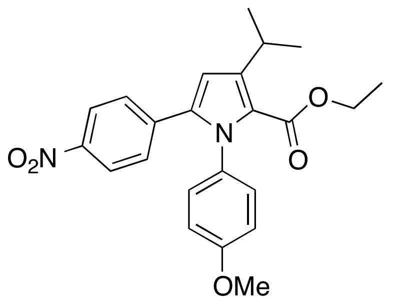

Ethyl 3-isopropyl-1-(4-methoxyphenyl)-5-(4-nitrophenyl)-1*H*-pyrrole-2-carboxylate (3ia): Orange solid (52% yield, eluent = hexane/EtOAc (95:5)); Mp = 106–107 °C; **^{1}H NMR** (400 MHz, $CDCl_3$): δ 8.02 (d, *J* = 8.8 Hz, 2H), 7.23 (d, *J* = 8.8 Hz, 2H), 7.09 (d, *J* = 8.8 Hz, 2H), 6.85 (d, *J* = 8.8 Hz, 2H), 6.50 (s, 1H), 4.13 (q, *J* = 7.1 Hz, 2H), 3.82 (s, 3H), 3.60 (sept, *J* = 6.8 Hz, 1H), 1.30 (d, *J* = 6.8 Hz, 6H), 1.14 (t, *J* = 7.1 Hz, 3H); **^{13}C NMR** (100 MHz, $CDCl_3$): δ 161.5, 159.5, 146.5, 141.8, 139.1, 137.2, 132.5, 129.8, 129.1, 123.6, 123.3, 114.0, 110.2, 60.2, 55.7, 26.3, 24.1, 14.3; **HRMS** (ESI) Calcd for $C_{23}H_{25}N_2O_5$ $[M + H]^+$ 409.1763, found 409.1767.

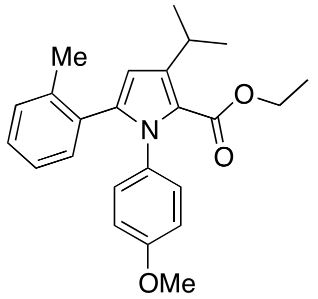

Ethyl 3-isopropyl-1-(4-methoxyphenyl)-5-(*o*-tolyl)-1*H*-pyrrole-2-carboxylate (3ja): Orange solid (88% yield, eluent = hexane/EtOAc (95:5)); Mp = 98–99 °C; **^{1}H NMR** (400 MHz, $CDCl_3$): δ 7.15–7.03 (m, 2H), 7.03–6.87 (m, 4H), 6.69 (d, *J* = 8.8 Hz, 2H), 6.17 (s, 1H), 4.11 (q, *J* = 7.1 Hz, 2H), 3.72 (s, 3H), 3.63 (sept, *J* = 6.8 Hz, 1H), 2.09 (s, 3H), 1.29 (d, *J* = 6.9 Hz, 6H), 1.11 (t, *J* = 7.1 Hz, 3H); **^{13}C NMR** (100 MHz, $CDCl_3$): δ 161.8, 158.5, 141.7, 139.6, 137.9, 133.1, 132.6, 131.7, 129.9, 129.1, 128.2, 125.1, 120.0, 113.3, 108.8, 59.7, 55.5, 26.4, 24.2, 20.6, 14.3; **HRMS** (ESI) Calcd for $C_{24}H_{28}NO_3$ $[M + H]^+$ 378.2069, found 378.2072. Recrystallization from EtOAc/hexane afforded single crystals suitable for X-ray diffraction analysis, which unambiguously confirmed the substitution pattern of **3ja** (see below).[6]

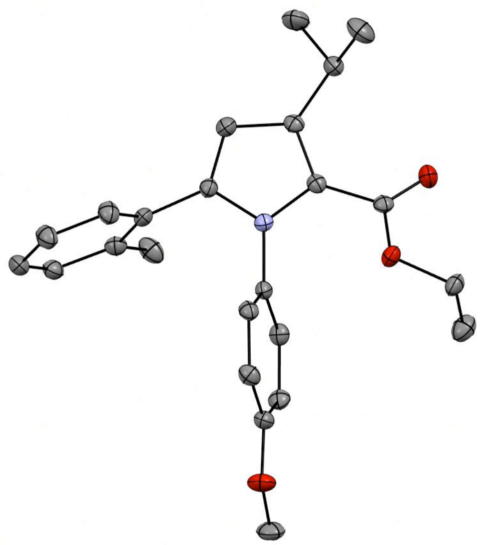

Figure S1. ORTEP diagram of **3ja**. Thermal ellipsoids drawn at 50% probability.

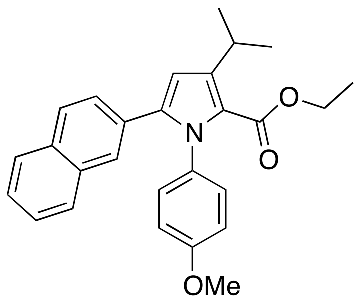

Ethyl 3-isopropyl-1-(4-methoxyphenyl)-5-(naphthalen-2-yl)-1*H*-pyrrole-2-carboxylate (3ka): Yellow solid (87% yield, eluent = hexane/EtOAc (95:5)); Mp = 130–132 °C; **^{1}H NMR** (400 MHz, $CDCl_3$): δ 7.73–7.70 (m, 1H), 7.68–7.64 (m, 1H), 7.64–7.54 (m, 2H), 7.44–7.34 (m, 2H), 7.17–7.09 (m, 3H), 6.80 (d, *J* = 8.8 Hz, 2H), 6.47 (s, 1H), 4.12 (q, *J* = 7.1 Hz, 2H), 3.77 (s, 3H), 3.64 (sept, *J* = 6.8 Hz, 1H), 1.33 (d, *J* = 6.9 Hz, 6H), 1.13 (t, *J* = 7.1 Hz, 3H); **^{13}C NMR** (100 MHz, $CDCl_3$): δ 161.7, 159.0, 142.0, 140.0, 133.3, 133.2, 132.4, 130.2, 129.9, 128.3, 128.2, 127.7, 127.6, 126.9, 126.34, 126.28, 121.6, 113.7, 108.9, 59.8, 55.6, 26.4, 24.2, 14.3; **HRMS** (ESI) Calcd for $C_{27}H_{28}NO_3$ $[M + H]^+$ 414.2069, found 414.2072.

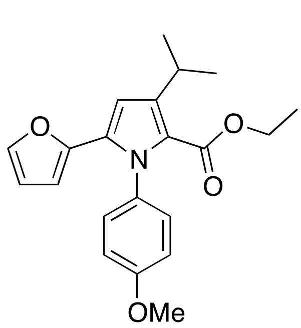

Ethyl 5-(furan-2-yl)-3-isopropyl-1-(4-methoxyphenyl)-1*H*-pyrrole-2-carboxylate (3la): Yellow solid (83% yield, eluent = hexane/EtOAc (95:5)); Mp = 80–82 °C; ^{1}**H NMR** (400 MHz, $CDCl_3$): δ 7.30 (d, *J* = 1.3 Hz, 1H), 7.19 (d, *J* = 8.8 Hz, 2H), 6.95 (d, *J* = 8.8 Hz, 2H), 6.61 (s, 1H), 6.16 (dd, *J* = 3.4, 1.8 Hz, 1H), 5.13 (d, *J* = 3.4 Hz, 1H), 4.09 (q, *J* = 7.1 Hz, 2H), 3.87 (s, 3H), 3.60 (sept, *J* = 6.8 Hz, 1H), 1.29 (d, *J* = 6.9 Hz, 6H), 1.10 (t, *J* = 7.1 Hz, 3H); ^{13}**C NMR** (100 MHz, $CDCl_3$): δ 161.5, 159.7, 147.0, 142.0, 141.6, 133.5, 131.4, 129.6, 121.5, 114.1, 111.4, 106.6, 106.4, 59.7, 55.7, 26.3, 24.1, 14.2; **HRMS** (ESI) Calcd for $C_{21}H_{24}NO_4$ $[M + H]^+$ 354.1705, found 354.1706.

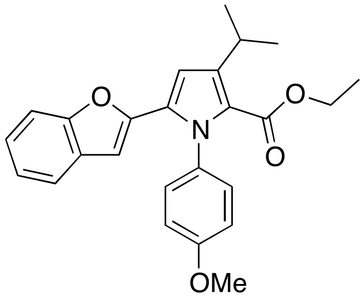

Ethyl 5-(benzofuran-2-yl)-3-isopropyl-1-(4-methoxyphenyl)-1*H*-pyrrole-2-carboxylate (3ma): Yellow solid (87% yield, eluent = hexane/EtOAc (95:5)); Mp = 85–86 °C; ^{1}**H NMR** (400 MHz, $CDCl_3$): δ 7.41 (d, *J* = 8.2 Hz, 1H), 7.32 (d, *J* = 7.5 Hz, 1H), 7.27–7.23 (m, 2H), 7.23–7.17 (m, 1H), 7.11 (t, *J* = 7.4 Hz, 1H), 7.00 (d, *J* = 8.8 Hz, 2H), 6.86 (s, 1H), 5.43 (s, 1H), 4.11 (q, *J* = 7.1 Hz, 2H), 3.90 (s, 3H), 3.62 (sept, *J* = 6.8 Hz, 1H), 1.33 (d, *J* = 6.9 Hz, 6H), 1.12 (t, *J* = 7.1 Hz, 3H); ^{13}**C NMR** (100 MHz, $CDCl_3$): δ 161.4, 159.9, 154.1, 148.6, 141.9, 133.2, 130.6, 129.7, 129.0, 124.6, 123.1, 122.9, 121.2, 114.3, 110.9, 108.4, 102.5, 59.9, 55.7, 26.4, 24.1, 14.2; **HRMS** (ESI) Calcd for $C_{25}H_{26}NO_4$ $[M + H]^+$ 404.1862, found 404.1862.

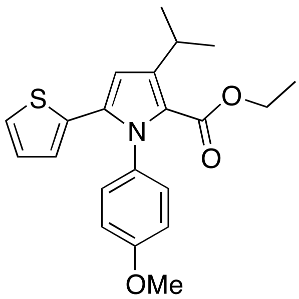

Ethyl 3-isopropyl-1-(4-methoxyphenyl)-5-(thiophen-2-yl)-1*H*-pyrrole-2-carboxylate (3na): Yellow solid (73% yield, eluent = hexane/EtOAc (95:5)); Mp = 73–75 °C; ^{1}**H NMR** (400 MHz, $CDCl_3$): δ 7.18 (d, *J* = 8.8 Hz, 2H), 7.08 (d, *J* = 5.1 Hz, 1H), 6.91 (d, *J* = 8.8 Hz,

2H), 6.84–6.80 (m, 1H), 6.65 (d, *J* = 3.7 Hz, 1H), 6.46 (s, 1H), 4.09 (q, *J* = 7.1 Hz, 2H), 3.85 (s, 3H), 3.60 (sept, *J* = 6.9 Hz, 1H), 1.29 (d, *J* = 6.9 Hz, 6H), 1.11 (t, *J* = 7.1 Hz, 3H); ^{13}**C NMR** (100 MHz, $CDCl_3$): δ 161.4, 159.8, 142.0, 134.4, 134.0, 132.9, 130.3, 127.1, 125.7, 125.4, 121.7, 114.0, 107.9, 59.7, 55.7, 26.3, 24.1, 14.3; **HRMS** (ESI) Calcd for $C_{21}H_{24}NO_3S$ $[M + H]^+$ 370.1477, found 370.1476.

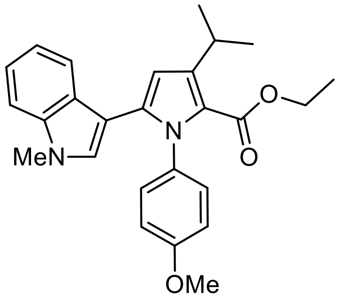

Ethyl 3-isopropyl-1-(4-methoxyphenyl)-5-(1-methyl-1*H*-indol-3-yl)-1*H*-pyrrole-2-carboxylate (3oa): Yellow solid (70% yield, eluent = hexane/EtOAc (95:5)); Mp = 134–135 °C; ^{1}**H NMR** (400 MHz, $CDCl_3$): δ 7.83 (d, *J* = 7.9 Hz, 1H), 7.23 (dd, *J* = 3.4, 1.0 Hz, 2H), 7.21–7.04 (m, 3H), 6.85 (d, *J* = 8.9 Hz, 2H), 6.55 (s, 1H), 6.15 (s, 1H), 4.11 (q, *J* = 7.1 Hz, 2H), 3.81 (s, 3H), 3.67 (sept, *J* = 6.8 Hz, 1H), 3.56 (s, 3H), 1.35 (d, *J* = 6.9 Hz, 6H), 1.12 (t, *J* = 7.1 Hz, 3H); ^{13}**C NMR** (100 MHz, $CDCl_3$): δ 161.7, 159.1, 142.4, 136.5, 134.7, 134.0, 129.9, 127.8, 127.5, 122.3, 120.4, 120.2, 120.0, 113.7, 109.5, 107.6, 107.0, 59.5, 55.6, 33.0, 26.5, 24.2, 14.3; **HRMS** (ESI) Calcd for $C_{26}H_{29}N_2O_3$ $[M + H]^+$ 417.2178, found 417.2177.

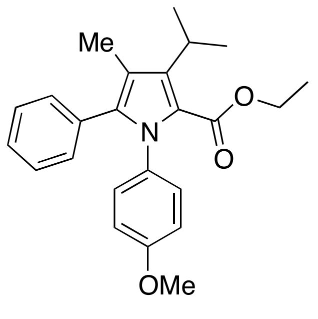

Ethyl 3-isopropyl-1-(4-methoxyphenyl)-4-methyl-5-phenyl-1*H*-pyrrole-2-carboxylate (3pa): Orange solid (77% yield, eluent = hexane/EtOAc (95:5)); Mp = 88–89 °C; ^{1}**H NMR** (400 MHz, $CDCl_3$): δ 7.22–7.14 (m, 3H), 7.03 (dd, *J* = 7.6, 1.9 Hz, 2H), 6.95 (d, *J* = 8.9 Hz, 2H), 6.70 (d, *J* = 8.9 Hz, 2H), 4.03 (q, *J* = 7.1 Hz, 2H), 3.76 (sept, *J* = 6.8 Hz, 1H) 3.74 (s, 3H), 2.07 (s, 3H), 1.38 (d, *J* = 7.2 Hz, 6H), 0.99 (t, *J* = 7.1 Hz, 3H); ^{13}**C NMR** (100 MHz, $CDCl_3$): δ 162.3, 158.5, 138.2, 137.7, 133.6, 132.2, 131.2, 129.5, 127.9, 127.3, 121.0, 116.9,

113.3, 59.8, 55.5, 26.1, 22.2, 14.1, 11.8; **HRMS** (ESI) Calcd for $C_{24}H_{28}NO_3$ $[M + H]^+$ 378.2069, found 378.2069.

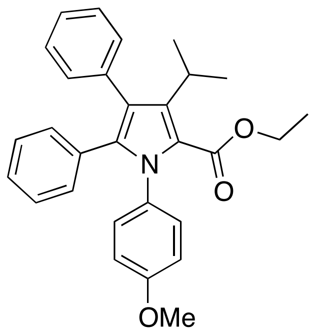

Ethyl 3-isopropyl-1-(4-methoxyphenyl)-4,5-diphenyl-1*H*-pyrrole-2-carboxylate (3qa): Brown solid (76% yield, eluent = hexane/EtOAc (95:5)); Mp = 126–128 °C; ^{1}**H NMR** (400 MHz, $CDCl_3$): δ 7.25–7.13 (m, 5H), 7.07 (d, *J* = 8.8 Hz, 2H), 6.97 (d, *J* = 6.9 Hz, 3H), 6.87 (dd, *J* = 7.4, 1.8 Hz, 2H), 6.73 (d, *J* = 8.8 Hz, 2H), 4.08 (q, *J* = 7.1 Hz, 2H), 3.74 (s, 3H), 3.43 (sept, *J* = 7.1 Hz, 1H), 1.26 (d, *J* = 7.1 Hz, 6H), 1.06 (t, *J* = 7.1 Hz, 3H); ^{13}**C NMR** (100 MHz, $CDCl_3$): δ 162.3, 158.7, 137.6, 137.5, 136.4, 133.2, 131.78, 131.76, 131.3, 129.8, 127.8, 127.5, 127.0, 126.5, 125.0, 121.6, 113.4, 60.1, 55.5, 26.5, 22.9, 14.2; **HRMS** (ESI) Calcd for $C_{29}H_{30}NO_3$ $[M + H]^+$ 440.2226, found 440.2221.

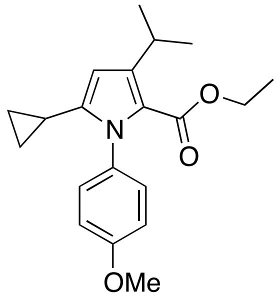

Ethyl 5-cyclopropyl-3-isopropyl-1-(4-methoxyphenyl)-1*H*-pyrrole-2-carboxylate (3ra): Yellow solid (84% yield, eluent = hexane/EtOAc (95:5)); Mp = 72–73 °C; ^{1}**H NMR** (400 MHz, $CDCl_3$): δ 7.18 (d, *J* = 8.8 Hz, 2H), 6.93 (d, *J* = 8.8 Hz, 2H), 5.75 (s, 1H), 4.05 (q, *J* = 7.1 Hz, 2H), 3.84 (s, 3H), 3.56 (sept, *J* = 6.8 Hz, 1H), 1.40 – 1.31 (m, 1H), 1.22 (d, *J* = 6.9 Hz, 6H), 1.07 (t, *J* = 7.1 Hz, 3H), 0.76 – 0.66 (m, 2H), 0.66 – 0.55 (m, 2H); ^{13}**C NMR** (100 MHz, $CDCl_3$): δ 161.4, 159.0, 143.3, 141.9, 133.5, 129.2, 119.7, 113.8, 102.3, 59.3, 55.6, 26.3, 24.1, 14.2, 8.3, 8.0; **HRMS** (ESI) Calcd for $C_{20}H_{26}NO_3$ $[M + H]^+$ 328.1913, found 328.1909.

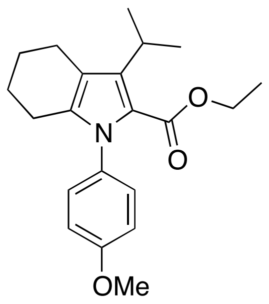

Ethyl 3-isopropyl-1-(4-methoxyphenyl)-4,5,6,7-tetrahydro-1*H*-indole-2-carboxylate (3sa): Yellow oil (73% yield, eluent = hexane/EtOAc (95:5)); **^{1}H NMR** (400 MHz, $CDCl_3$): δ 7.08 (d, *J* = 8.8 Hz, 2H), 6.89 (d, *J* = 8.8 Hz, 2H), 4.00 (q, *J* = 7.1 Hz, 2H), 3.83 (s, 3H), 3.79 (sept, *J* = 6.8 Hz, 1H), 2.64 (t, *J* = 5.4 Hz, 2H), 2.23 (t, *J* = 5.6 Hz, 2H), 1.74–1.69 (m, 4H), 1.30 (d, *J* = 7.1 Hz, 6H), 0.97 (t, *J* = 7.1 Hz, 3H); **^{13}C NMR** (100 MHz, $CDCl_3$): δ 162.0, 158.8, 137.7, 136.5, 133.5, 128.7, 119.3, 117.5, 113.8, 59.3, 55.6, 26.1, 23.8, 23.7, 23.4, 22.9, 22.4, 14.1; **HRMS** (ESI) Calcd for $C_{21}H_{28}NO_3$ $[M + H]^+$ 342.2069, found 342.2068.

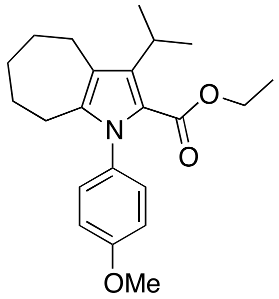

Ethyl 3-isopropyl-1-(4-methoxyphenyl)-1,4,5,6,7,8-hexahydrocyclohepta[*b*]pyrrole-2-carboxylate (3ta): Yellow solid (68% yield, eluent = hexane/EtOAc (95:5)); Mp = 85–87 °C; **^{1}H NMR** (400 MHz, $CDCl_3$): δ 7.06 (d, *J* = 8.7 Hz, 2H), 6.89 (d, *J* = 8.7 Hz, 2H), 3.97 (q, *J* = 7.1 Hz, 2H), 3.83 (s, 3H), 3.82 (sept, *J* = 7.1 Hz, 1H), 2.71–2.68 (m, 2H), 2.40–2.25 (m, 2H), 1.80–1.76 (m, 2H), 1.71–1.60 (m, 2H), 1.53–1.57 (m, 2H), 1.32 (d, *J* = 7.2 Hz, 6H), 0.95 (t, *J* = 7.1 Hz, 3H); **^{13}C NMR** (100 MHz, $CDCl_3$): δ 161.9, 158.7, 140.8, 137.4, 133.5, 129.1, 122.7, 118.3, 113.5, 59.1, 55.4, 32.3, 28.2, 26.9, 26.5, 26.2, 25.1, 22.5, 13.9; **HRMS** (ESI) Calcd for $C_{22}H_{30}NO_3$ $[M + H]^+$ 356.2226, found 356.2227.

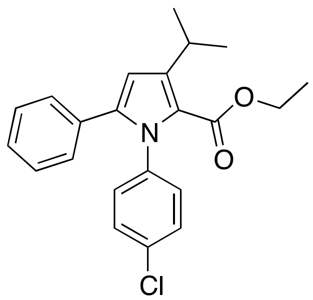

Ethyl 1-(4-chlorophenyl)-3-isopropyl-5-phenyl-1*H*-pyrrole-2-carboxylate (3ua): Orange solid (42% yield, eluent = hexane/EtOAc (95:5)); Mp = 90–91 °C; **^{1}H NMR** (400 MHz, $CDCl_3$): δ 7.29–7.23 (m, 2H), 7.19–7.17 (m, 3H), 7.12–7.03 (m, 4H), 6.36 (s, 1H), 4.11 (q, J = 7.1 Hz, 2H), 3.61 (sept, J = 6.8 Hz, 1H), 1.30 (d, J = 6.9 Hz, 6H), 1.13 (t, J = 7.1 Hz, 3H); **^{13}C NMR** (100 MHz, $CDCl_3$): δ 161.3, 142.2, 139.9, 138.8, 133.4, 132.0, 130.0, 129.0, 128.4, 128.1, 127.4, 121.0, 108.8, 59.7, 26.1, 23.9, 14.0; **HRMS** (ESI) Calcd for $C_{22}H_{23}ClNO_2$ $[M + H]^+$ 368.1417, found 368.1417.

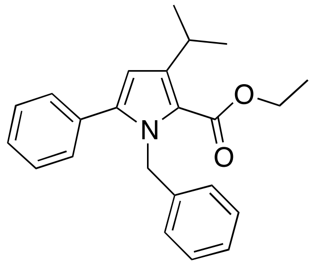

Ethyl 1-benzyl-3-isopropyl-5-phenyl-1*H*-pyrrole-2-carboxylate (3va): Yellow oil (72% yield, eluent = hexane/EtOAc (97:3)); **^{1}H NMR** (400 MHz, $CDCl_3$): δ 7.31 (s, 5H), 7.26–7.20 (m, 2H), 7.16 (t, J = 7.2 Hz, 1H), 6.84 (d, J = 7.2 Hz, 2H), 6.25 (s, 1H), 5.53 (s, 2H), 4.17 (q, J = 7.1 Hz, 2H), 3.60 (sept, J = 6.8 Hz, 1H), 1.26 (d, J = 6.8 Hz, 6H), 1.21 (t, J = 7.1 Hz, 3H); **^{13}C NMR** (100 MHz, $CDCl_3$): δ 162.0, 142.7, 141.2, 140.3, 132.8, 129.6, 128.64, 128.60, 128.3, 126.8, 125.8, 119.4, 108.5, 59.8, 50.0, 26.6, 24.2, 14.4; **HRMS** (ESI) Calcd for $C_{23}H_{26}NO_2$ $[M + H]^+$ 348.1964, found 348.1961.

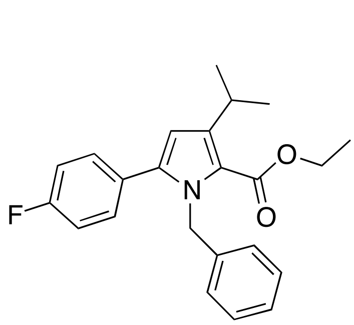

Ethyl 1-benzyl-5-(4-fluorophenyl)-3-isopropyl-1*H*-pyrrole-2-carboxylate (3wa): Yellow oil (68% yield, eluent = hexane/EtOAc (97:3)); **^{1}H NMR** (400 MHz, $CDCl_3$): δ 7.72–7.15 (m, 5H), 7.00 (t, *J* = 8.6 Hz, 2H), 6.82 (d, *J* = 7.4 Hz, 2H), 6.21 (s, 1H), 5.49 (s, 2H), 4.18 (q, *J* = 7.1 Hz, 2H), 3.60 (sept, *J* = 6.8 Hz, 1H), 1.26 (d, *J* = 6.8 Hz, 6H), 1.22 (t, *J* = 7.1 Hz, 3H); **^{13}C NMR** (100 MHz, $CDCl_3$): δ 162.85 (d, $J_{C\text{-}F}$ = 248.1 Hz), 162.0, 142.5, 140.1, 140.0, 131.39 (d, $J_{C\text{-}F}$ = 8.2 Hz), 128.82 (d, $J_{C\text{-}F}$ = 3.3 Hz), 128.7, 126.9, 125.7, 119.5, 115.64 (d, $J_{C\text{-}F}$ = 21.5 Hz), 108.5, 59.9, 49.9, 26.5, 24.1, 14.4; **HRMS** (ESI) Calcd for $C_{23}H_{25}FNO_2$ [M + H]$^+$ 366.1869, found 366.1866.

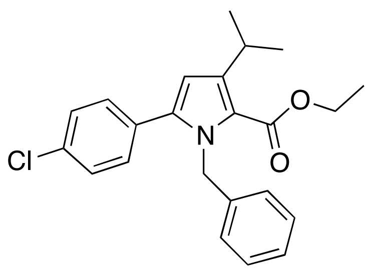

Ethyl 1-benzyl-5-(4-chlorophenyl)-3-isopropyl-1*H*-pyrrole-2-carboxylate (3xa): Yellow oil (72% yield, eluent = hexane/EtOAc (97:3)); **^{1}H NMR** (400 MHz, $CDCl_3$): δ 7.35 – 7.12 (m, 7H), 6.83 (d, *J* = 7.5 Hz, 2H), 6.23 (s, 1H), 5.50 (s, 2H), 4.18 (q, *J* = 7.1 Hz, 2H), 3.60 (sept, *J* = 6.7 Hz, 1H), 1.26 (d, *J* = 6.9 Hz, 6H), 1.22 (t, *J* = 7.1 Hz, 3H); **^{13}C NMR** (100 MHz, $CDCl_3$): δ 162.0, 142.6, 140.0, 139.8, 134.4, 131.2, 130.8, 128.9, 128.7, 127.0, 125.7, 119.8, 108.6, 59.9, 50.0, 26.5, 24.1, 14.4; **HRMS** (ESI) Calcd for $C_{23}H_{25}ClNO_2$ [M + H]$^+$ 382.1574, found 382.1569.

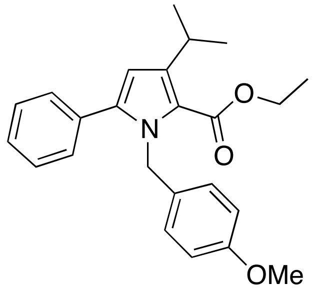

Ethyl 3-isopropyl-1-(4-methoxybenzyl)-5-phenyl-1*H*-pyrrole-2-carboxylate (3ya): Yellow oil (73% yield, eluent = hexane/EtOAc (97:3)); **^{1}H NMR** (400 MHz, $CDCl_3$): δ 7.32 (s, 5H), 6.76 (s, 4H), 6.22 (s, 1H), 5.47 (s, 2H), 4.20 (q, *J* = 7.1 Hz, 2H), 3.74 (s, 3H), 3.59 (sept, *J* = 6.8 Hz, 1H), 1.25 (d, *J* = 6.8 Hz, 6H). 1.25 (t, *J* = 7.1 Hz, 3H); **^{13}C NMR** (100 MHz, $CDCl_3$): δ 162.1, 158.5, 142.6, 141.1, 132.8, 132.3, 129.6, 128.6, 128.2, 127.1, 119.3,

114.0, 108.4, 59.8, 55.4, 49.3, 26.5, 24.2, 14.5; **HRMS** (ESI) Calcd for $C_{24}H_{28}NO_3$ [M + H]$^+$ 378.2069, found 378.2068.

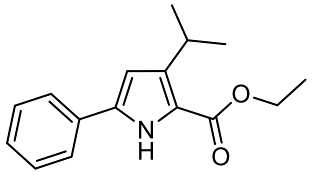

Ethyl 3-isopropyl-5-phenyl-1*H*-pyrrole-2-carboxylate (3ya'): The deprotection of the PMB group from **3za** was performed according to the literature procedure.[7] To a 50 mL oven dried Schlenk tube was added **3za** (56.6 mg, 0.15 mmol), anisole (152 mg, 1.35 mmol) and trifluoroacetic acid (1.5 mL) in dichloromethane (6.0 mL). The resulting mixture was stirred at 37 °C for 40 h. The reaction mixture was concentrated under reduced pressure, and the residue was purified by flash chromatography on silica gel to afford the desired N-H pyrrole. White solid (90% yield, eluent = hexane/EtOAc (90:10)); Mp = 104-106 °C; **^{1}H NMR** (400 MHz, Acetone) δ 10.62 (s, 1H), 7.79 (d, *J* = 7.4 Hz, 2H), 7.39 (t, *J* = 7.7 Hz, 2H), 7.28 (d, *J* = 7.4 Hz, 1H), 6.63 (d, *J* = 2.8 Hz, 1H), 4.28 (q, *J* = 7.1 Hz, 2H), 3.61 (sept, *J* = 6.9 Hz, 1H), 1.32 (t, *J* = 7.1 Hz, 3H), 1.25 (d, *J* = 6.9 Hz, 6H); **^{13}C NMR** (100 MHz, Acetone) δ 160.7, 140.9, 135.4, 131.9, 128.7, 127.2, 124.9, 118.7, 105.7, 59.2, 25.4, 23.2, 13.9; **HRMS** (ESI) Calcd for $C_{16}H_{20}NO_2$ [M + H]$^+$ 258.1494, found 258.1498.

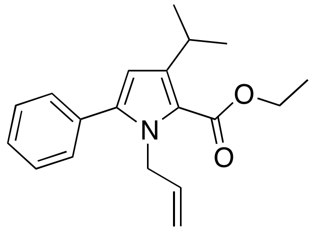

Ethyl 1-allyl-3-isopropyl-5-phenyl-1*H*-pyrrole-2-carboxylate (3za): Yellow oil (57% yield, eluent = hexane/EtOAc (97:3)); **^{1}H NMR** (400 MHz, $CDCl_3$): δ 7.48–7.32 (m, 5H), 6.17 (s, 1H), 6.04–5.84 (m, 1H), 5.06 (dd, *J* = 10.4, 1.3 Hz, 1H), 4.92–4.87 (m, 2H), 4.76 (dd, *J* = 17.2, 1.3 Hz, 1H), 4.31 (q, *J* = 7.1 Hz, 2H), 3.58 (sept, *J* = 6.8 Hz, 1H), 1.36 (t, *J* = 7.1 Hz, 3H), 1.24 (d, *J* = 6.9 Hz, 6H); **^{13}C NMR** (100 MHz, $CDCl_3$): δ 162.2, 142.1, 140.7, 136.4, 132.9, 129.7, 128.5, 128.3, 119.0, 115.2, 108.2, 59.9, 48.7, 26.6, 24.2, 14.6; **HRMS** (ESI) Calcd for $C_{19}H_{24}NO_2$ [M + H]$^+$ 298.1807, found 298.1805.

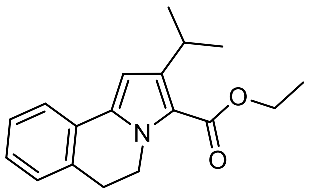

Ethyl 2-isopropyl-5,6-dihydropyrrolo[2,1-*a*]isoquinoline-3-carboxylate (3aaa): Yellow oil (73%, eluent = hexane/EtOAc (95:5)); ^{1}**H NMR** (400 MHz, $CDCl_3$) δ 7.55 (d, *J* = 7.6 Hz, 1H), 7.28–7.19 (m, 1H), 7.20–7.09 (m, 2H), 6.50 (s, 1H), 4.63–4.52 (m, 2H), 4.35 (d, *J* = 7.1 Hz, 2H), 3.56 (sept, *J* = 6.8 Hz, 1H), 3.04 (t, *J* = 6.8 Hz, 2H), 1.38 (t, *J* = 7.1 Hz, 3H), 1.27 (s, 3H), 1.25 (s, 3H); ^{13}**C NMR** (100 MHz, $CDCl_3$) δ 162.4, 142.5, 135.1, 132.1, 128.6, 127.9, 127.4, 127.2, 123.8, 118.5, 103.0, 59.9, 42.8, 29.3, 26.5, 24.2, 14.6. **HRMS** (ESI) Calcd for $C_{18}H_{22}NO_2$ $[M + H]^+$ 284.1651, found 284,1652.

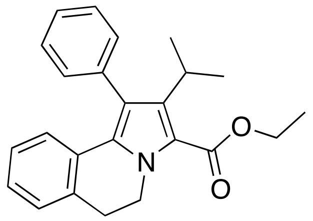

Ethyl 2-isopropyl-1-phenyl-5,6-dihydropyrrolo[2,1-*a*]isoquinoline-3-carboxylate (3aba): White solid (48%, eluent = hexane/EtOAc (98:2)); Mp = 135-136 °C; ^{1}**H NMR** (400 MHz, $CDCl_3$): δ 7.42–7.36 (m, 3H), 7.33–7.27 (m, 2H), 7.15 (d, *J* = 7.5 Hz, 1H), 7.04 (t, *J* = 7.4 Hz, 1H), 6.85 (t, *J* = 7.7 Hz, 1H), 6.68 (d, *J* = 7.9 Hz, 1H), 4.54 (t, *J* = 6.6 Hz, 2H), 4.37 (q, *J* = 7.1 Hz, 2H), 3.33 (sept, *J* = 7.1 Hz, 1H), 3.05 (t, *J* = 6.6 Hz, 2H), 1.41 (t, *J* = 7.1 Hz, 3H), 1.16 (d, *J* = 7.2 Hz, 6H); ^{13}**C NMR** (100 MHz, $CDCl_3$): δ 162.7, 138.2, 137.5, 133.5, 131.6, 131.1, 128.9, 128.7, 127.8, 127.3, 126.8, 126.7, 125.3, 123.1, 118.7, 60.3, 43.0, 29.9, 26.5, 22.8, 14.6; **HRMS** (ESI) Calcd for $C_{24}H_{26}NO_2$ $[M + H]^+$ 360.1964, found 360.1960.

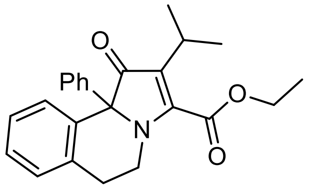

Ethyl 2-isopropyl-1-oxo-10*b*-phenyl-1,5,6,10*b*-tetrahydropyrrolo[2,1-*a*]isoquinoline-3-carboxylate (3aba'): Orange oil (50%, eluent = hexane/EtOAc (96:4)); ^{1}**H NMR** (400 MHz, $CDCl_3$) δ 7.88 (dd, *J* = 7.6, 1.6 Hz, 1H), 7.38–7.25 (m, 5H), 7.20-7.18 (m, 1H), 7.12–6.91 (m, 2H), 4.47 (q, *J* = 6.9 Hz, 2H), 3.73-3.67 (m, 1H), 3.58–3.43 (m, 1H), 3.02-3.98 (m, 1H), 2.89–2.66 (m, 2H), 1.45 (t, *J* = 7.1 Hz, 3H), 1.22 (d, *J* = 10.8, 3H), 1.21 (d, *J* = 10.8, 3H); ^{13}**C**

NMR (100 MHz, $CDCl_3$) δ 201.5, 163.1, 162.0, 140.9, 134.3, 133.1, 128.5, 128.5, 128.4, 127.8, 127.7, 127.4, 126.6, 120.1, 73.3, 62.4, 41.6, 29.5, 24.2, 21.21, 21.15, 14.2; **HRMS** (ESI) Calcd for $C_{24}H_{26}NO_3$ $[M + H]^+$ 376.1913, found 376.1908.

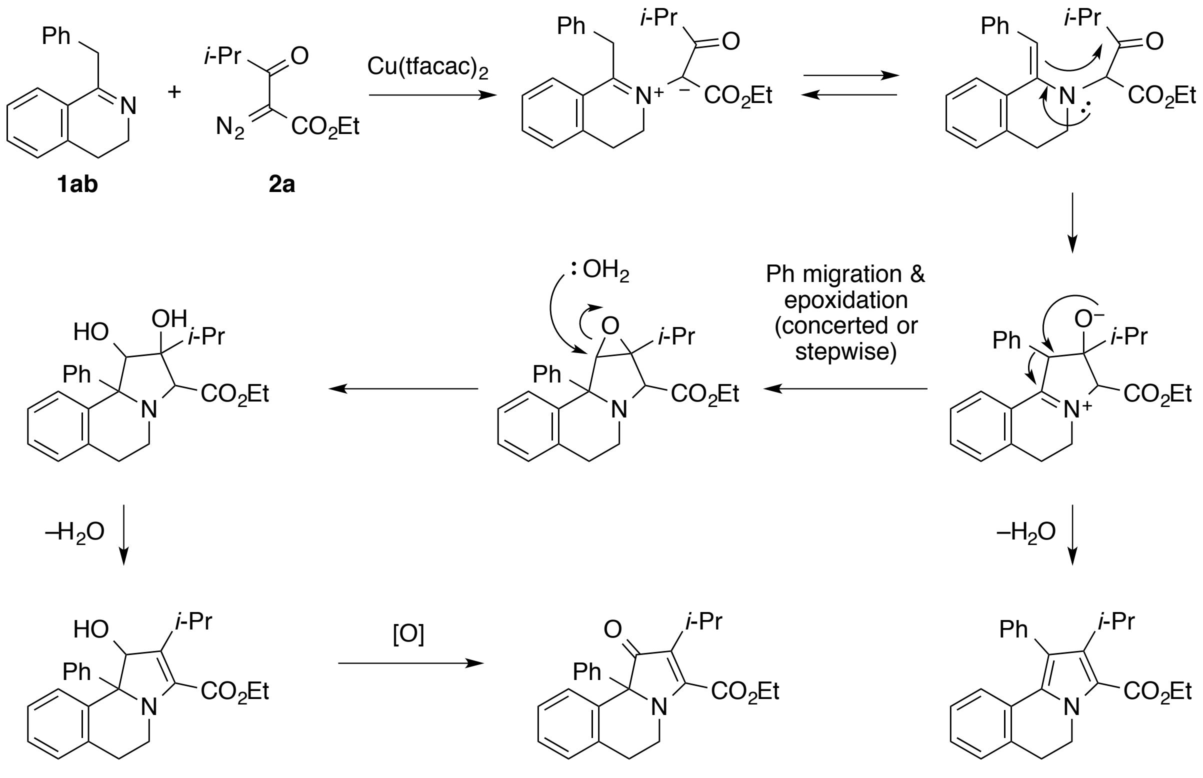

Scheme S1. Outline of possible pathways for the formation of pyrrole **3aba** and 5,6-dihydropyrrolo[2,1-*a*]isoquinoline **3aba'**

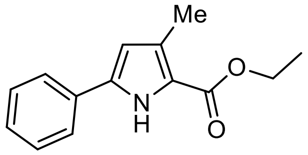

Ethyl 3-methyl-5-phenyl-1*H*-pyrrole-2-carboxylate (3acb): White solid (35%, eluent = hexane/EtOAc (96:4)); Mp = 114-116 °C; ^{1}**H NMR** (400 MHz, $CDCl_3$) δ 9.15 (s, 1H), 7.59 – 7.54 (m, 2H), 7.42 (t, J = 7.7 Hz, 2H), 7.34 – 7.27 (m, 1H), 6.41 (d, J = 3.0 Hz, 1H), 4.37 (q, J = 7.1 Hz, 2H), 2.41 (s, 3H), 1.41 (t, J = 7.1 Hz, 3H); ^{13}**C NMR** (100 MHz, $CDCl_3$) δ 161.8, 135.1, 131.4, 129.4, 129.0, 127.6, 124.6, 119.9, 110.2, 60.1, 14.6, 13.0; **HRMS** (ESI) Calcd for $C_{14}H_{16}NO_2$ $[M + H]^+$ 230.1181, found 230.1183.

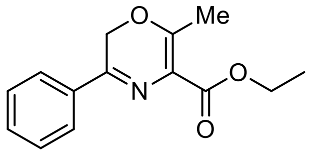

Ethyl 6-methyl-3-phenyl-2*H*-1,4-oxazine-5-carboxylate (4):[8] Yellow oil (22%, eluent = hexane/EtOAc (96:4)); ^{1}**H NMR** (400 MHz, $CDCl_3$) δ 7.97– 7.76 (m, 2H), 7.51–7.39 (m, 3H), 4.82 (s, 2H), 4.37 (q, *J* = 7.1 Hz, 2H), 2.41 (s, 3H), 1.42 (t, *J* = 7.1 Hz, 3H); ^{13}**C NMR** (100 MHz, $CDCl_3$) δ 165.8, 157.6, 148.3, 135.0, 130.6, 128.7, 126.6, 120.7, 62.0, 60.6, 17.9, 14.4; **HRMS** (ESI) Calcd for $C_{14}H_{16}NO_3$ $[M + H]^+$ 246.1130, found 246.1136.

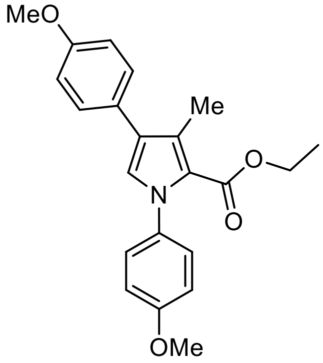

Ethyl 1,4-bis(4-methoxyphenyl)-3-methyl-1*H*-pyrrole-2-carboxylate (3adb): Yellow oil (32%, eluent = hexane/EtOAc (98:2)); ^{1}**H NMR** (400 MHz, $CDCl_3$) δ 7.34 (d, *J* = 8.5 Hz, 2H), 7.25 (d, *J* = 8.7 Hz, 2H), 6.98-6.94 (m, 4H), 6.89 (s, 1H), 4.18 (q, *J* = 7.1 Hz, 2H), 3.868 (s, 3H), 3.866 (s, 3H) 2.47 (s, 3H), 1.17 (t, *J* = 7.1 Hz, 3H); ^{13}**C NMR** (100 MHz, $CDCl_3$) δ 161.5, 158.8, 158.3, 134.5, 129.7, 127.5, 127.32, 127.28, 126.8, 125.8, 121.6, 113.9, 113.6, 59.7, 55.5, 55.3, 14.2, 12.2; **HRMS** (ESI) Calcd for $C_{22}H_{24}NO_4$ $[M + H]^+$ 366.1705, found 366.1701.

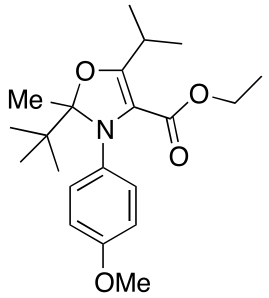

Ethyl 2-(*t*-butyl)-5-isopropyl-3-(4-methoxyphenyl)-2-methyl-2,3-dihydrooxazole-4-carboxylate (5a): Orange oil (95% yield, eluent = hexane/EtOAc (95:5)); ^{1}**H NMR** (400 MHz, $CDCl_3$): δ 7.07 (s, 2H), 6.80 (d, *J* = 9.1 Hz, 2H), 4.09 (dq, *J* = 10.8, 7.1 Hz, 1H), 3.94 (dq, *J* = 10.8, 7.1 Hz, 1H), 3.79 (s, 3H), 3.64 (sept, *J* = 7.0 Hz, 1H), 1.28 (d, *J* = 7.0 Hz, 3H),

1.24 (d, J = 6.9 Hz, 3H), 1.11 (s, 9H), 1.10 (s, 3H), 1.01 (t, J = 7.1 Hz, 3H); ^{13}**C NMR** (100 MHz, $CDCl_3$): δ 164.0, 163.0, 156.7, 140.7, 116.3, 113.6, 108.7, 59.3, 55.3, 41.4, 26.0, 25.0, 20.1, 19.8, 19.7, 14.1; **HRMS** (ESI) Calcd for $C_{21}H_{32}NO_4$ $[M + H]^+$ 362.2331, found 362.2327.

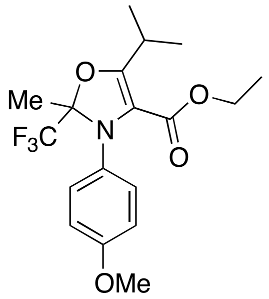

Ethyl 5-isopropyl-3-(4-methoxyphenyl)-2-methyl-2-(trifluoromethyl)-2,3-dihydrooxazole-4-carboxylate (5b): Yellow oil (70% yield, eluent = hexane/EtOAc (95:5)); ^{1}H NMR (400 MHz, $CDCl_3$): δ 7.13 (s, 1H), 6.95 (s, 1H), 6.85 (s, 2H), 4.14 (dq, J = 10.8, 7.1 Hz, 1H), 3.96 (dq, J = 10.8, 7.1 Hz, 1H), 3.81 (s, 3H), 3.64 (sept, J = 7.0 Hz, 1H), 1.31 (s, 3H), 1.27 (d, J = 7.0 Hz, 3H), 1.22 (d, J = 6.9 Hz, 3H), 1.02 (t, J = 7.1 Hz, 3H); ^{13}**C NMR** (100 MHz, $CDCl_3$): δ 163.1, 162.1, 157.9, 137.2, 129.4, 125.9, 123.3 (q, $J_{C\text{-}F}$ = 287.4 Hz), 115.9, 114.3, 114.0, 98.6 (q, $J_{C\text{-}F}$ = 31.4 Hz), 60.2, 55.6, 26.0, 20.3, 19.2, 17.5, 14.1; **HRMS** (ESI) Calcd for $C_{18}H_{23}F_3NO_4$ $[M + H]^+$ 374.1579, found 374.1578.

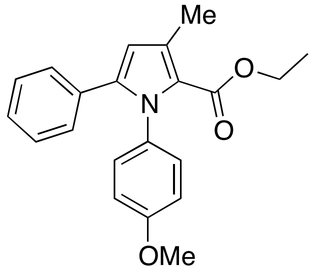

Ethyl 1-(4-methoxyphenyl)-3-methyl-5-phenyl-1*H*-pyrrole-2-carboxylate (3ab): Yellow solid (81% yield, eluent = hexane/EtOAc (95:5)); Mp = 104–105 °C; ^{1}**H NMR** (400 MHz, $CDCl_3$): δ 7.17–7.15 (m, 3H), 7.12–7.01 (m, 4H), 6.81 (d, J = 8.9 Hz, 2H), 6.26 (s, 1H), 4.12 (q, J = 7.1 Hz, 2H), 3.79 (s, 3H), 2.44 (s, 3H), 1.16 (t, J = 7.1 Hz, 3H); ^{13}**C NMR** (100 MHz, $CDCl_3$): δ 161.7, 159.0, 140.1, 133.1, 132.4, 130.3, 129.9, 129.1, 128.2, 127.4, 122.6, 113.6, 112.9, 59.7, 55.6, 14.5, 14.4; **HRMS** (ESI) Calcd for $C_{21}H_{22}NO_3$ $[M + H]^+$ 336.1600, found 336.1599.

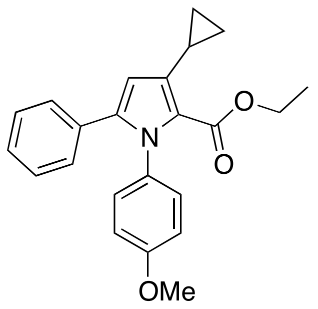

Ethyl 3-cyclopropyl-1-(4-methoxyphenyl)-5-phenyl-1*H*-pyrrole-2-carboxylate (3ac): Yellow solid (80% yield, eluent = hexane/EtOAc (95:5)); Mp = 108–109 °C; **^{1}H NMR** (400 MHz, $CDCl_3$): δ 7.19–7.10 (m, 3H), 7.10–7.00 (m, 4H), 6.80 (d, *J* = 8.8 Hz, 2H), 5.95 (s, 1H), 4.13 (q, *J* = 7.1 Hz, 2H), 3.78 (s, 3H), 2.60 (tt, *J* = 8.5, 5.2 Hz, 1H), 1.13 (t, *J* = 7.1 Hz, 3H), 1.05 – 0.90 (m, 2H), 0.75 – 0.59 (m, 2H); **^{13}C NMR** (100 MHz, $CDCl_3$): δ 161.9, 159.0, 140.5, 137.4, 133.1, 132.4, 129.9, 129.1, 128.2, 127.4, 123.1, 113.6, 106.7, 59.8, 55.5, 14.4, 9.3, 8.7; **HRMS** (ESI) Calcd for $C_{23}H_{24}NO_3$ $[M + H]^+$ 362.1756, found 362.1754.

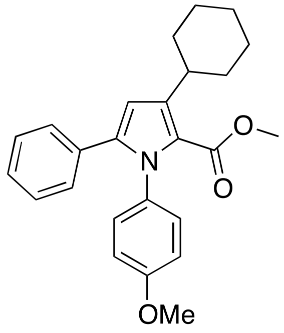

Methyl 3-cyclohexyl-1-(4-methoxyphenyl)-5-phenyl-1*H*-pyrrole-2-carboxylate (3ad): Yellow solid (88% yield, eluent = hexane/EtOAc (95:5)); Mp = 128–130 °C; **^{1}H NMR** (400 MHz, $CDCl_3$): δ 7.18–7.13 (m, 3H), 7.11–7.06 (m, 4H), 6.81 (d, *J* = 8.9 Hz, 2H), 6.33 (s, 1H), 3.79 (s, 3H), 3.64 (s, 3H), 3.23–3.17 (m, 1H), 1.99–1.97 (m, 2H), 1.89–1.71 (m, 3H), 1.50–1.37 (m, 4H), 1.32–1.25 (m, 1H); **^{13}C NMR** (100 MHz, $CDCl_3$): δ 162.2, 158.9, 141.1, 140.4, 133.1, 132.6, 129.8, 129.1, 128.1, 127.3, 121.2, 113.6, 109.1, 55.5, 51.0, 36.7, 34.7, 27.2, 26.6; **HRMS** (ESI) Calcd for $C_{25}H_{28}NO_3$ $[M + H]^+$ 390.2069, found 390.2066.

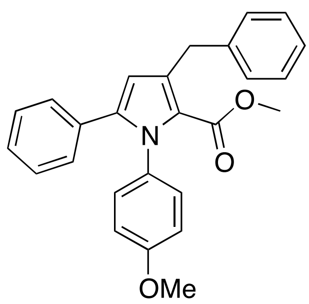

Methyl 3-benzyl-1-(4-methoxyphenyl)-5-phenyl-1*H*-pyrrole-2-carboxylate (3ae): Yellow solid (94% yield, eluent = hexane/EtOAc (95:5)); Mp = 139–141 °C; **^{1}H NMR** (400 MHz, $CDCl_3$): δ 7.35–7.27 (m, 4H), 7.24–7.16 (m, 1H), 7.16–7.01 (m, 7H), 6.82 (d, *J* = 8.8 Hz, 2H), 6.14 (s, 1H), 4.22 (s, 2H), 3.79 (s, 3H), 3.65 (s, 3H); **^{13}C NMR** (100 MHz, $CDCl_3$): δ 162.0, 159.1, 141.5, 140.5, 133.5, 132.8, 132.2, 129.8, 129.2, 129.1, 128.6, 128.2, 127.4, 126.1, 121.9, 113.7, 112.3, 55.5, 51.0, 34.4; **HRMS** (ESI) Calcd for $C_{26}H_{24}NO_3$ $[M + H]^+$ 398.1756, found 398.1757.

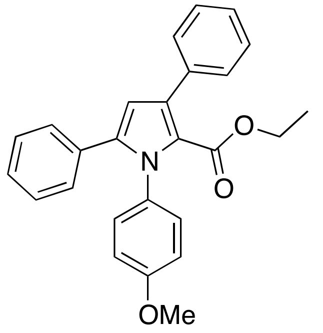

Ethyl 1-(4-methoxyphenyl)-3,5-diphenyl-1*H*-pyrrole-2-carboxylate (3af): Yellow solid (77% yield, eluent = hexane/EtOAc (95:5)); Mp = 125–127 °C; ^{1}H NMR (400 MHz, $CDCl_3$): δ 7.52 (d, *J* = 7.2 Hz, 2H), 7.38 (t, *J* = 7.4 Hz, 2H), 7.30 (t, *J* = 7.3 Hz, 1H), 7.20 – 7.08 (m, 7H), 6.84 (d, *J* = 8.8 Hz, 2H), 6.46 (s, 1H), 3.97 (q, *J* = 7.1 Hz, 2H), 3.79 (s, 3H), 0.90 (t, *J* = 7.1 Hz, 3H); **^{13}C NMR** (100 MHz, $CDCl_3$): δ 161.4 159.0, 139.6, 136.2, 133.2, 132.3, 132.0, 129.6, 129.5, 129.0, 128.1, 127.7, 127.3, 126.8, 121.9, 113.6, 111.9, 60.0, 55.4, 13.7; **HRMS** (ESI) Calcd for $C_{26}H_{24}NO_3$ $[M + H]^+$ 398.1756, found 398.1752.

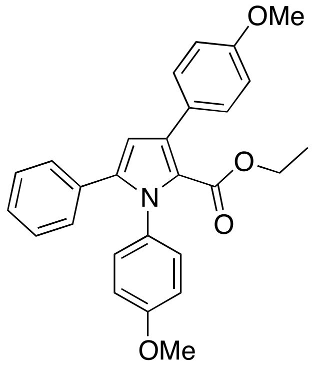

Ethyl 1,3-bis(4-methoxyphenyl)-5-phenyl-1*H*-pyrrole-2-carboxylate (3ag): Yellow solid (77% yield, eluent = hexane/EtOAc (95:5)); Mp = 107–108 °C; **^{1}H NMR** (400 MHz, $CDCl_3$): δ 7.47 (d, *J* = 8.7 Hz, 2H), 7.23–7.06 (m, 7H), 6.93 (d, *J* = 8.7 Hz, 2H), 6.84 (d, *J* = 8.8 Hz, 2H), 6.43 (s, 1H), 3.98 (q, *J* = 7.1 Hz, 2H), 3.85 (s, 3H), 3.80 (s, 3H), 0.93 (t, *J* = 7.1 Hz, 3H);

^{13}C NMR (100 MHz, $CDCl_3$): δ 161.6, 159.2, 158.9, 139.8, 133.3, 132.7, 132.3, 130.8, 129.8, 129.2, 128.8, 128.3, 127.5, 121.9, 113.8, 113.4, 112.1, 60.1, 55.6, 55.5, 14.0; **HRMS** (ESI) Calcd for $C_{27}H_{26}NO_4$ $[M + H]^+$ 428.1862, found 428.1857.

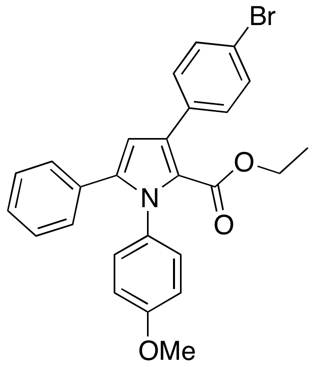

Ethyl 3-(4-bromophenyl)-1-(4-methoxyphenyl)-5-phenyl-1*H*-pyrrole-2-carboxylate (3ah): Orange solid (86% yield, eluent = hexane/EtOAc (95:5)); Mp = 109–110 °C; **^{1}H NMR** (400 MHz, $CDCl_3$): δ 7.50 (d, J = 8.4 Hz, 2H), 7.41 (d, J = 8.5 Hz, 2H), 7.23–7.10 (m, 7H), 6.84 (d, J = 8.9 Hz, 2H), 6.42 (s, 1H), 3.98 (q, J = 7.1 Hz, 2H), 3.80 (s, 3H), 0.93 (t, J = 7.1 Hz, 3H); **^{13}C NMR** (100 MHz, $CDCl_3$): δ 161.3, 159.3, 140.0, 135.4, 132.4, 132.2, 132.0, 131.4, 131.0, 129.7, 129.2, 128.3, 127.7, 122.0, 121.1, 113.8, 111.9, 60.3, 55.6, 13.9; **HRMS** (ESI) Calcd for $C_{26}H_{23}BrNO_3$ $[M + H]^+$ 470.0861, found 476.0860.

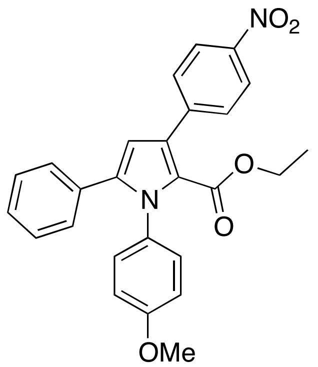

Ethyl 1-(4-methoxyphenyl)-3-(4-nitrophenyl)-5-phenyl-1*H*-pyrrole-2-carboxylate (3ai): Orange solid (80% yield, eluent = hexane/EtOAc (95:5)); Mp = 122–123 °C; **^{1}H NMR** (400 MHz, $CDCl_3$): δ 8.25 (d, J = 8.8 Hz, 2H), 7.70 (d, J = 8.7 Hz, 2H), 7.24–7.19 (m, 3H), 7.17–7.10 (m, 4H), 6.86 (d, J = 8.8 Hz, 2H), 6.48 (s, 1H), 3.99 (q, J = 7.1 Hz, 2H), 3.82 (s, 3H), 0.92 (t, J = 7.1 Hz, 3H); **^{13}C NMR** (100 MHz, $CDCl_3$): δ 161.1, 159.5, 146.9, 143.50, 140.4, 132.1, 131.7, 130.9, 130.4, 129.7, 129.2, 128.4, 127.9, 123.2, 122.4, 113.9, 111.8, 60.5, 55.6, 13.9; **HRMS** (ESI) Calcd for $C_{26}H_{23}N_2O_5$ $[M + H]^+$ 443.1607, found 443.1611.

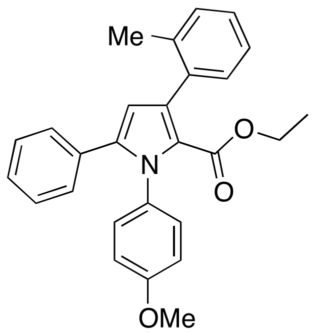

Ethyl 1-(4-methoxyphenyl)-5-phenyl-3-(*o*-tolyl)-1*H*-pyrrole-2-carboxylate (3aj): Yellow solid (67% yield, eluent = hexane/EtOAc (95:5)); Mp = 114–116 °C; **^{1}H NMR** (400 MHz, $CDCl_3$): δ 7.33–7.12 (m, 11H), 6.86 (d, *J* = 8.8 Hz, 2H), 6.35 (s, 1H), 3.87 (q, *J* = 7.1 Hz, 2H), 3.81 (s, 3H), 2.28 (s, 3H), 0.76 (t, *J* = 7.1 Hz, 3H); **^{13}C NMR** (100 MHz, $CDCl_3$): δ 161.3, 159.2, 140.0, 137.1, 136.9, 133.4, 132.6, 132.2, 130.2, 129.9, 129.5, 129.1, 128.3, 127.5, 127.2, 125.2, 122.6, 113.8, 112.3, 59.8, 55.0, 20.6, 13.6; **HRMS** (ESI) Calcd for $C_{27}H_{26}NO_3$ $[M + H]^+$ 412.1913, found 412.1908.

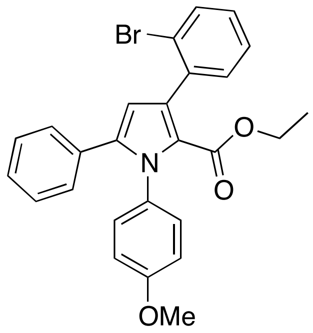

Ethyl 3-(2-bromophenyl)-1-(4-methoxyphenyl)-5-phenyl-1*H*-pyrrole-2-carboxylate (3ak): Orange solid (60% yield, eluent = hexane/EtOAc (95:5)); Mp = 117–119 °C; **^{1}H NMR** (400 MHz, $CDCl_3$): δ 7.63 (d, *J* = 8.0 Hz, 1H), 7.41 (dd, *J* = 7.6, 1.6 Hz, 1H), 7.32 (t, *J* = 7.5 Hz, 1H), 7.22–7.12 (m, 8H), 6.86 (d, *J* = 8.8 Hz, 2H), 6.40 (s, 1H), 3.91 (q, *J* = 7.1 Hz, 2H), 3.80 (s, 3H), 0.80 (t, *J* = 7.1 Hz, 3H); **^{13}C NMR** (100 MHz, $CDCl_3$): δ 160.9, 159.3, 139.8, 138.5, 132.4, 132.4, 132.3, 132.1, 131.6, 129.9, 129.1, 128.5, 128.3, 127.5, 126.9, 124.7, 122.8, 113.8, 112.2, 60.0, 55.6, 13.6; **HRMS** (ESI) Calcd for $C_{26}H_{23}BrNO_3$ $[M + H]^+$ 476.0861, found 476.0864.

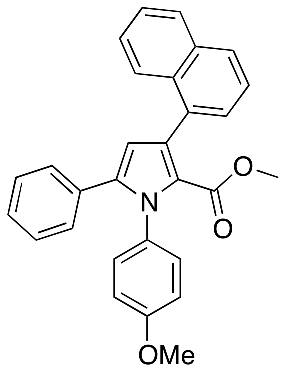

Methyl 1-(4-methoxyphenyl)-3-(naphthalen-1-yl)-5-phenyl-1*H*-pyrrole-2-carboxylate (3al): Orange solid (74% yield, eluent = hexane/EtOAc (90:10)); Mp = 125–127 °C; **^{1}H NMR** (400 MHz, $CDCl_3$): δ 8.03–7.94 (m, 1H), 7.91–7.85 (m, 1H), 7.83 (dd, *J* = 5.9, 3.6 Hz, 1H), 7.55–7.47 (m, 2H), 7.44 (tt, *J* = 12.6, 3.4 Hz, 2H), 7.28–7.16 (m, 7H), 6.88 (d, *J* = 9.0 Hz, 2H), 6.52 (s, 1H), 3.80 (s, 3H), 3.15 (s, 3H); **^{13}C NMR** (100 MHz, $CDCl_3$): δ 161.8, 159.3, 140.2, 135.0, 133.7, 132.7, 132.4, 132.1, 131.6, 129.9, 129.1, 128.3, 128.3, 127.6, 127.5, 127.4, 126.5, 125.9, 125.7, 125.3, 123.2, 113.9, 113.5, 55.5, 50.9; **HRMS** (ESI) Calcd for $C_{29}H_{24}NO_3$ $[M + H]^+$ 434.1756, found 434.1759.

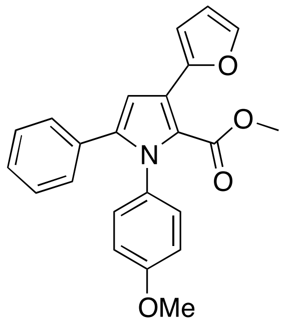

Methyl 3-(furan-2-yl)-1-(4-methoxyphenyl)-5-phenyl-1*H*-pyrrole-2-carboxylate (3am): Orange solid (70% yield, eluent = hexane/EtOAc (95:5)); Mp = 140–141 °C; **^{1}H NMR** (400 MHz, $CDCl_3$): δ 7.47 (d, *J* = 1.2 Hz, 1H), 7.22–7.16 (m, 3H), 7.15–7.04 (m, 4H), 6.91 (d, *J* = 3.3 Hz, 1H), 6.83 (d, *J* = 8.9 Hz, 2H), 6.76 (s, 1H), 6.49 (dd, *J* = 3.3, 1.8 Hz, 1H), 3.80 (s, 3H), 3.64 (s, 3H); **^{13}C NMR** (100 MHz, $CDCl_3$): δ 161.6, 159.2, 149.2, 141.7, 140.4, 132.4, 131.9, 129.6, 129.2, 128.3, 127.7, 122.9, 120.7, 113.8, 111.6, 110.0, 109.0, 55.6, 51.3; **HRMS** (ESI) Calcd for $C_{23}H_{20}NO_4$ $[M + H]^+$ 374.1392, found 374.1395.

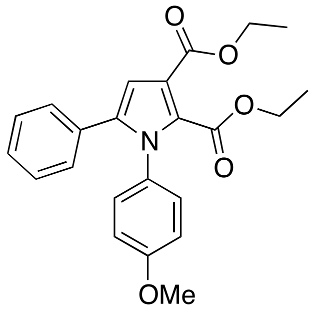

Diethyl 1-(4-methoxyphenyl)-5-phenyl-1*H*-pyrrole-2,3-dicarboxylate (3an): Yellow oil (79% yield, eluent = hexane/EtOAc (95:5)); **^{1}H NMR** (400 MHz, $CDCl_3$): δ 7.23–7.15 (m, 3H), 7.14–7.02 (m, 4H), 6.83 (d, *J* = 8.9 Hz, 2H), 6.75 (s, 1H), 4.33 (q, *J* = 7.1 Hz, 2H), 4.16 (q, *J* = 7.1 Hz, 2H), 3.80 (s, 3H), 1.35 (t, *J* = 7.1 Hz, 3H), 1.13 (d, *J* = 7.1 Hz, 3H); **^{13}C NMR** (100 MHz, $CDCl_3$): δ 164.5, 161.9, 159.7, 137.3, 131.5, 130.8, 129.5, 129.3, 129.0 128.4, 127.7, 118.5, 114.1, 110.5, 61.6, 60.7, 55.6, 14.5, 14.1; **HRMS** (ESI) Calcd for $C_{23}H_{24}NO_5$ $[M + H]^+$ 394.1654, found 394.1658.

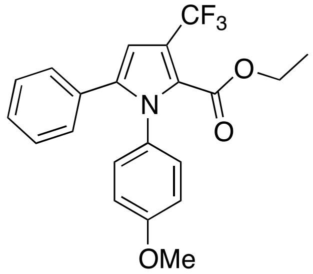

Ethyl 1-(4-methoxyphenyl)-5-phenyl-3-(trifluoromethyl)-1*H*-pyrrole-2-carboxylate (3ao): Yellow solid (41% yield, eluent = hexane/EtOAc (95:5)); Mp = 111–113 °C; **^{1}H NMR** (400 MHz, $CDCl_3$): δ 7.21–7.19 (m, 3H), 7.15–7.02 (m, 4H), 6.84 (d, *J* = 8.9 Hz, 2H), 6.65 (s, 1H), 4.18 (q, *J* = 7.1 Hz, 2H), 3.80 (s, 3H), 1.21 (t, *J* = 7.1 Hz, 3H); **^{13}C NMR** (100 MHz, $CDCl_3$): 159.69, 159.66, 139.2, 131.2, 131.1, 129.6, 129.3, 128.4, 128.1, 126.2 (q, $J_{C\text{-}F}$ = 3.3 Hz) 123.2 (q, $J_{C\text{-}F}$ = 265.0 Hz), 120.5 (q, $J_{C\text{-}F}$ = 37.2 Hz), 114.0, 109.3 (q, $J_{C\text{-}F}$ = 4.2 Hz), 61.2, 55.6, 13.9; **HRMS** (ESI) Calcd for $C_{21}H_{19}F_3NO_3$ $[M + H]^+$ 390.1317, found 390.1297.

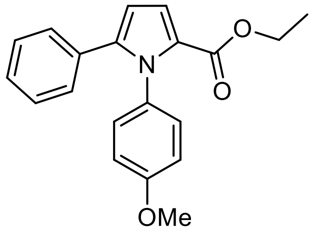

Ethyl 1-(4-methoxyphenyl)-5-phenyl-1*H*-pyrrole-2-carboxylate (3ap): White solid (41% yield, eluent = hexane/EtOAc (95:5)); Mp = 132-133 °C; **^{1}H NMR** (400 MHz, $CDCl_3$) δ

7.23–7.08 (m, 8H), 6.84 (d, *J* = 8.8 Hz, 2H), 6.40 (d, *J* = 4.0 Hz, 1H), 4.16 (q, *J* = 7.1 Hz, 2H), 3.80 (s, 3H), 1.22 (t, *J* = 7.1 Hz, 3H); **^{13}C NMR** (100 MHz, $CDCl_3$) δ 160.8, 159.2, 141.6, 132.4, 132.2, 129.8, 129.1, 128.3, 127.5, 125.4, 118.4, 113.8, 109.9, 60.0, 55.6, 14.6; **HRMS** (ESI) Calcd for $C_{20}H_{20}NO_3$ $[M + H]^+$ 322.1443, found 322.1444.

Cu/Yb-Catalyzed Condensation of Imine with α-Diazo-β-diketone

Table S1. Screening of Cocatalysts

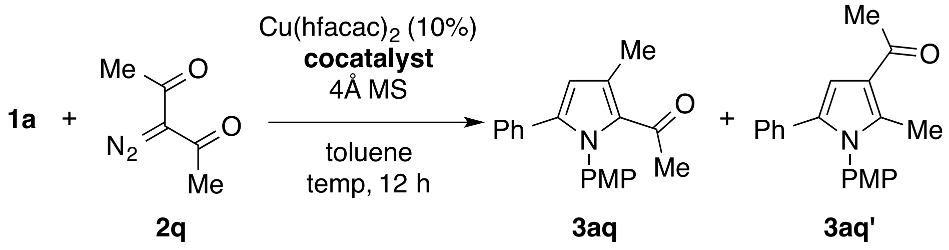

entry	cocatalyst	temp (°C)	yield (%)[a]	
			**3aq**	**3aq'**
1	none	110	11[b]	12[b]
2	$Yb(OTf)_3$ (10%)	110	36	5
3	$Gd(OTf)_3$ (10%)	110	23	trace
4	$La(OTf)_3$ (10%)	110	22	trace
5	$In(OTf)_3$ (10%)	110	23	trace
**6**	**$Yb(OTf)_3$ (10%)**	**80**	**57[b]**	**trace**
7	$Yb(OTf)_3$ (20%)	80	21	trace

[a] Determined by GC using *n*-tridecane as an internal standard. [b] Isolated yield.

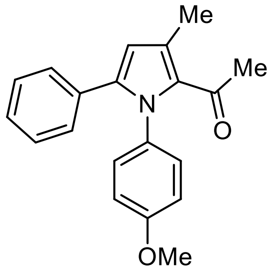

1-(1-(4-Methoxyphenyl)-3-methyl-5-phenyl-1*H*-pyrrol-2-yl)ethanone (3aq): Brown solid (57% yield, eluent = hexane/EtOAc (95:5)); Mp = 118–120 °C; **^{1}H NMR** (400 MHz, $CDCl_3$): δ 7.23–7.14 (m, 3H), 7.10–7.03 (m, 4H), 6.83 (d, *J* = 8.8 Hz, 2H), 6.26 (s, 1H), 3.80 (s, 3H), 2.46 (s, 3H), 2.15 (s, 3H); **^{13}C NMR** (100 MHz, $CDCl_3$): δ 189.1, 159.2, 140.4, 133.1, 132.5, 132.2, 130.0 129.8, 129.2, 128.2, 127.6, 114.0, 113.6, 55.6, 30.7, 15.3; **HRMS** (ESI) Calcd for $C_{20}H_{20}NO_2$ $[M + H]^+$ 306.1494, found 306.1490.

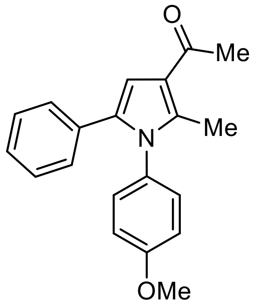

1-(1-(4-Methoxyphenyl)-2-methyl-5-phenyl-1*H*-pyrrol-3-yl)ethanone (3aq'): Brown solid (12% yield obtained in the absence of Yb(OTf)$_3$, eluent = hexane/EtOAc (98:2)); Mp = 98–100 °C; **^{1}H NMR** (400 MHz, $CDCl_3$): δ 7.29–7.26 (m, 3H), 7.21–7.18 (m, 2H), 6.95 (d, *J* = 8.9 Hz, 2H), 6.73 (d, *J* = 8.9 Hz, 2H), 6.64 (s, 1H), 3.75 (s, 3H), 2.36 (s, 3H), 1.93 (s, 3H); **^{13}C NMR** (100 MHz, $CDCl_3$): δ 196.9, 158.7, 138.5, 133.0, 132.6, 131.5, 128.5, 128.4, 127.6, 124.2, 122.5, 121.4, 114.2, 55.6, 30.9, 12.8; **HRMS** (ESI) Calcd for $C_{20}H_{20}NO_2$ [M + H]$^+$ 306.1494, found 306.1490.

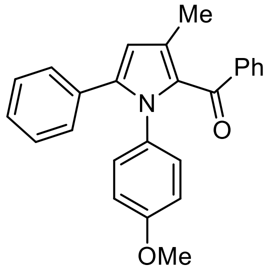

(1-(4-Methoxyphenyl)-3-methyl-5-phenyl-1*H*-pyrrol-2-yl)(phenyl)methanone (3ar): Yellow oil (50%, eluent = hexane/EtOAc (95:5)); **^{1}H NMR** (400 MHz, $CDCl_3$): δ 7.76–7.72 (m, 2H), 7.50–7.45 (m, 1H), 7.38 (t, *J* = 7.5 Hz, 2H), 7.20-7.19 (m, 3H), 7.15–7.09 (m, 2H), 7.05 (d, *J* = 8.9 Hz, 2H), 6.73 (d, *J* = 8.9 Hz, 2H), 6.28 (s, 1H), 3.73 (s, 3H), 1.96 (s, 3H); **^{13}C NMR** (100 MHz, $CDCl_3$): δ 188.1, 158.7, 140.6, 140.3, 132.4, 132.2, 132.1, 129.6, 129.4, 129.0, 128.8, 128.4, 128.3, 127.5, 113.9, 113.0, 55.5, 14.0; **HRMS** (ESI) Calcd for $C_{25}H_{22}NO_2$ [M + H]$^+$ 368.1651, found 368.1653.

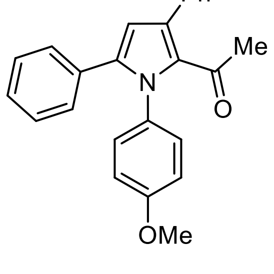

1-(1-(4-Methoxyphenyl)-3,5-diphenyl-1*H*-pyrrol-2-yl)ethanone (3ar'): Yellow solid (13%, eluent = hexane/EtOAc (95:5)); Mp = 126-128 °C; **^{1}H NMR** (400 MHz, $CDCl_3$) δ 7.51 – 7.33 (m, 5H), 7.21 – 7.17 (m, 3H), 7.15 – 7.11 (m, 4H), 6.84 (d, *J* = 8.9 Hz, 2H), 6.42 (s, 1H), 3.81 (s, 3H), 1.98 (s, 3H); **^{13}C NMR** (100 MHz, $CDCl_3$): δ 190.5, 158.9, 140.0, 136.7, 135.7, 134.9, 133.8, 132.5, 131.7, 129.5, 129.0, 128.3, 128.1, 127.5, 127.4, 113.7, 112.3, 55.4, 30.6; **HRMS** (ESI) Calcd for $C_{25}H_{22}NO_2$ $[M + H]^+$ 368.1651, found 368.1653.

Application to Lamellarin Scaffolds

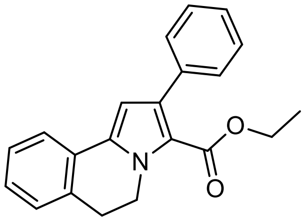

Ethyl 2-phenyl-5,6-dihydropyrrolo[2,1-*a*]isoquinoline-3-carboxylate (3aaf): White solid (62% yield, eluent = hexane/EtOAc (95:5)); Mp = 119-121 °C; ^{1}H NMR (400 MHz, $CDCl_3$) δ 7.57 (d, *J* = 7.5 Hz, 1H), 7.44-7.41 (m, 2H), 7.37-7.34 (m, 2H), 7.32–7.26 (m, 2H), 7.23-7.22 (m, 2H), 6.57 (s, 1H), 4.73–4.41 (m, 2H), 4.14 (q, *J* = 7.1 Hz, 2H), 3.12 (t, *J* = 6.8 Hz, 2H), 1.06 (t, *J* = 7.1 Hz, 3H); ^{13}C NMR (100 MHz, $CDCl_3$) δ 161.9, 136.9, 134.7, 134.4, 132.0, 129.6, 128.1, 127.9, 127.51, 127.45, 127.2, 126.7, 123.6, 118.7, 106.9, 59.9, 42.7, 29.0, 13.8; HRMS (ESI) Calcd for $C_{21}H_{20}NO_2$ $[M + H]^+$ 318.1494, found 318.1495.

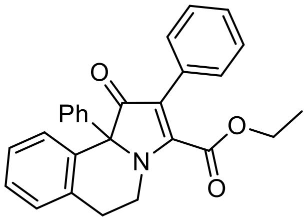

Ethyl 1-oxo-2,10*b*-diphenyl-1,5,6,10*b*-tetrahydropyrrolo[2,1-*a*]isoquinoline-3-carboxylate (6): Yellow solid (63% yield, eluent = hexane/EtOAc (93:7)); Mp = 168-169 °C; ^{1}H NMR (400 MHz, $CDCl_3$) δ 8.05–7.94 (m, 1H), 7.42-7.40 (m, 2H), 7.37–7.27 (m, 7H), 7.25–7.19 (m, 2H), 7.17 –7.11 (m, 2H), 4.37 (q, *J* = 7.2 Hz, 2H), 3.86-3.80 (m, 1H), 3.64-3.57 (m, 1H), 3.08-3.04 (m, 1H), 2.88-2.83 (m, 1H), 1.23 (t, *J* = 7.1 Hz, 3H); ^{13}C NMR (100 MHz, $CDCl_3$) δ 199.2, 162.9, 162.4, 140.3, 134.0, 132.8, 130.2, 128.61, 128.55, 128.5, 128.2, 127.8, 127.7, 127.7, 126.8, 113.8, 74.2, 62.8, 41.6, 29.9, 13.8; HRMS (ESI) Calcd for $C_{27}H_{24}NO_3$ $[M + H]^+$ 410.1756, found 410.1757. Recrystallization from EtOAc/hexane afforded single crystals suitable for X-ray diffraction analysis, which unambiguously confirmed structure of **6** (see below).[9]

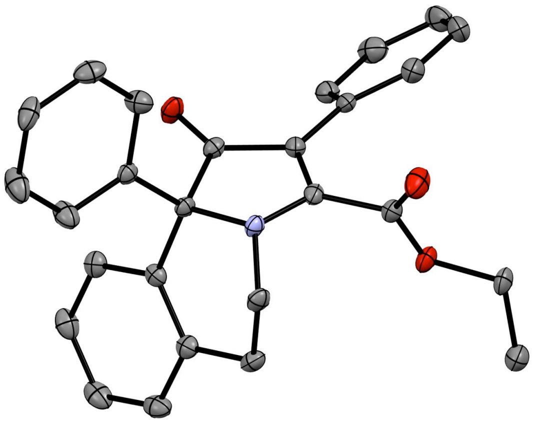

Figure S2. ORTEP diagram of **6**. Thermal ellipsoids drawn at 50% probability.

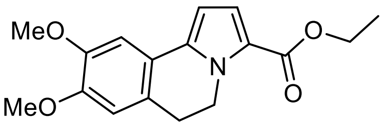

Ethyl 8,9-dimethoxy-5,6-dihydropyrrolo[2,1-*a*]isoquinoline-3-carboxylate (3agp): White solid (68% yield, eluent = hexane/EtOAc (95:5)); Mp = 134-136 °C; ^{1}H NMR (400 MHz, $CDCl_3$) δ 7.12–6.94 (m, 2H), 6.73 (s, 1H), 6.42 (d, *J* = 4.1 Hz, 1H), 4.61 (t, *J* = 6.8 Hz, 2H), 4.29 (q, *J* = 7.1 Hz, 2H), 3.92 (s, 3H), 3.90 (s, 3H), 3.00 (t, *J* = 6.8 Hz, 2H), 1.36 (t, *J* = 7.1 Hz, 3H); ^{13}C NMR (100 MHz, $CDCl_3$) δ 161.6, 148.9, 148.5, 136.5, 124.8, 121.9, 121.3, 118.5, 111.2, 107.1, 103.5, 60.0, 56.3, 56.2, 42.5, 28.8, 14.7; HRMS (ESI) Calcd for $C_{17}H_{20}NO_4$ $[M + H]^+$ 302.1392, found 302.1403.

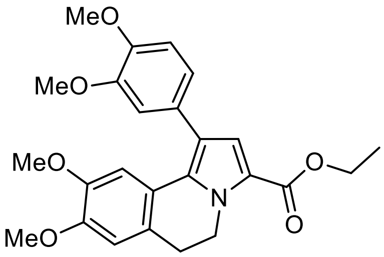

Ethyl 1-(3,4-dimethoxyphenyl)-8,9-dimethoxy-5,6-dihydropyrrolo[2,1-*a*]isoquinoline-3-carboxylate (3ahp):[10] Pale yellow solid (48% yield, eluent = hexane/EtOAc (95:5)); Mp = 148-149 °C; ^{1}H NMR (400 MHz, $CDCl_3$) δ 7.02-6.98 (m, 3H), 6.91-6.89 (m, 2H), 6.73 (s, 1H), 4.62 (t, *J* = 6.6 Hz, 2H), 4.32 (q, *J* = 7.1 Hz, 2H), 3.91 (s, 3H), 3.89 (s, 3H), 3.84 (s, 3H), 3.44 (s, 3H), 3.03 (t, *J* = 6.5 Hz, 2H), 1.37 (t, *J* = 7.1 Hz, 3H); ^{13}C NMR (100 MHz, $CDCl_3$) δ 161.6, 149.0, 148.4, 148.2, 147.5, 131.7, 129.5, 126.1, 121.9, 121.7, 121.3, 120.7, 119.3, 112.9, 111.5, 111.0, 108.9, 60.1, 56.2, 56.1, 56.1, 55.7, 42.7, 29.3, 14.7; HRMS (ESI) Calcd for $C_{25}H_{28}NO_6$ $[M + H]^+$ 438.1917, found 438.1918.

References

(1) (a) Mrsic, N.; Panella, L.; Minnaard, A. J.; Feringa, B. L.; de Vries, J. G. *J. Am. Chem.*

(2) (a) For **1a**, **1h**, Samec, J. S. M.; Ell, A. H.; Bäckvall, J.-E. *Chem. Eur. J.* **2005**, *11*, 2327. (b) For **1b**, **1c**, **1k**, **1p**, Moessner, C.; Bolm, C. *Angew. Chem. Int. Ed.* **2005**, *44*, 7564. (c) For **1d**, Triforiova, A.; Diesen, J. S.; Chapman, C. J.; Andersson, P. G. *Org. Lett*. **2004**, *6*, 3825. (d) For **1e**, **1f**, **1g**, Yoshikai, N.; Matsumoto, A.; Norinder, J.; Nakamura, E. *Angew. Chem. Int. Ed*. **2009**, *48*, 2925. (e) For **1i**, Gautier, F.-M.; Jones, S.; Martin, S. J. *Org. Biomol. Chem.* **2009**, *7*, 229. (f) For **1l**, **1m**, **1n**, **1ae**, Malkov, A. V.; Vrankova, K.; Stoncius, S.; Kocovsky, P. *J. Org. Chem*. **2009**, *74*, 5839. (g) For **1o**, Dong, J.; Lee, P.-S.; Yoshikai, N. *Chem. Lett*. **2013**, *42*, 1140. (h) For **1q**, **1r**, Wei, Y.; Deb, I.; Yoshikai, N. *J. Am. Chem. Soc*. **2012**, *134*, 9098. (i) For **1s**, Kayukova, L. A.; Erzhanov, K. B.; Umarova, Z. N. *Zh. Org. Khim*. **1988**, *24*, 127. (j) For **1u**, Imamoto, T.; Iwadate, N.; Yoshida, K. *Org. Lett.* **2006**, *8*, 2289. (k) For **1v**, Samec, J. S. M.; Bäckvall, J.-E. *Chem. Eur. J.* **2002**, *8*, 2955. (l) For **1w**, **1x**, Han, Z.; Wang, Z.; Zhang, X.; Ding, K. *Angew. Chem. Int. Ed*. **2009**, *48*, 5345. (m) For **1y**, Vachal, P.; Jacobsen, E. N. *Org. Lett*. **2000**, *2*, 867. (n) For **1z**, Dalili, S.; Yudin, A. K. *Org. Lett.* **2005**, *7*, 1161. (o) For **1af**, Stauffer, S. R.; Sun, J.; Katzenellenbogen, B. S.; Katzenellenbogen, J. A. *Bioorg. Med. Chem*. **2000**, *8*, 1293.

(3) Malkov, A. V.; Vrankova, K.; Stoncius, S.; Kocovsky, P. *Chem. Eur. J.* **2008**, *14*, 8082.

(4) (a) For **1aa**, Rathelot, P.; Vanelle, P.; Gasquet, M.; Delmas, F.; Crozet M. P.; Timon-David, P.; Maldonado, J. *Eur. J. Med. Chem.* **1995**, *30*, 503. (b) For **1ab**, Martin, N. H.; Jefford, C. W. *Helv. Chim. Acta*. **1982**, *65*, 762. (c) For **1ad**, Reeves, J. T.; Tan, Z.; Han, Z. S.; Li, G.; Zhang, Y.; Xu, Y.; Reeves, D. C.; Gonnella, N. C.; Ma. S.; Lee, H.; Lu, B. Z.; Senanayake, C. H. *Angew. Chem. Int. Ed.* **2012**, *51*, 1400. (d) For **1ad**, corresponding aldehyde (1.3 equiv) and *p*-anisidine (1.0 equiv) was added and stirred in toluene for 1 hour at room temperature, and then subjected to standard reaction conditions in one pot with **2b**. (e) For **1ag**, Bremner, J. B.; Coban, B.; Griffith, R.; Groenewoud, K. M.; Yates, B. F. *Bioorg. Med. Chem*. **2000**, *8*, 201. (f) For **1ah**, Wu, J.; Talwar, D.; Johnston, S.; Yan, M.; Xiao, J. *Angew. Chem. Int. Ed*. **2013**, *52*, 6983.

(5) (a) For **2b**, **2f**, Davies, J. R.; Kane, P. D.; Moody, C. J. *Tetrahedron* **2004**, *60*, 3967. (b) For **2d**, **2e**, **2i**, **2n**, Jiang, Y.; Khong, V. Z. Y.; Lourdasamy, E.; Park, C.-M. *Chem. Commun*. **2012**, *48*, 3133. (c) For **2g**, Erhunmwunse, M. O.; Steel, P. G. *J. Org.*

Chem. **2008**, *73*, 8675. (d) For **2h**, Peng, C.; Cheng, J.; Wang, J. *J. Am. Chem. Soc.* **2007**, *129*, 8708. (e) For **2m**, Phun, L. H.; Patil, D. V.; Cavitt, M. A.; France, S. *Org. Lett.* **2011**, *13*, 1952. (f) For **2o**, Honey, M. A.; Pasceri, R.; Lewis, W.; Moody, C. J. *J. Org. Chem.* **2012**, *77*, 1396. (g) For **2p**, Linder, J.; Garner, T. P.; Williams, H. E. L.; Searle, M. S.; Moody, C. J. *J. Am. Chem. Soc.* **2011**, *133*, 1044. (h) For **2q**, Sharpe, R. J.; Malinowski, J. T.; Johnson, J. S. *J. Am. Chem. Soc.* **2013**, *135*, 17990.

(6) CCDC 1040843 contains the supplementary crystallographic data for this paper. These data can be obtained free of charge from The Cambridge Crystallographic Data Centre via www.ccdc.cam.ac.uk/data_request/cif.

(7) Banwell, M. G.; Hamel, E.; Hockless, D. C. R.; Verdiner-Pinard, P.; Willis, A. C.; Wong, D. J. *Bioorg. Med. Chem.* **2006**, *14*, 4627.

(8) Rostovskii, N. V.; Novikov, M. S.; Khlebnikov, A. F.; Khlebnikov, V. A.; Korneev, S. M. *Tetrahedron* **2013**, *69*, 4292.

(9) CCDC 1063222 contains the supplementary crystallographic data for this paper. These data can be obtained free of charge from The Cambridge Crystallographic Data Centre via www.ccdc.cam.ac.uk/data_request/cif.

(10) Handy, S. T.; Zhang, Y.; Bregman, H. *J. Org. Chem.* **2004**, *69*, 2362.

^{1}H and ^{13}C NMR Spectra

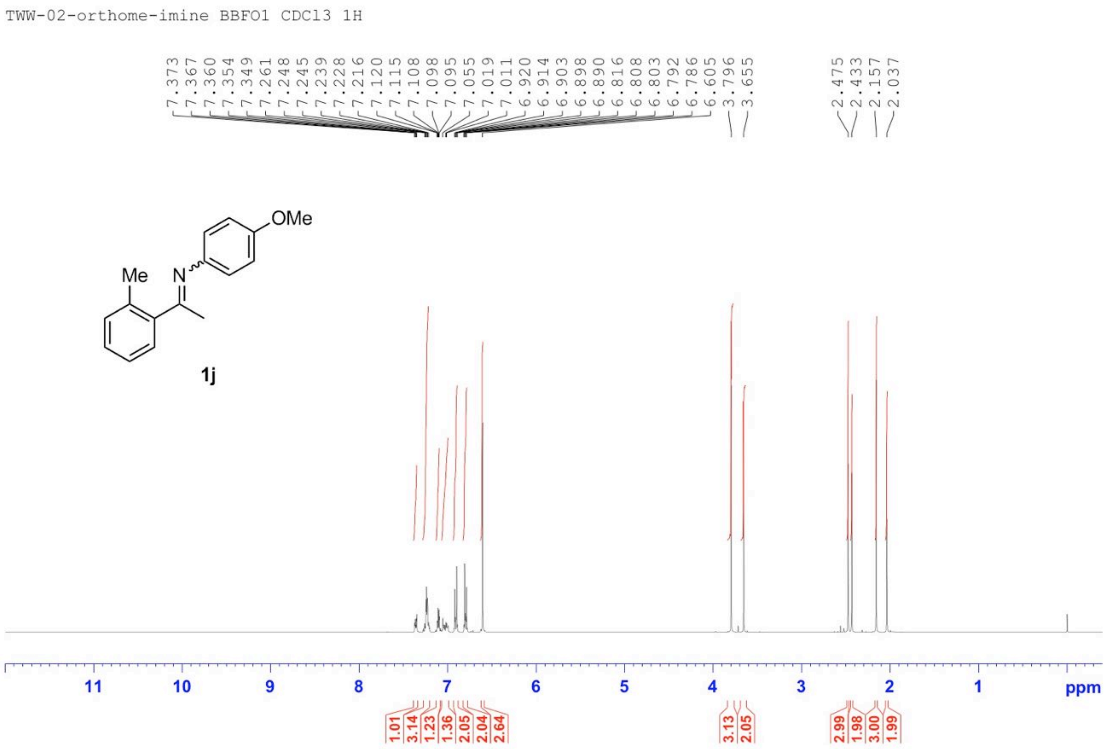

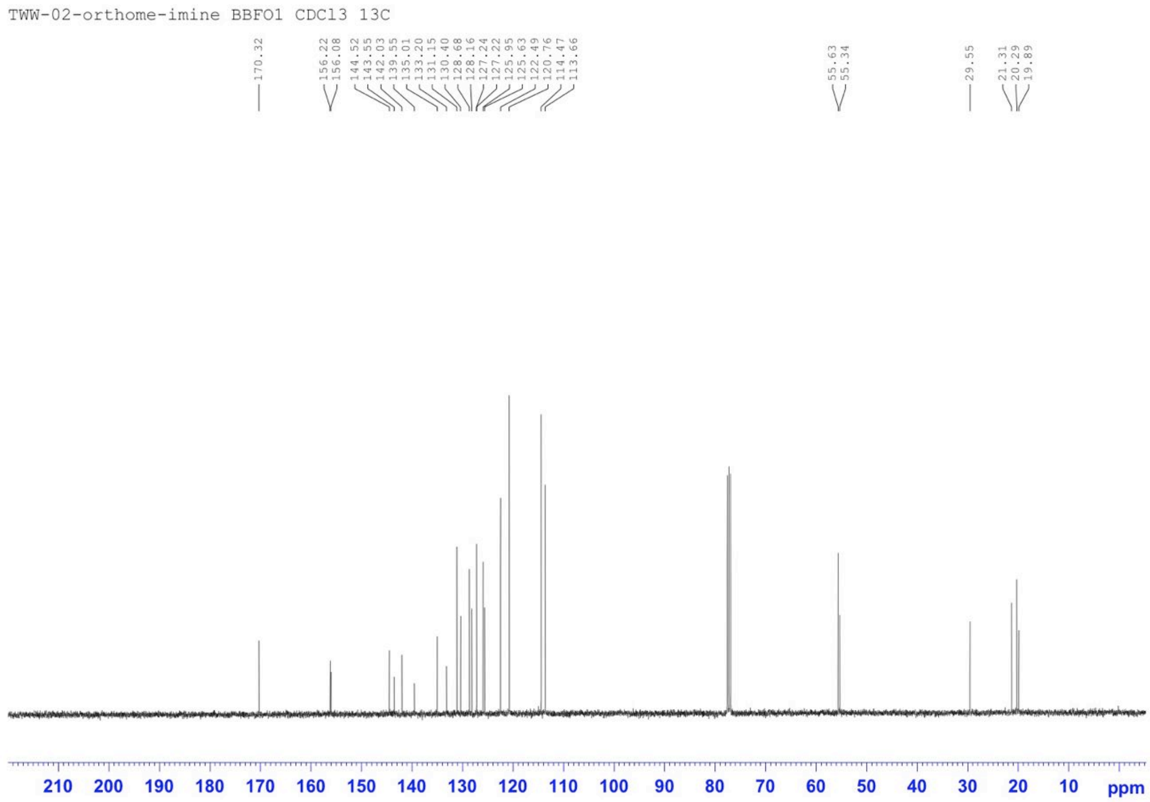

cyclohep-imine

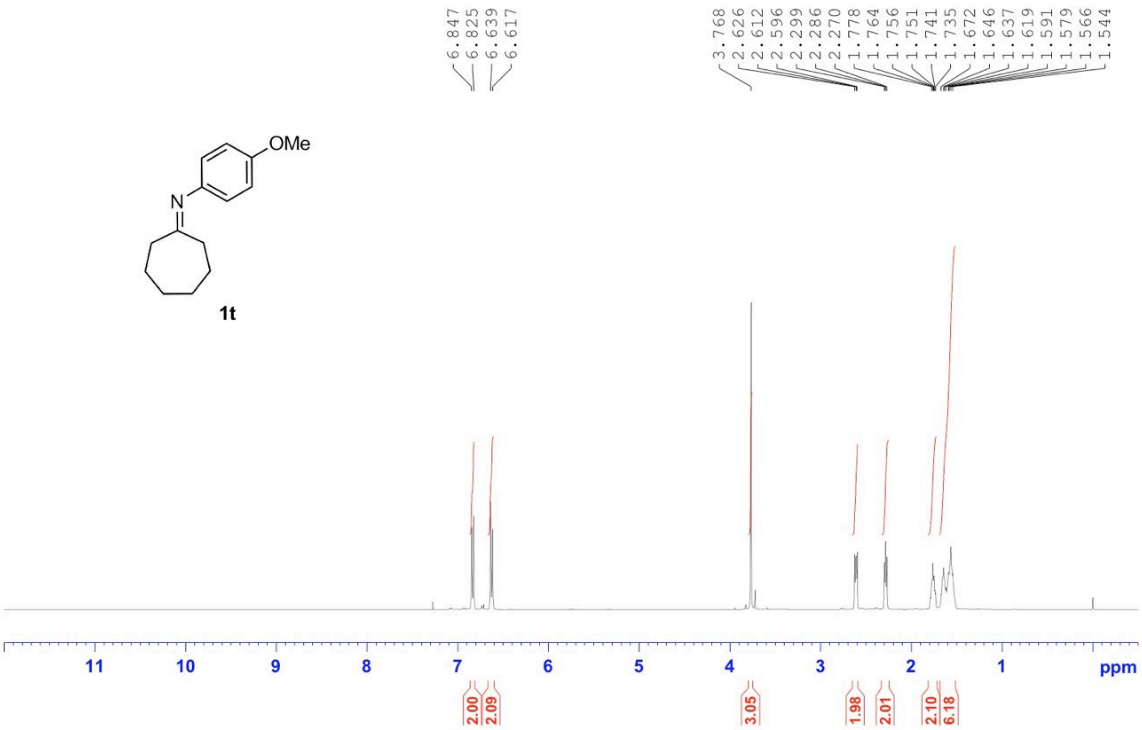

cyclohep-imine

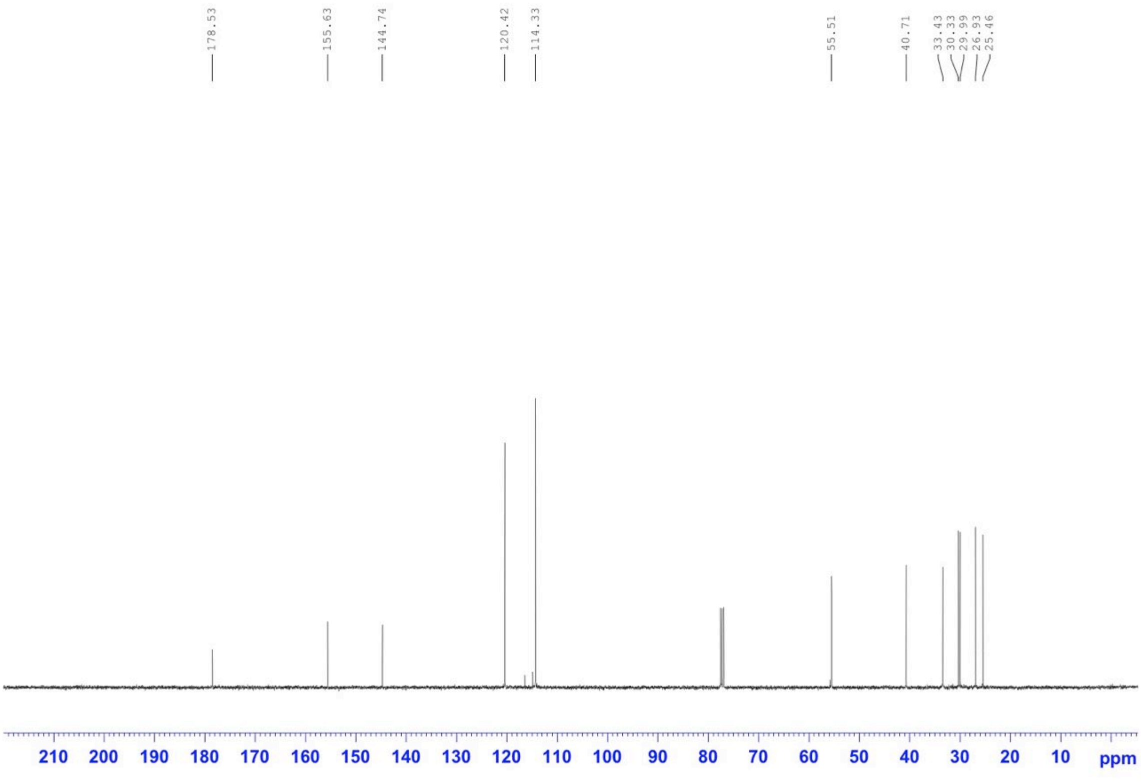

TWW-02-205, 1H, BBFO1, CDCl3

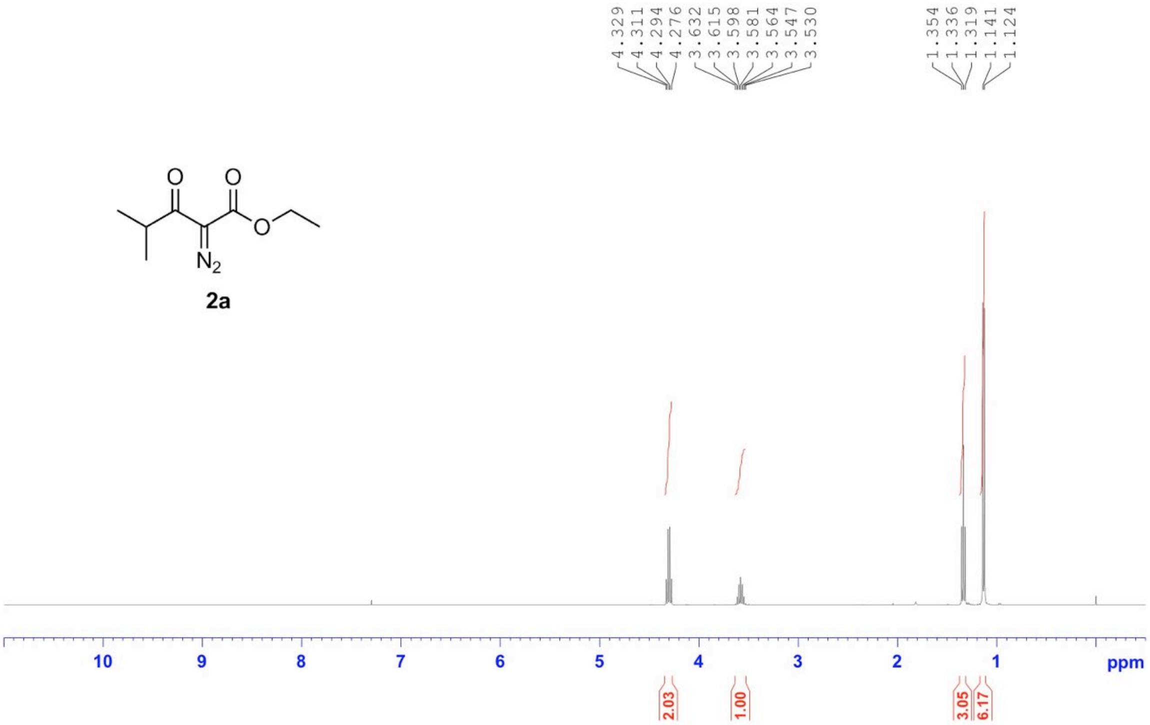

2a

TWW-02-205x, 13C, BBFO1, CDCl3

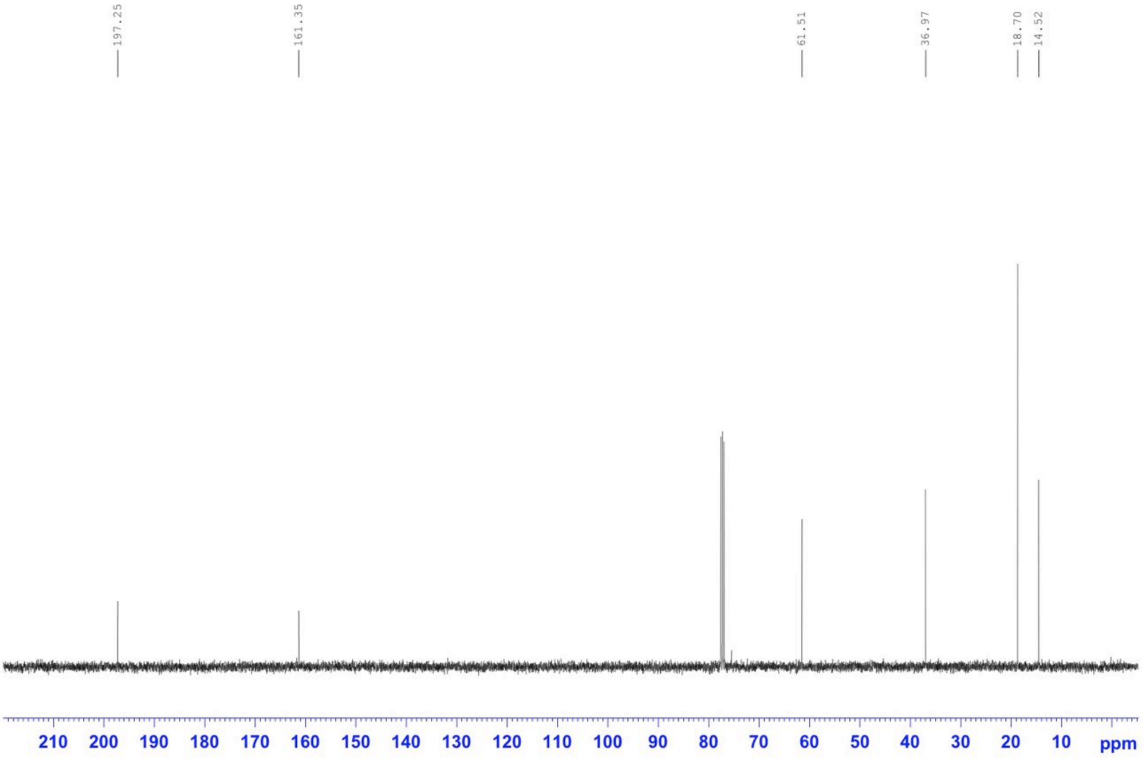

TWW-02-191, 1H BBFO1, CDCl3

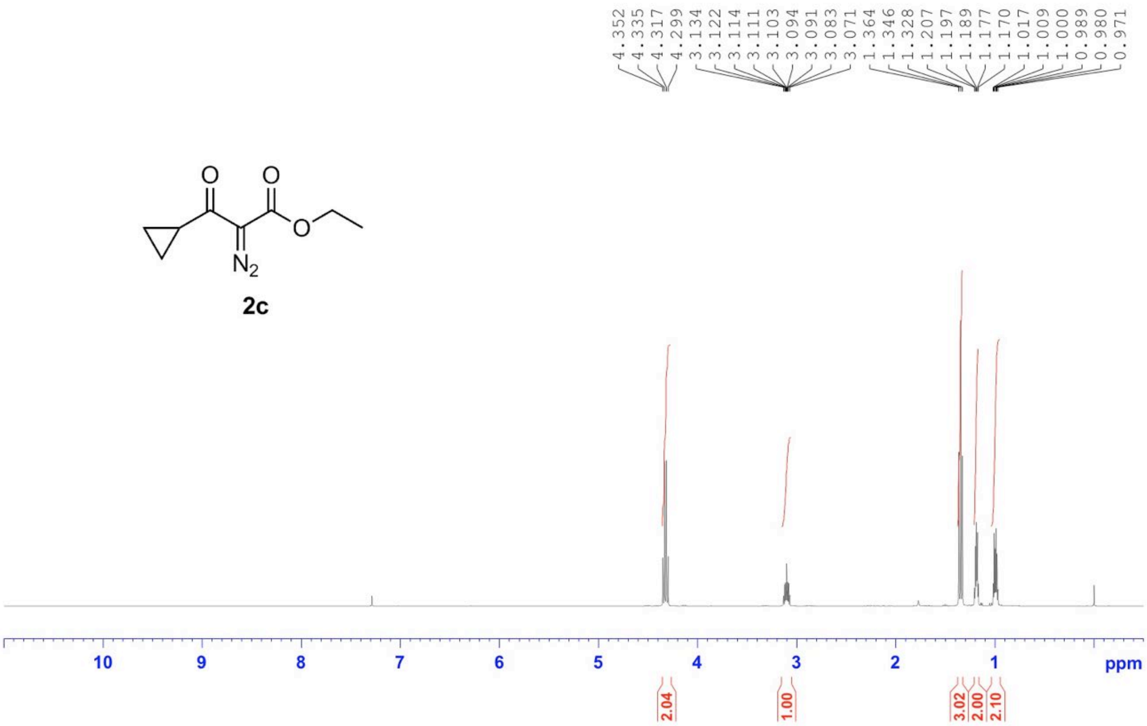

TWW-02-191x, 13C, BBFO1, CDCl3

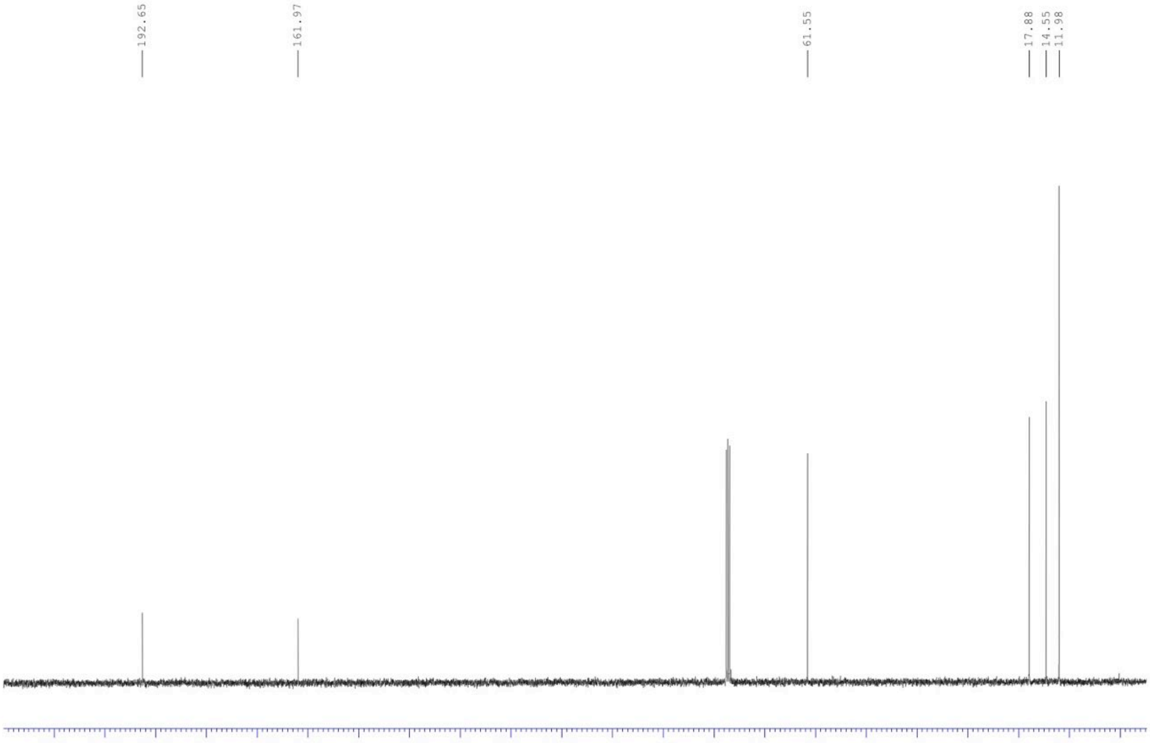

TWW-02-192, 1H BBFO1, CDCl3

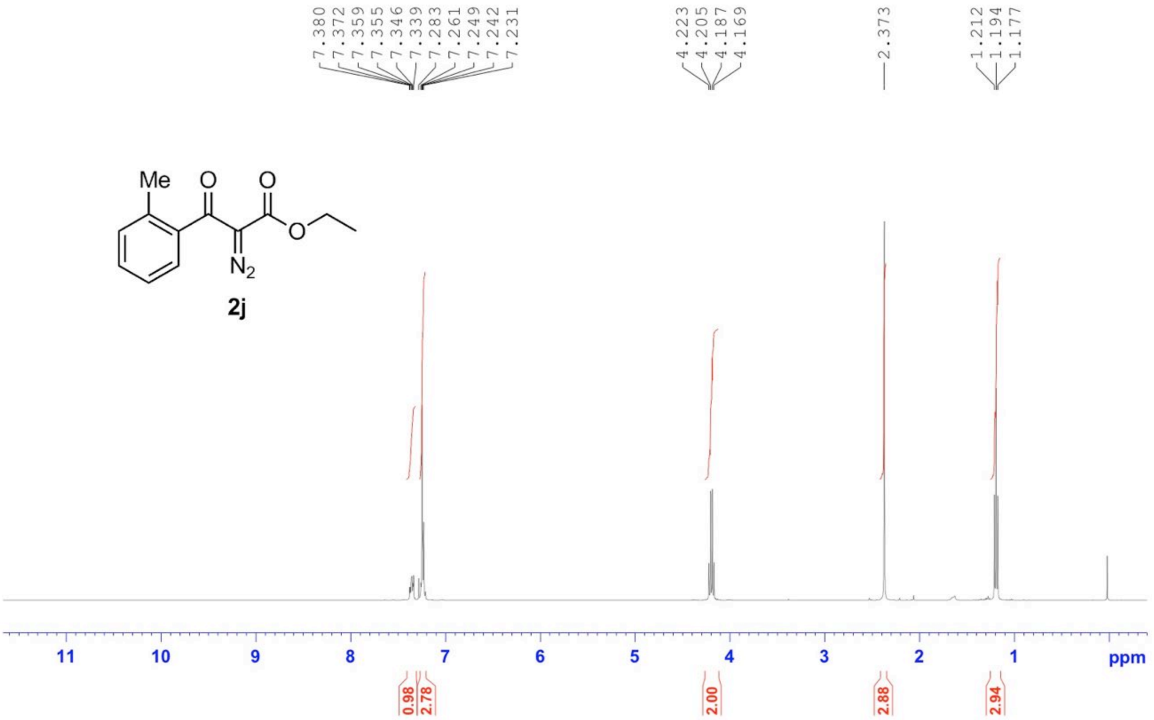

2j

TWW-02-192, 13C BBFO1, CDCl3

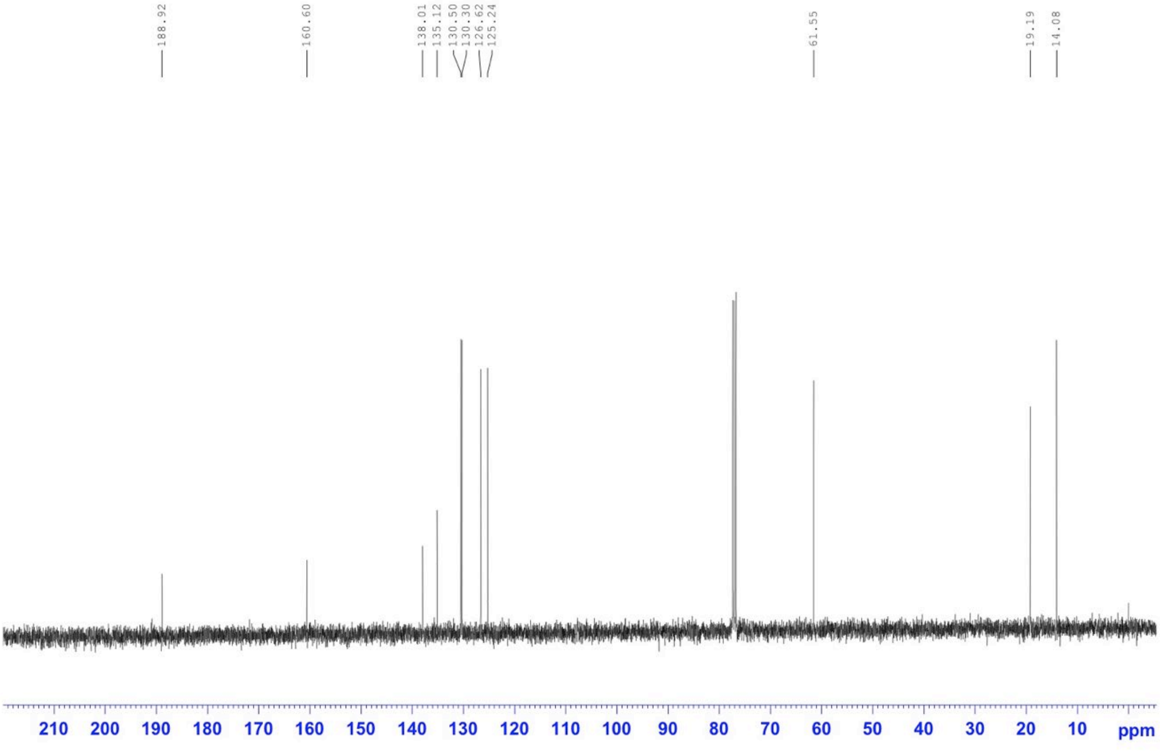

TWW-02-206, 1H, BBFO1, CDCl3

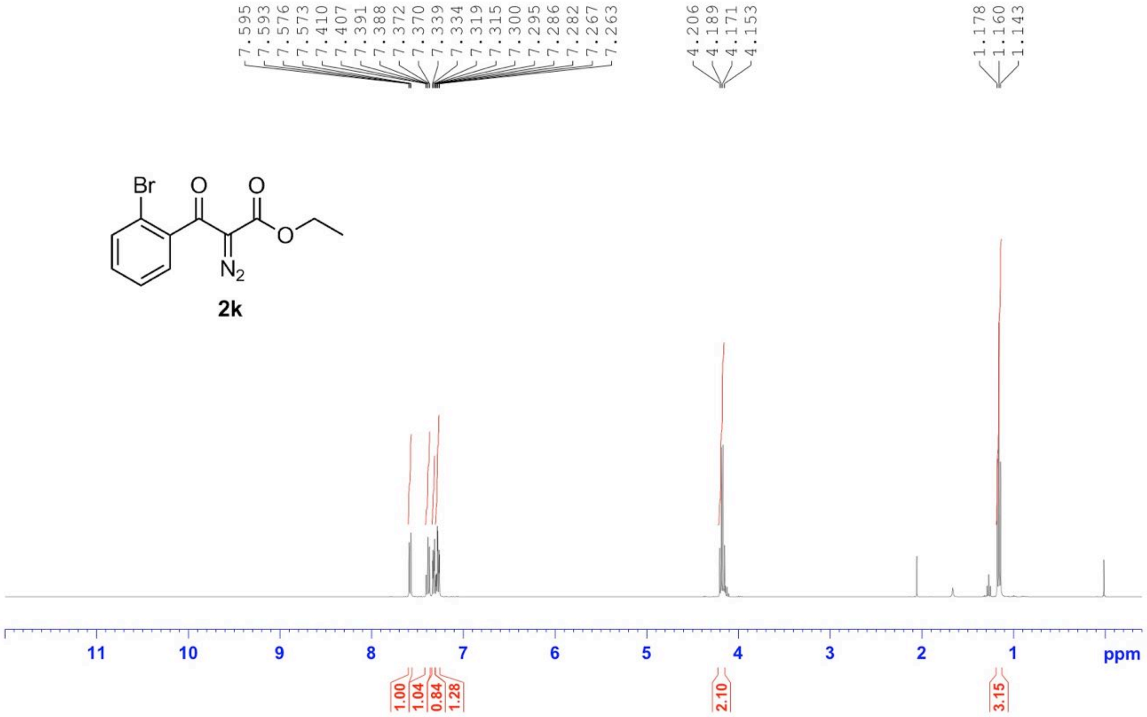

TWW-02-206, 13C, BBFO1, CDCl3

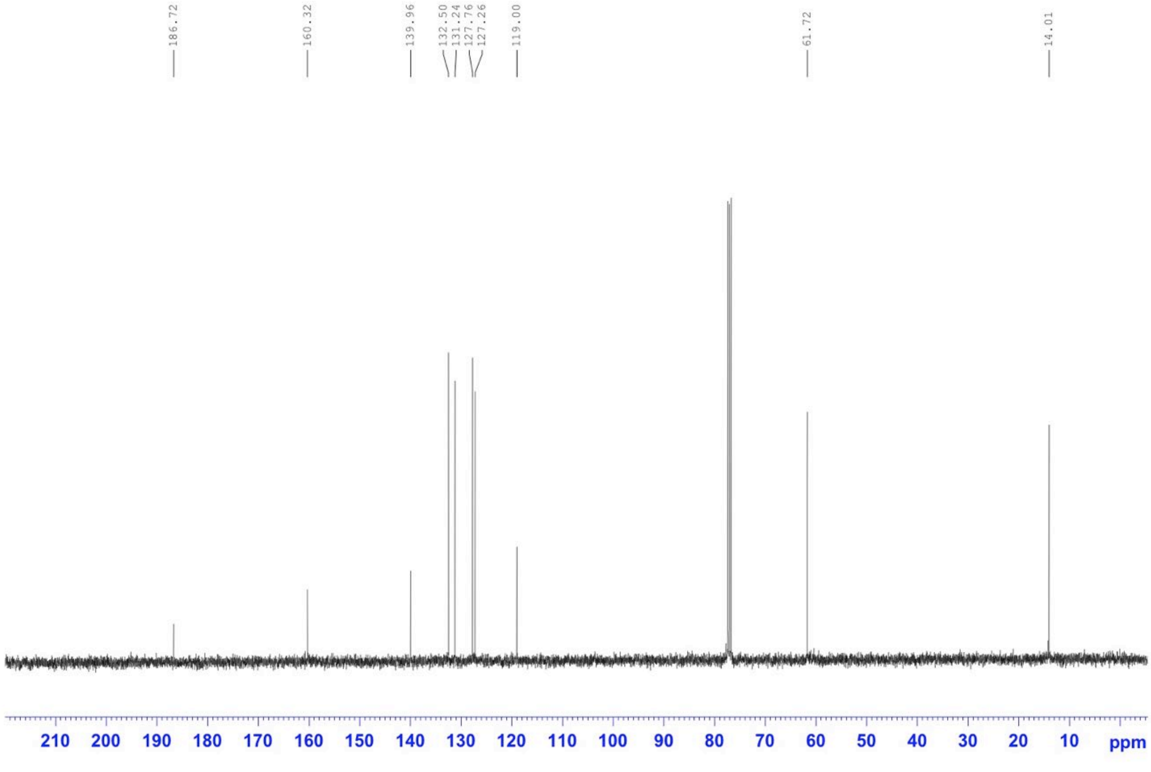

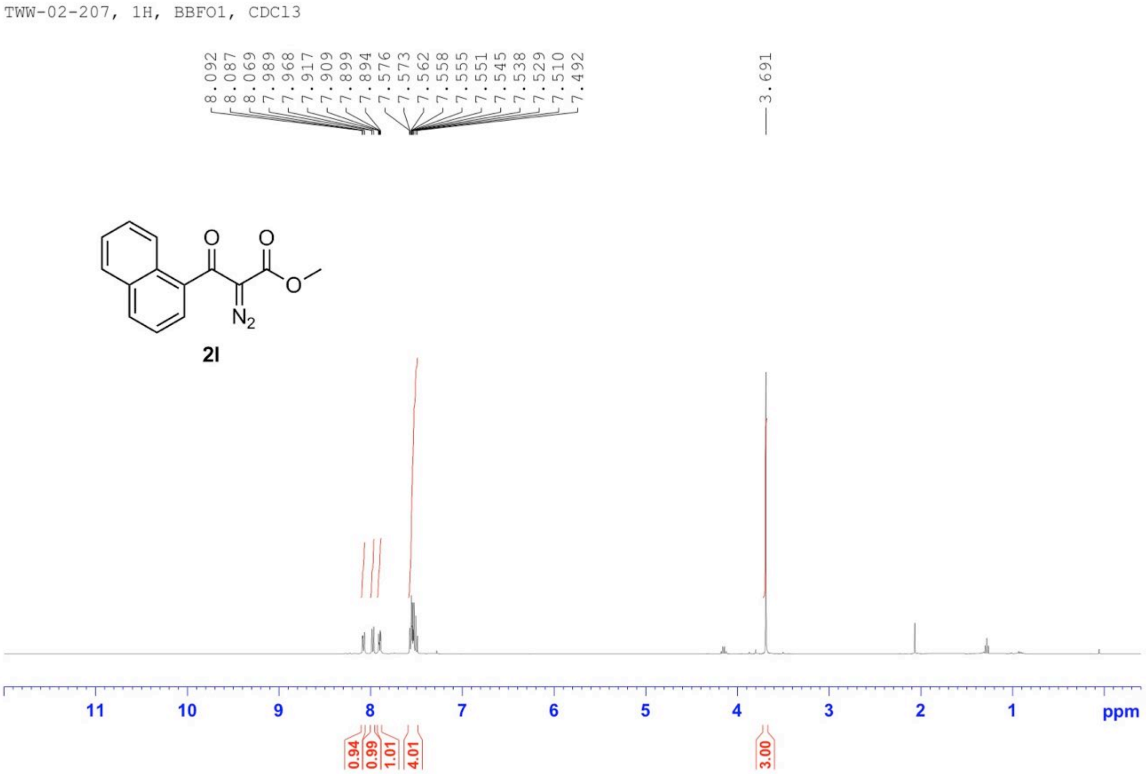
TWW-02-207, 1H, BBFO1, CDCl3
8.092
8.087
8.069
7.989
7.968
7.917
7.909
7.899
7.894
7.576
7.573
7.562
7.558
7.555
7.551
7.545
7.538
7.529
7.510
7.492
3.691
O
O
O
N2
2l
11
10
9
8
7
6
5
4
3
2
1
ppm
0.94
0.99
1.01
4.01
3.00

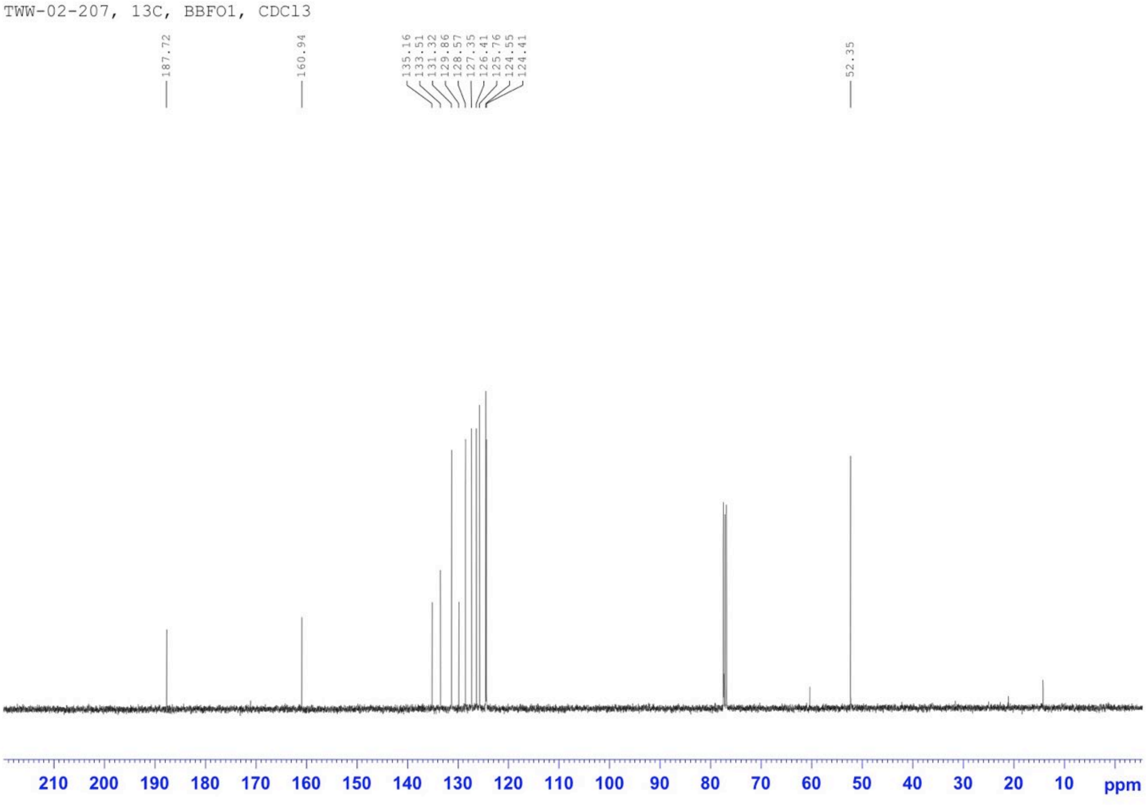
TWW-02-207, 13C, BBFO1, CDCl3
187.72
160.94
135.16
133.51
131.32
129.86
128.57
127.35
126.41
125.76
124.55
124.41
52.35
210
200
190
180
170
160
150
140
130
120
110
100
90
80
70
60
50
40
30
20
10
ppm

TWW-02-076, BBFO, 1H, CDCl3

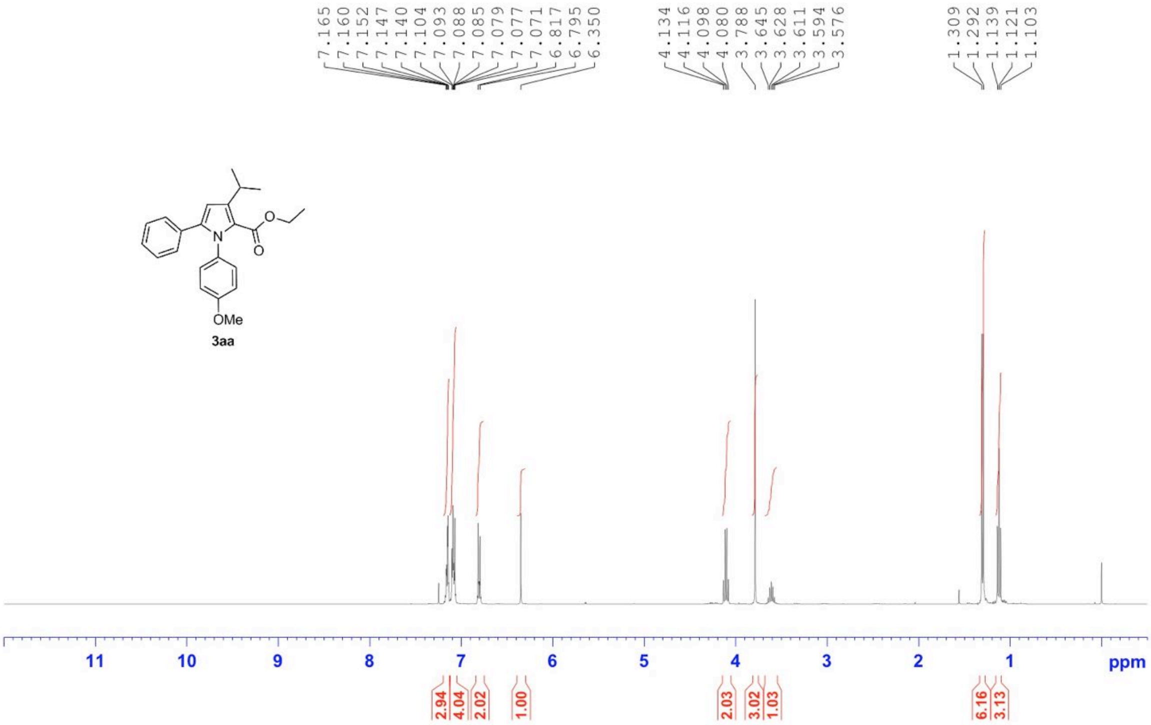

TWW-02-076, BBFO, 13C, CDCl3

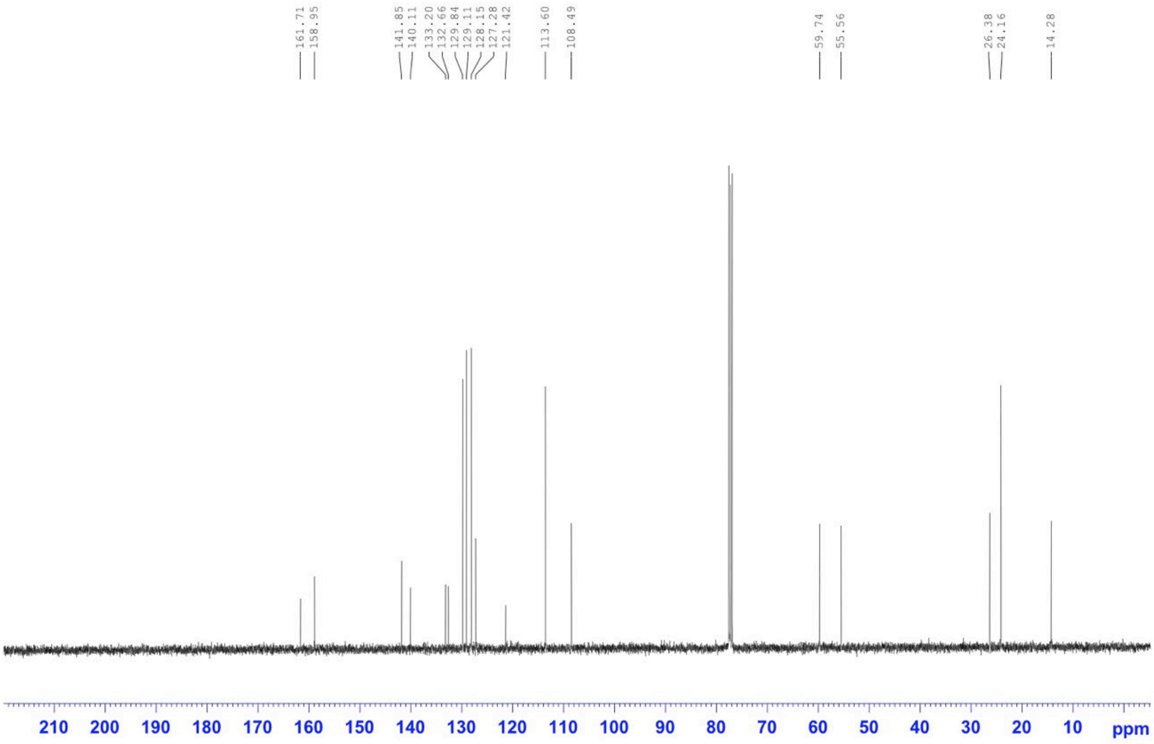

3aa-HMQC

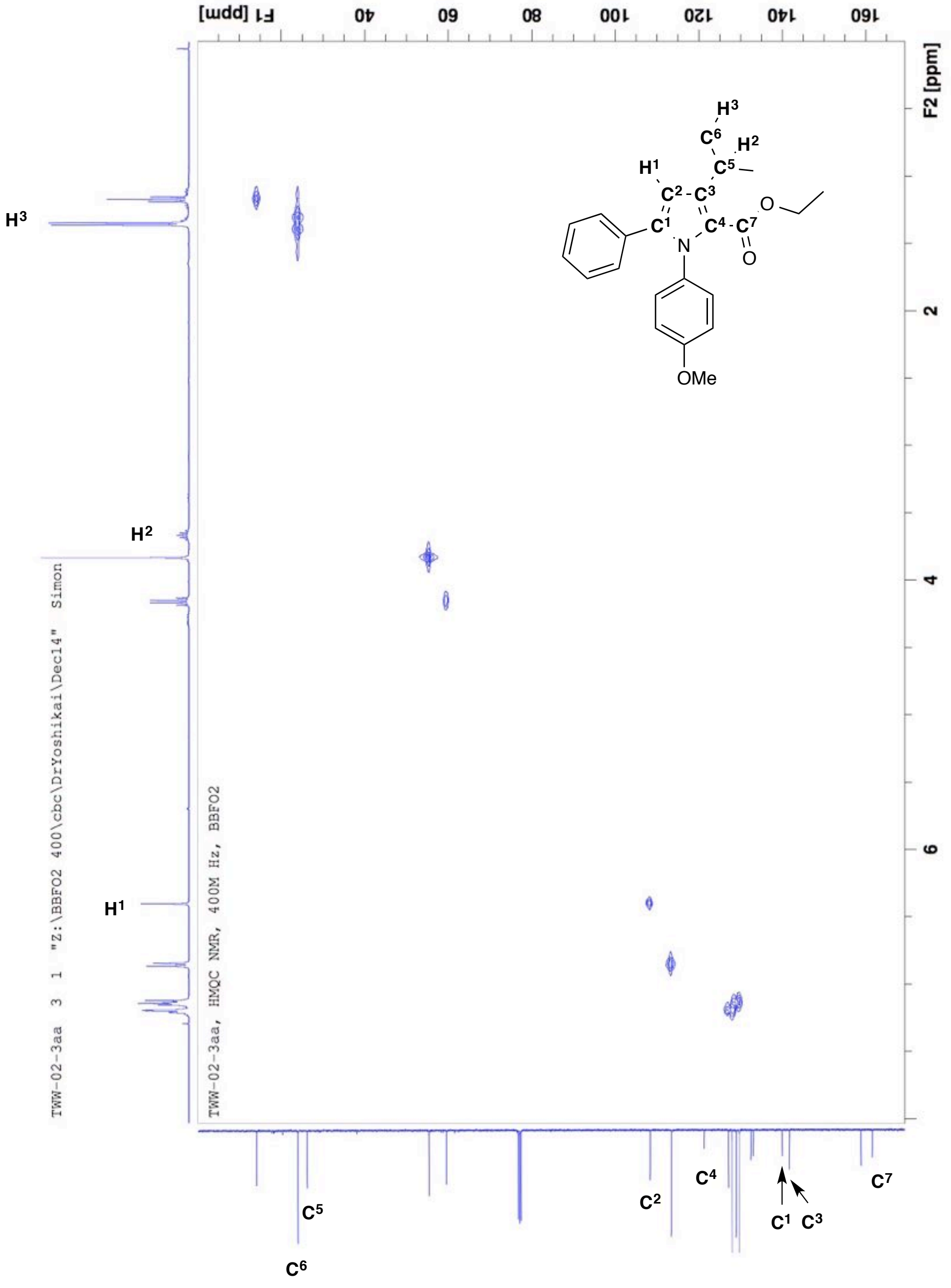
H³
H²
H¹
C⁵
C⁶
C²
C⁴
C¹
C³
C⁷
F1 [ppm]
F2 [ppm]
TWW-02-3aa, HMQC NMR, 400M Hz, BBFO2
OMe
N
O

3aa-HMBC

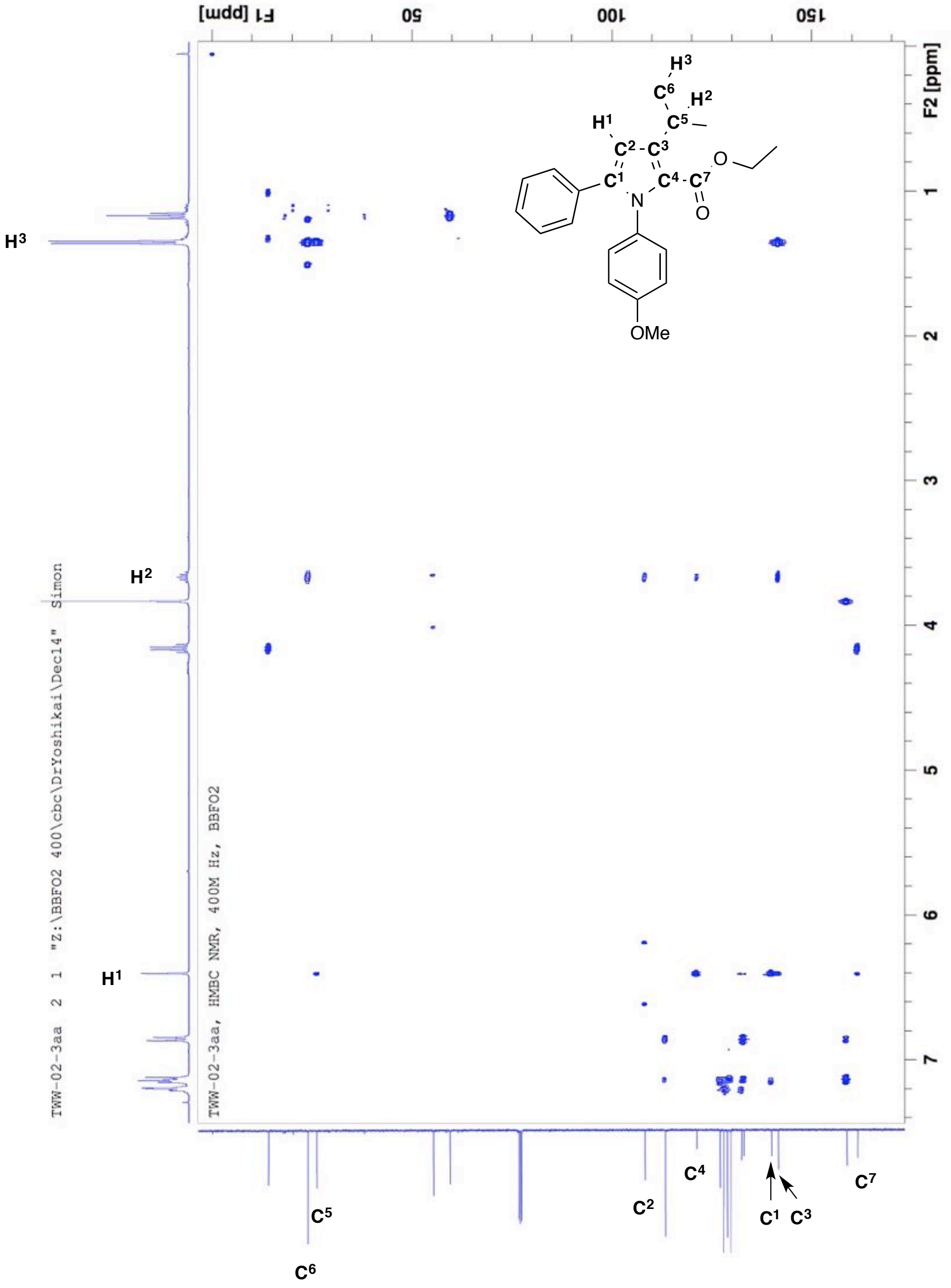
H3
H2
H1
C5
C6
C2
C4
C1
C3
C7
OMe
F1 [ppm]
F2 [ppm]
TWW-02-3aa, HMBC NMR, 400M Hz, BBFO2
TWW-02-3aa 2 1 "Z:\BBFO2 400\cbc\DrYoshikai\Dec14" Simon

TWW-02-089, BBFO, 1H, CDCl3

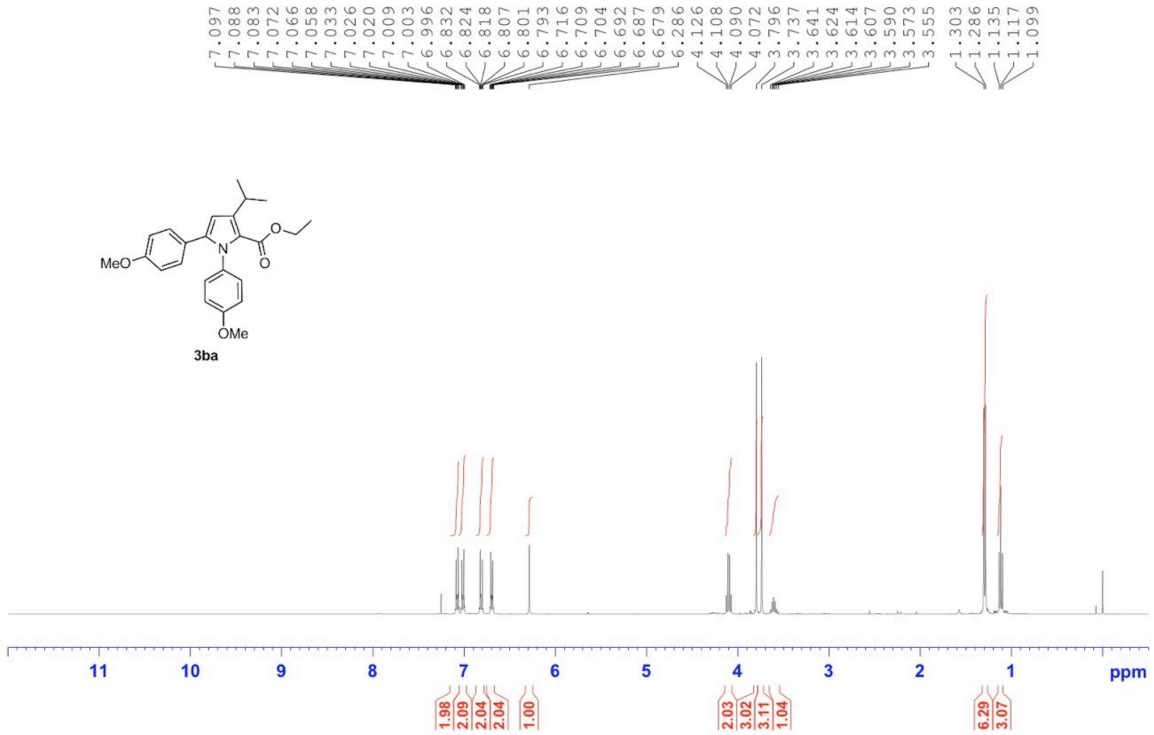

TWW-02-089, BBFO, 13C, CDCl3

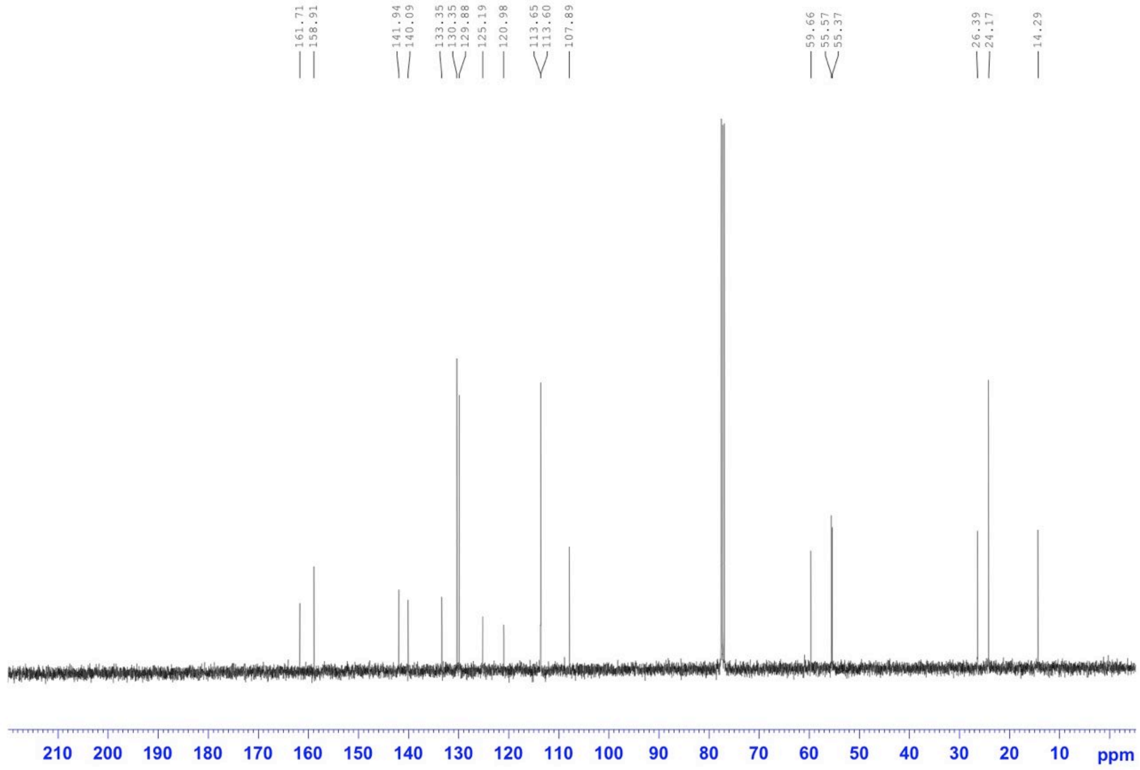

TWW-02-138 1H BBFO1 CDCl3

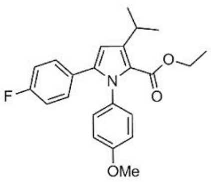

3ca

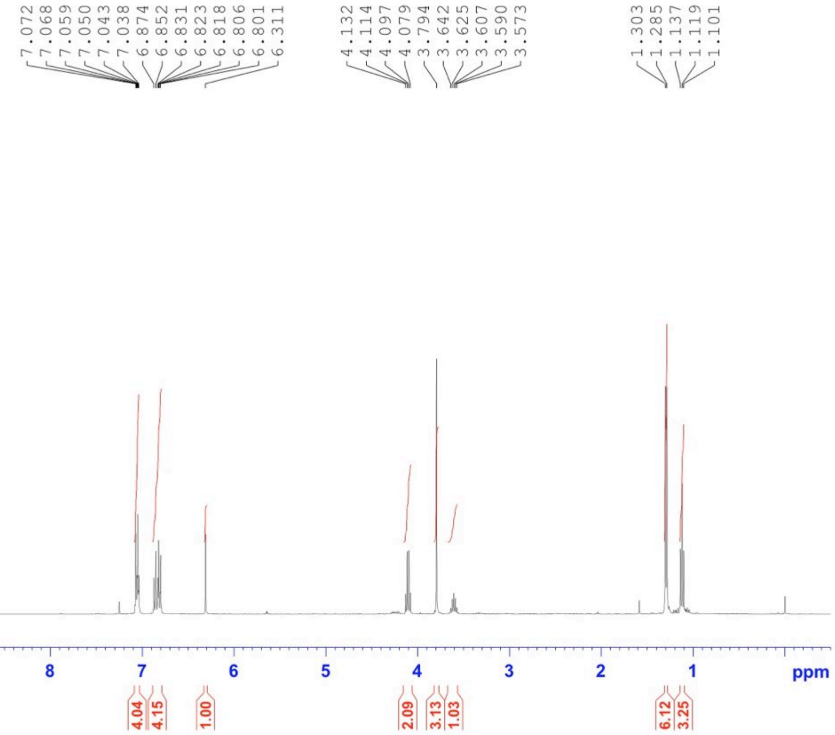

TWW-02-138 13C BBFO1 CDCl3

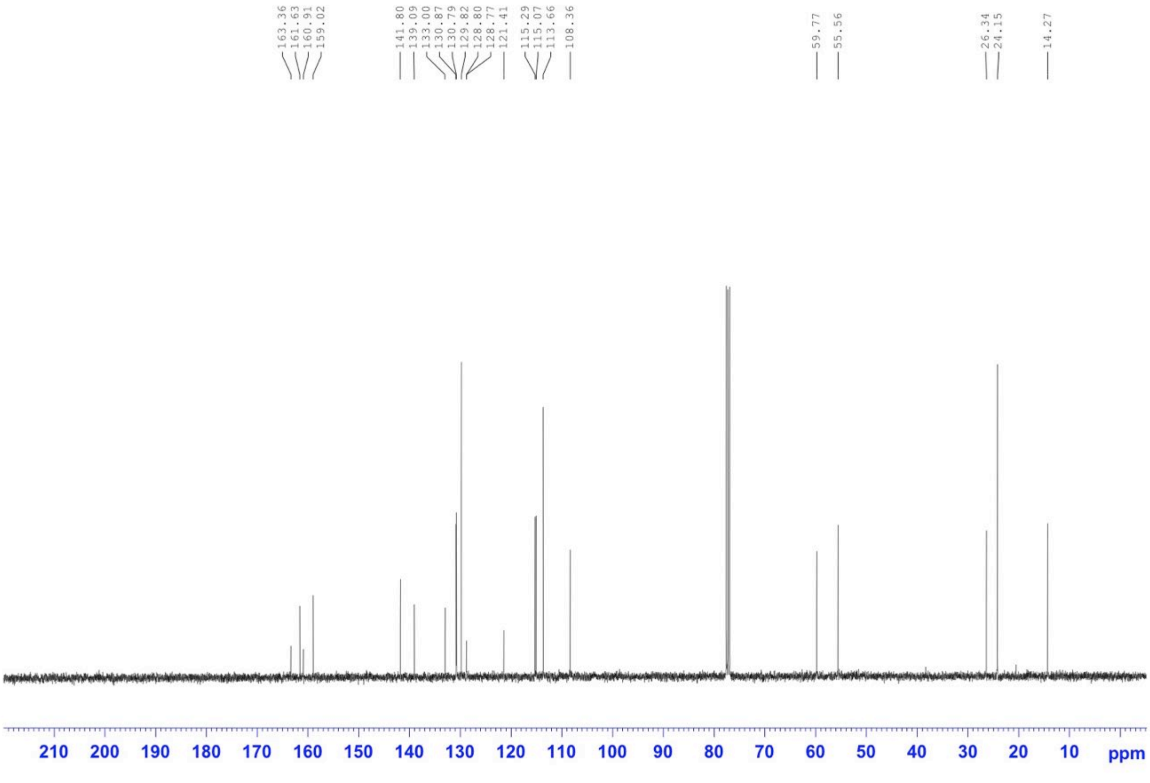

TWW-02-103, BBFO, 1H, CDCl3

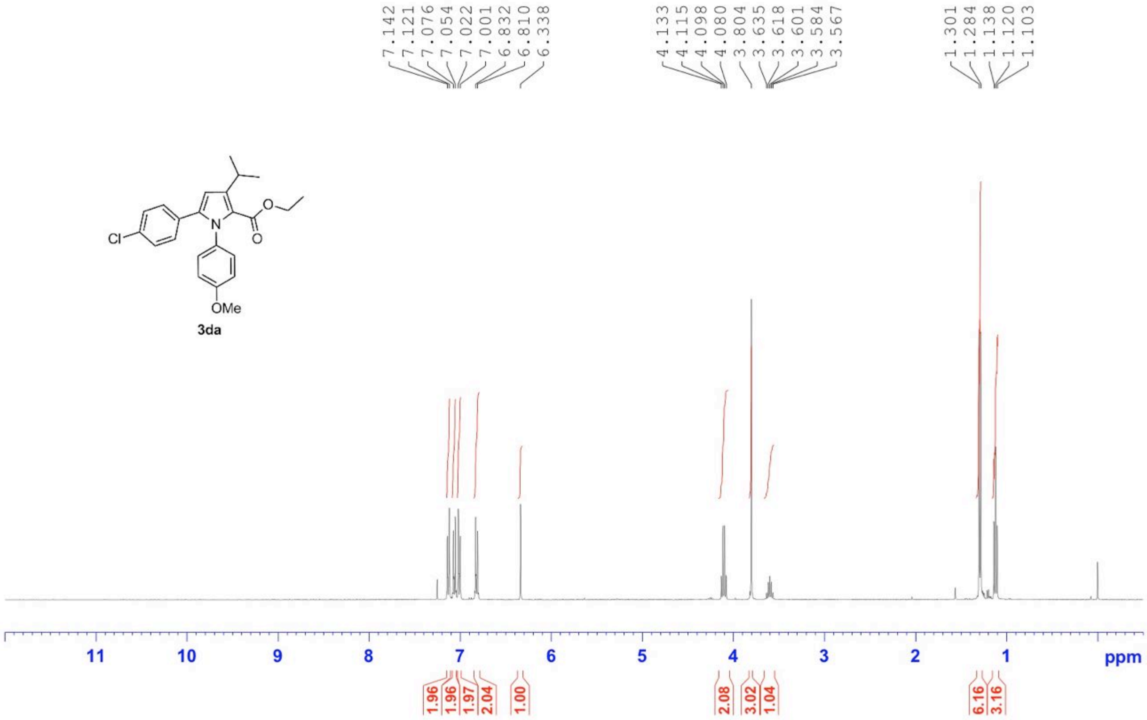

3da

TWW-02-103, BBFO, 13C, CDCl3

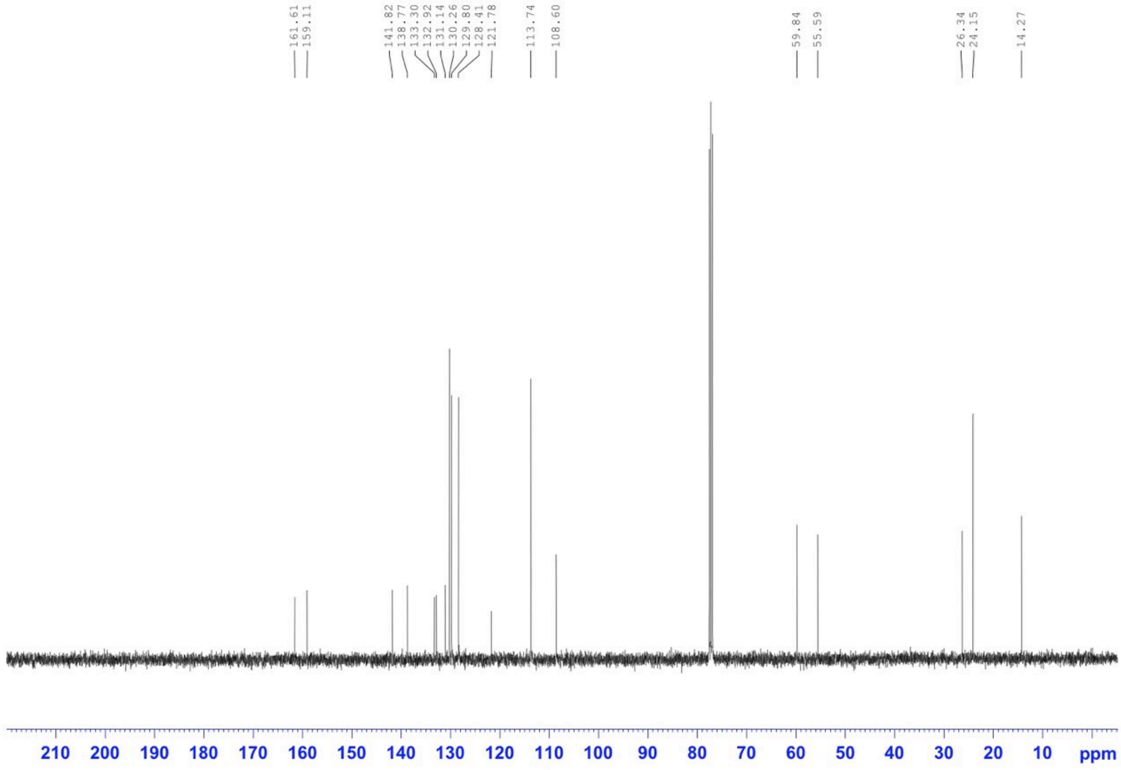

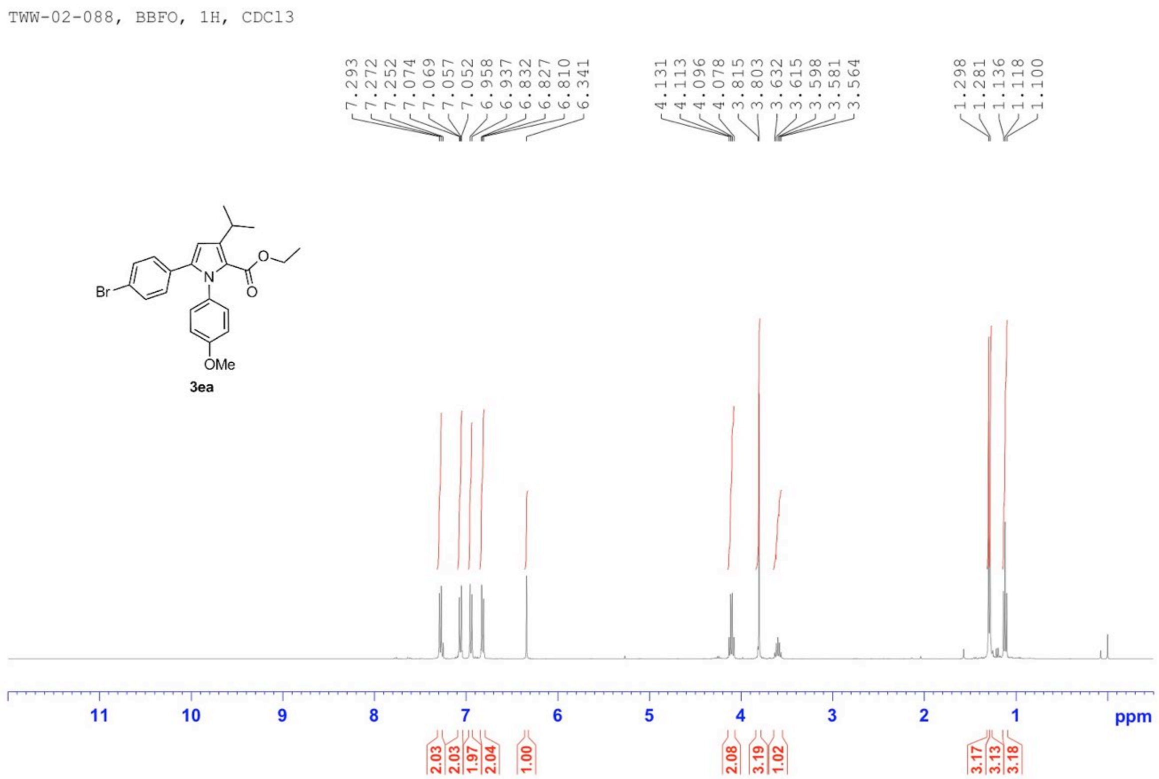
TWW-02-088, BBFO, 1H, CDCl3
7.293
7.272
7.252
7.074
7.069
7.057
7.052
6.958
6.937
6.832
6.827
6.810
6.341
4.131
4.113
4.096
4.078
3.815
3.803
3.632
3.615
3.598
3.581
3.564
1.298
1.281
1.136
1.118
1.100
Br
N
O
O
OMe
3ea
11
10
9
8
7
6
5
4
3
2
1
ppm
2.03
2.03
1.97
2.04
1.00
2.08
3.19
1.02
3.17
3.13
3.18

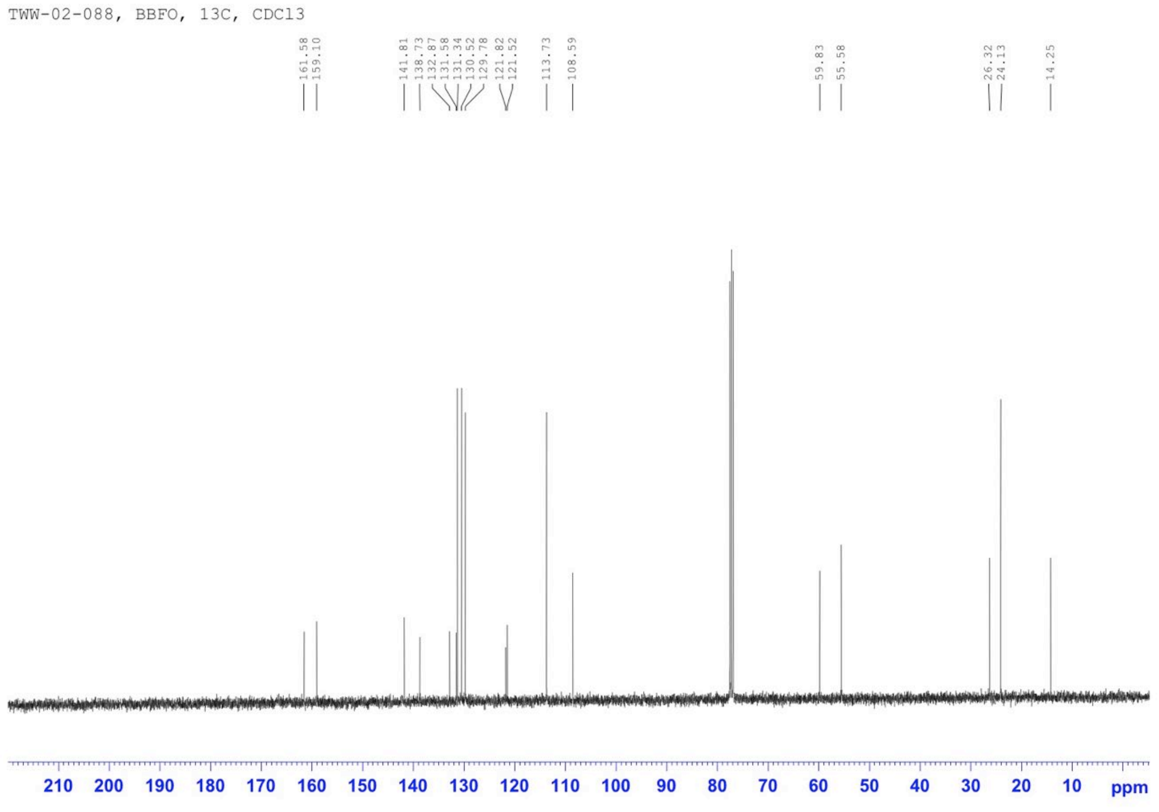
TWW-02-088, BBFO, 13C, CDCl3
161.58
159.10
141.81
138.73
132.87
131.58
131.34
130.52
129.78
121.82
121.52
113.73
108.59
59.83
55.58
26.32
24.13
14.25
210
200
190
180
170
160
150
140
130
120
110
100
90
80
70
60
50
40
30
20
10
ppm

TWW-02-139 1H BBFO1 CDCl3

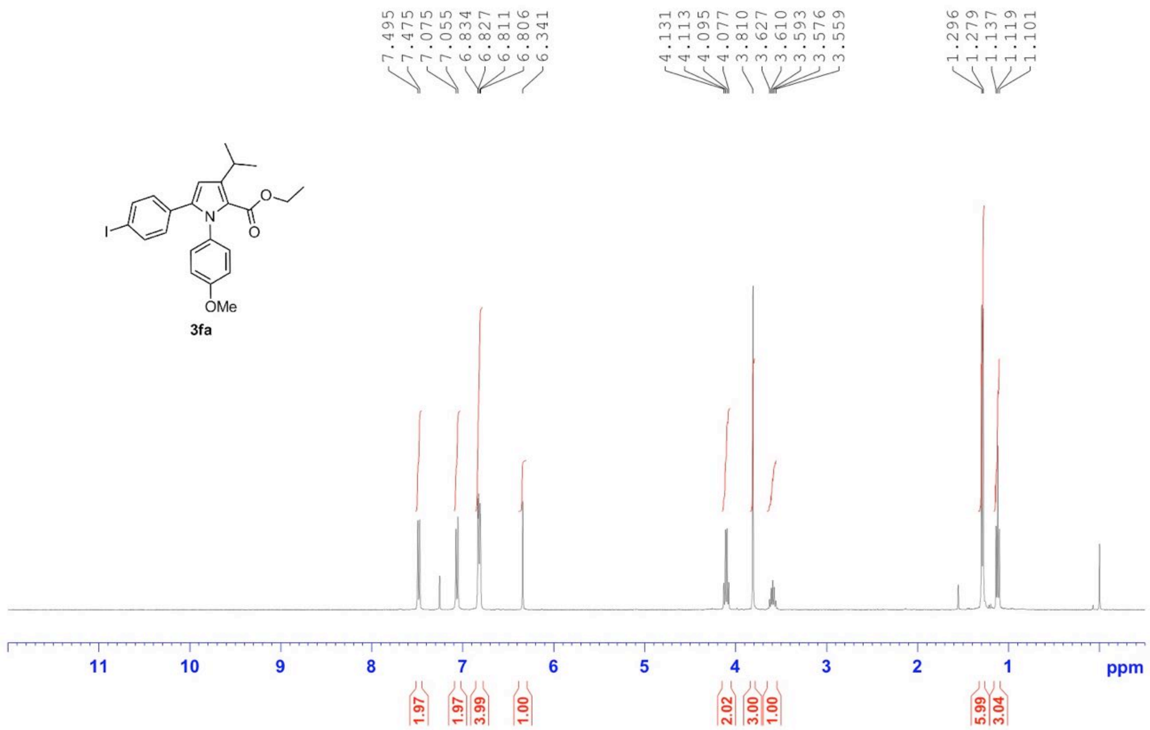

TWW-02-139 13C BBFO1 CDCl3

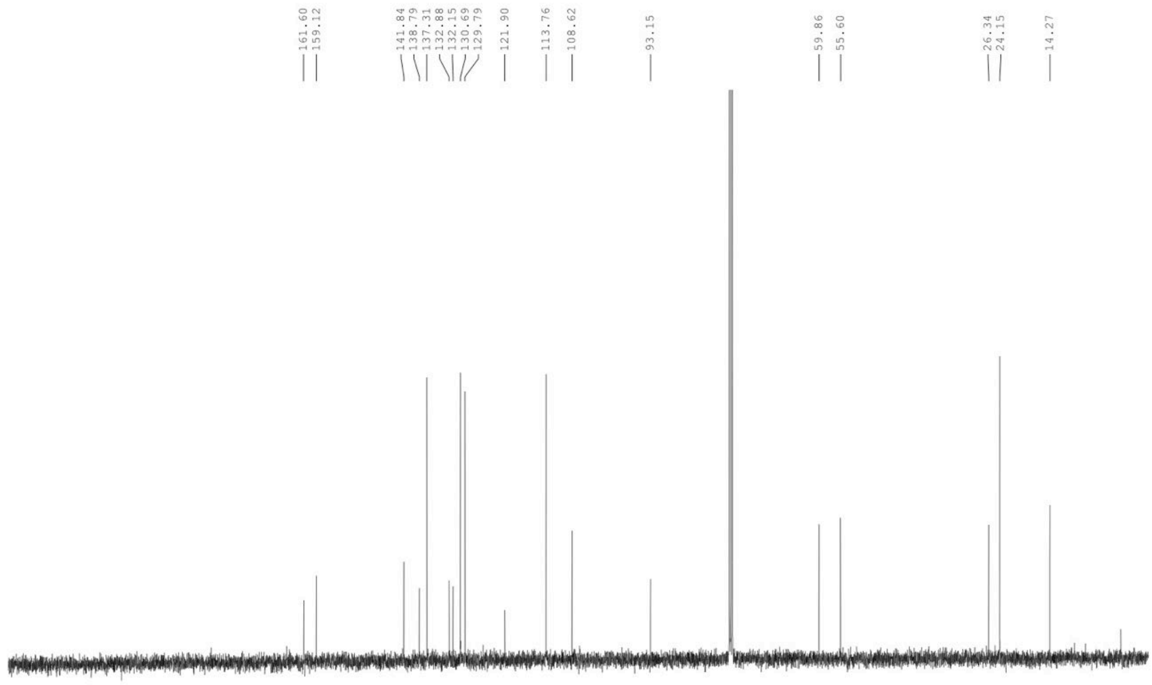

TWW-02-93-1 BBFO1 CDCl3 1H

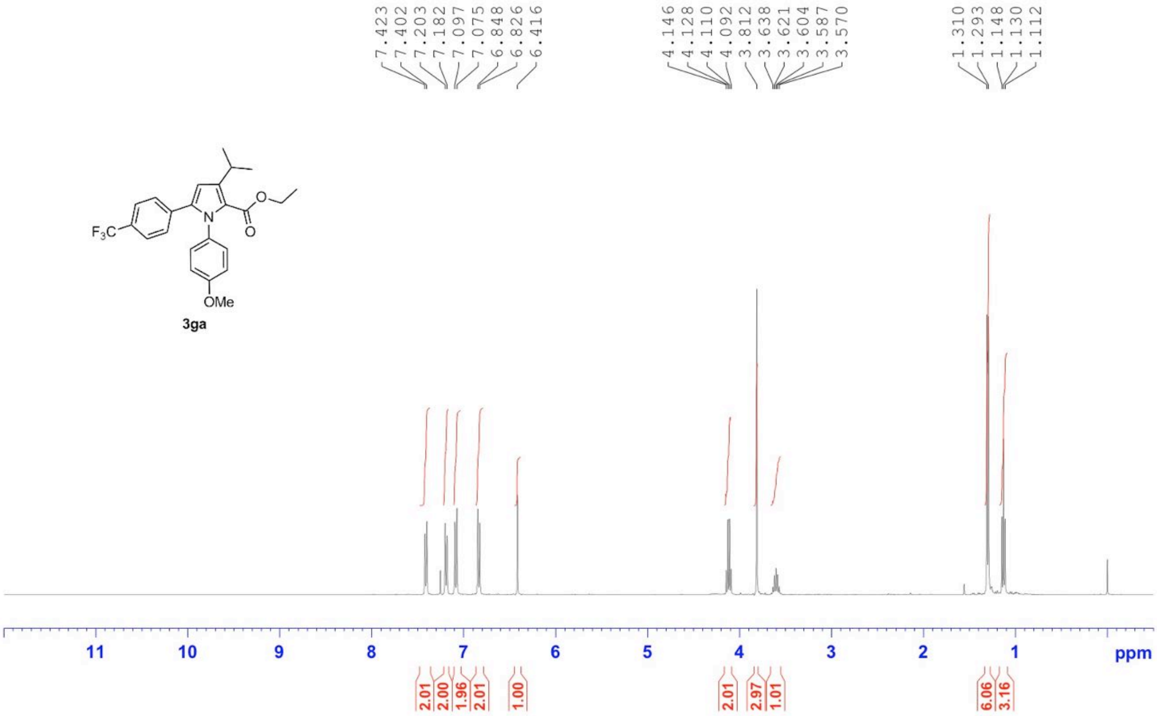

TWW-02-93-1 BBFO1 CDCl3 13C

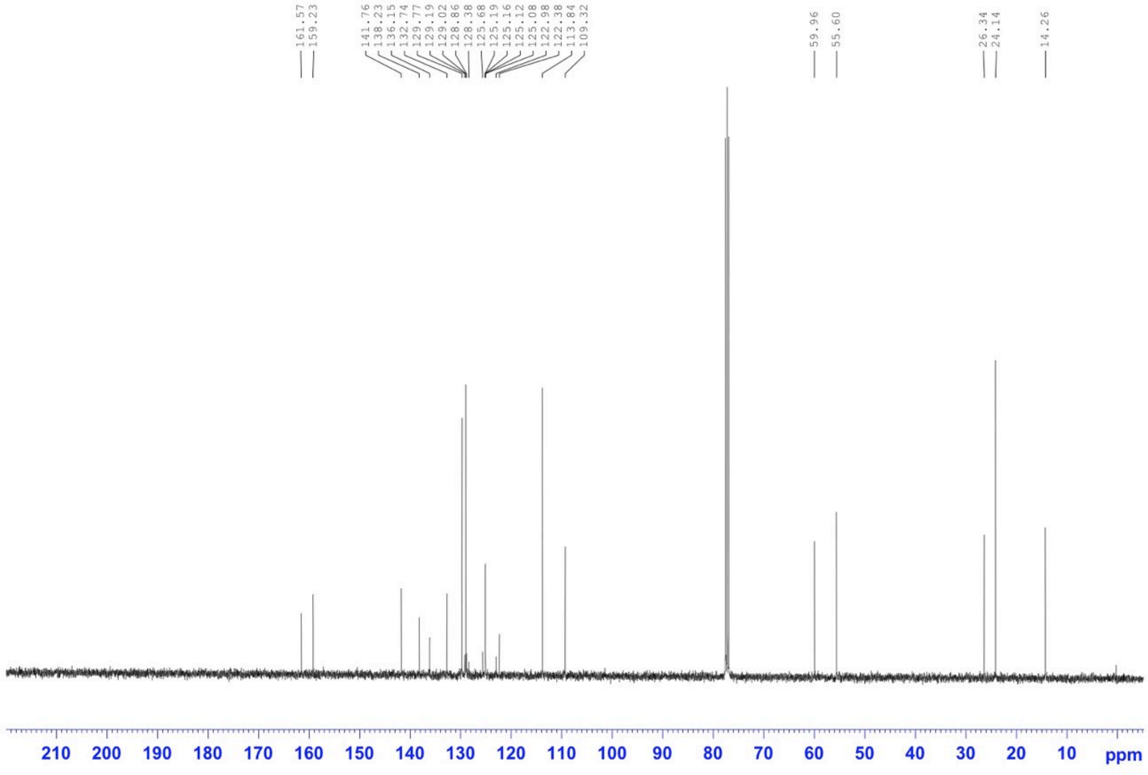

TWW-02-86-x BBFO1 CDCl3 1H

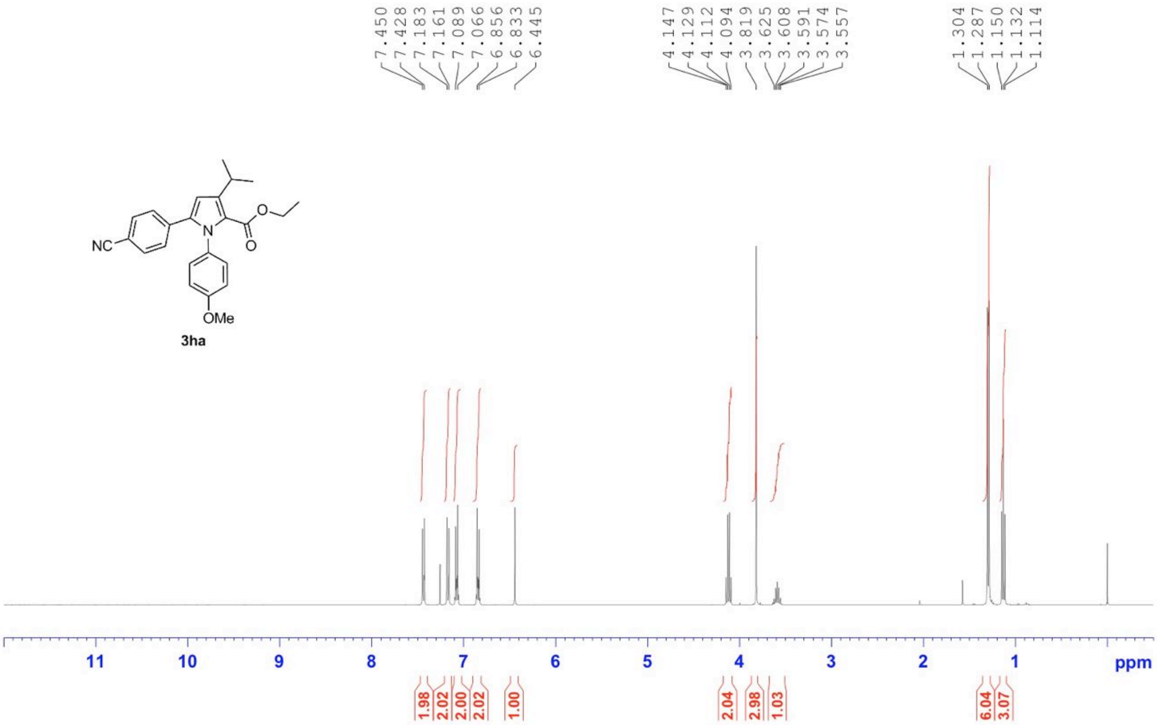

TWW-02-86-x BBFO1 CDCl3 13C

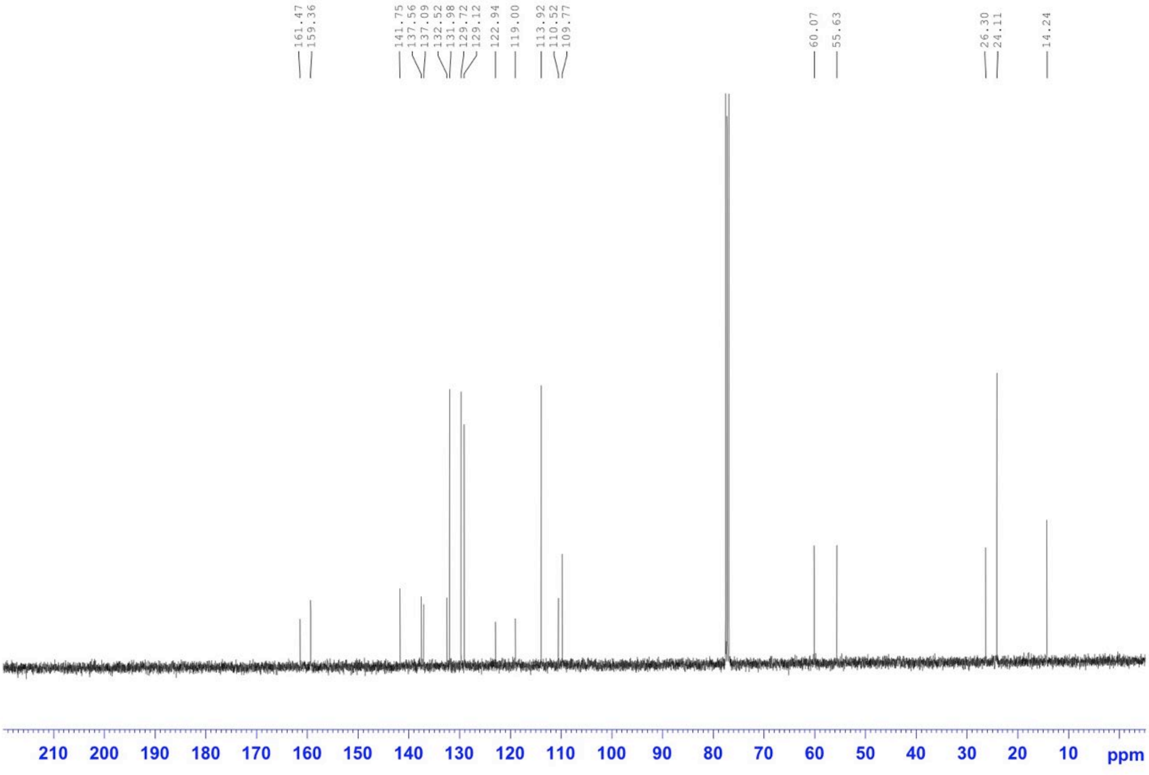

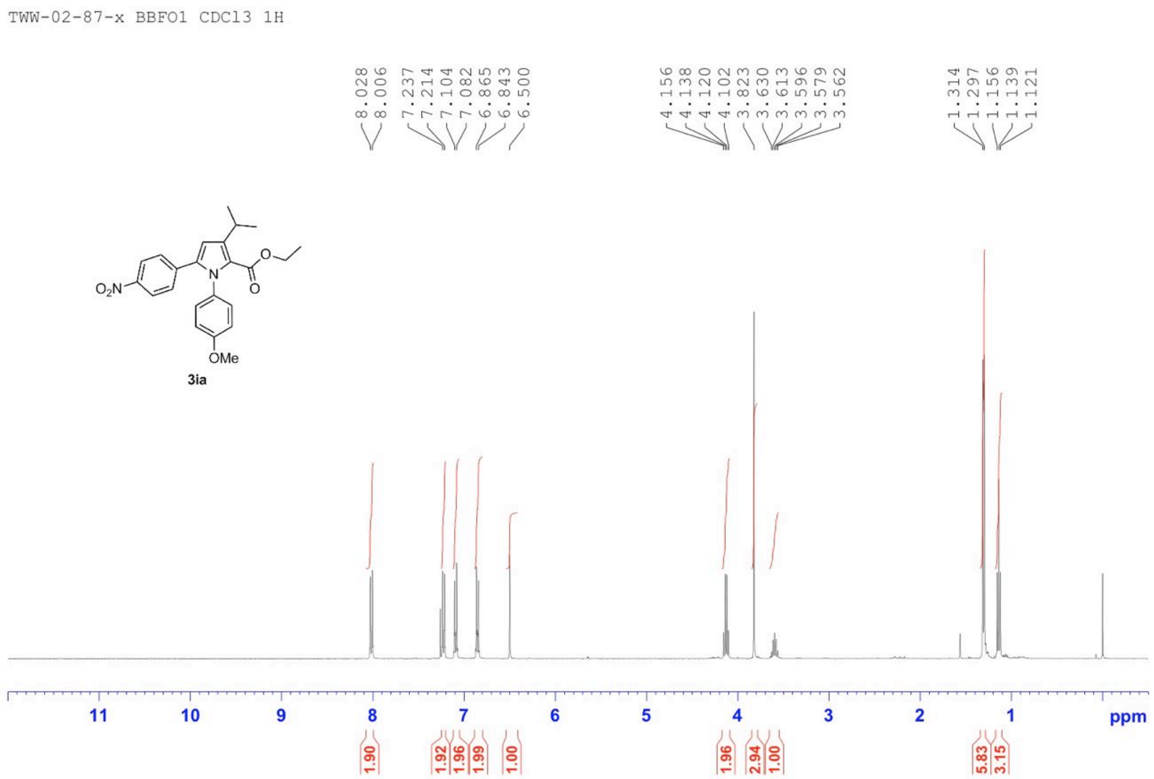
TWW-02-87-x BBFO1 CDCl3 1H
8.028
8.006
7.237
7.214
7.104
7.082
6.865
6.843
6.500
4.156
4.138
4.120
4.102
3.823
3.630
3.613
3.596
3.579
3.562
1.314
1.297
1.156
1.139
1.121
O2N
N
O
O
OMe
3ia
11 10 9 8 7 6 5 4 3 2 1 ppm
1.90
1.92
1.96
1.99
1.00
1.96
2.94
1.00
5.83
3.15

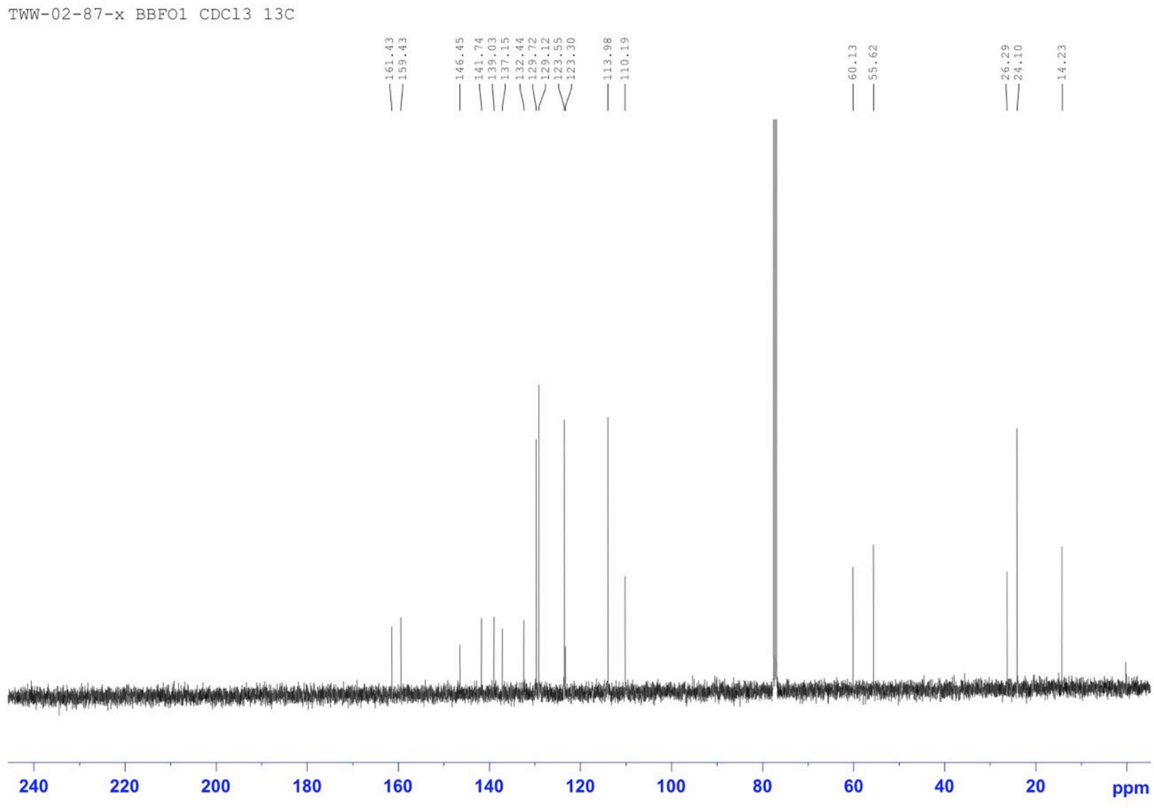
TWW-02-87-x BBFO1 CDCl3 13C
161.43
159.43
146.45
141.74
139.03
137.15
132.44
129.72
129.12
123.55
123.30
113.98
110.19
60.13
55.62
26.29
24.10
14.23
240 220 200 180 160 140 120 100 80 60 40 20 ppm

TWW-02-140 1H BBFO1 CDCl3

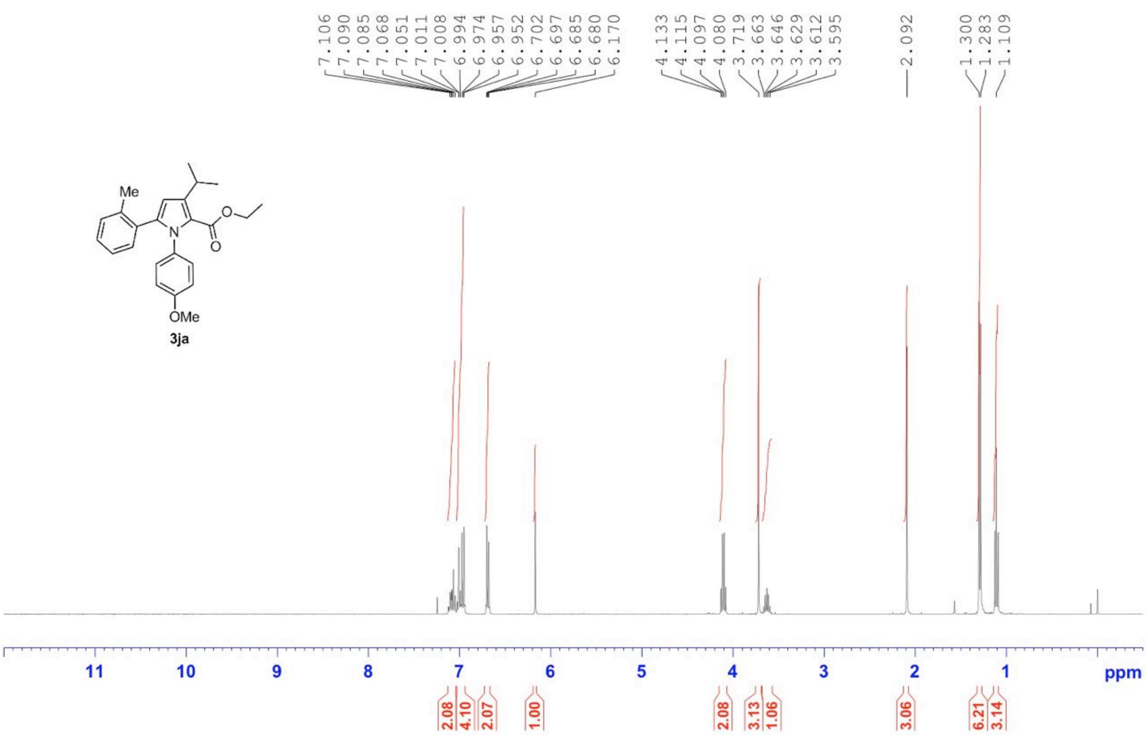

3ja

TWW-02-140 13C BBFO1 CDCl3

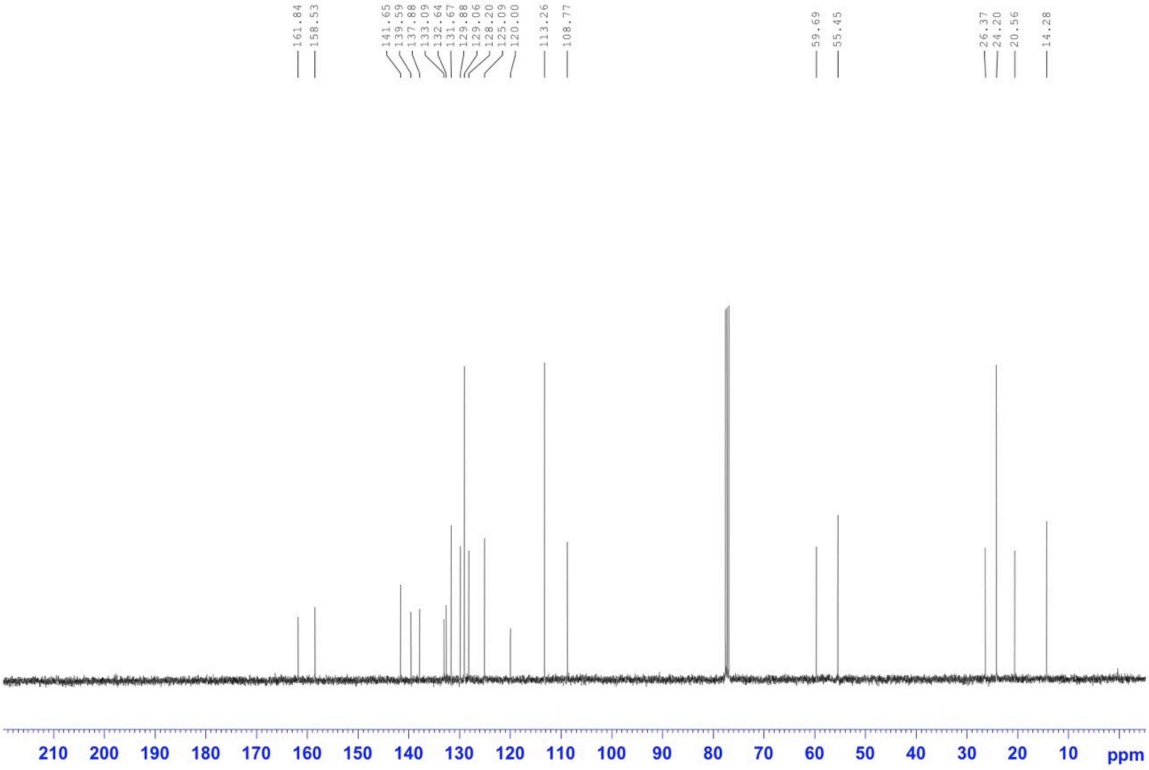

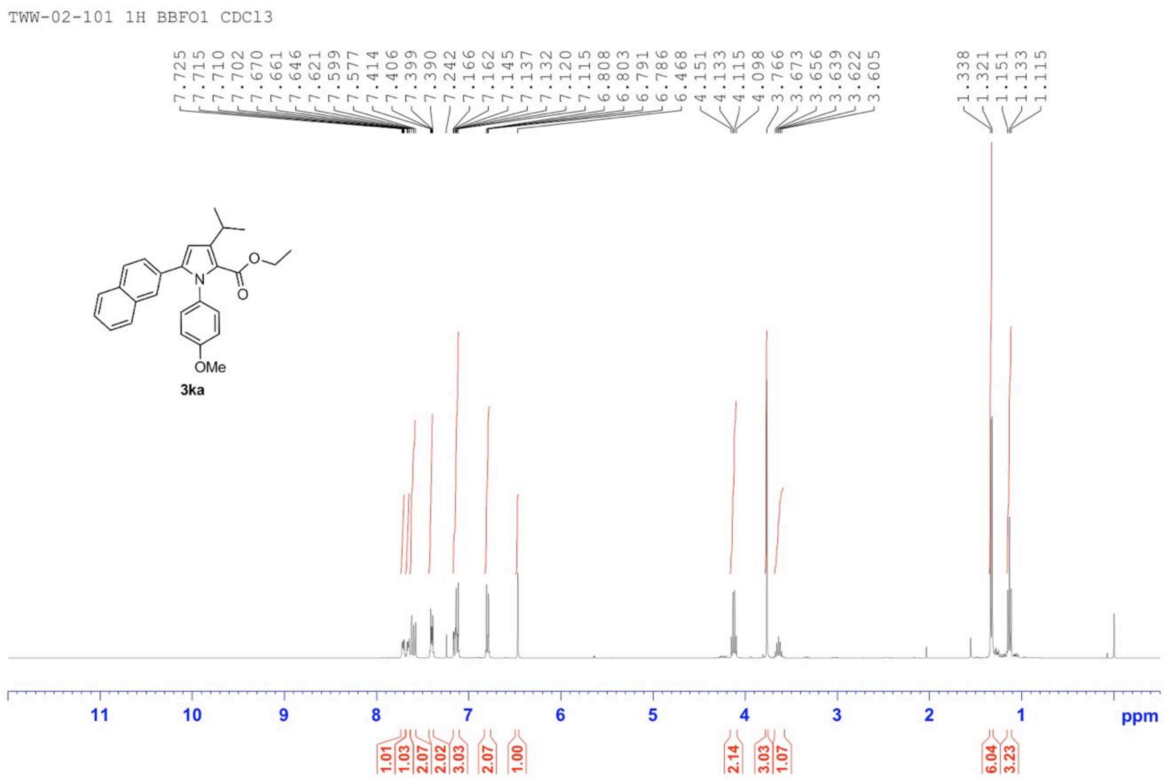
TWW-02-101 1H BBFO1 CDCl3
7.725 7.715 7.710 7.702 7.670 7.661 7.646 7.621 7.599 7.577 7.414 7.406 7.399 7.390 7.242 7.166 7.162 7.145 7.137 7.132 7.120 7.115 6.808 6.803 6.791 6.786 6.468 4.151 4.133 4.115 4.098 3.766 3.673 3.656 3.639 3.622 3.605 1.338 1.321 1.151 1.133 1.115
OMe
3ka
1.01 1.03 2.07 2.02 3.03 2.07 1.00 2.14 3.03 1.07 6.04 3.23
ppm

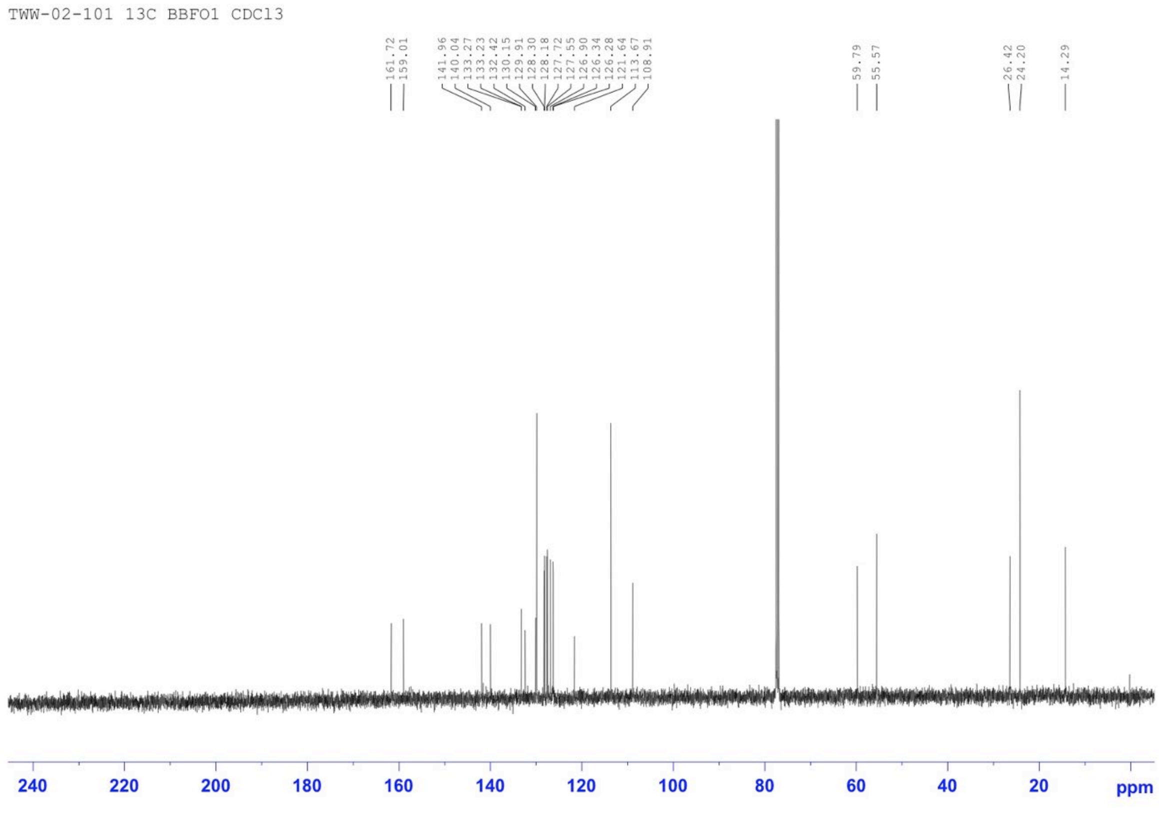
TWW-02-101 13C BBFO1 CDCl3
161.72 159.01 141.96 140.04 133.27 133.23 132.42 130.15 129.91 128.30 128.18 127.72 127.55 126.90 126.34 126.28 121.64 113.67 108.91 59.79 55.57 26.42 24.20 14.29
ppm

TWW-02-137 1H BBFO1 CDCl3

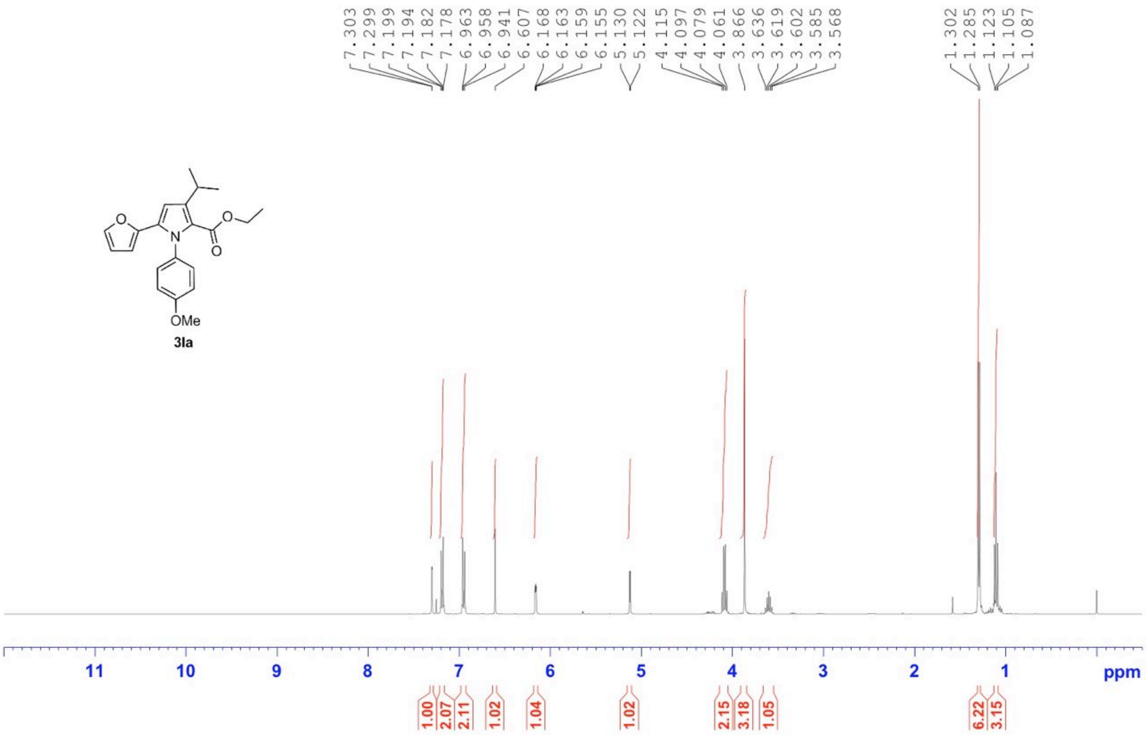

TWW-02-137 13C BBFO1 CDCl3

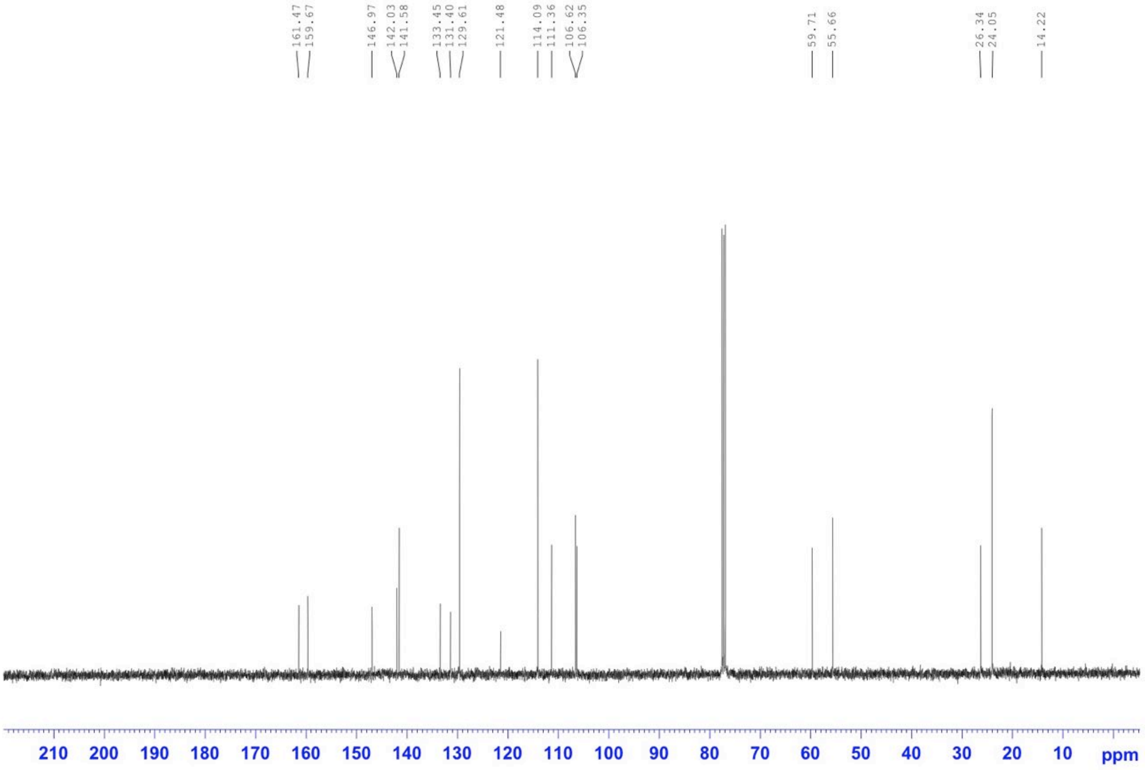

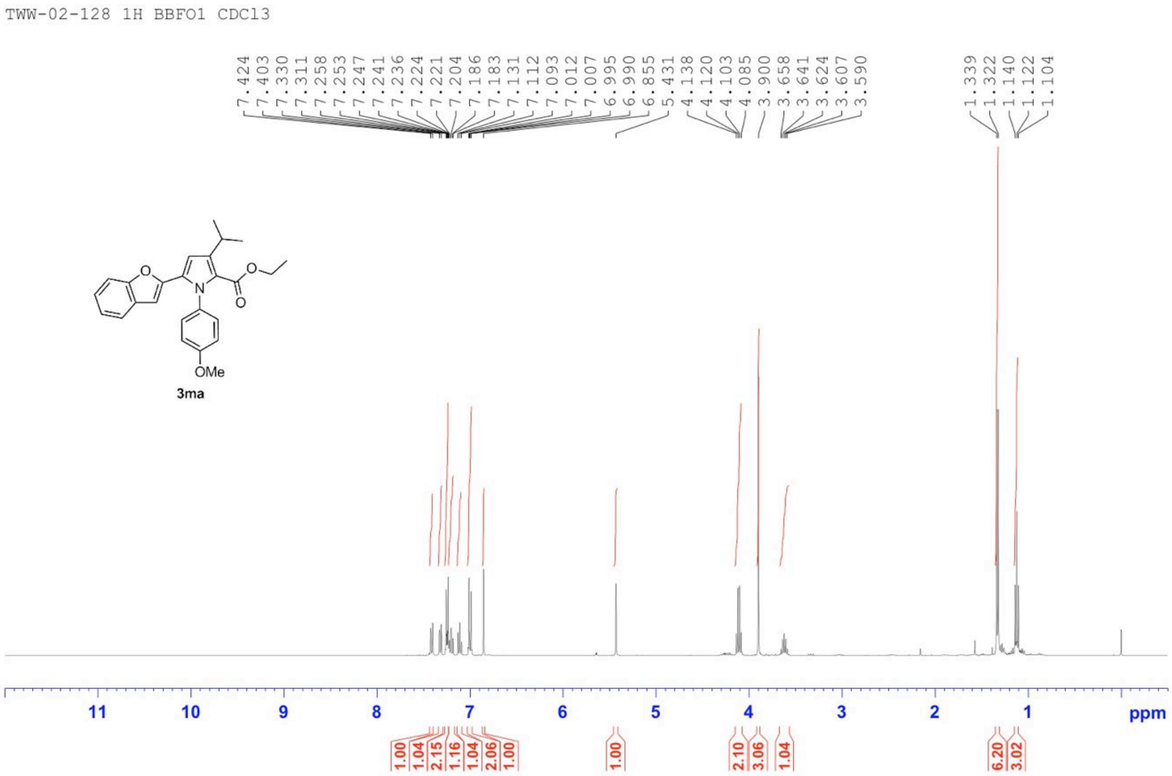
TWW-02-128 1H BBFO1 CDCl3
7.424
7.403
7.330
7.311
7.258
7.253
7.247
7.241
7.236
7.224
7.221
7.204
7.186
7.183
7.131
7.112
7.093
7.012
7.007
6.995
6.990
6.855
5.431
4.138
4.120
4.103
4.085
3.900
3.658
3.641
3.624
3.607
3.590
1.339
1.322
1.140
1.122
1.104
OMe
3ma
11 10 9 8 7 6 5 4 3 2 1 ppm
1.00 1.04 2.15 1.16 1.04 2.06 1.00
1.00
2.10 3.06 1.04
6.20 3.02

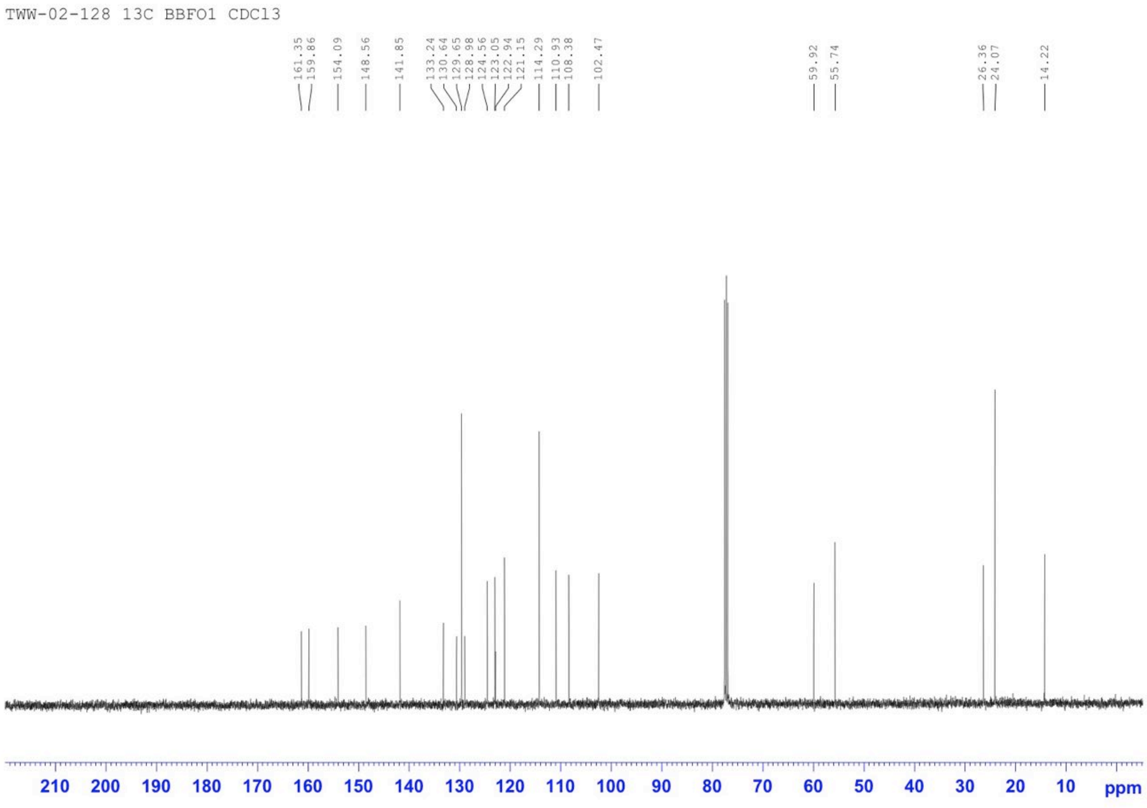
TWW-02-128 13C BBFO1 CDCl3
161.35
159.86
154.09
148.56
141.85
133.24
130.64
129.65
128.98
124.56
123.05
122.94
121.15
114.29
110.93
108.38
102.47
59.92
55.74
26.36
24.07
14.22
210 200 190 180 170 160 150 140 130 120 110 100 90 80 70 60 50 40 30 20 10 ppm

TWW-02-095, BBFO, 1H, CDCl3

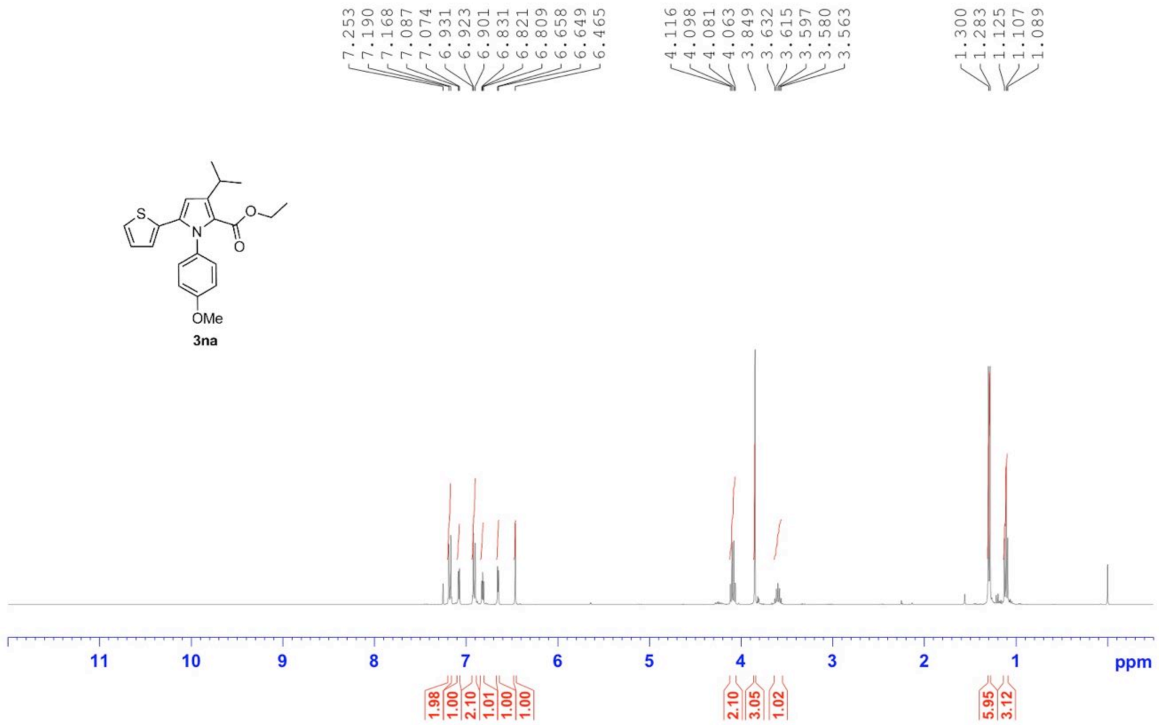

TWW-02-095, BBFO, 13C, CDCl3

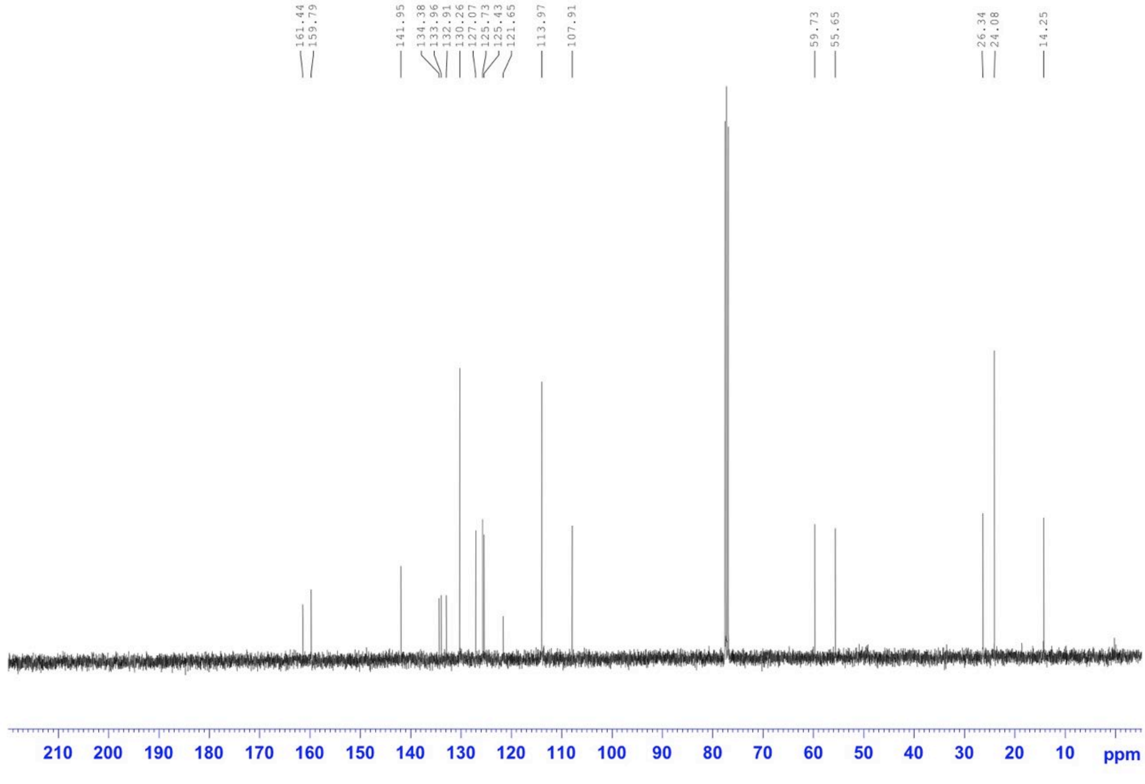

TWW-02-218, 1H, BBFO1, CDCl3

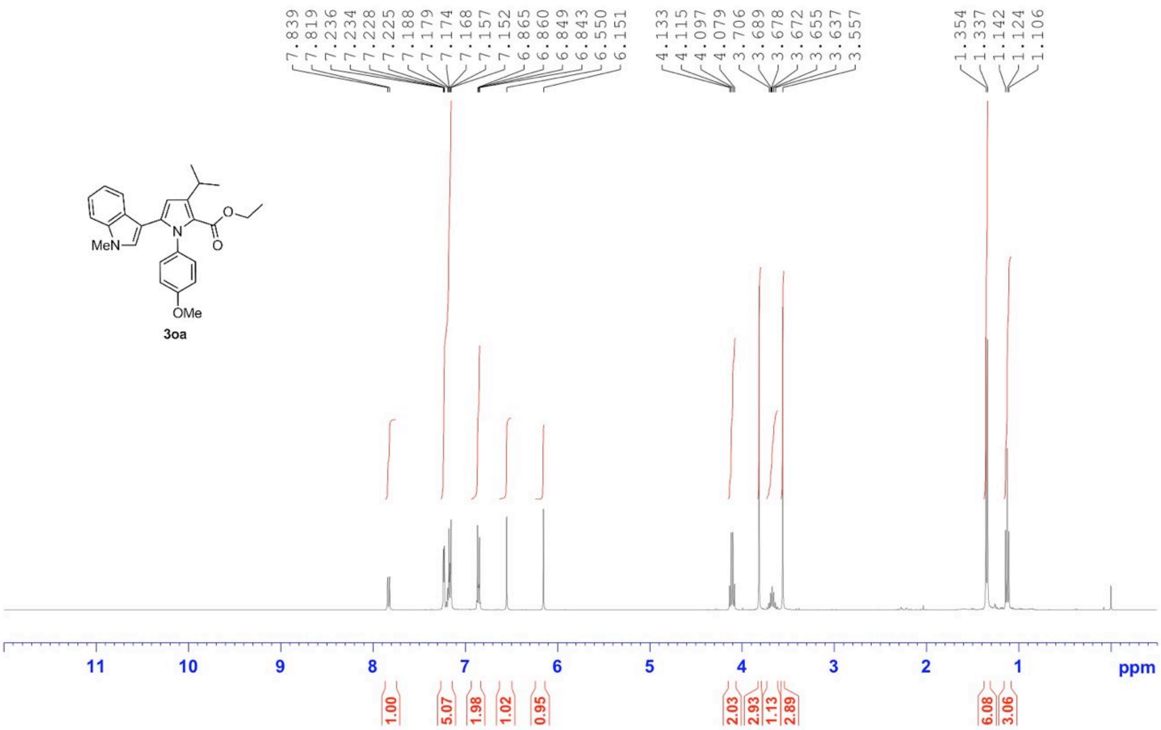

TWW-02-218, 13C, BBFO1, CDCl3

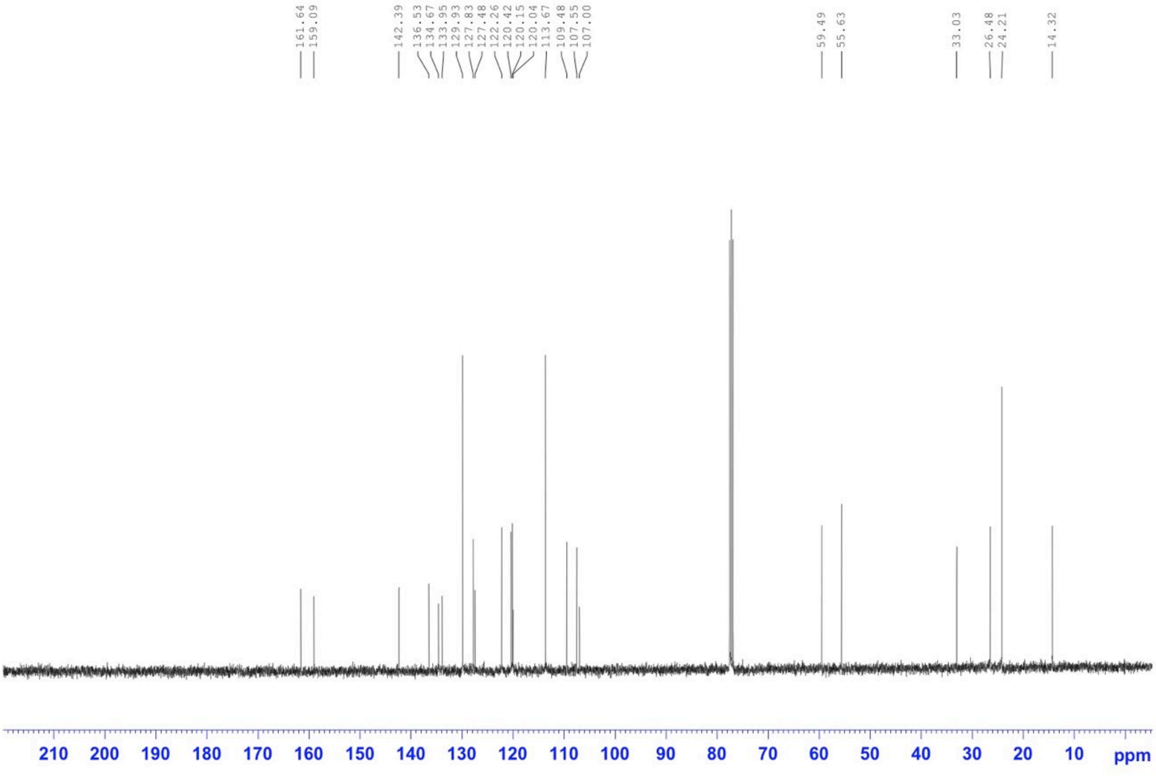

TWW-02-091, BBFO, 1H, CDCl3

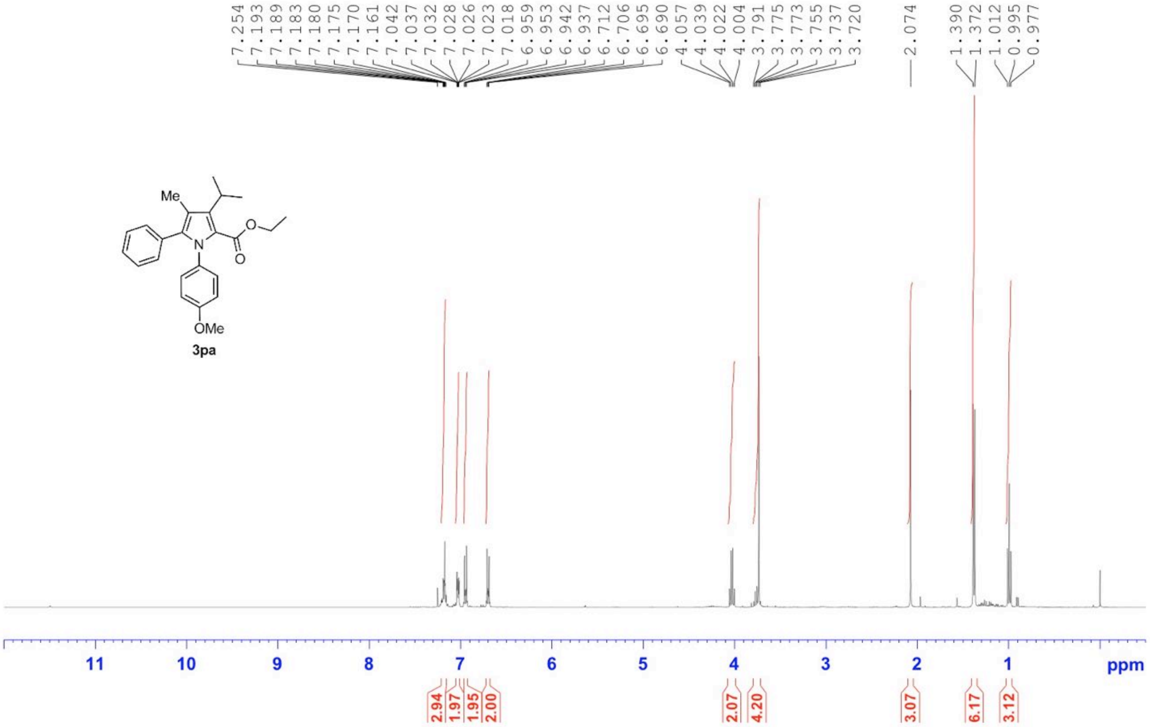

TWW-02-091, BBFO, 13C, CDCl3

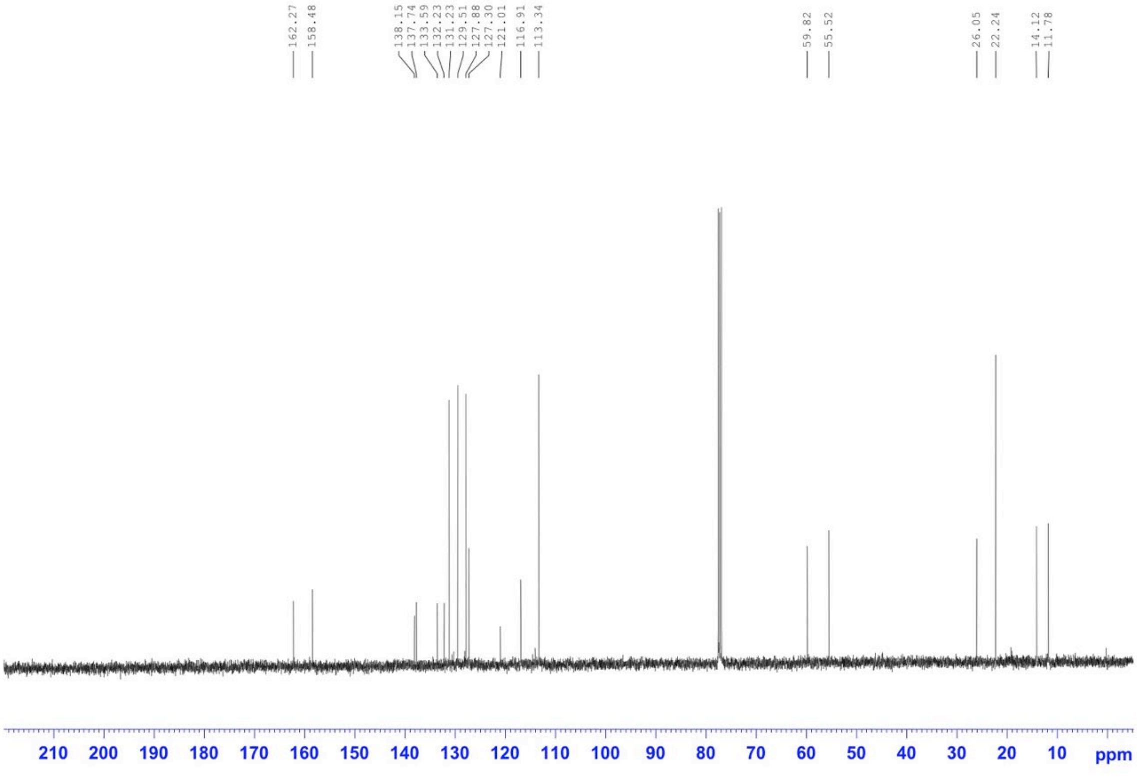

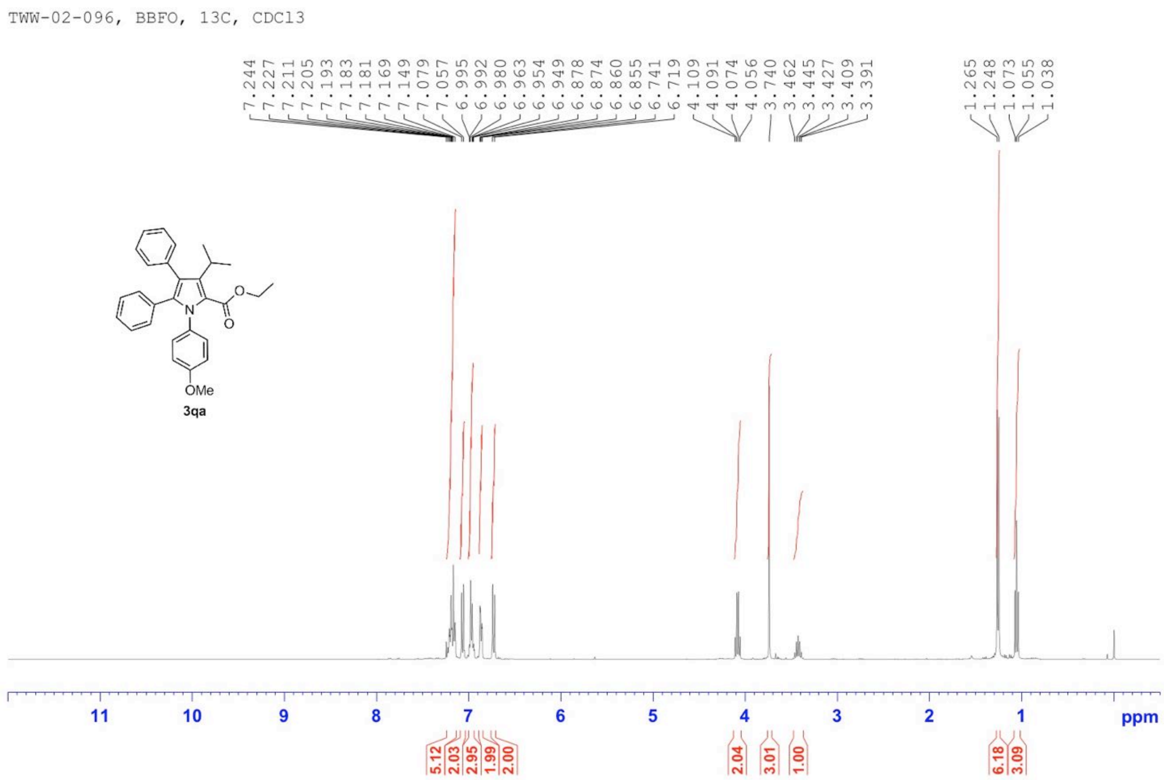
TWW-02-096, BBFO, 13C, CDCl3
7.244 7.227 7.211 7.205 7.193 7.183 7.181 7.169 7.149 7.079 7.057 6.995 6.992 6.980 6.963 6.954 6.949 6.878 6.874 6.860 6.855 6.741 6.719 4.109 4.091 4.074 4.056 3.740 3.462 3.445 3.427 3.409 3.391 1.265 1.248 1.073 1.055 1.038
OMe
N
O
3qa
5.12 2.03 2.95 1.99 2.00 2.04 3.01 1.00 6.18 3.09
11 10 9 8 7 6 5 4 3 2 1 ppm

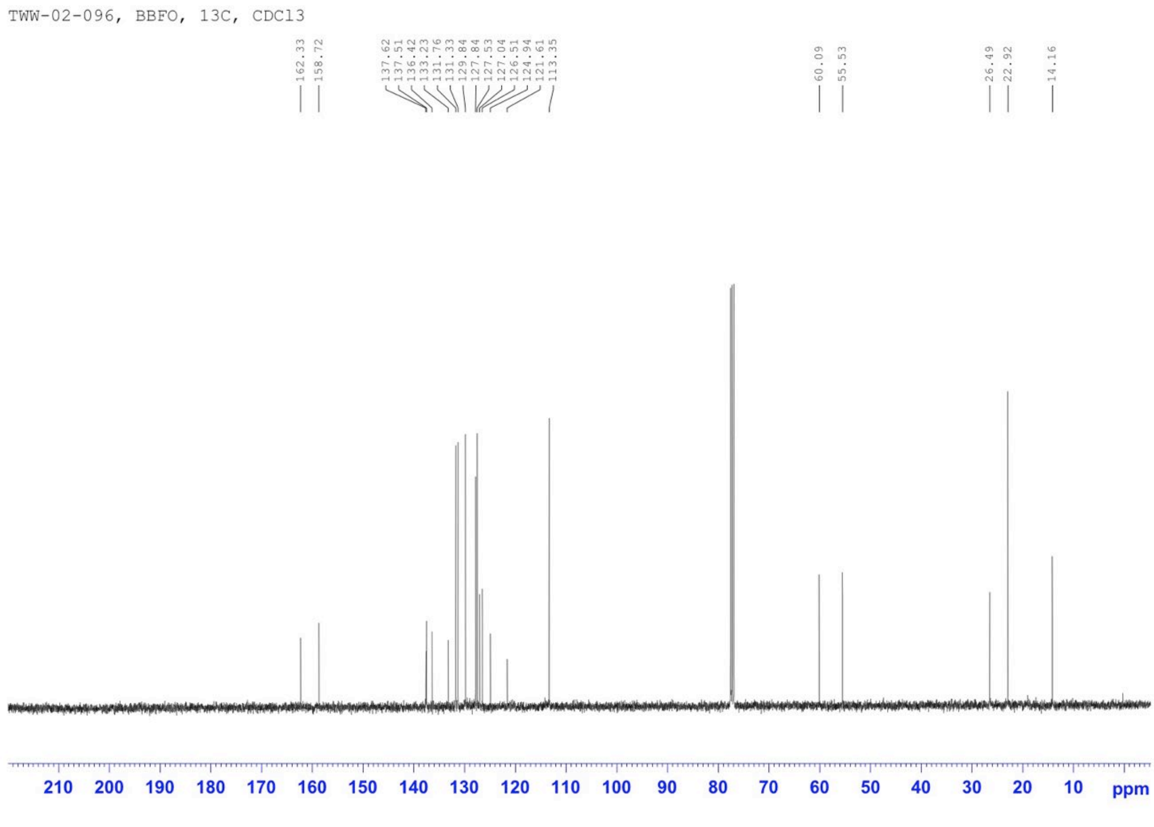
TWW-02-096, BBFO, 13C, CDCl3
162.33 158.72 137.62 137.51 136.42 133.23 131.76 131.33 129.84 127.84 127.53 127.04 126.51 124.94 121.61 113.35 60.09 55.53 26.49 22.92 14.16
210 200 190 180 170 160 150 140 130 120 110 100 90 80 70 60 50 40 30 20 10 ppm

TWW-02-240, 1H, BBFO1, CDCl3

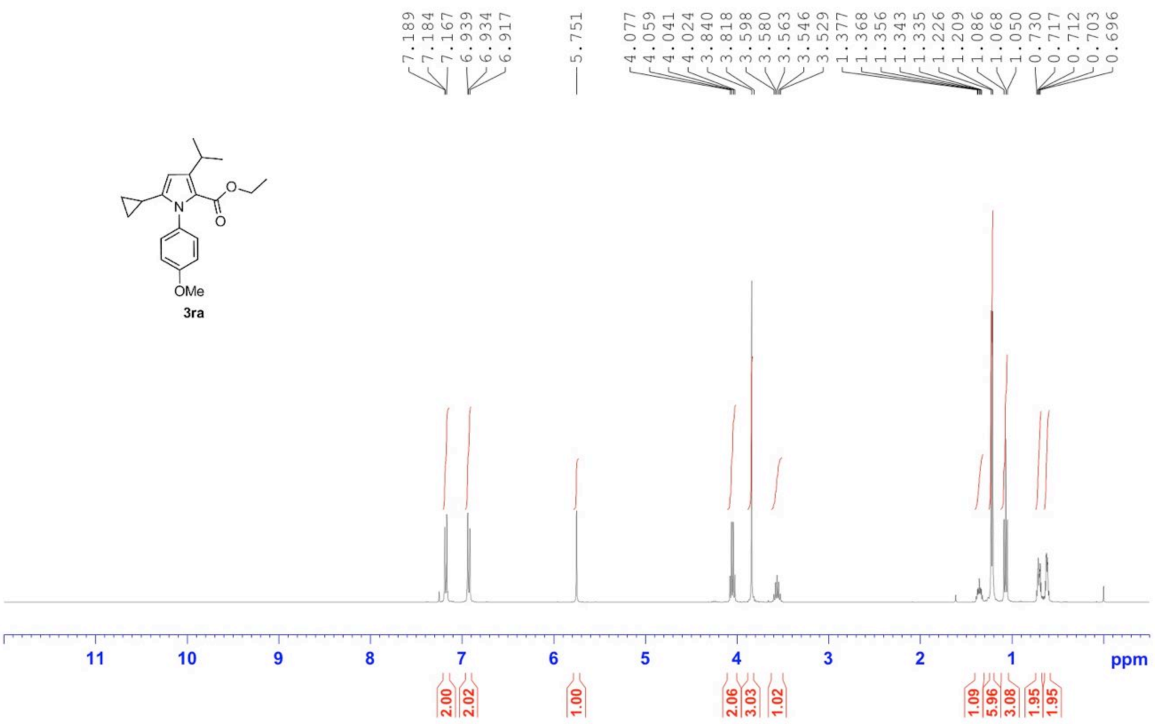

TWW-02-240, 13C, BBFO1, CDCl3

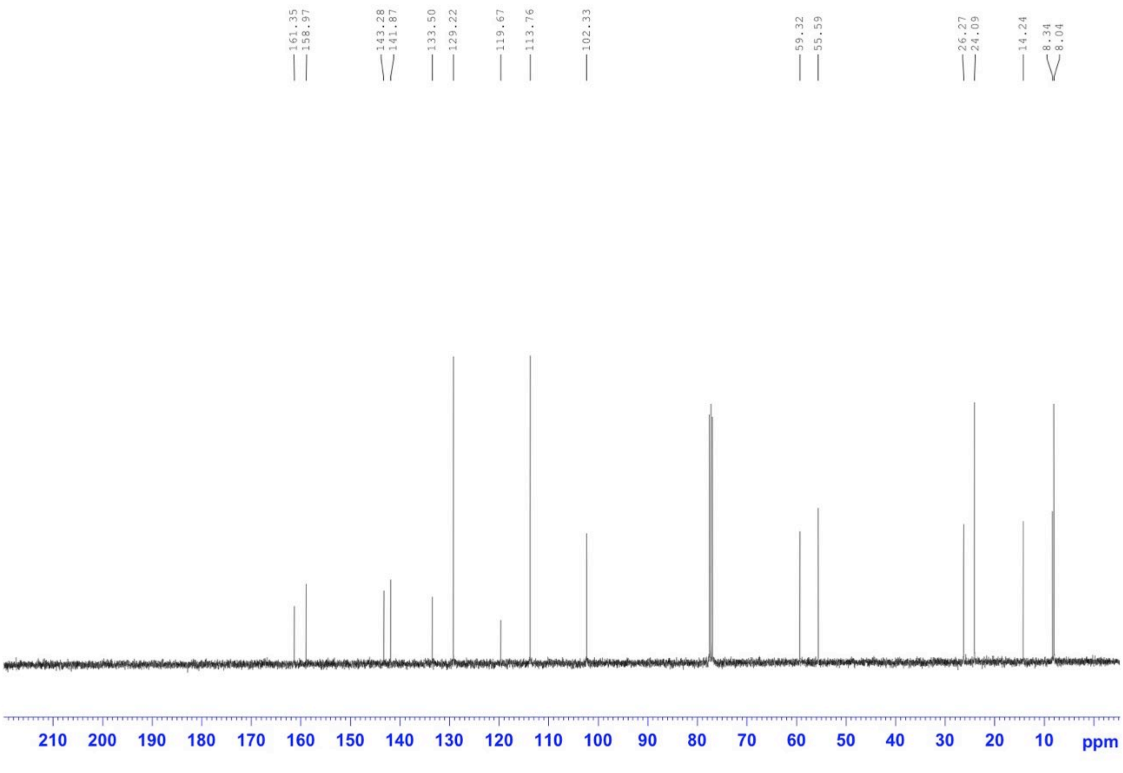

TWW-02-245, 1H, BBFO1, CDCl3

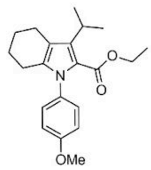

3sa

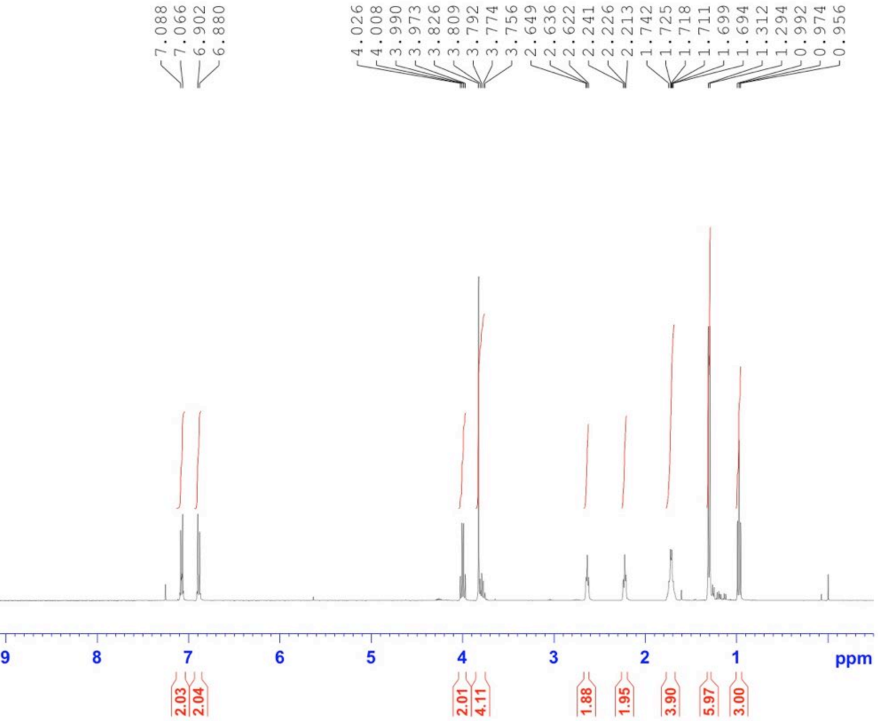

TWW-02-245, 13C, BBFO1, CDCl3

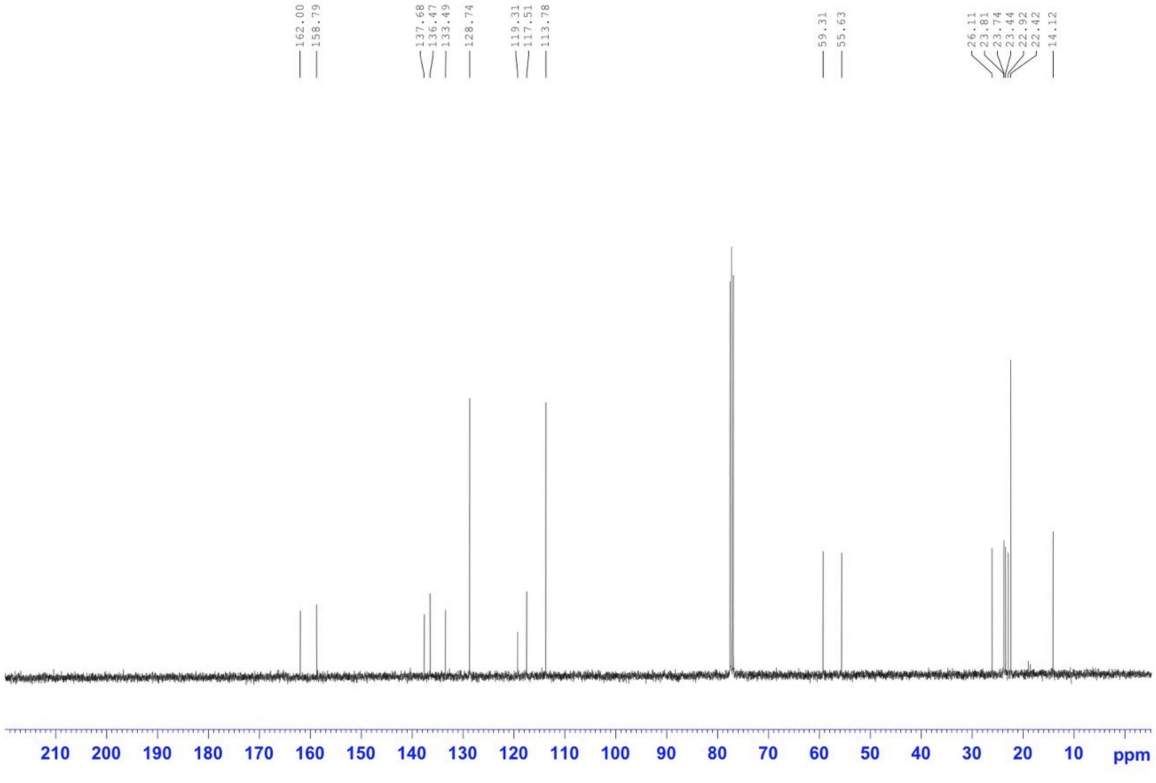

TWW-02-238, 1H, BBFO1, CDCl3

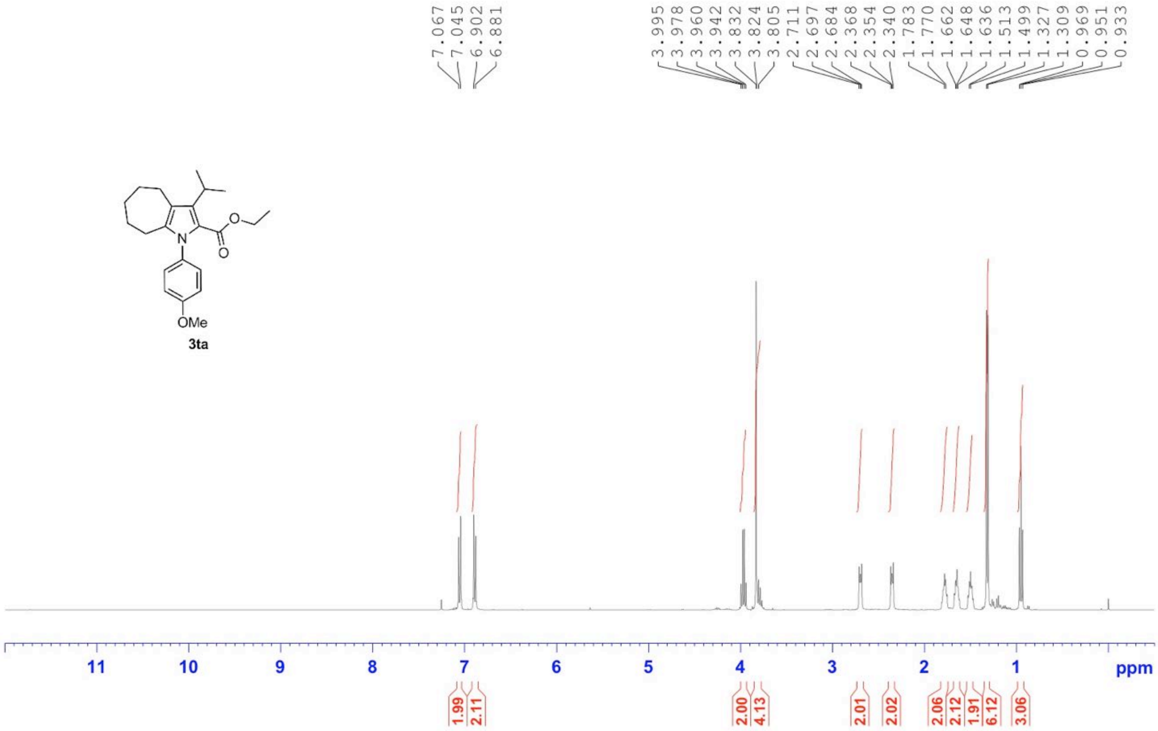

3ta

TWW-02-238, 13C, BBFO1, CDCl3

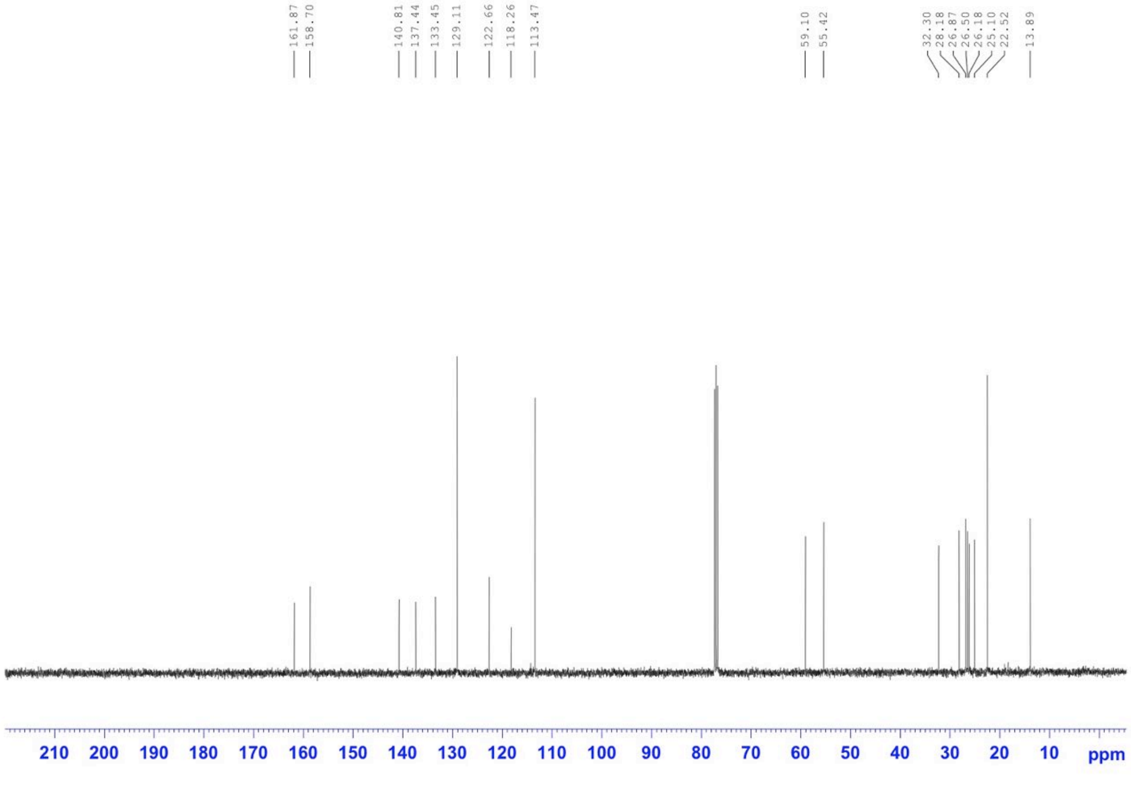

TWW-02-236-1, 1H NMR, 400M Hz, BBFO2

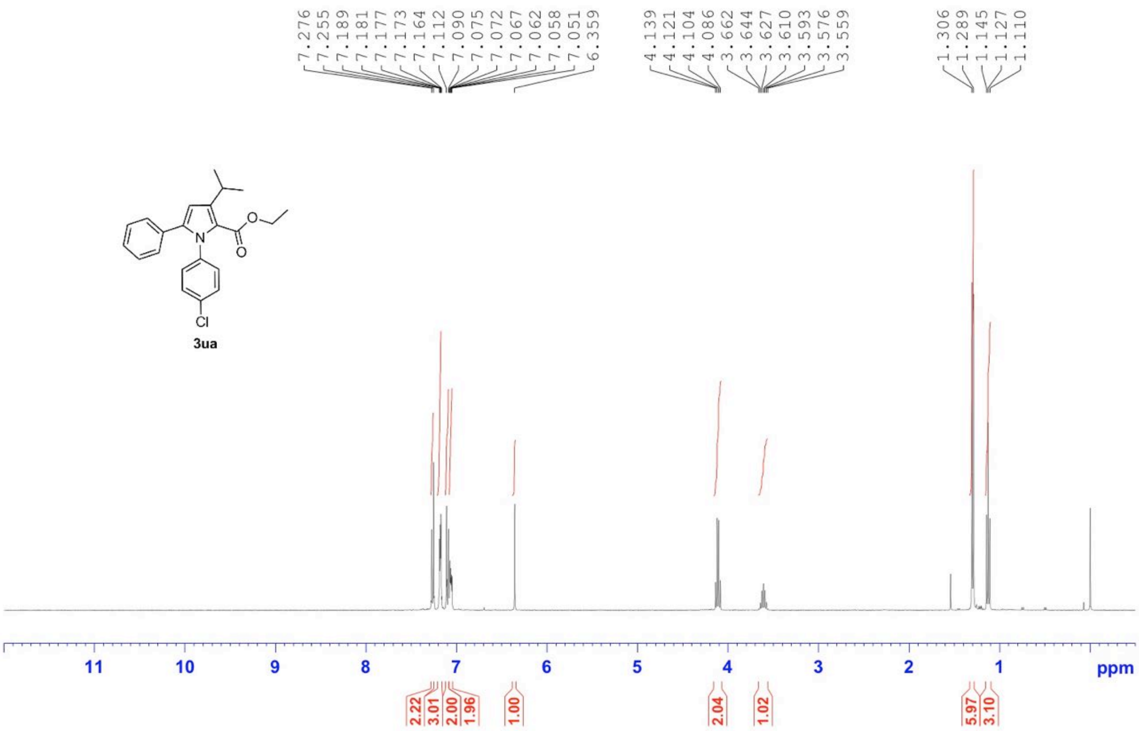

TWW-02-236-1, 13C NMR, 400M Hz, BBFO2

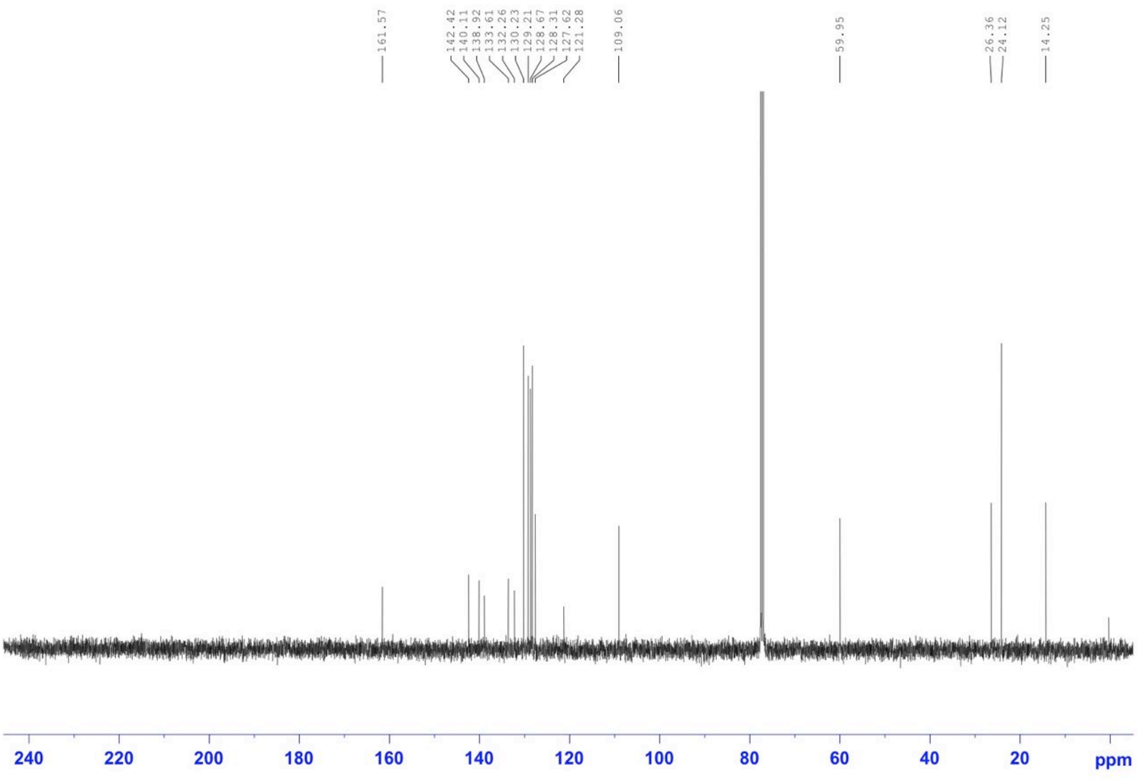

TWW-02-083, BBFO, 1H, CDCl3

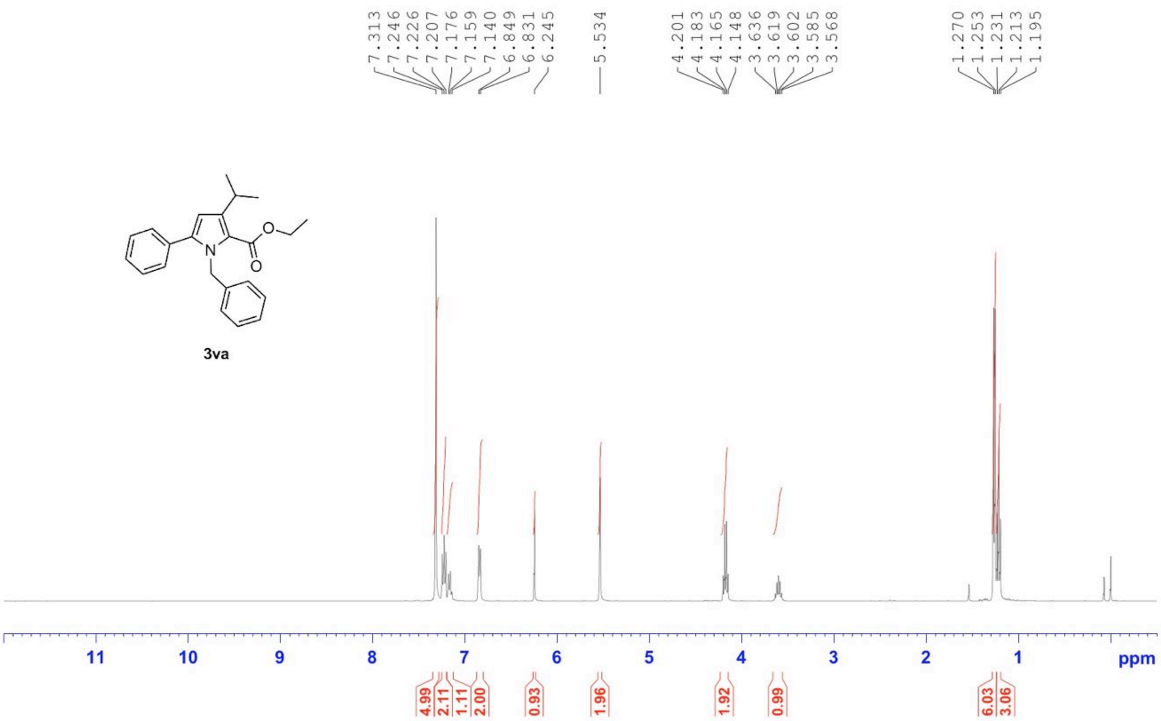

TWW-02-083, BBFO, 13C, CDCl3

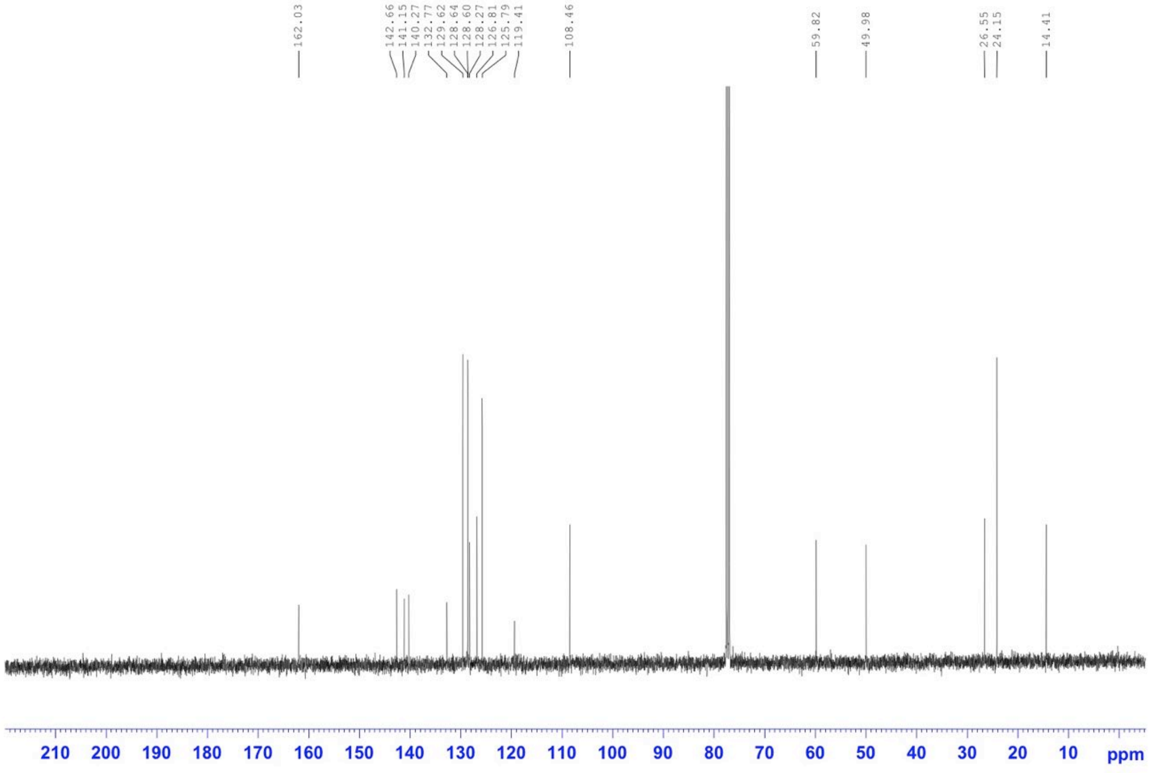

TWW-02-147 1H BBFO1 CDCl3

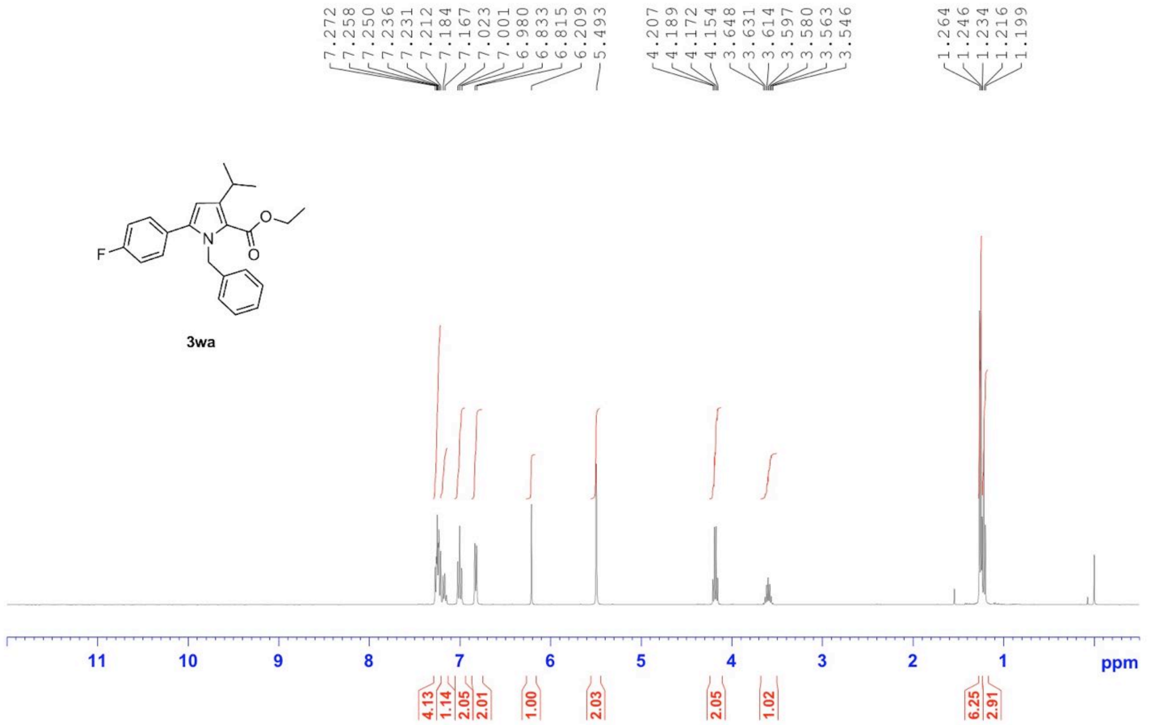

3wa

TWW-02-147 13C BBFO1 CDCl3

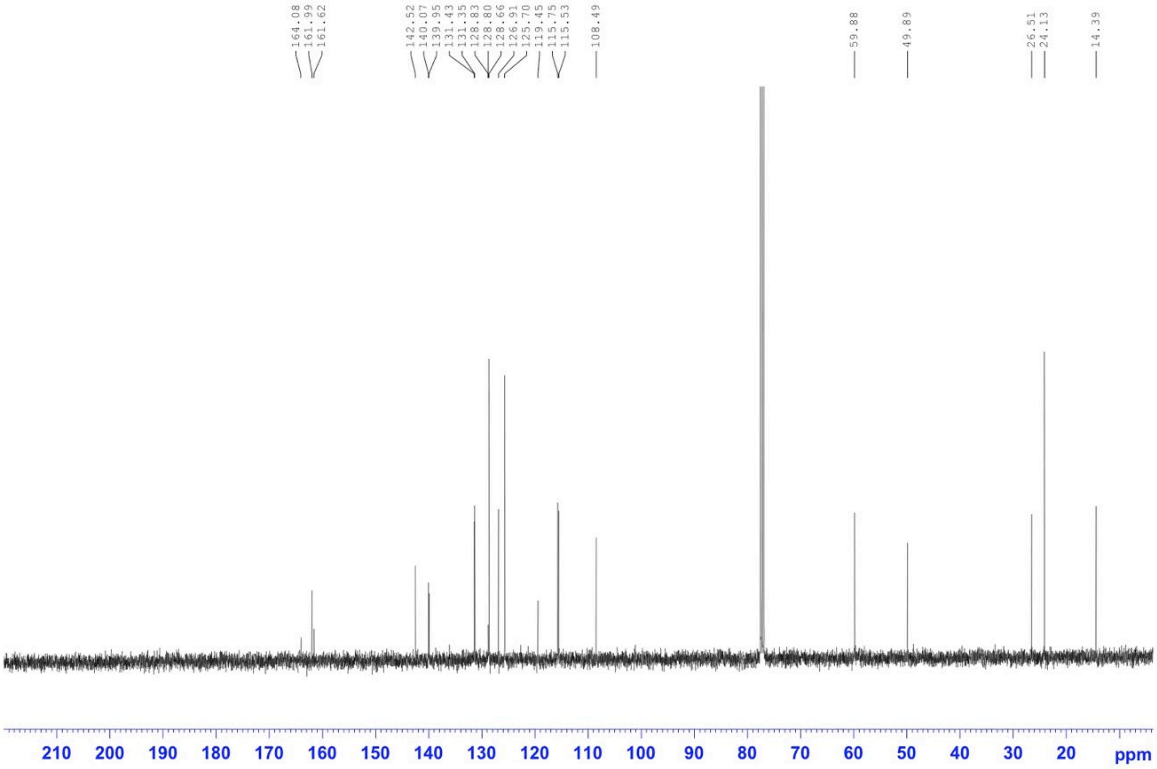

TWW-02-146 1H BBFO1 CDCl3

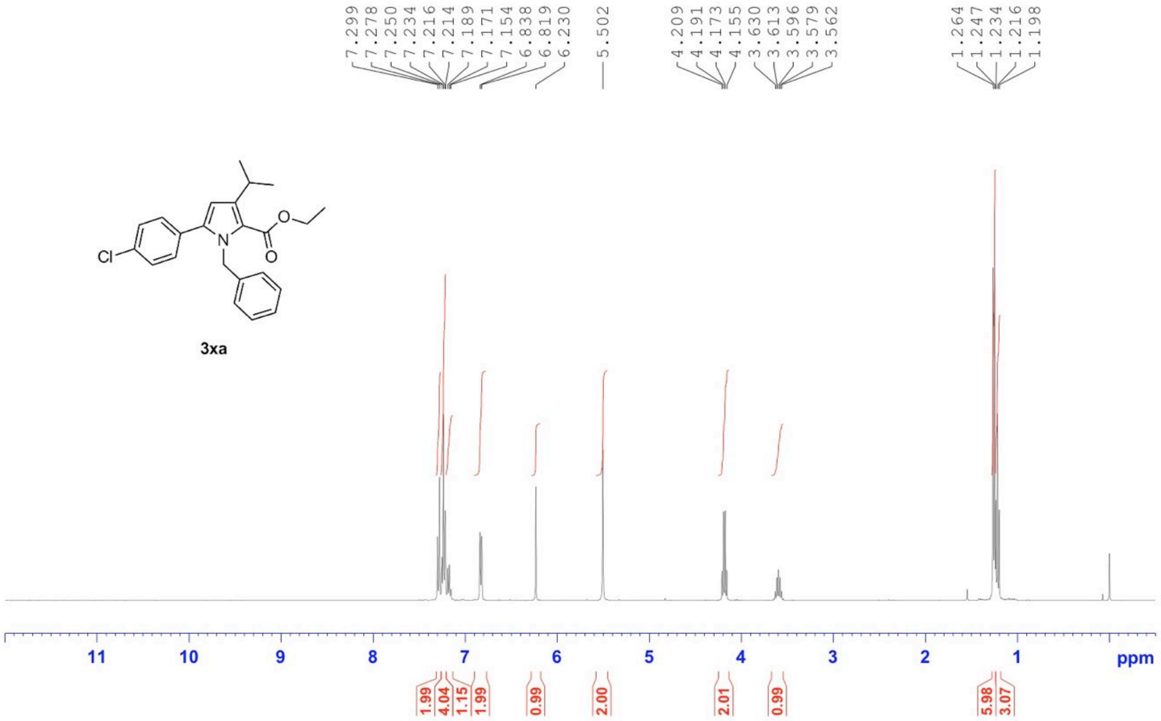

TWW-02-146 13C BBFO1 CDCl3

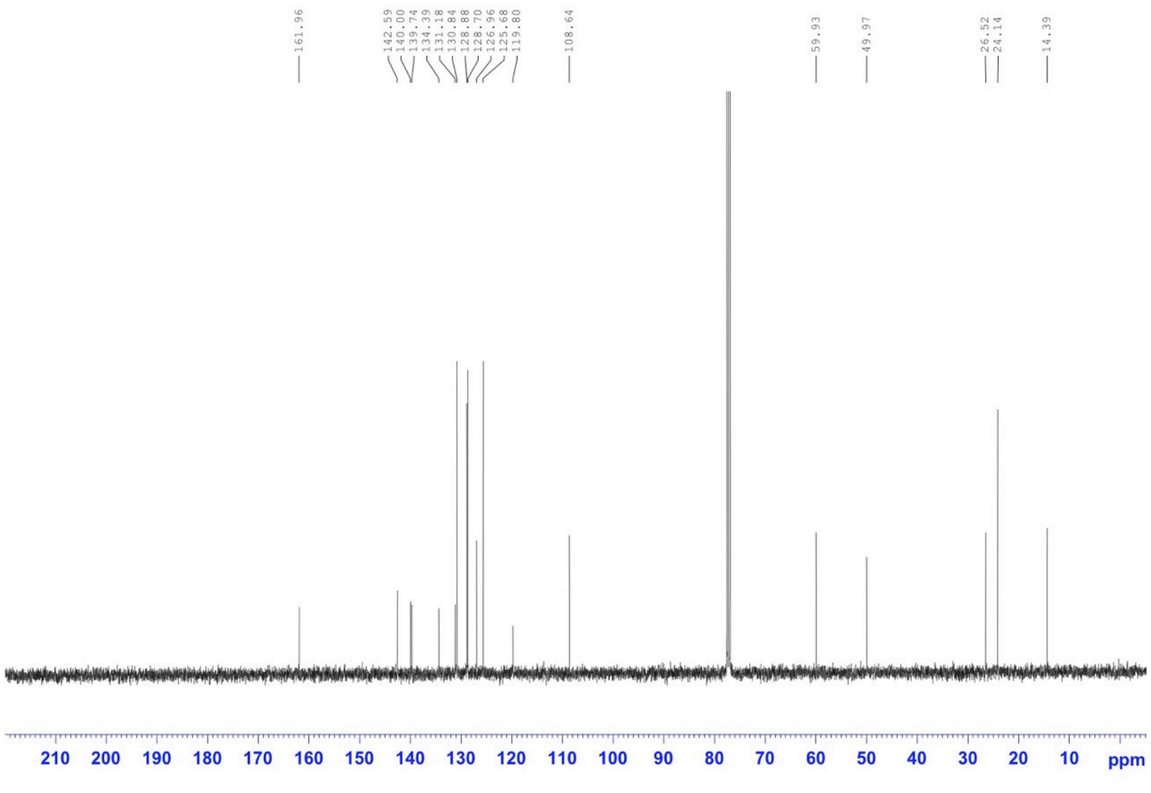

TWW-02-129 1H BBFO1 CDCl3

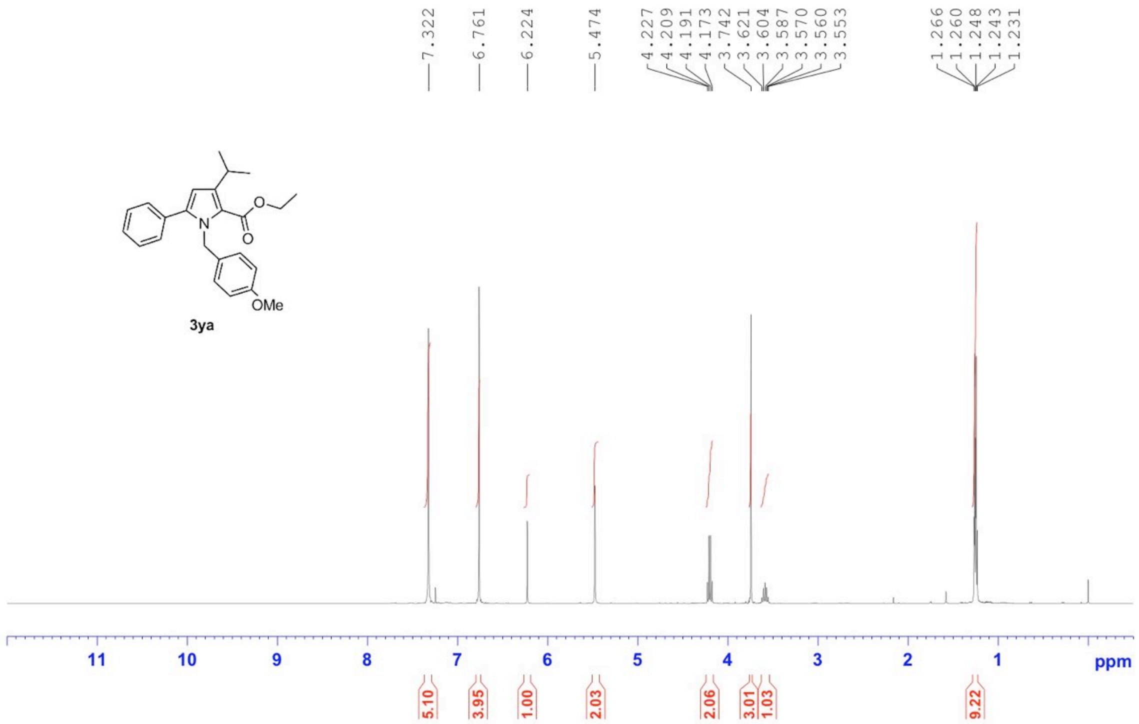

TWW-02-129 13C BBFO1 CDCl3

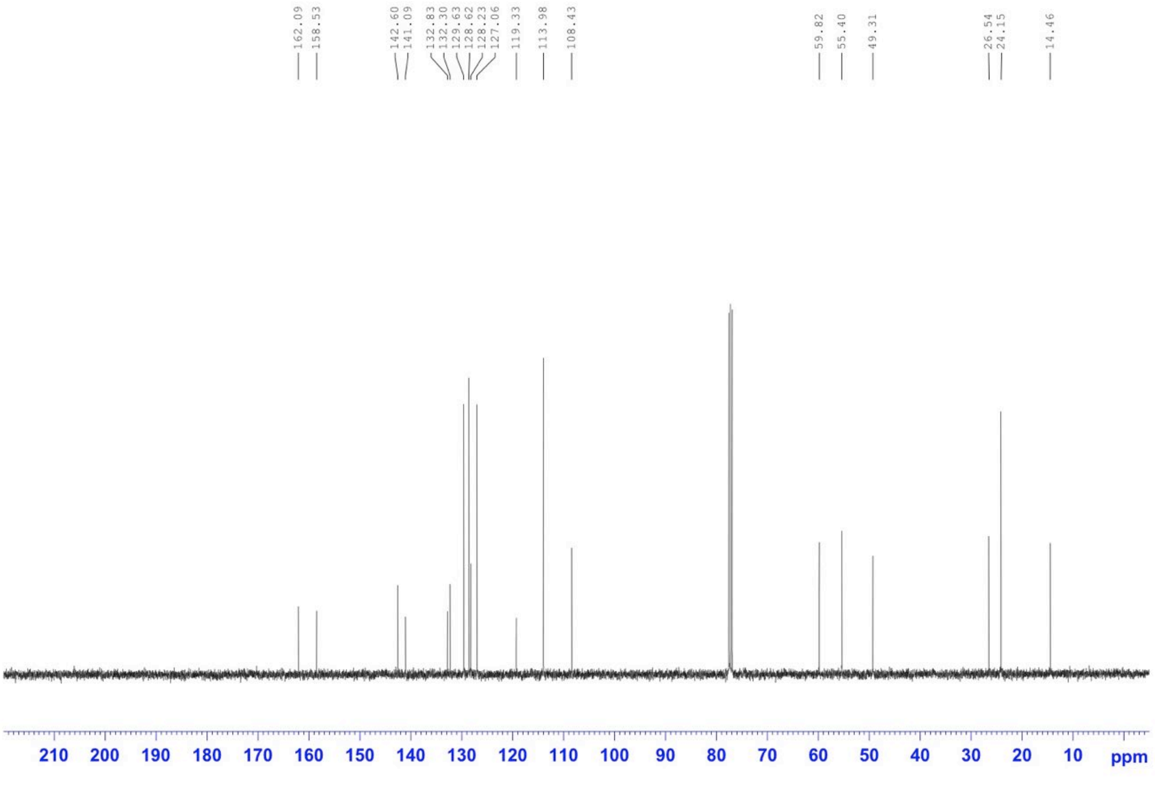

TWW-02-402-acetone, 1H, BBFO1, Acetone

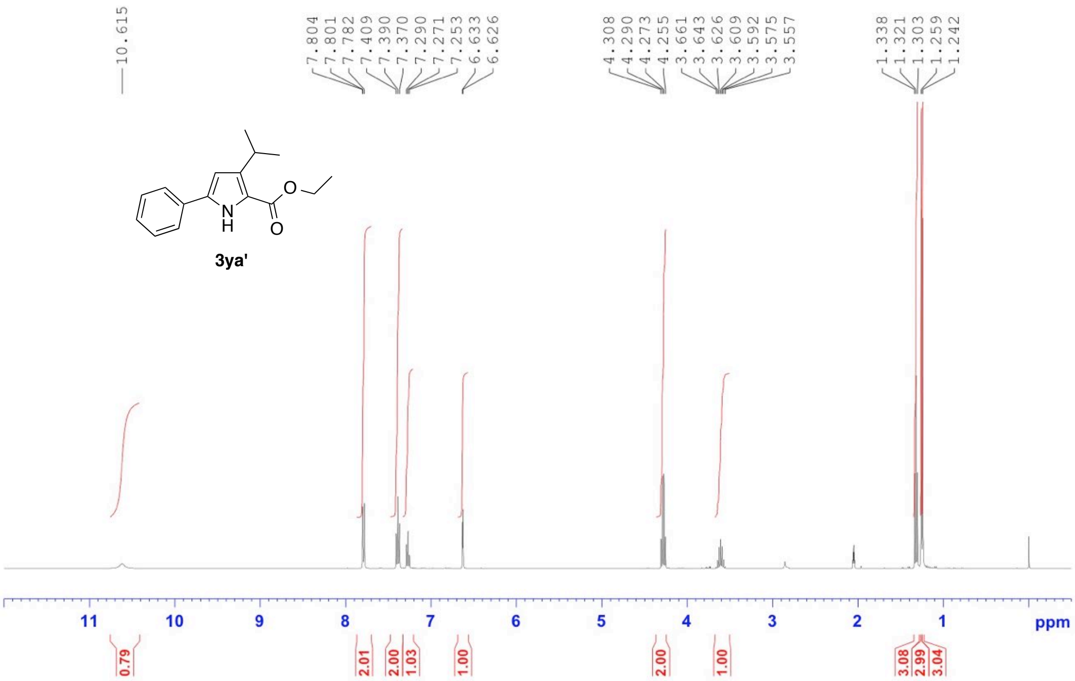

TWW-02-402-acetone, 13C, BBFO1, Acetone

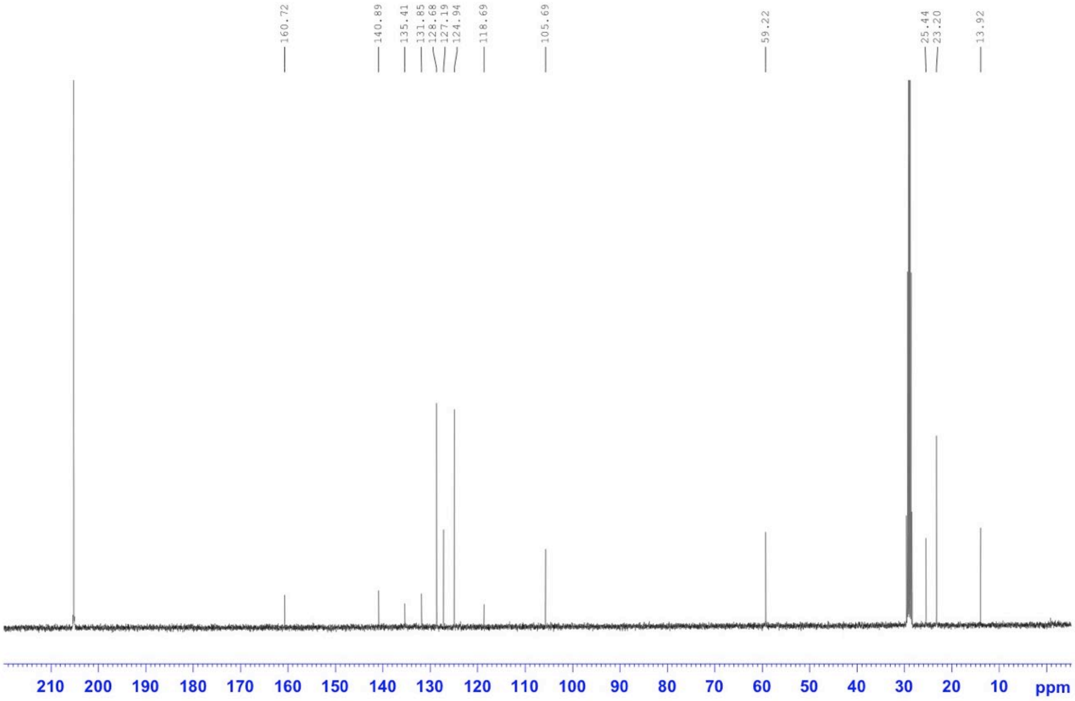

TWW-02-085, BBFO, 1H, CDCl3

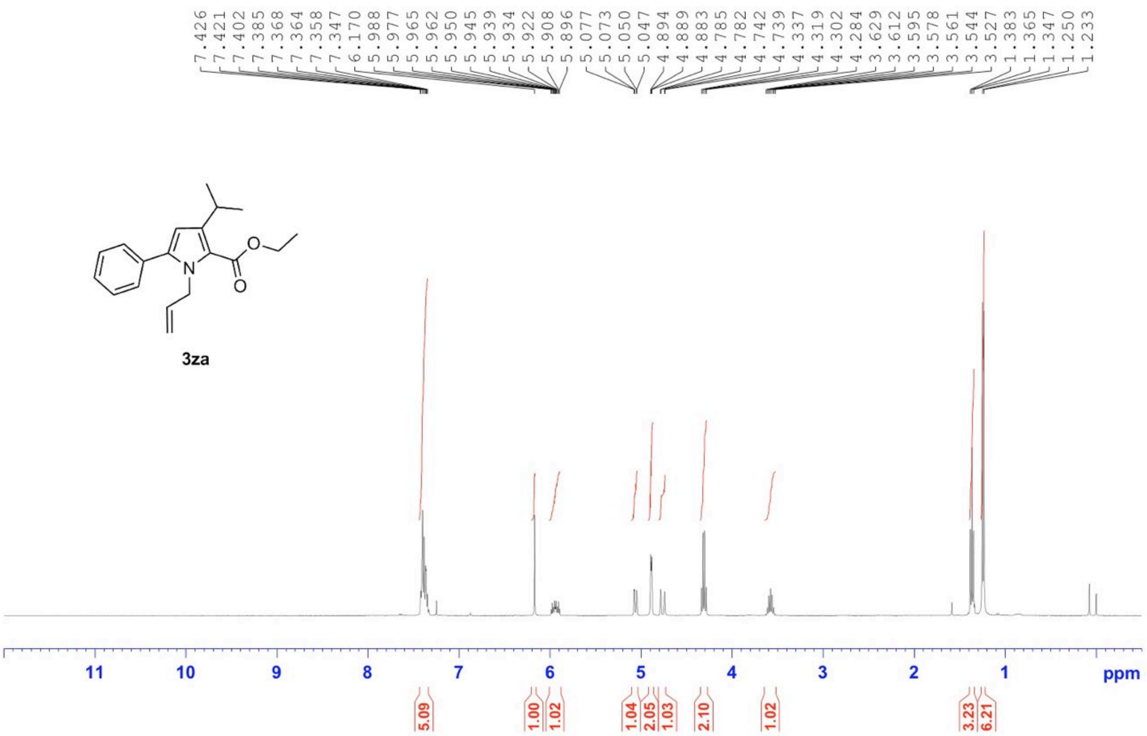

TWW-02-085, BBFO, 13C, CDCl3

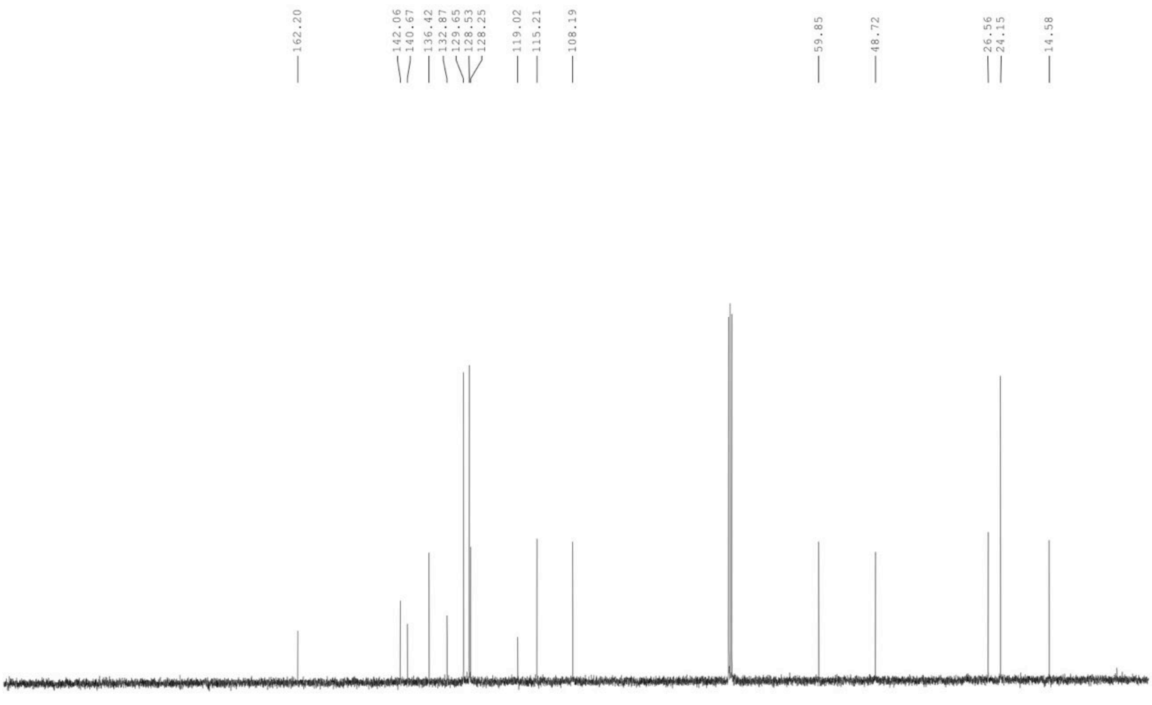

TWW-02-295, 1H, BBFO1, CDCl3

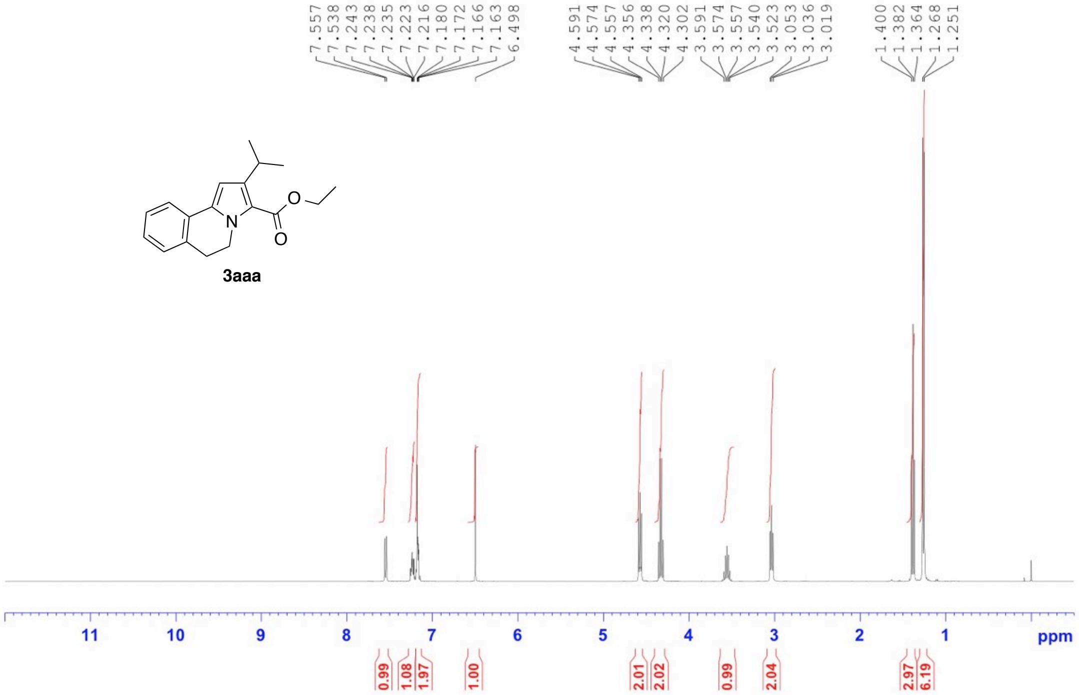

TWW-02-295, 13C, BBFO1, CDCl3

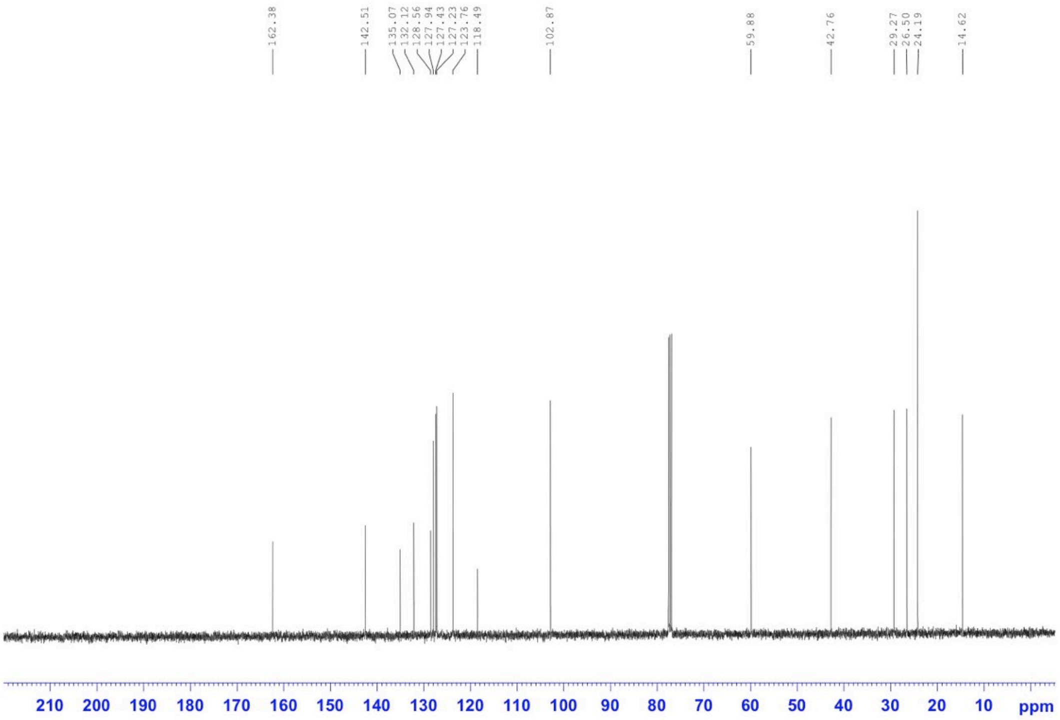

TWW-02-262-1, 1H, BBFO1, CDCl3

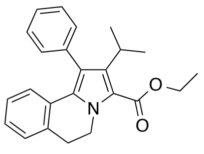

3aba

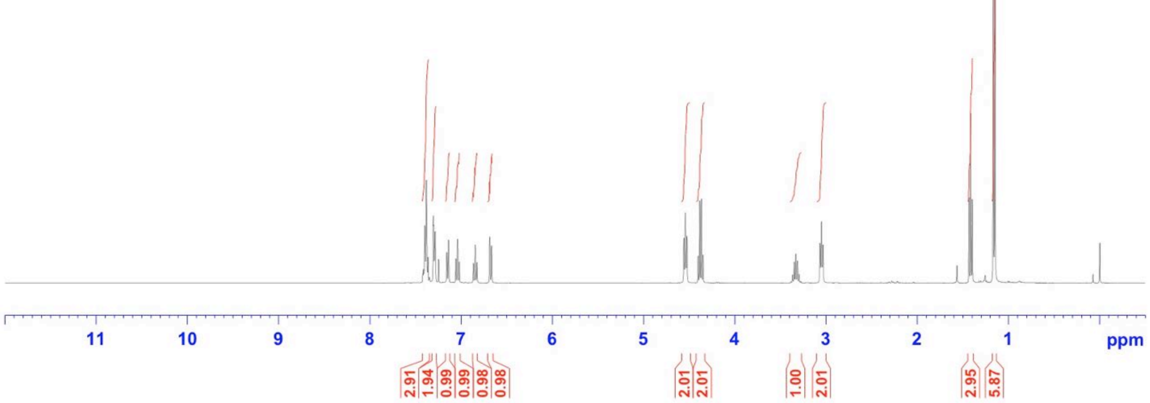

TWW-02-262-1, 13C, BBFO1, CDCl3

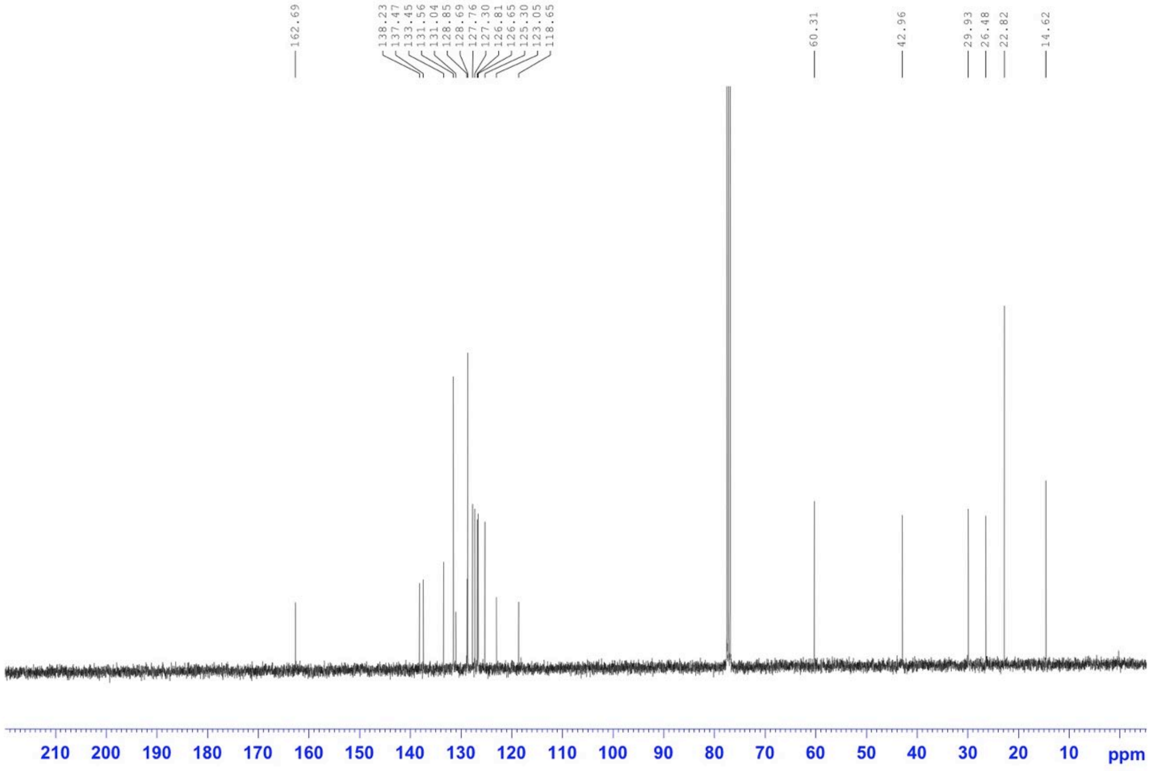

TWW-02-262-2 1H, BBFO1, CDCl3

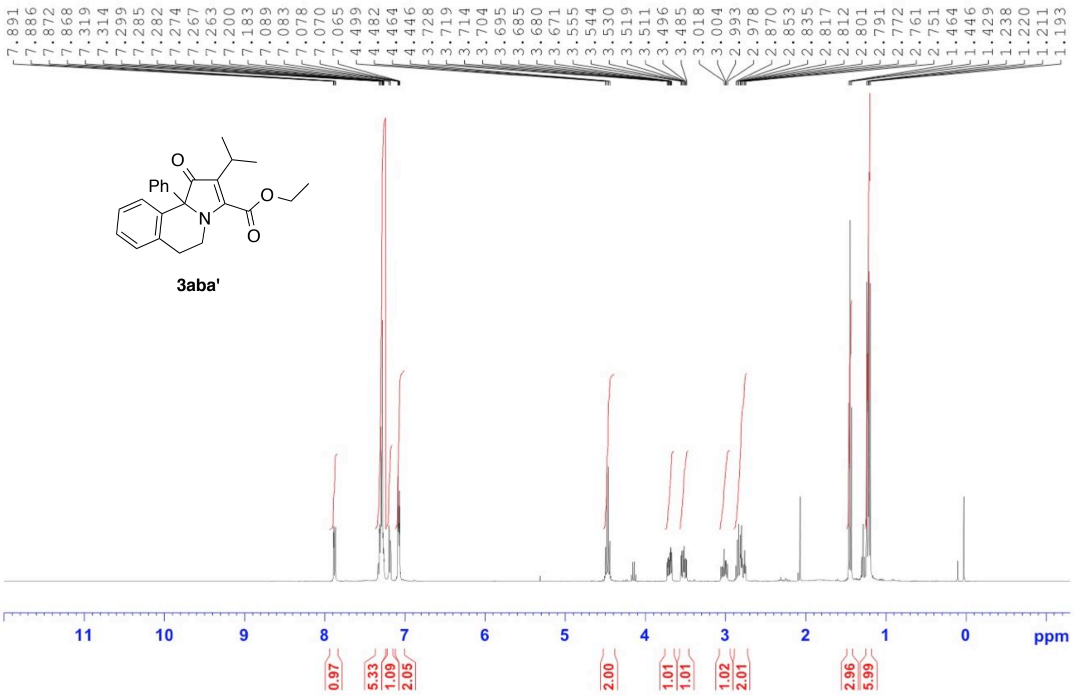

TWW-02-262-2 13C, BBFO1, CDCl3

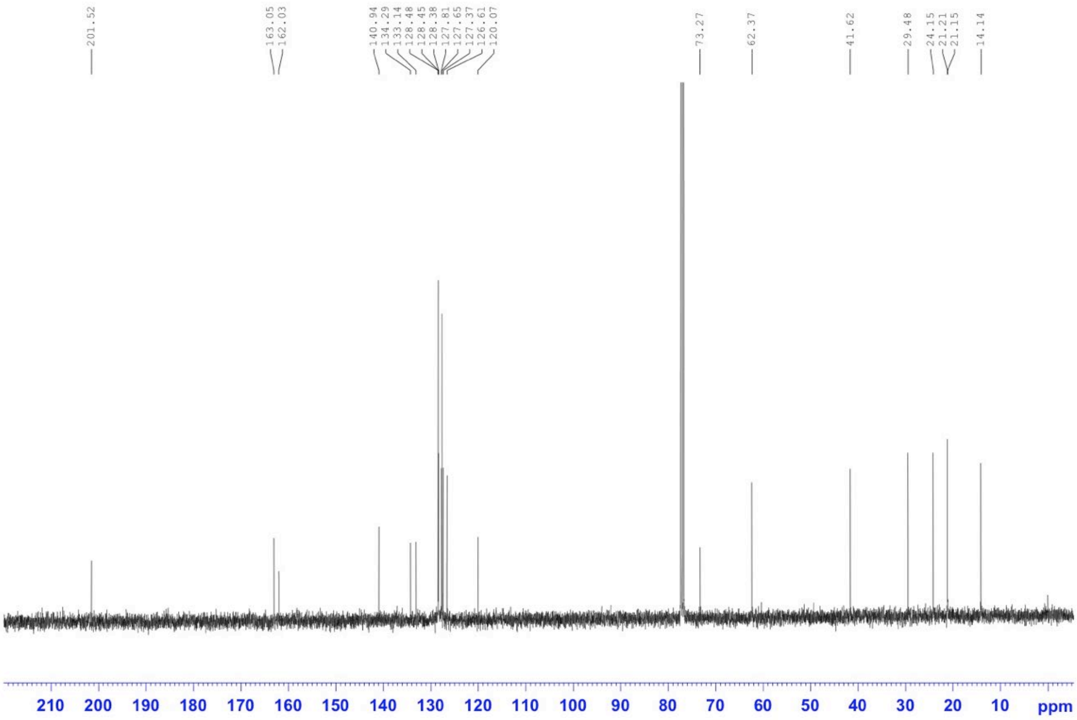

TWW-02-385-p, 1H, BBFO1, CDCl3

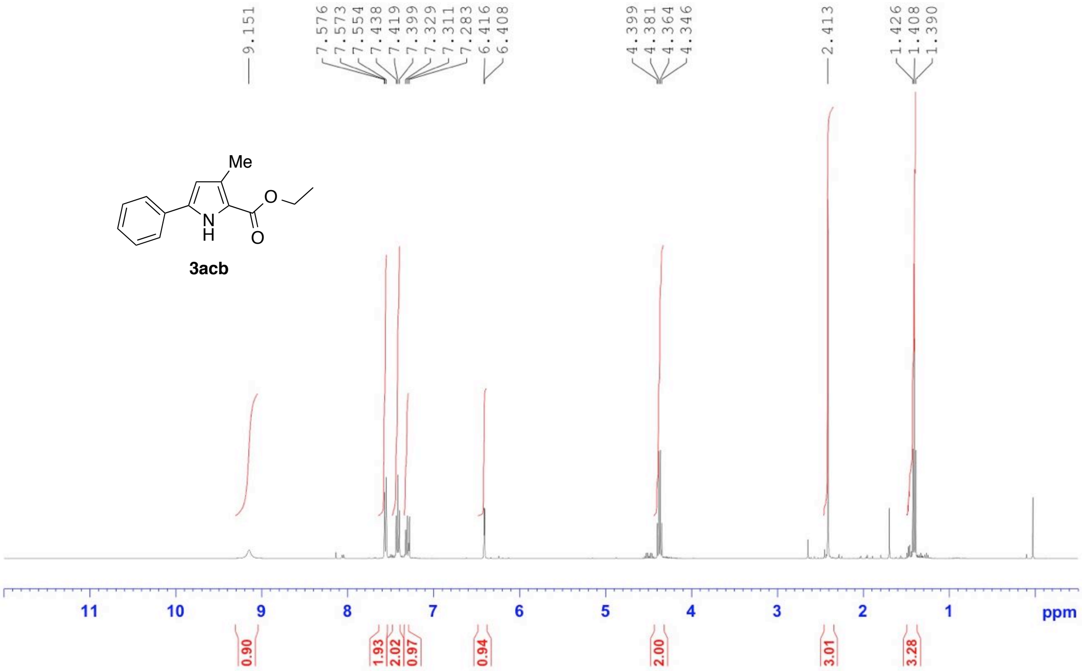

TWW-02-385-p, 13C, BBFO1, CDCl3

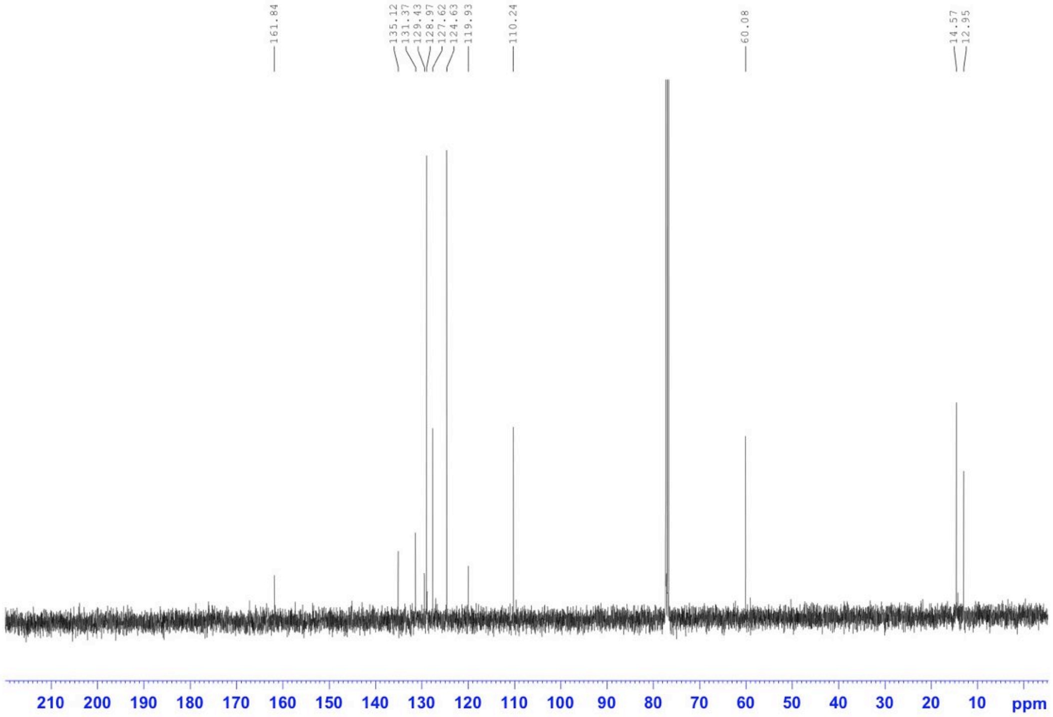

TWW-02-385, 1H, BBFO1, CDCl3

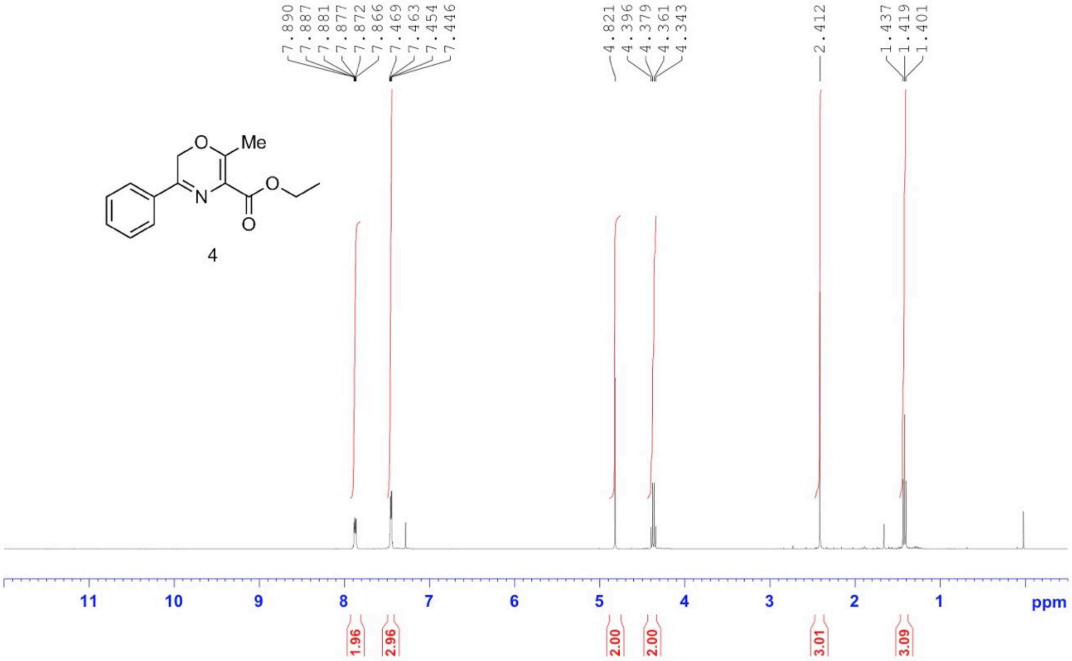

TWW-02-385, 13C, BBFO1, CDCl3

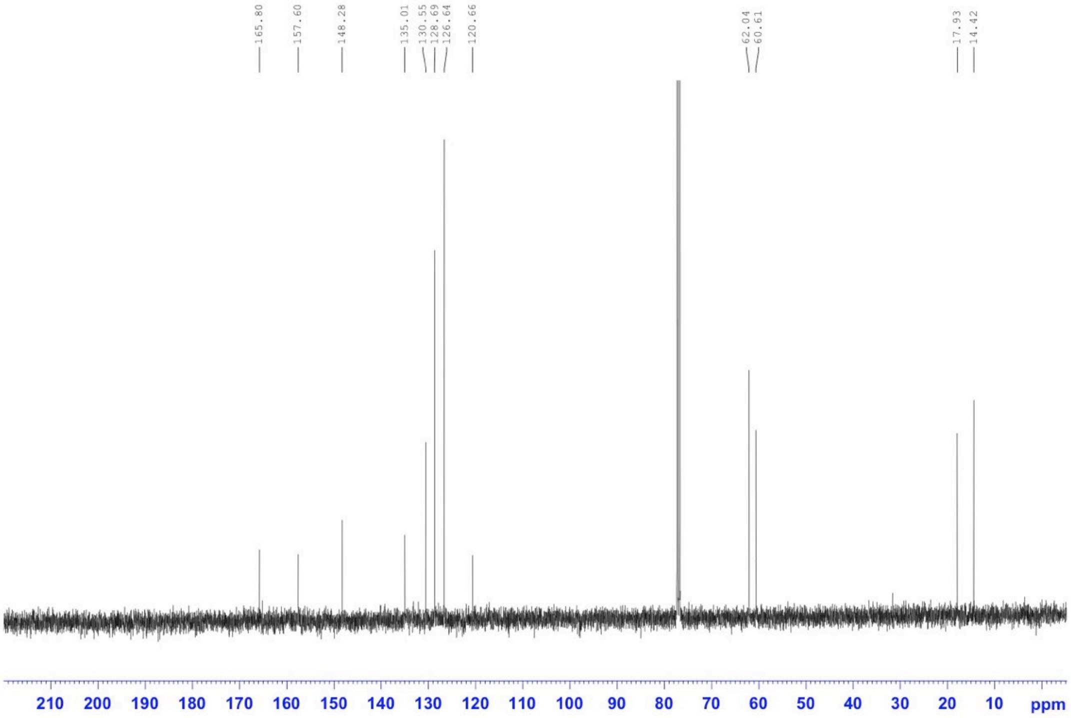

TWW-02-374-2, 1H, BBFO1, CDCl3

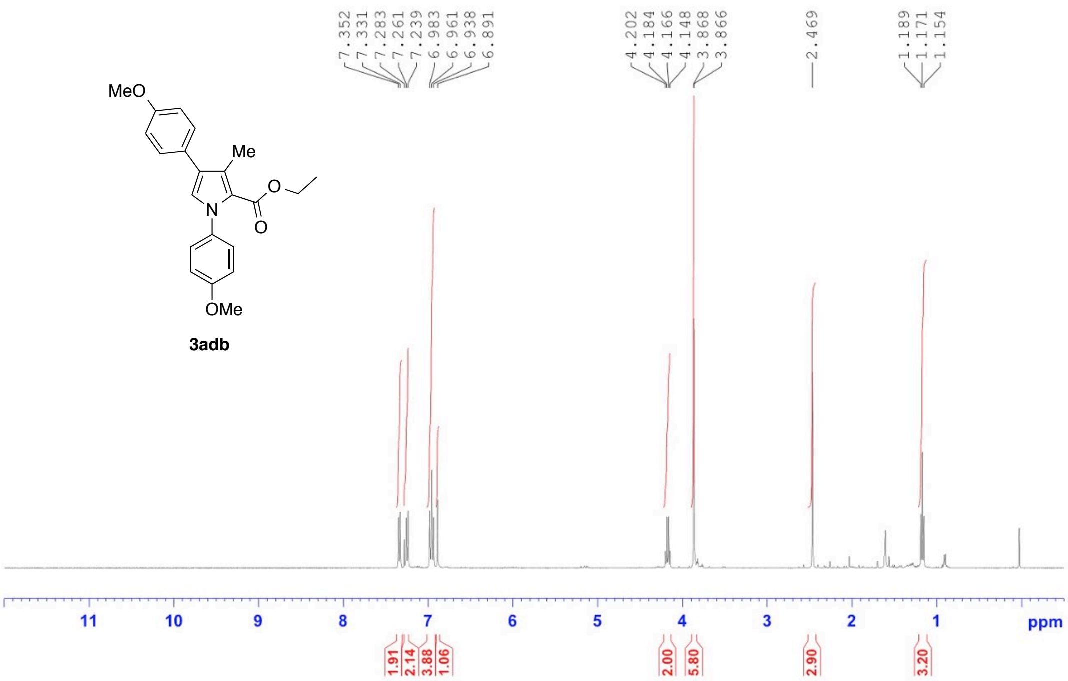

TWW-02-374-2, 13C, BBFO1, CDCl3

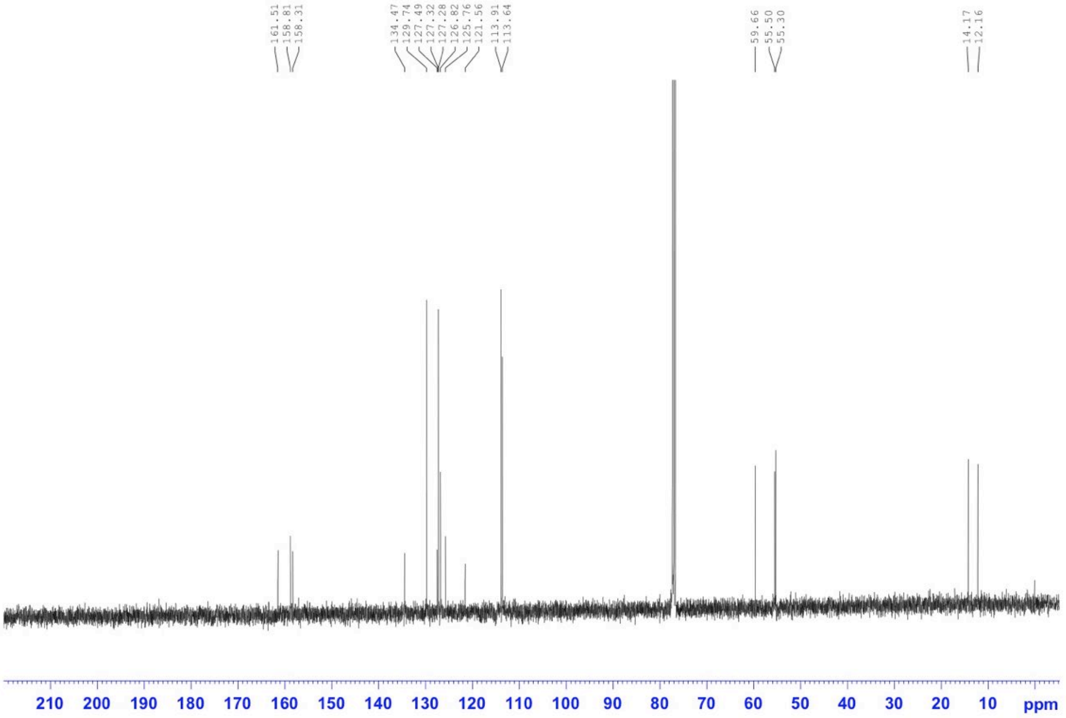

TWW-02-227, 1H, BBFO1, CDCl3

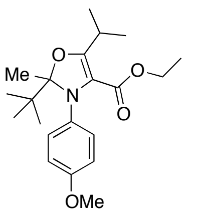

5a

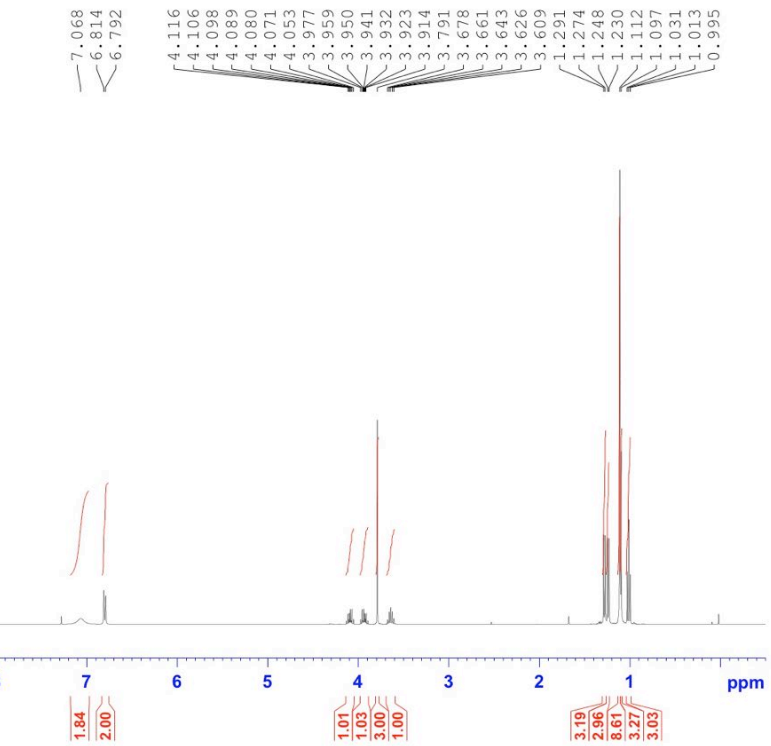

TWW-02-227, 13C, BBFO1, CDCl3

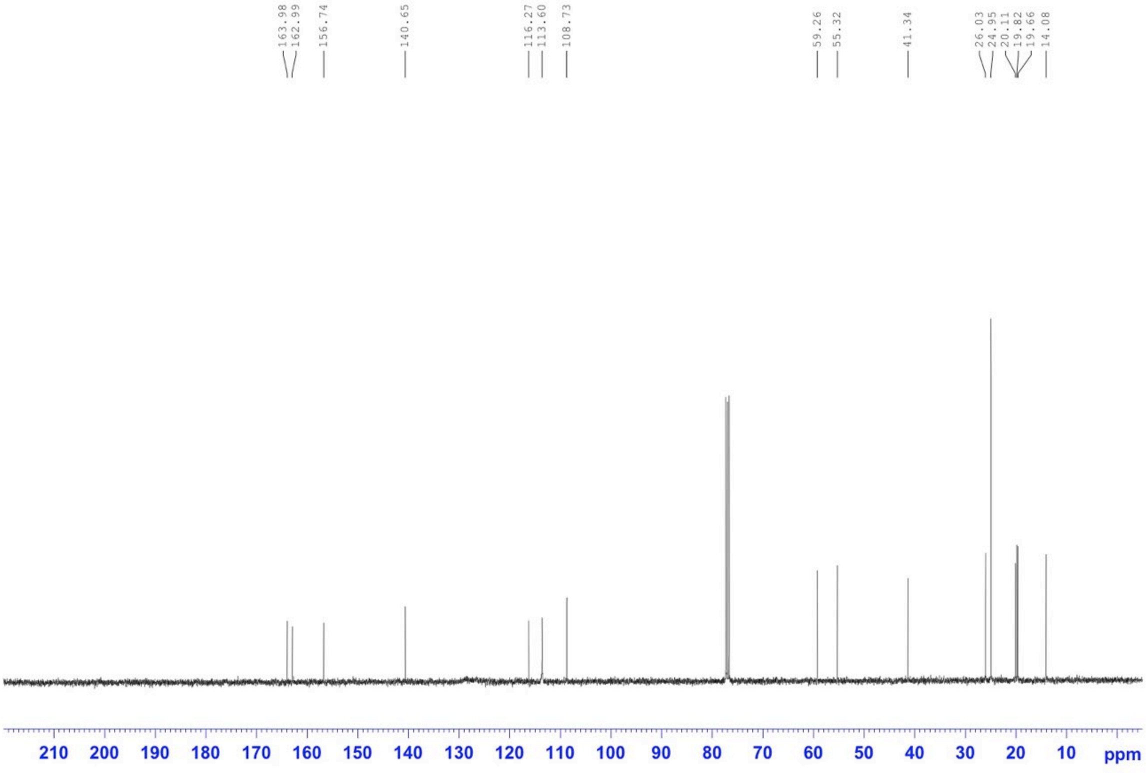

TWW-02-213, 1H, BBFO1, CDCl3

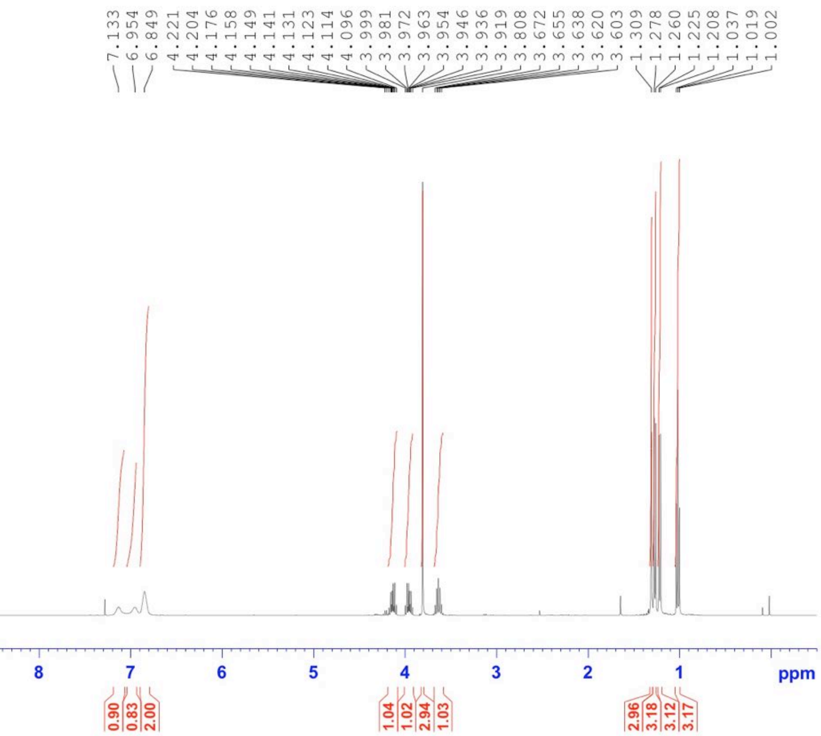

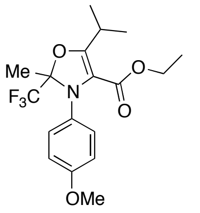

5b

TWW-02-213-R BBFO1 CDCl3 13C

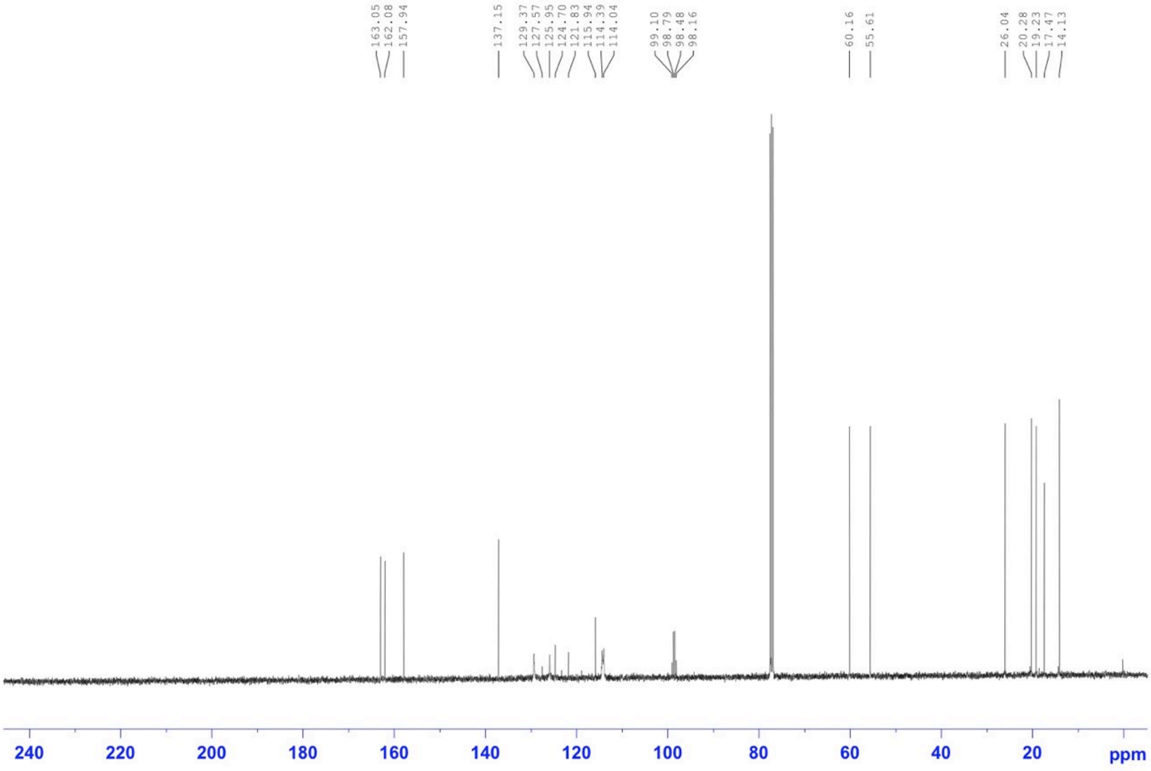

TWW-02-067, BBFO, 1H, CDCl3

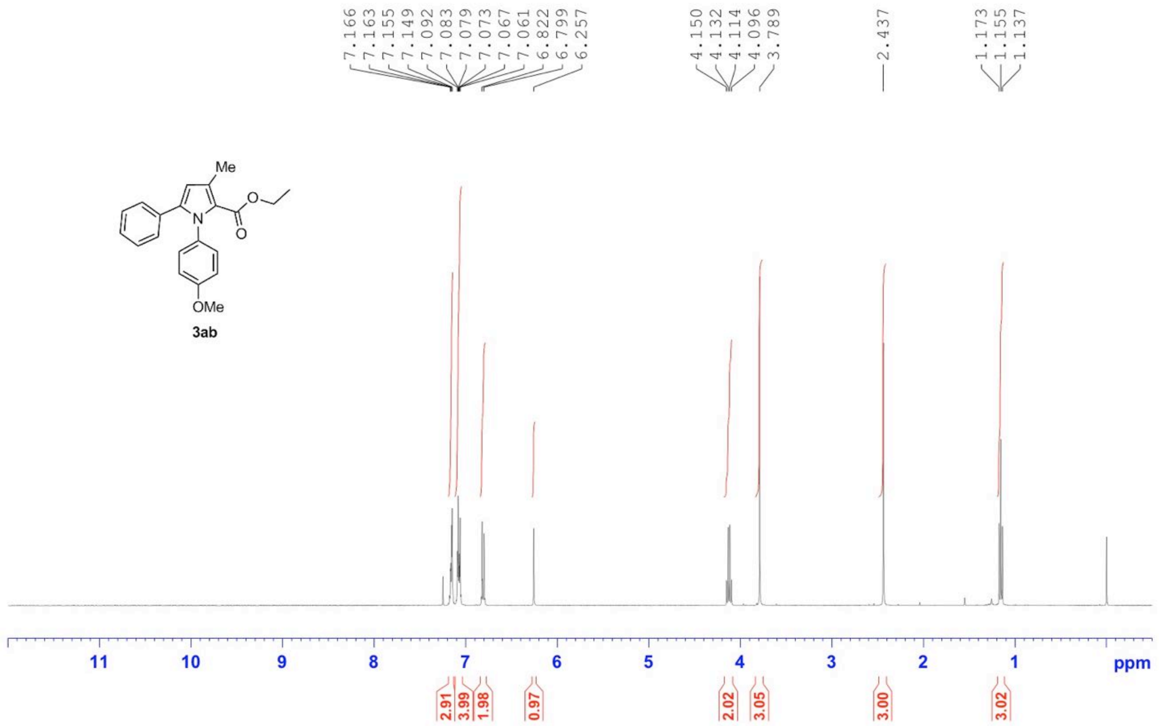

TWW-02-067, BBFO, 13C, CDCl3

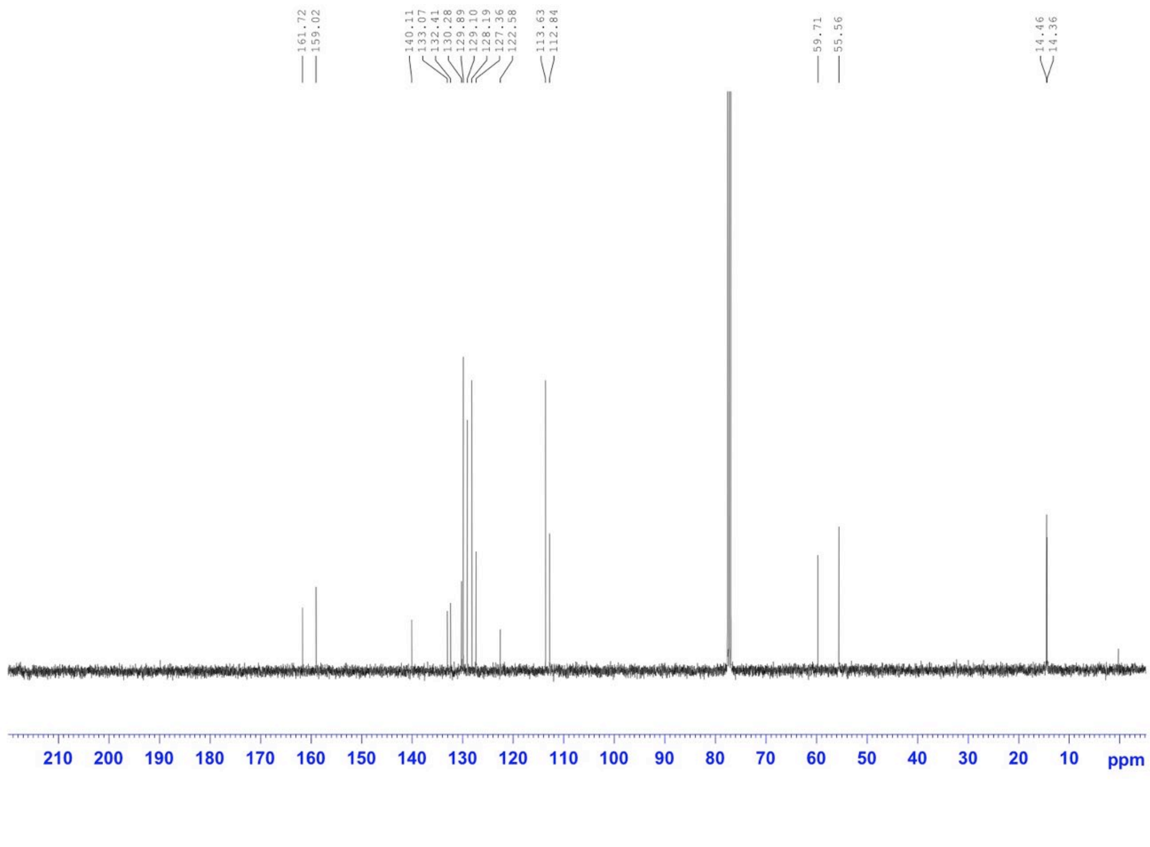

TWW-02-200, 1H BBFO1, CDCl3

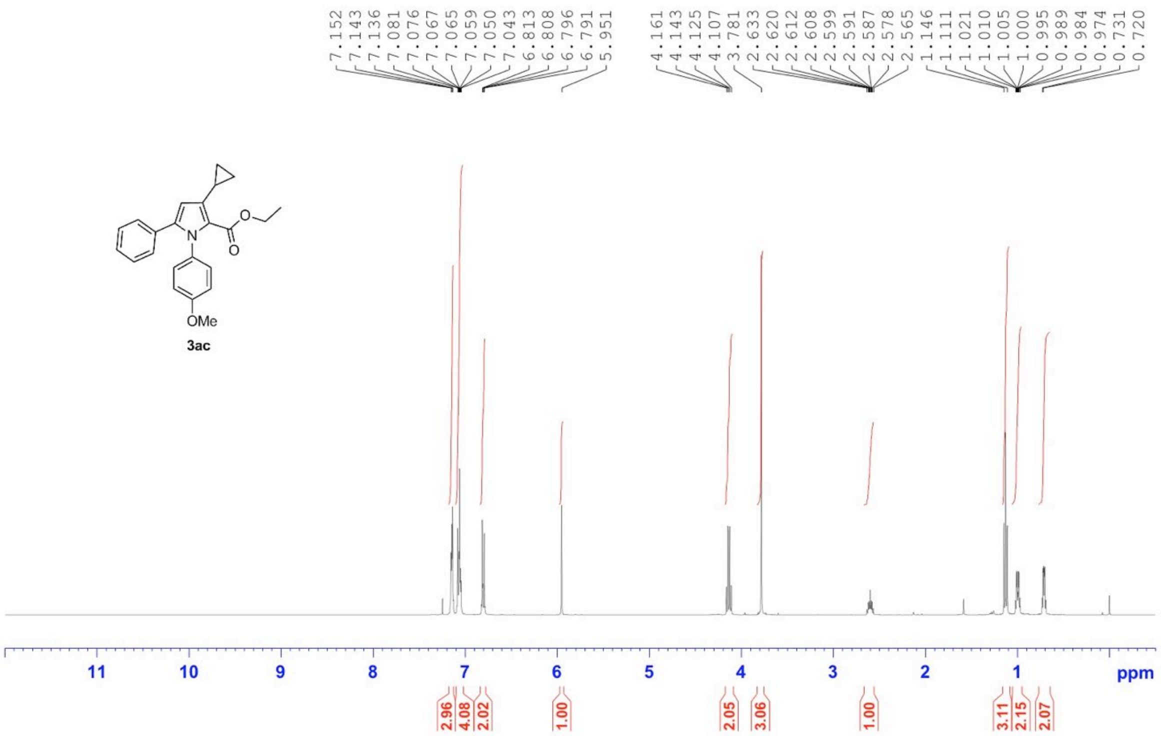

TWW-02-200, 13C BBFO1, CDCl3

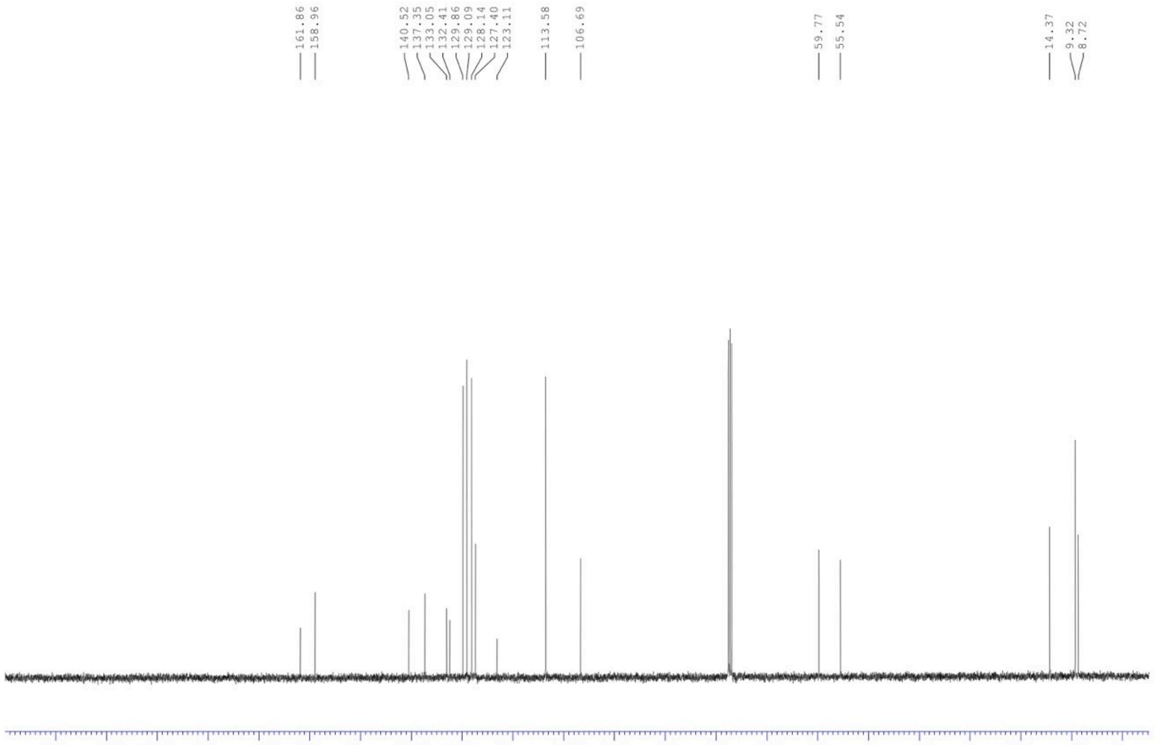

TWW-02-203, 1H, BBFO1, CDCl3

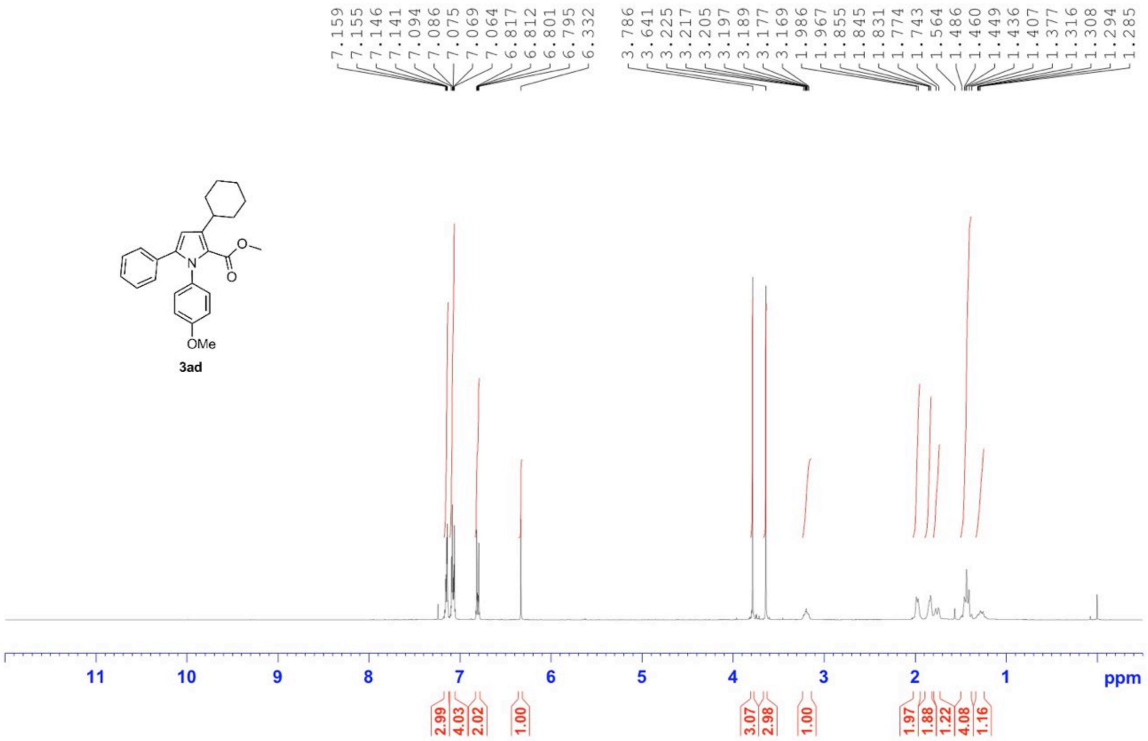

TWW-02-203, 13C, BBFO1, CDCl3

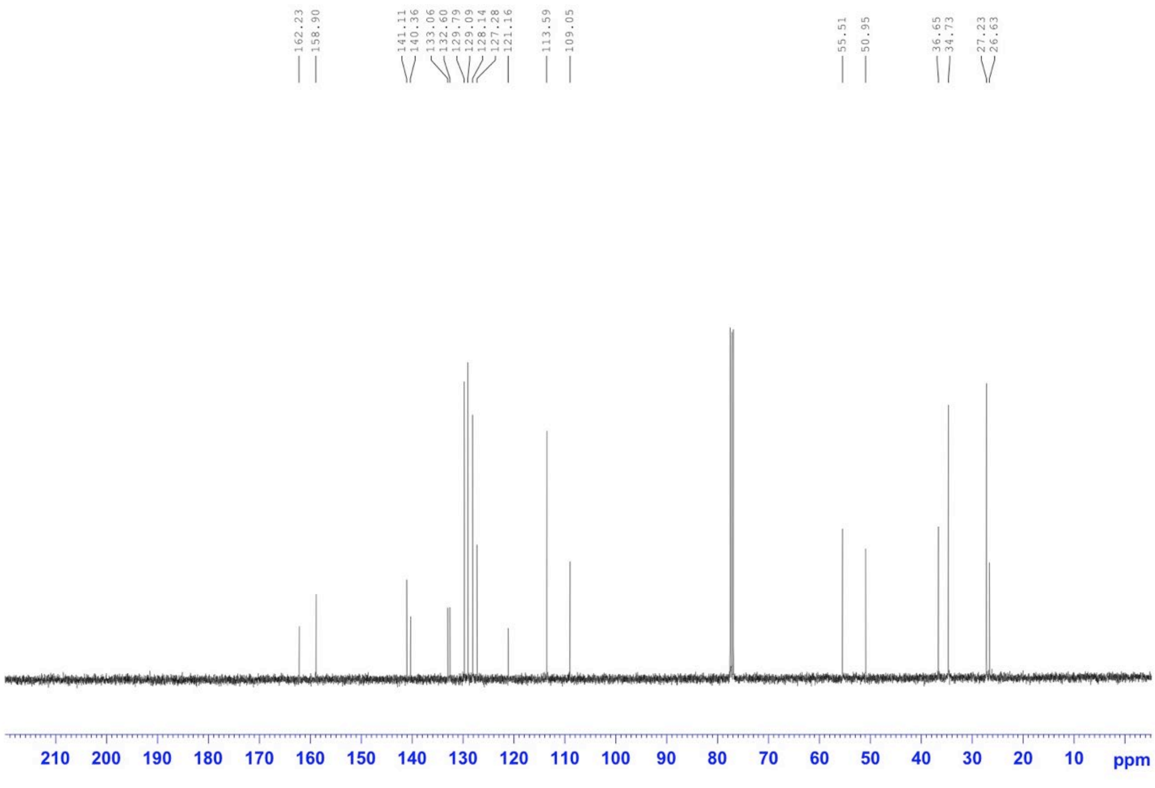

TWW-02-182, 1H BBFO1, CDCl3

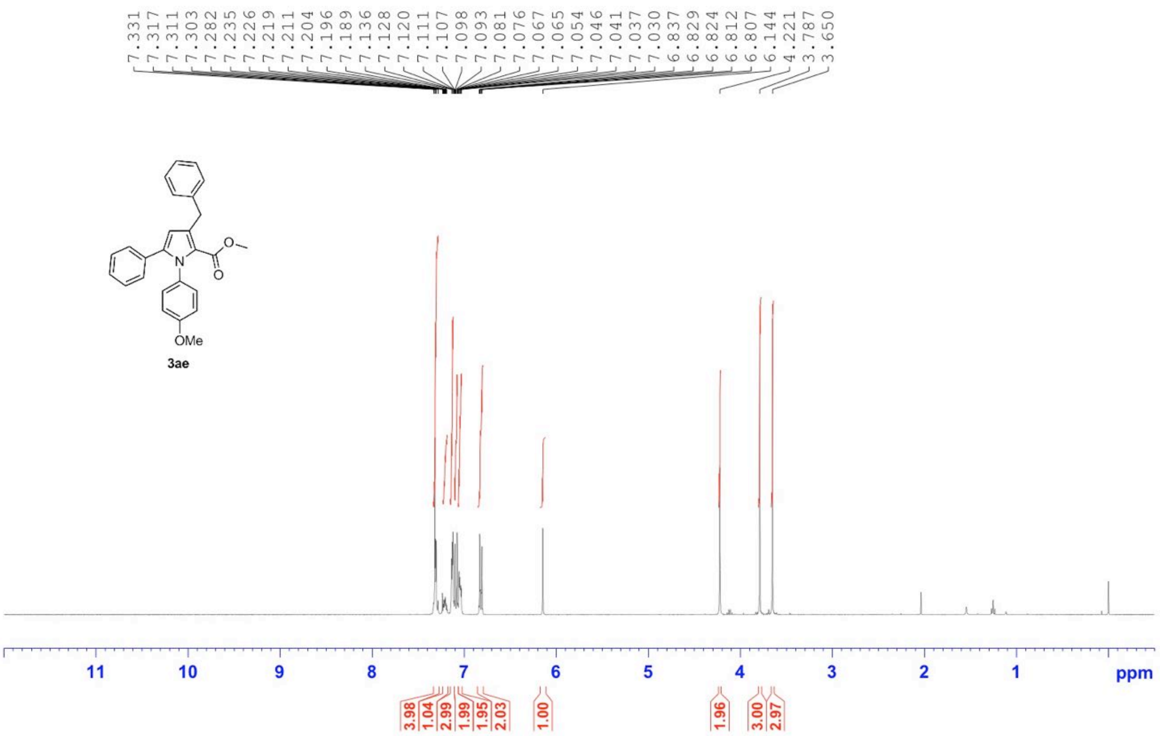

TWW-02-182, 13C BBFO1, CDCl3

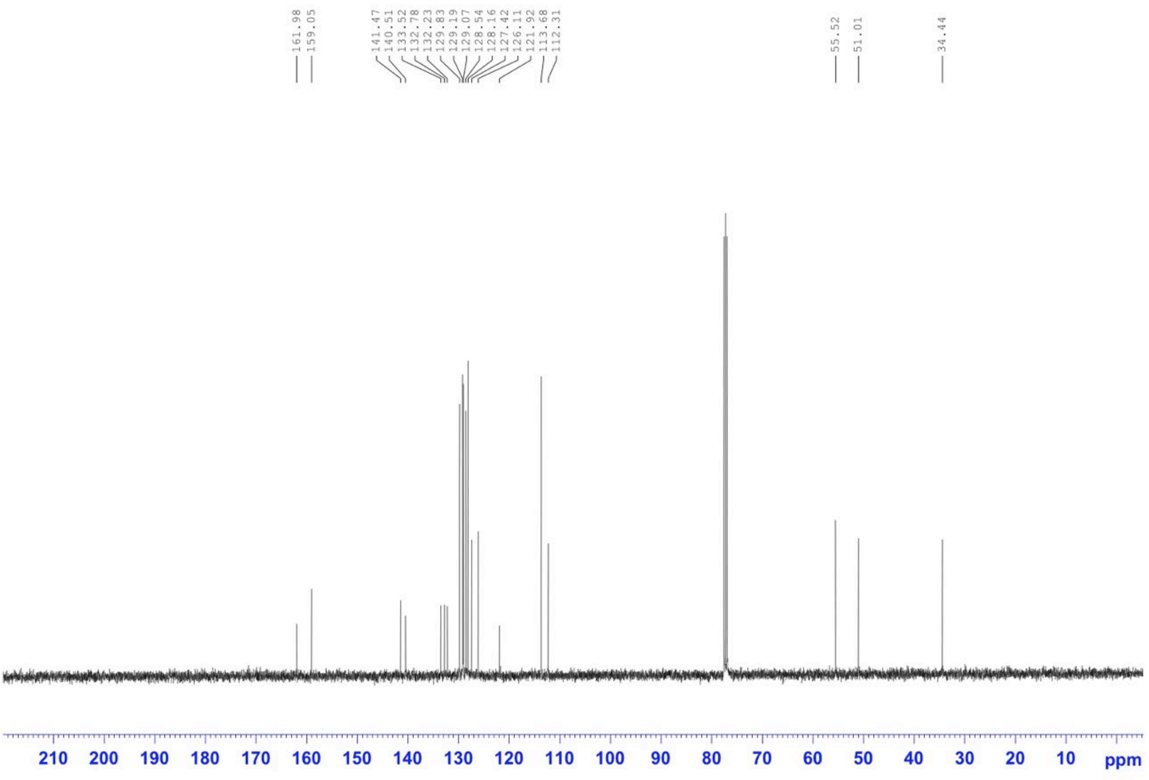

TWW-02-057, BBFO, 1H, CDCl3

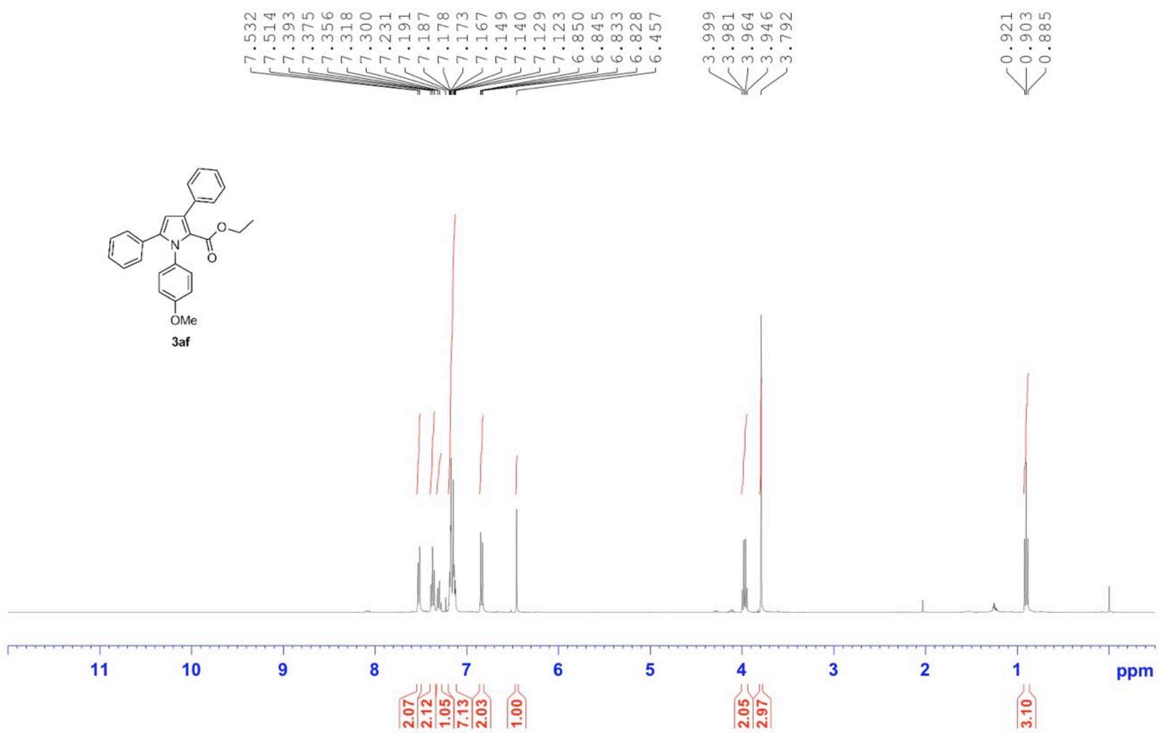

TWW-02-057, BBFO, 13C, CDCl3

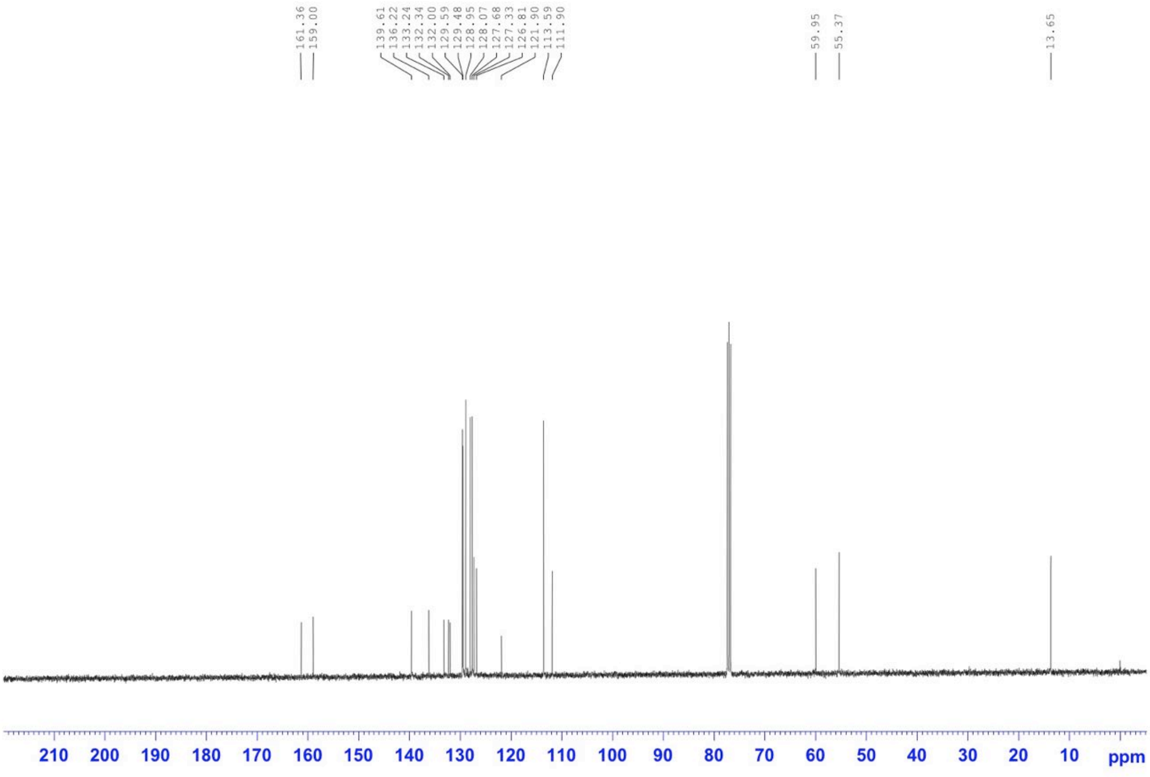

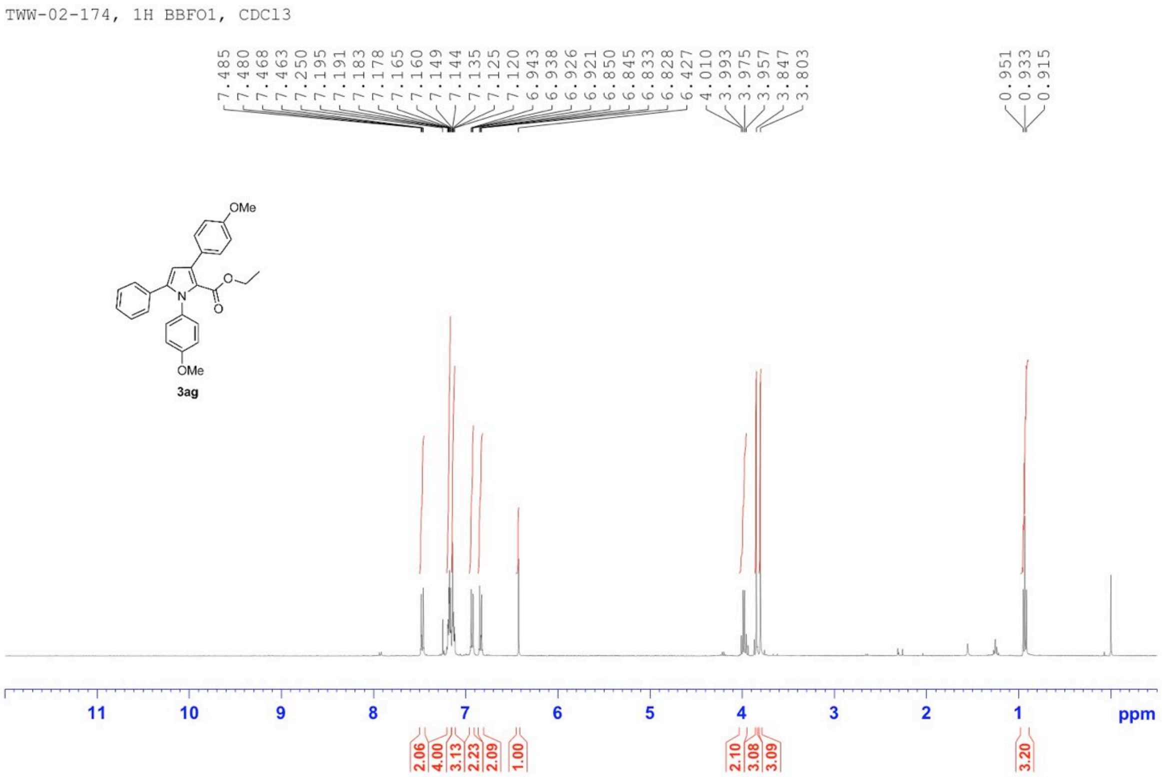
TWW-02-174, 1H BBFO1, CDCl3
7.485
7.480
7.468
7.463
7.250
7.195
7.191
7.183
7.178
7.165
7.160
7.149
7.144
7.135
7.125
7.120
6.943
6.938
6.926
6.921
6.850
6.845
6.833
6.828
6.427
4.010
3.993
3.975
3.957
3.847
3.803
0.951
0.933
0.915
OMe
N
O
O
OMe
3ag
11
10
9
8
7
6
5
4
3
2
1
ppm
2.06
4.00
3.13
2.23
2.09
1.00
2.10
3.08
3.09
3.20

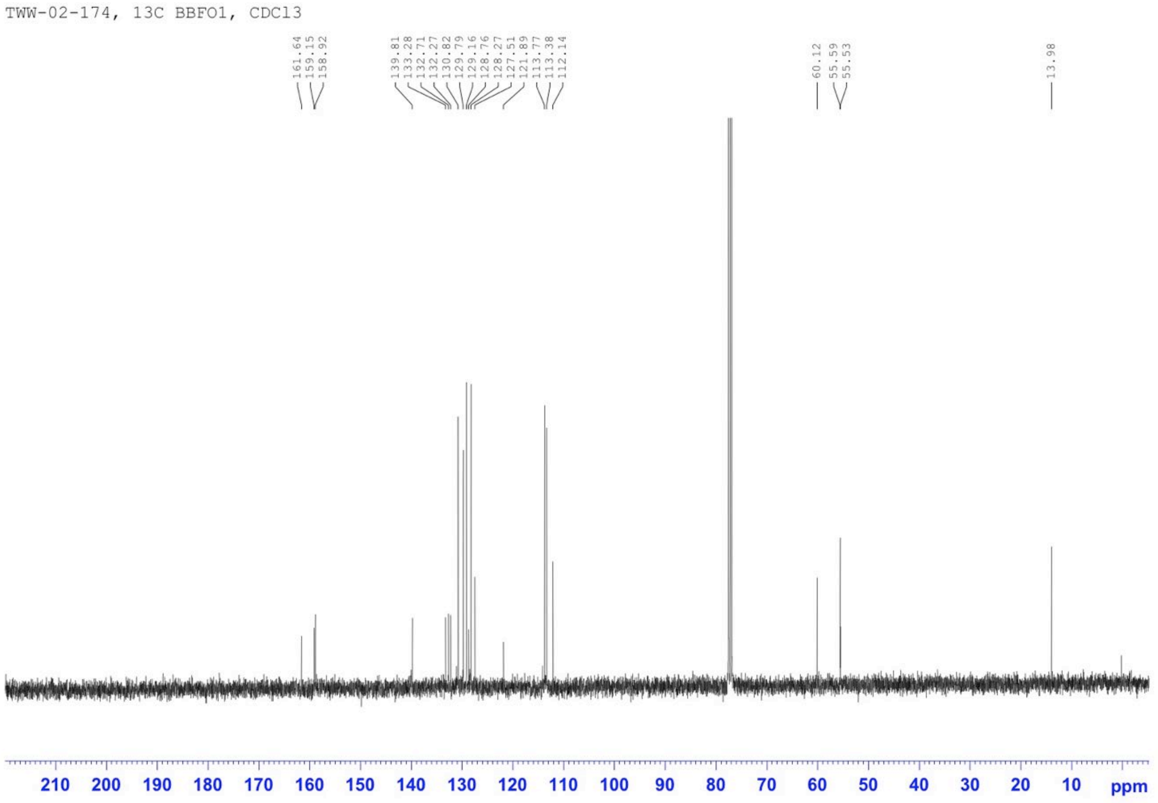
TWW-02-174, 13C BBFO1, CDCl3
161.64
159.15
158.92
139.81
133.28
132.71
132.27
130.82
129.79
129.16
128.76
128.27
127.51
121.89
113.77
113.38
112.14
60.12
55.59
55.53
13.98
210
200
190
180
170
160
150
140
130
120
110
100
90
80
70
60
50
40
30
20
10
ppm

TWW-02-202, 1H, BBFO1, CDCl3

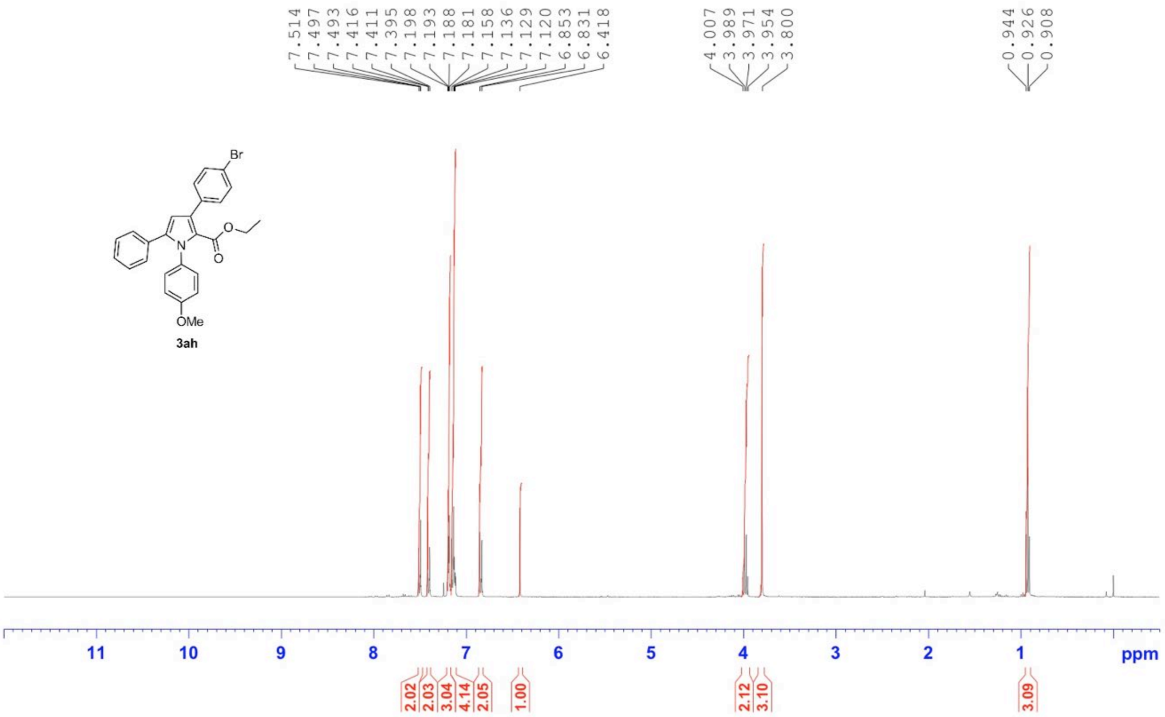

TWW-02-202, 13C, BBFO1, CDCl3

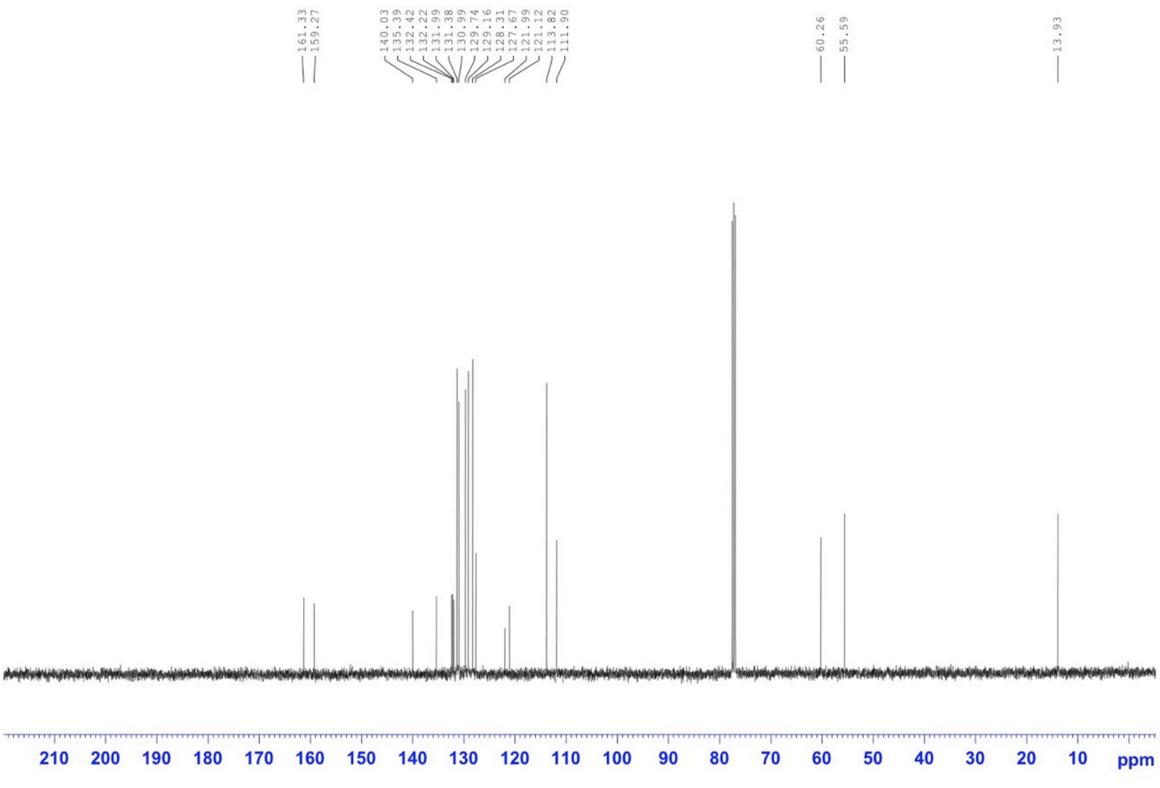

TWW-02-175, 1H BBFO1, CDCl3

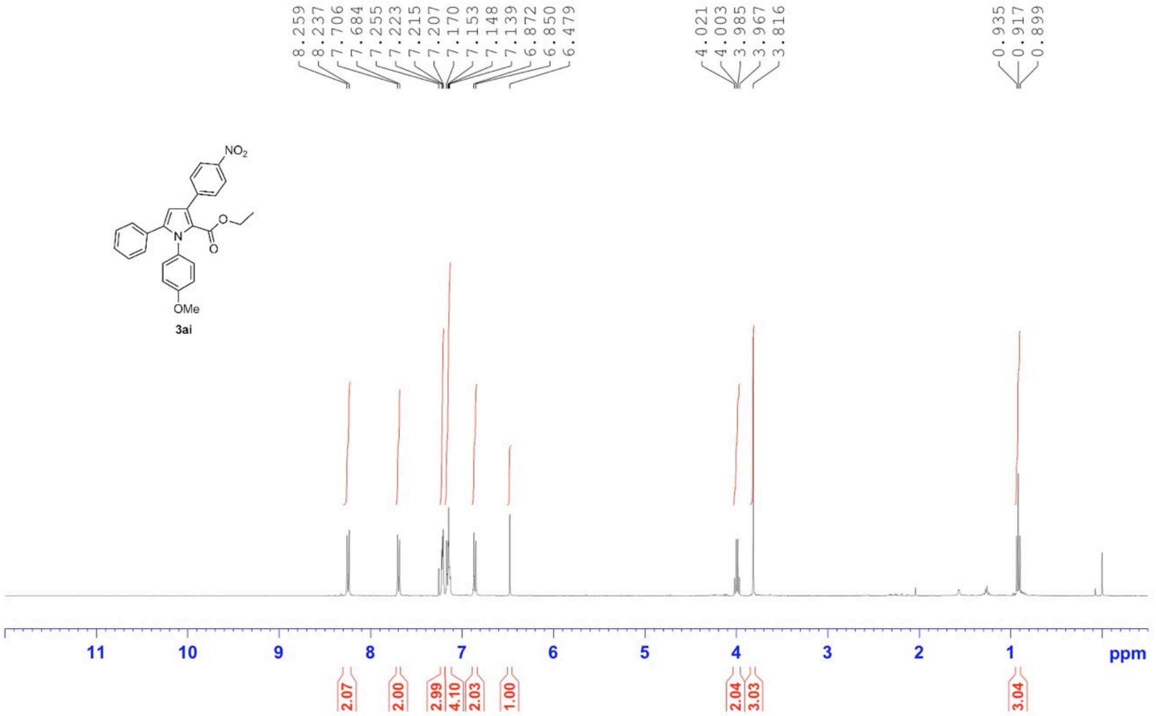

TWW-02-175, 13C BBFO1, CDCl3

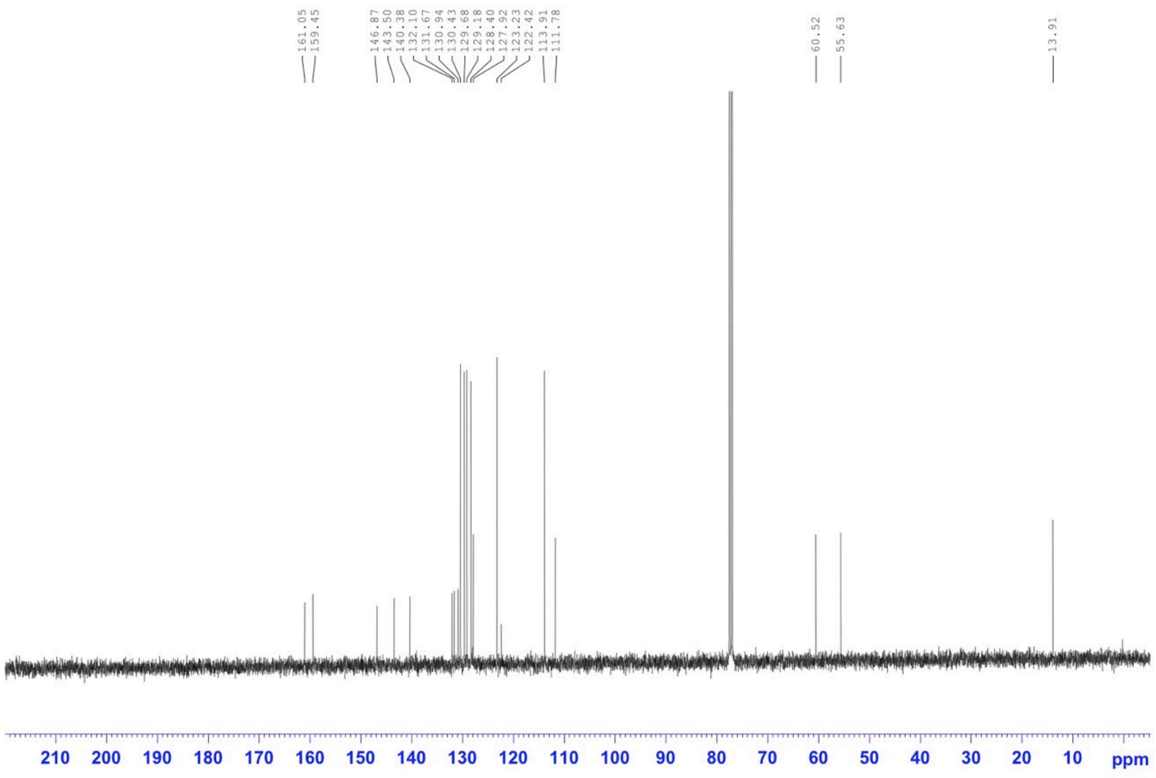

TWW-02-201, 1H, BBFO1, CDCl3

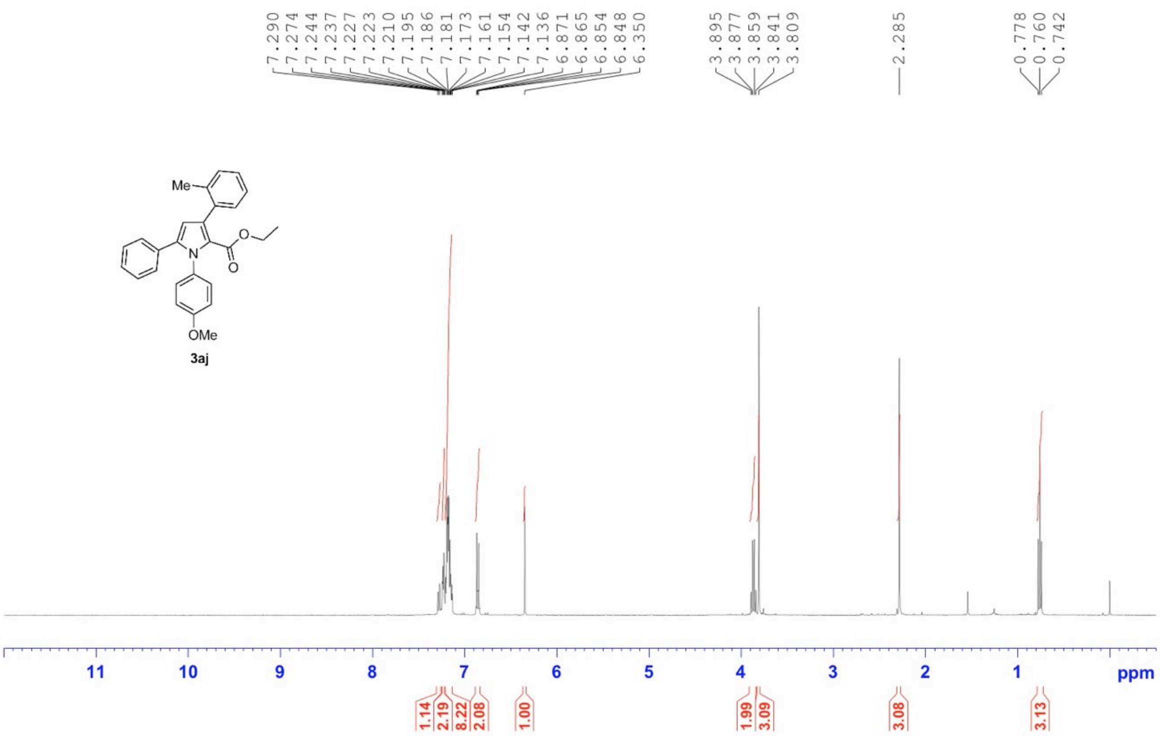

TWW-02-201, 13C, BBFO1, CDCl3

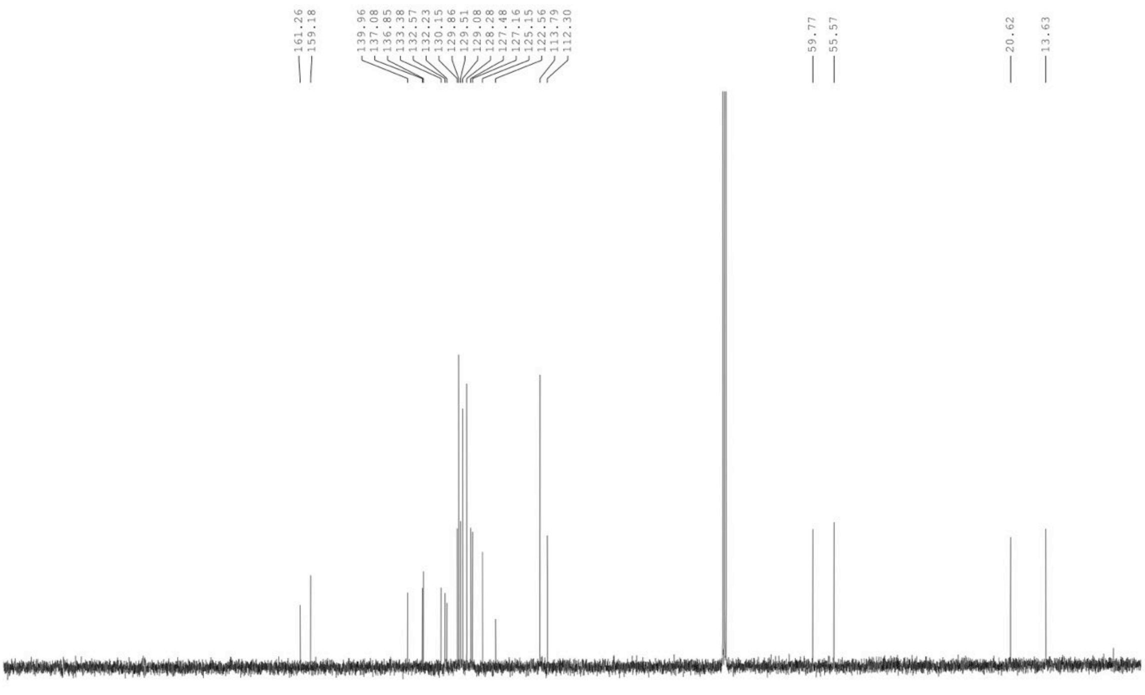

TWW-02-211, 1H, BBFO1, CDCl3

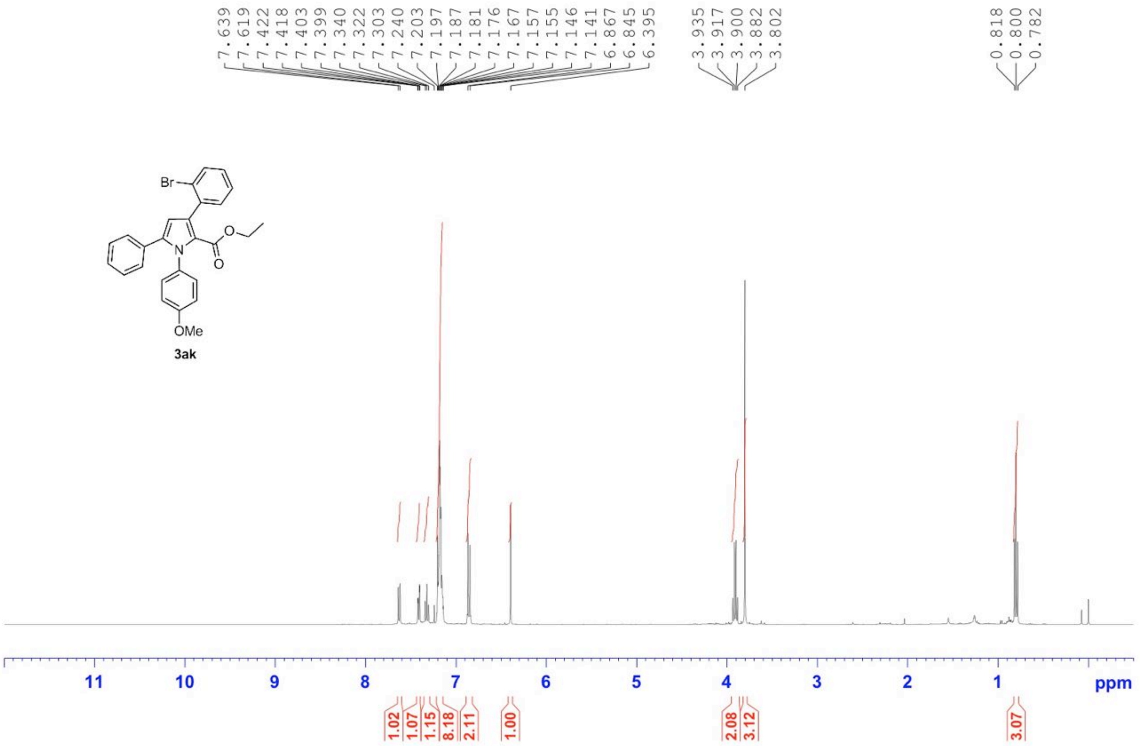

TWW-02-211, 13C, BBFO1, CDCl3

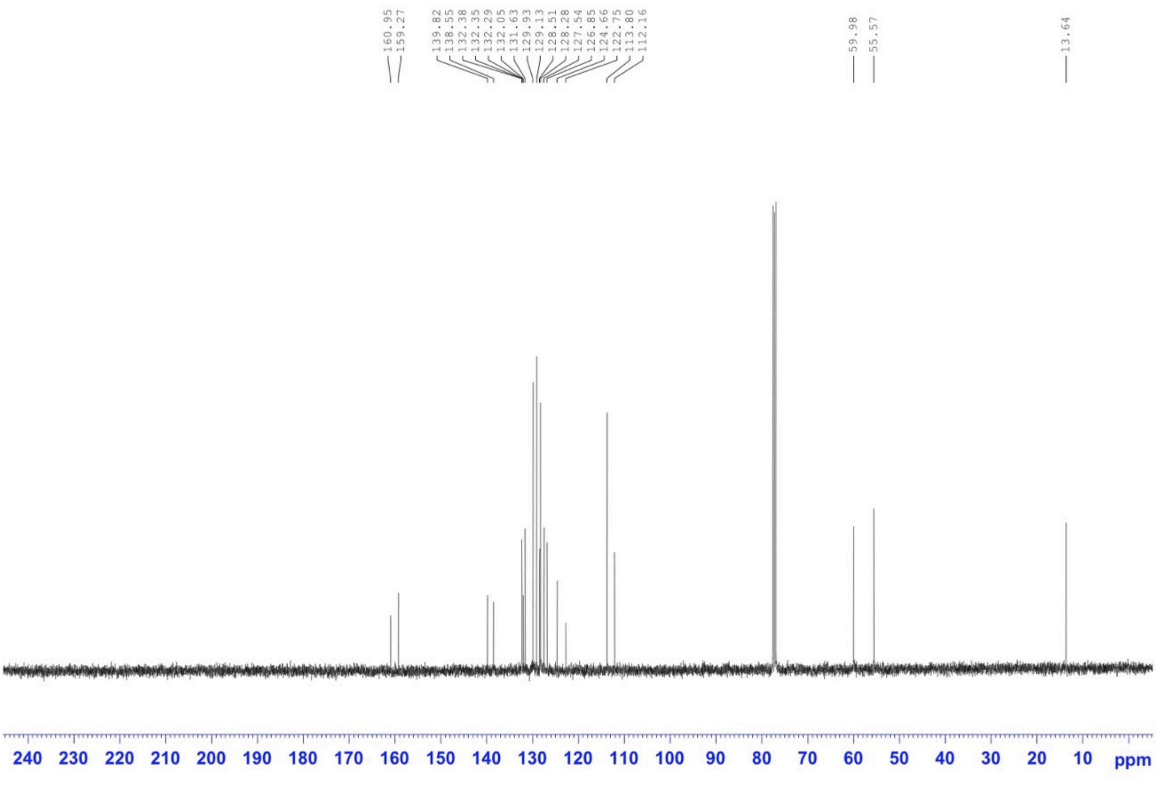

TWW-02-210, 1H, BBFO1, CDCl3

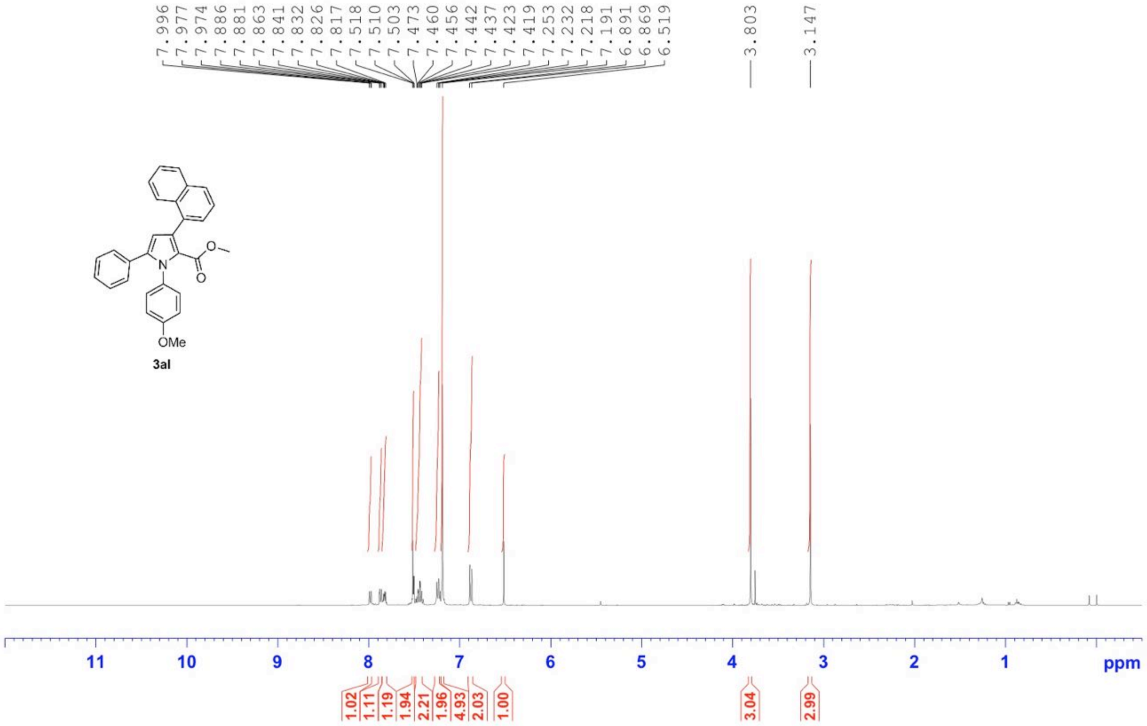

TWW-02-210-1, 13C NMR, 400M Hz, BBFO2

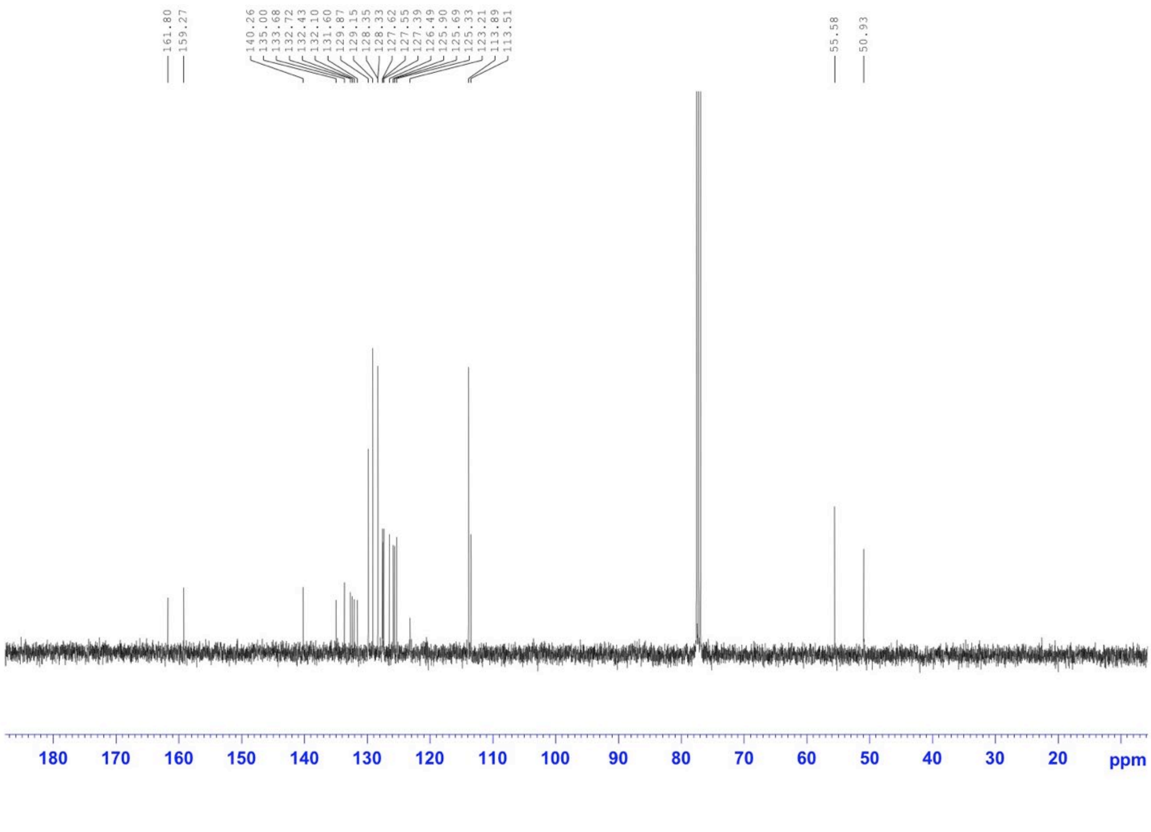

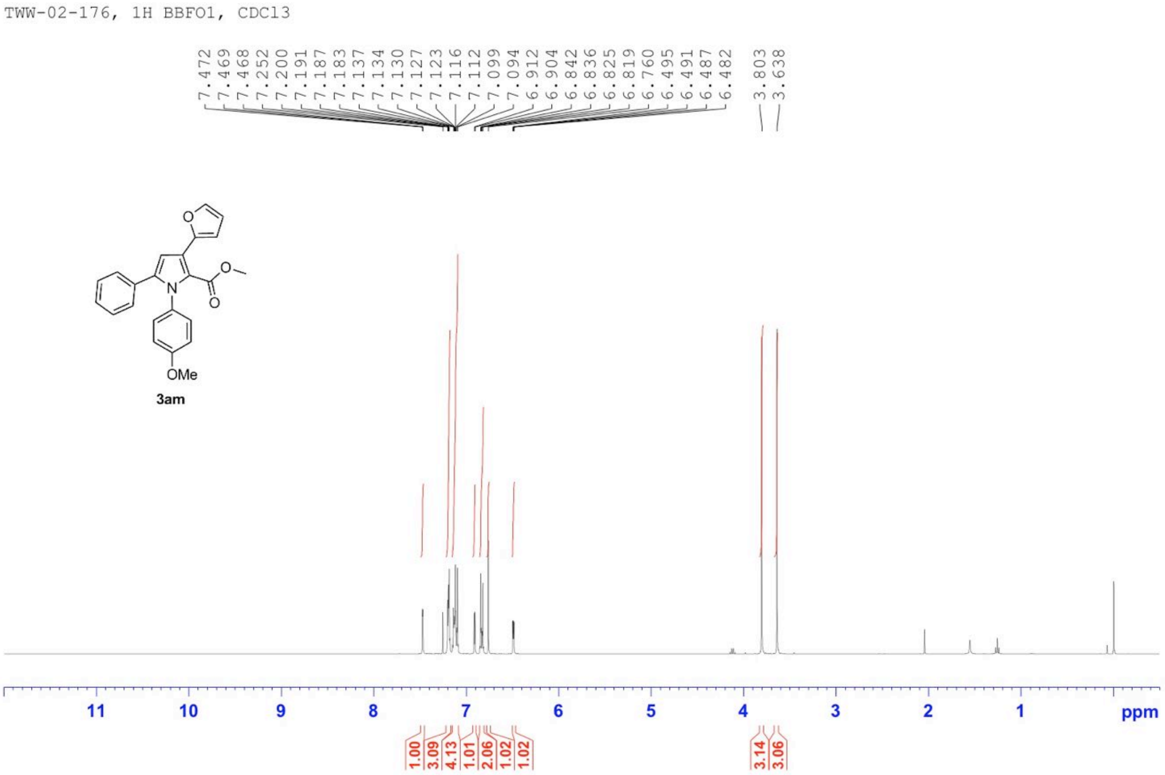
TWW-02-176, 1H BBFO1, CDCl3
7.472
7.469
7.468
7.252
7.200
7.191
7.187
7.183
7.137
7.134
7.130
7.127
7.123
7.116
7.112
7.099
7.094
6.912
6.904
6.842
6.836
6.825
6.819
6.760
6.495
6.491
6.487
6.482
3.803
3.638
OMe
3am
11
10
9
8
7
6
5
4
3
2
1
ppm
1.00
3.09
4.13
1.01
2.06
1.02
1.02
3.14
3.06

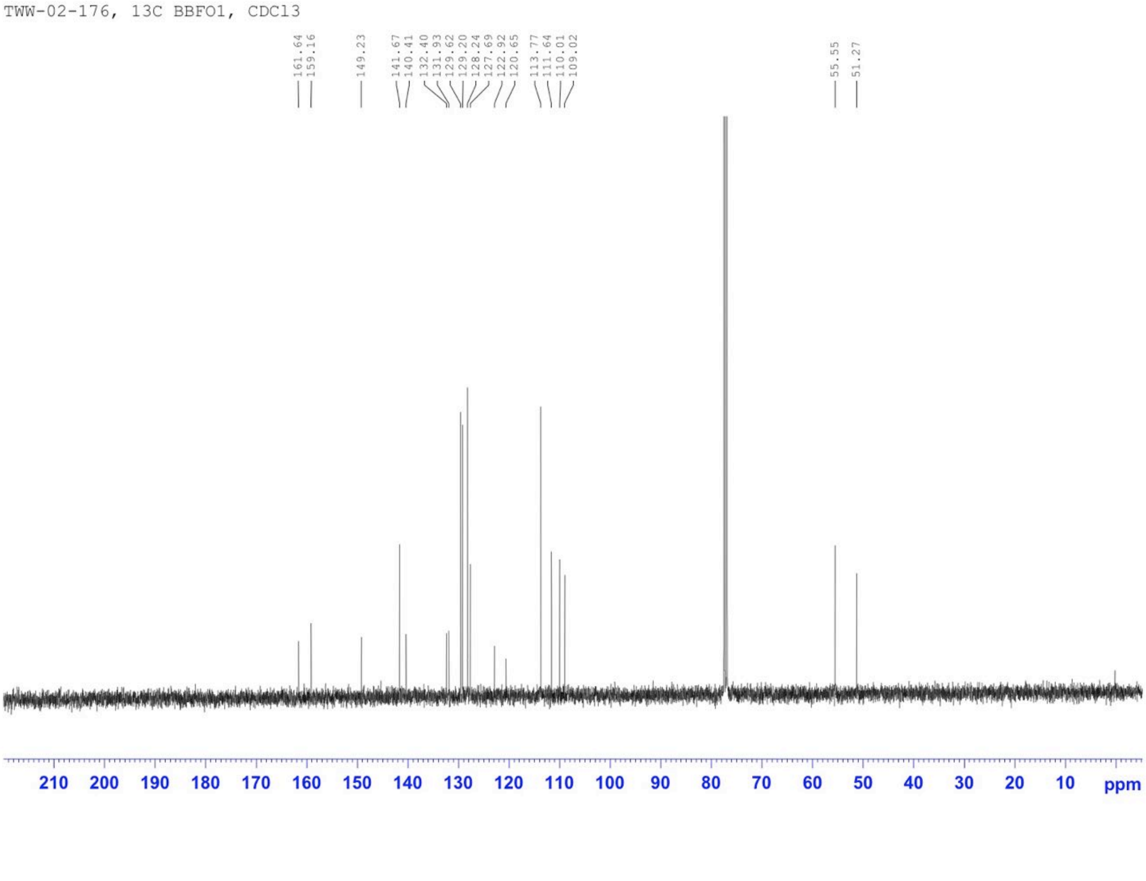
TWW-02-176, 13C BBFO1, CDCl3
161.64
159.16
149.23
141.67
140.41
132.40
131.93
129.62
129.20
128.24
127.69
122.92
120.65
113.77
111.64
110.01
109.02
55.55
51.27
210
200
190
180
170
160
150
140
130
120
110
100
90
80
70
60
50
40
30
20
10
ppm

TWW-02-186, 1H BBFO1, CDCl3

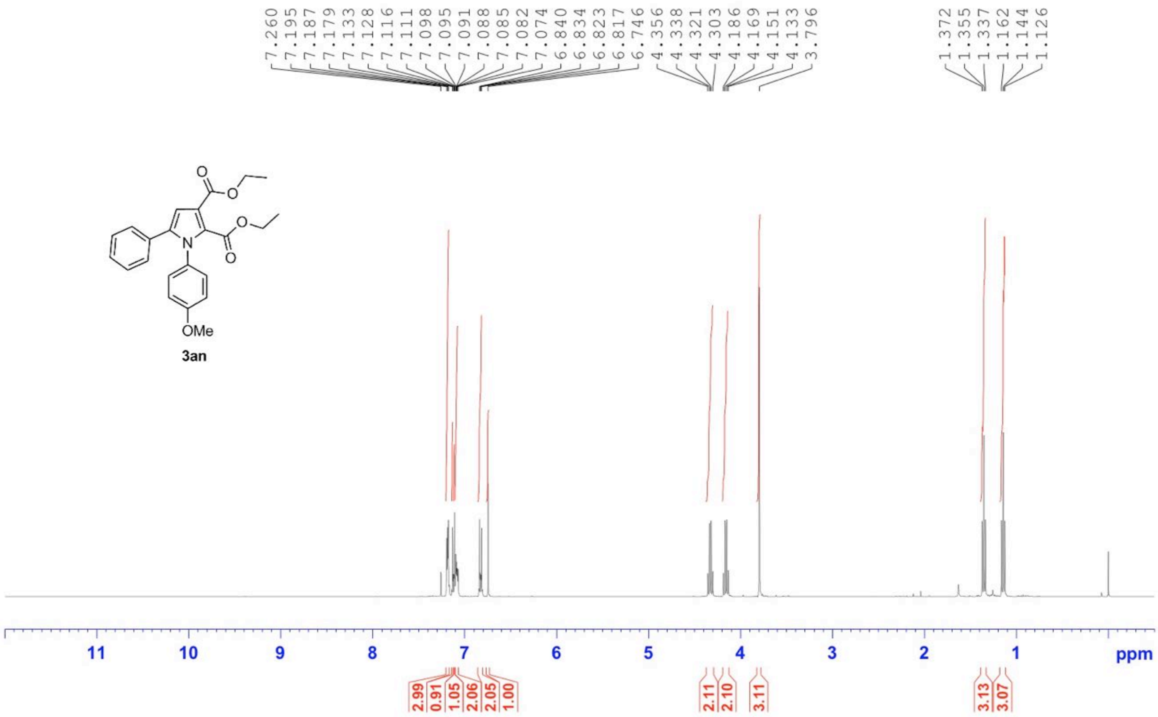

TWW-02-186, 13C BBFO1, CDCl3

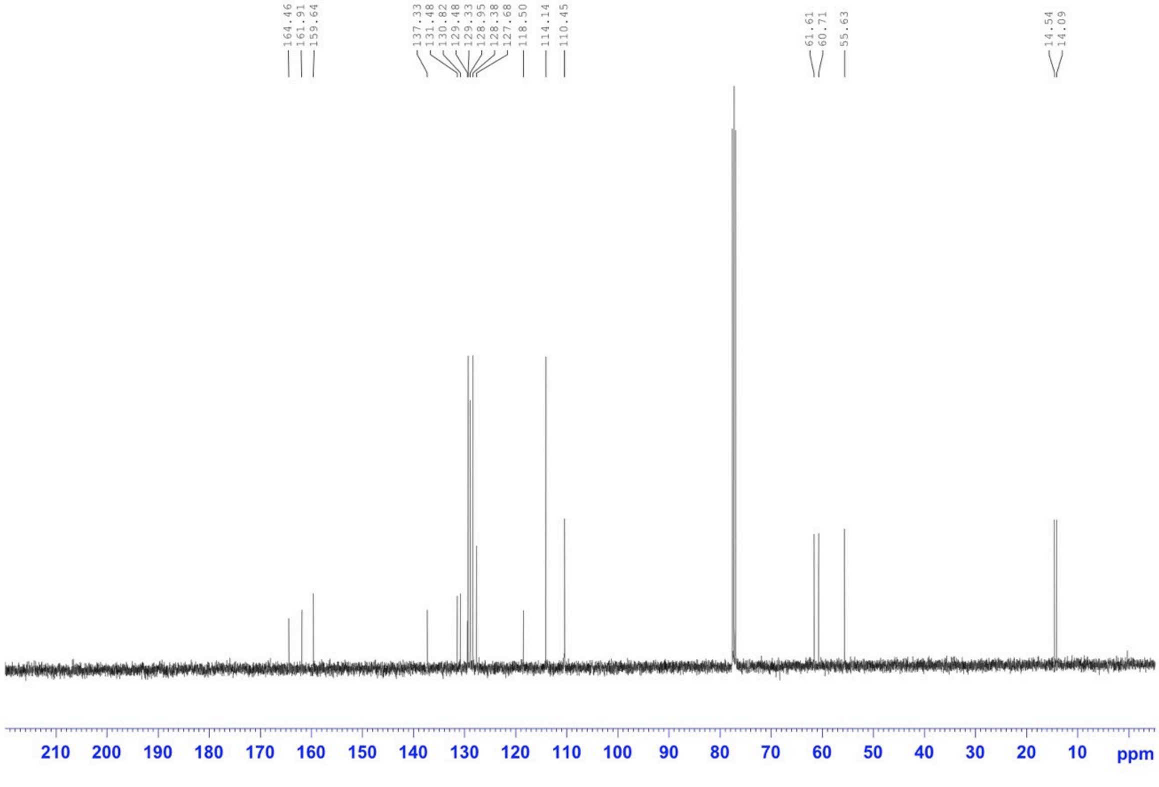

TWW-02-214, 1H, BBFO1, CDCl3

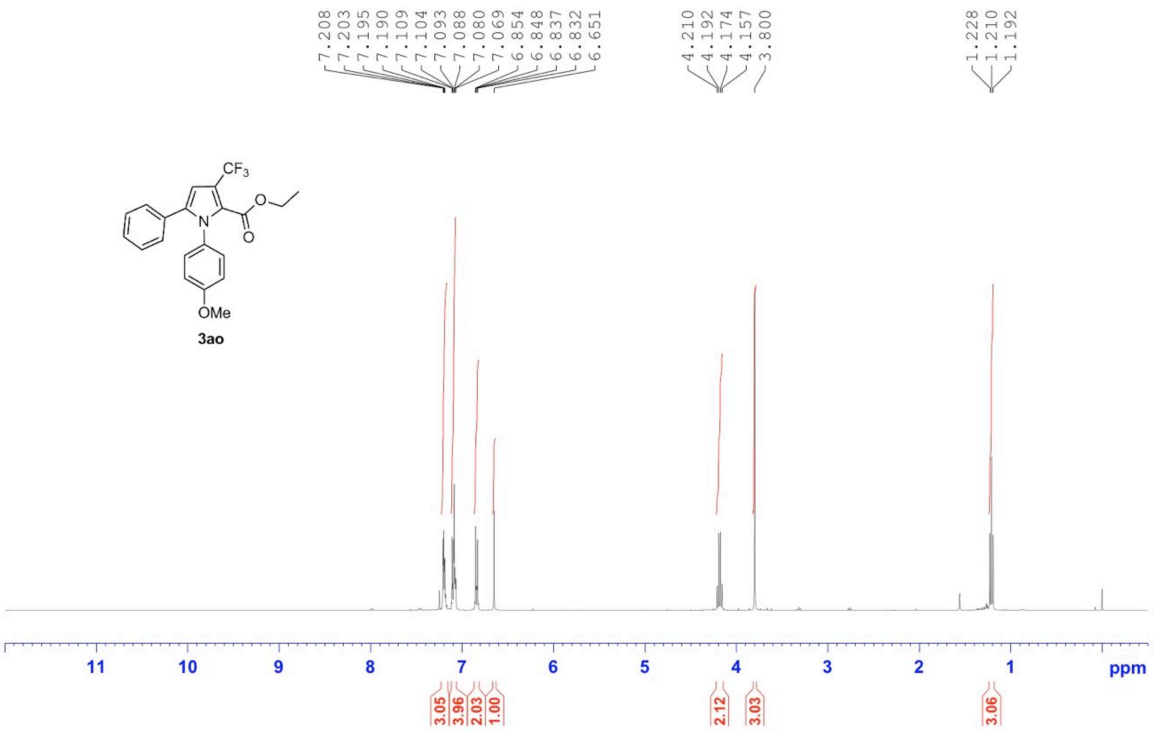

TWW-02-214, 13C, BBFO1, CDCl3

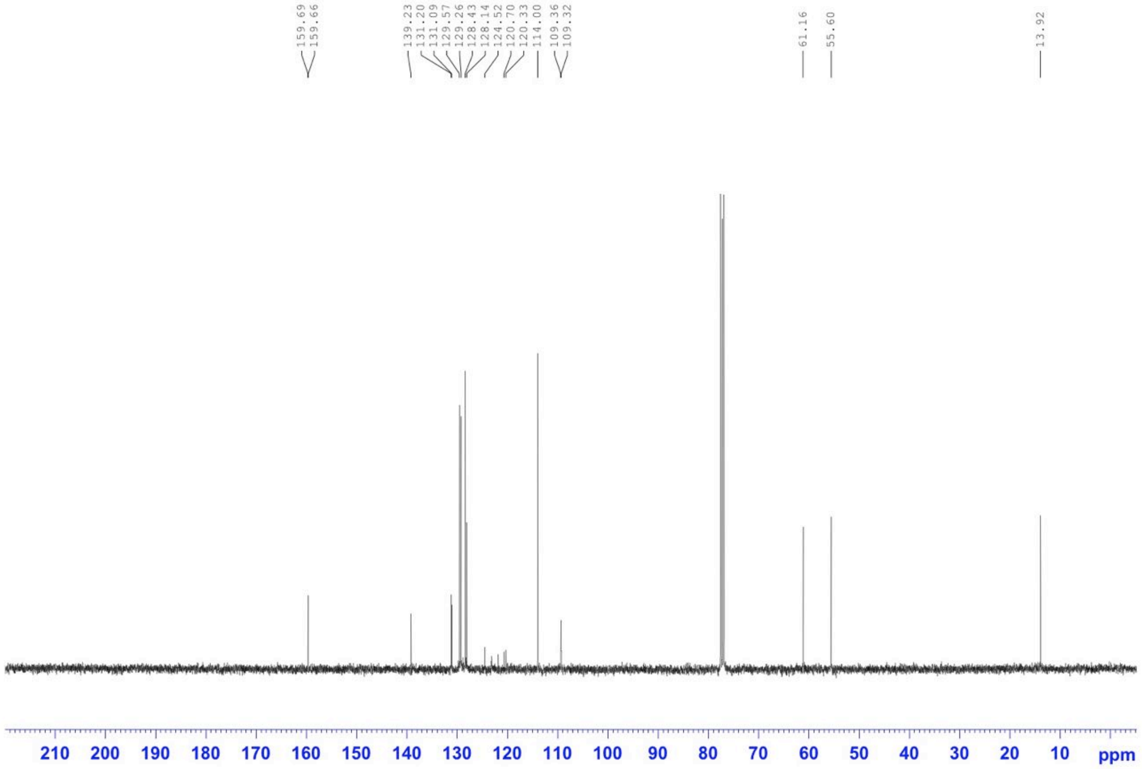

TWW-02-362-2 1HNMR
BBFO1 CDCl3

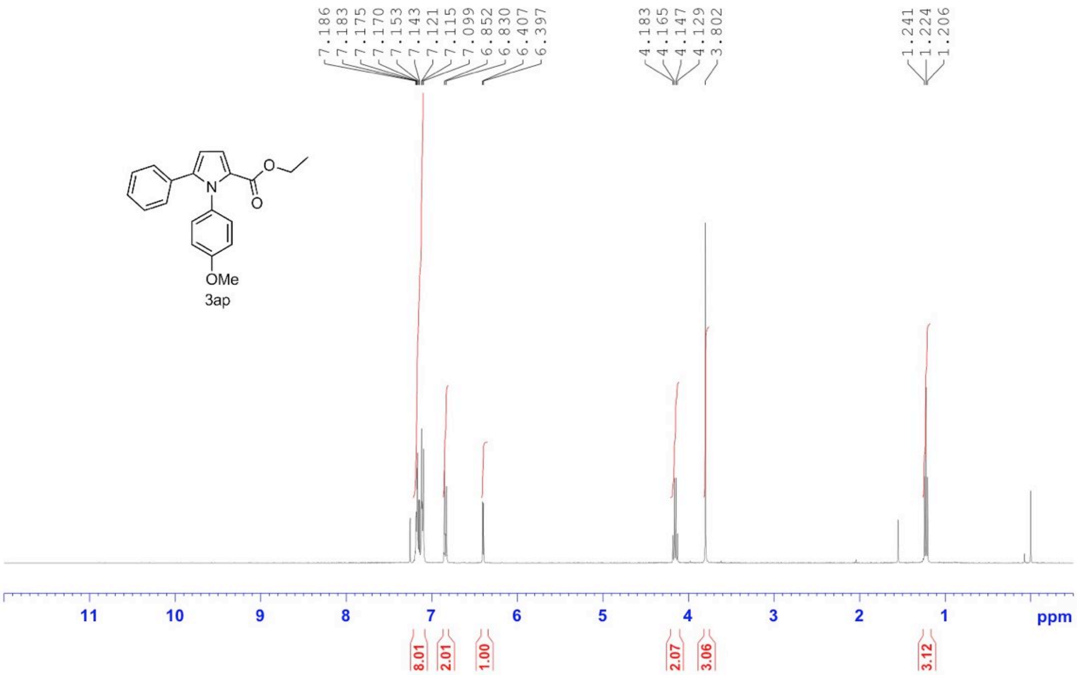

TWW-02-362-2 1HNMR
BBFO1 CDCl3

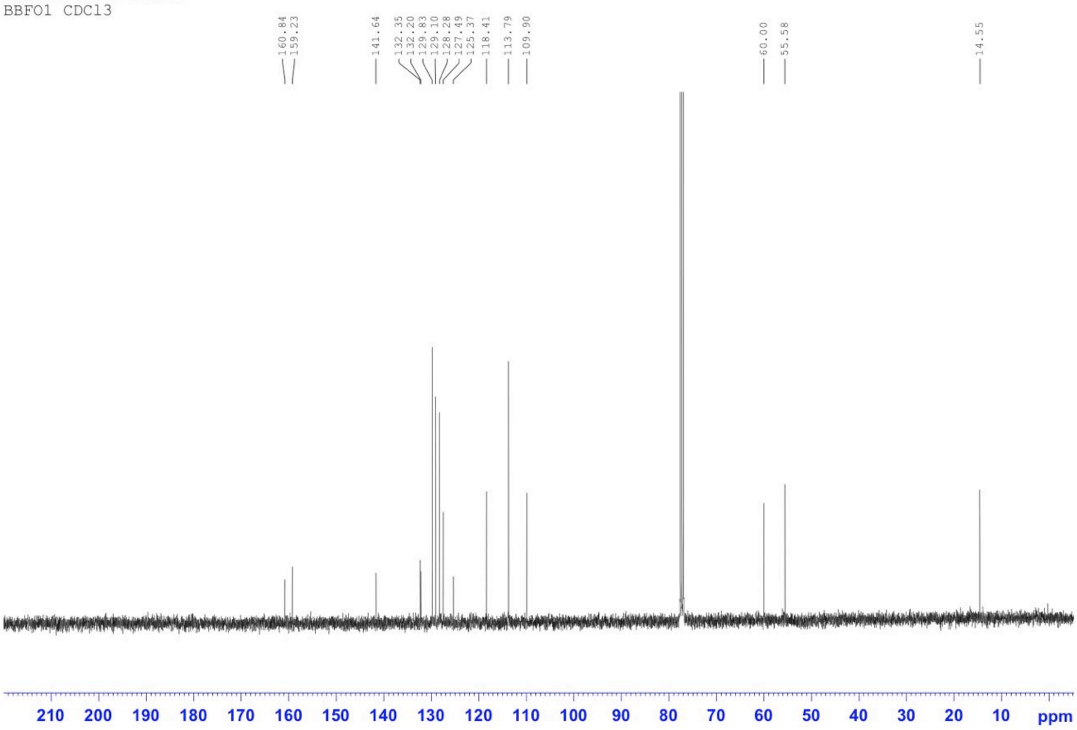

TWW-02-184-2, 1H BBFO1, CDCl3

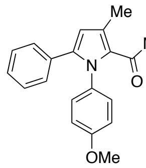

3aq

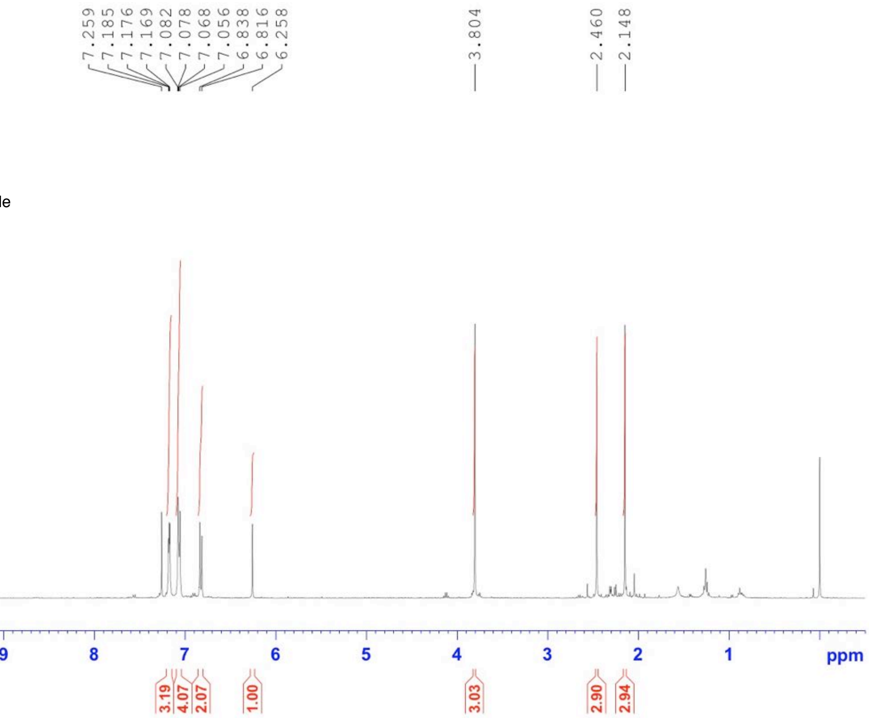

TWW-02-184-2x, 13C NMR, 400M Hz, BBFO2

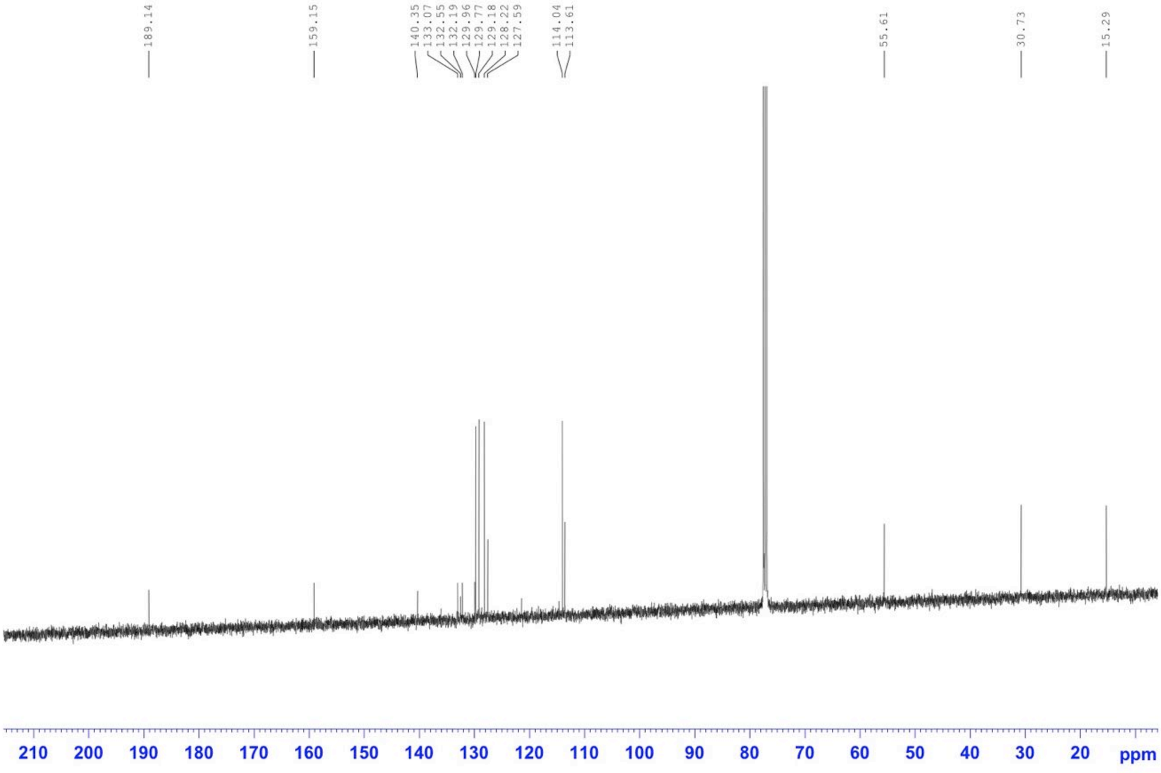

TWW-02-184-1, 1H BBFO1, CDCl3

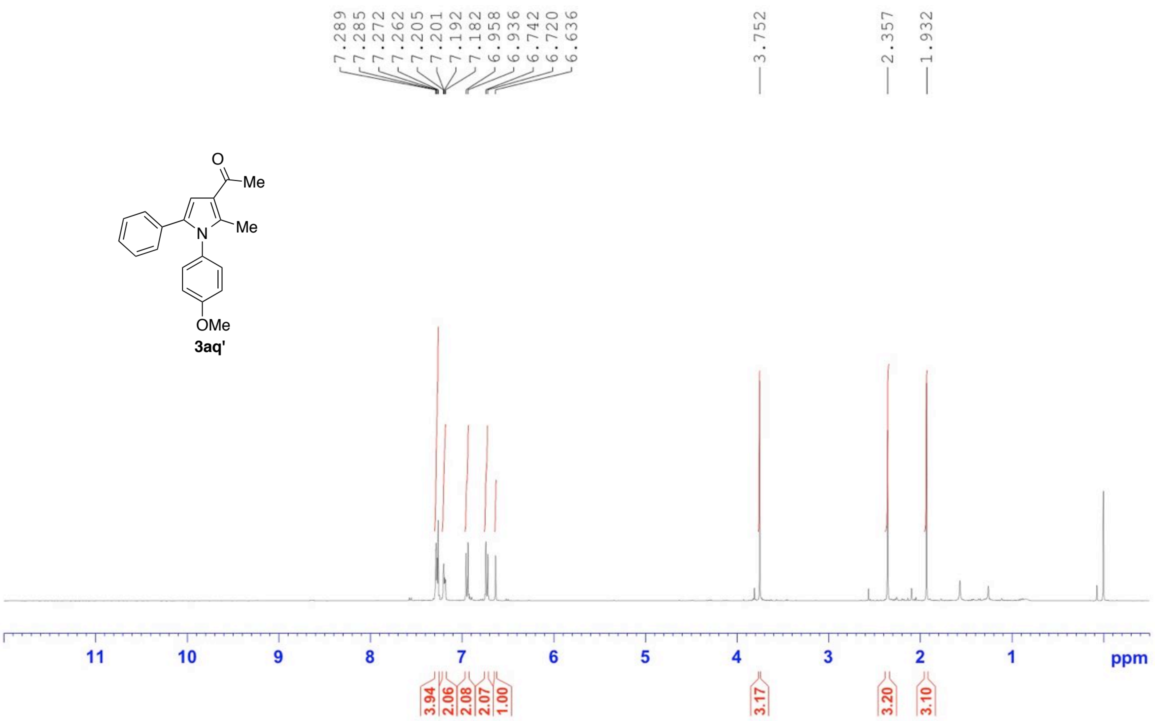

TWW-02-184-1x, 13C NMR, 400M Hz, BBFO2

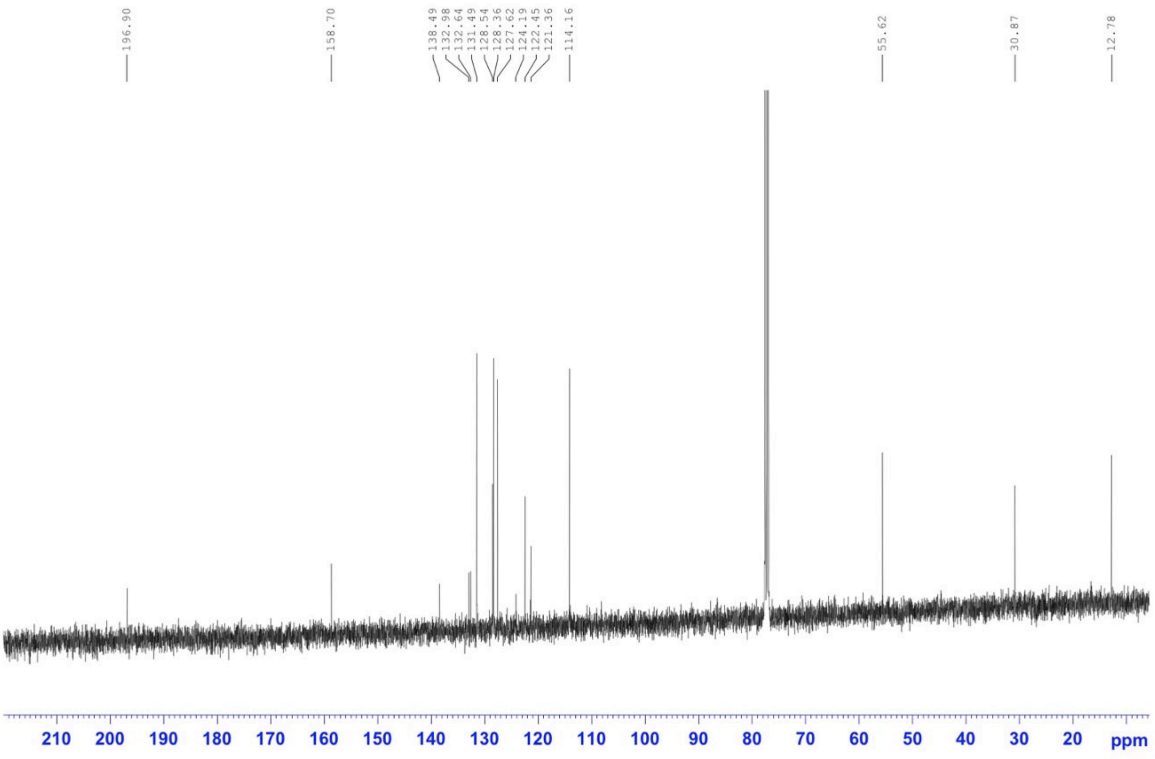

TWW-03-026-1, 1H, CDCl3, BBFO1

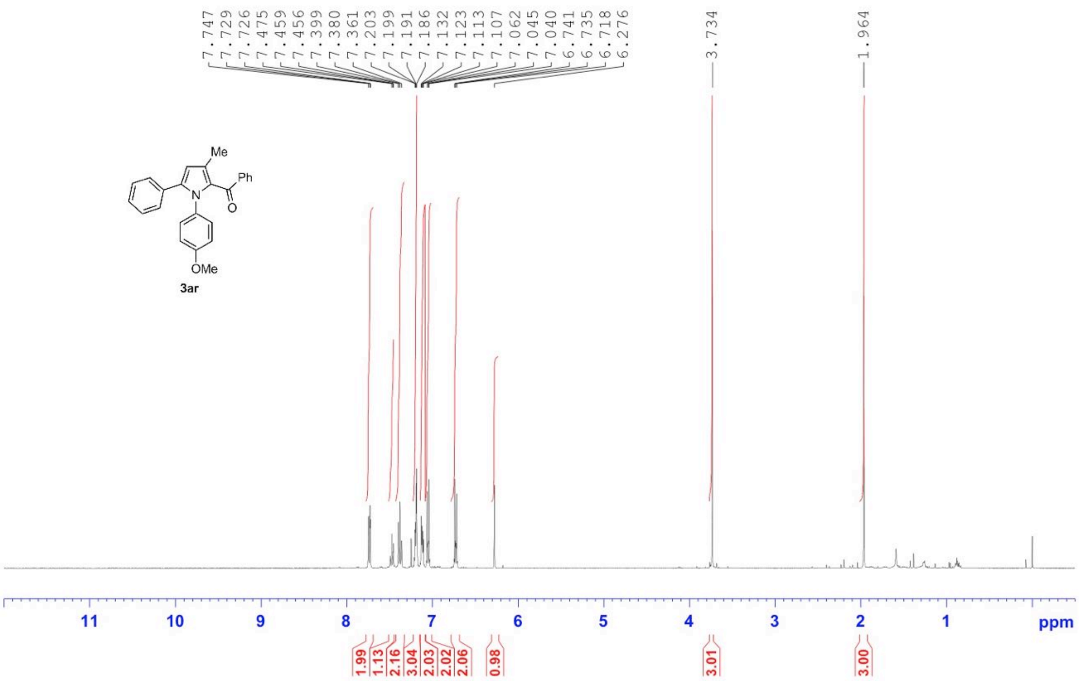

TWW-03-026-1, 13C, CDCl3, BBFO1

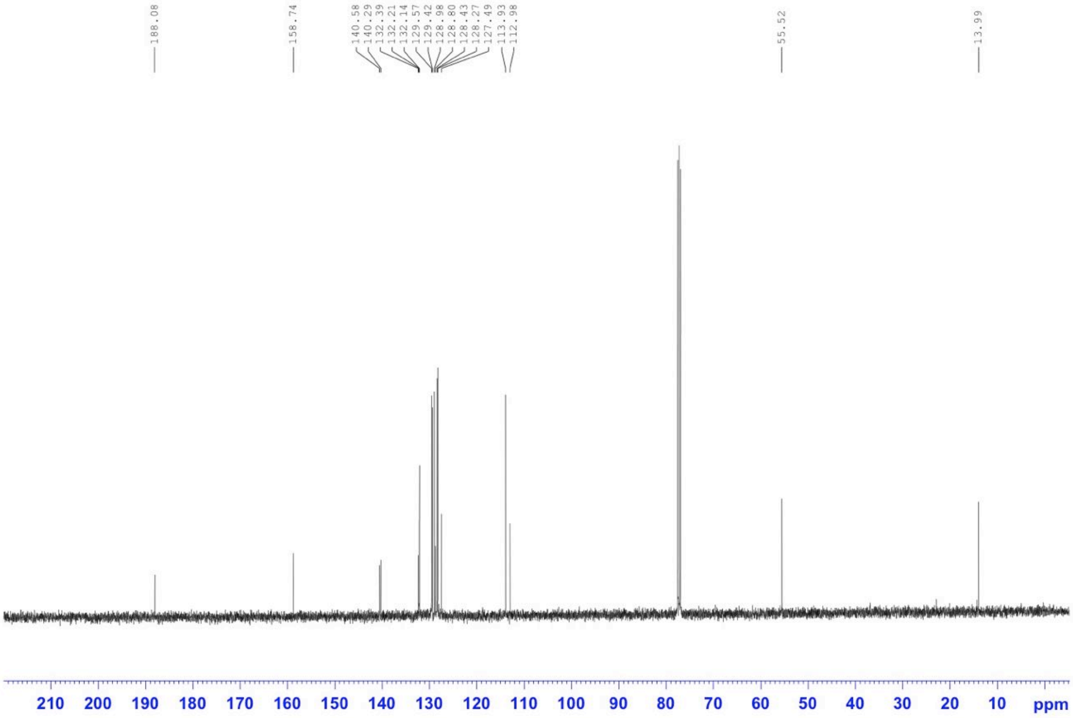

TWW-03-026-2, 1H, CDCl3, BBFO1

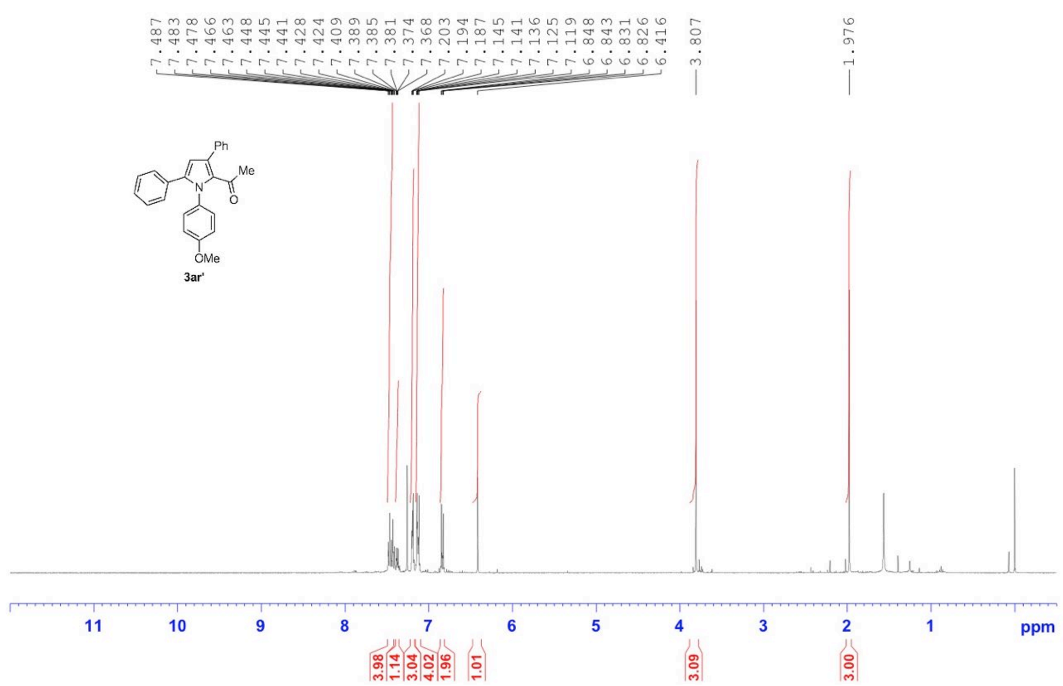

TWW-03-026-2, 13C, CDCl3, BBFO1

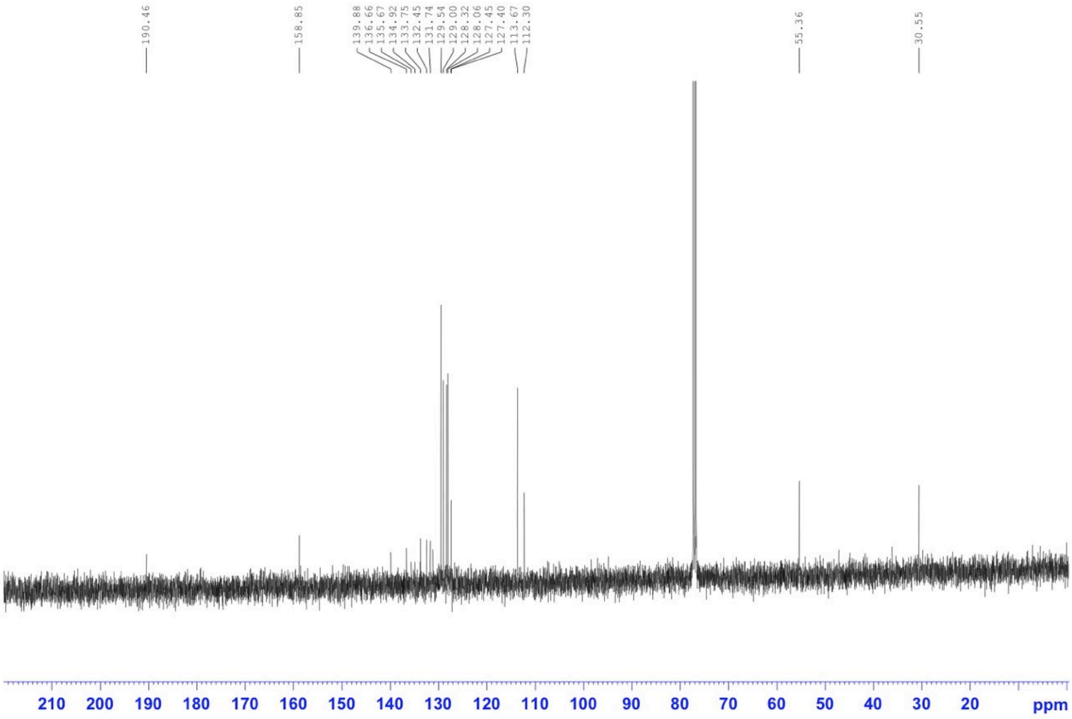

TWW-02-407x, 1H, BBFO1, CDCl3

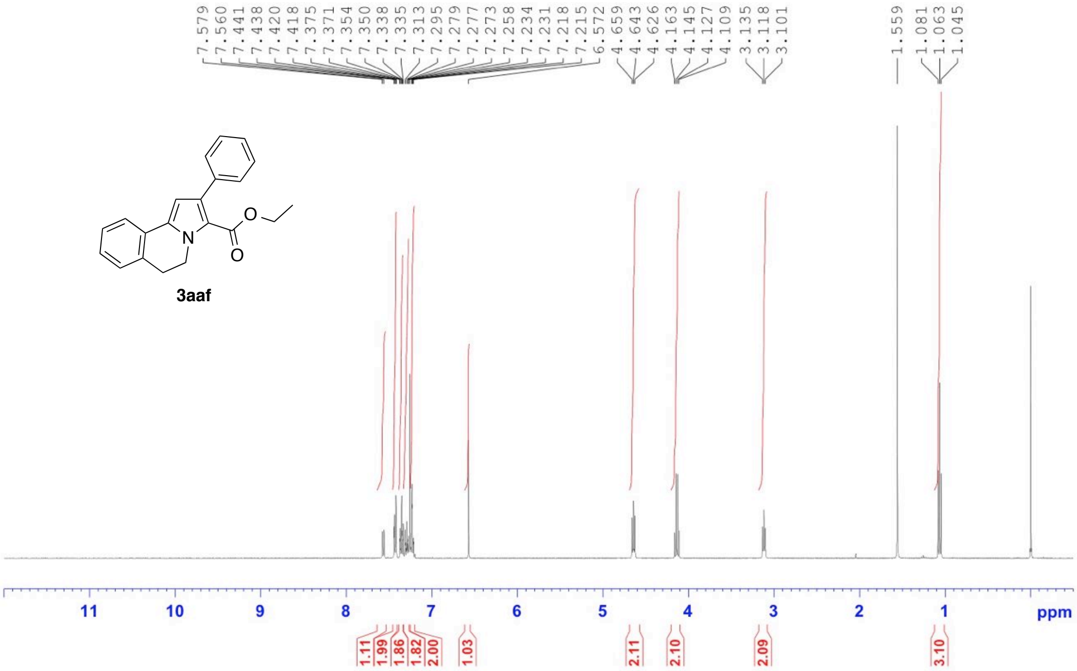

TWW-02-407, 13C, BBFO1, CDCl3

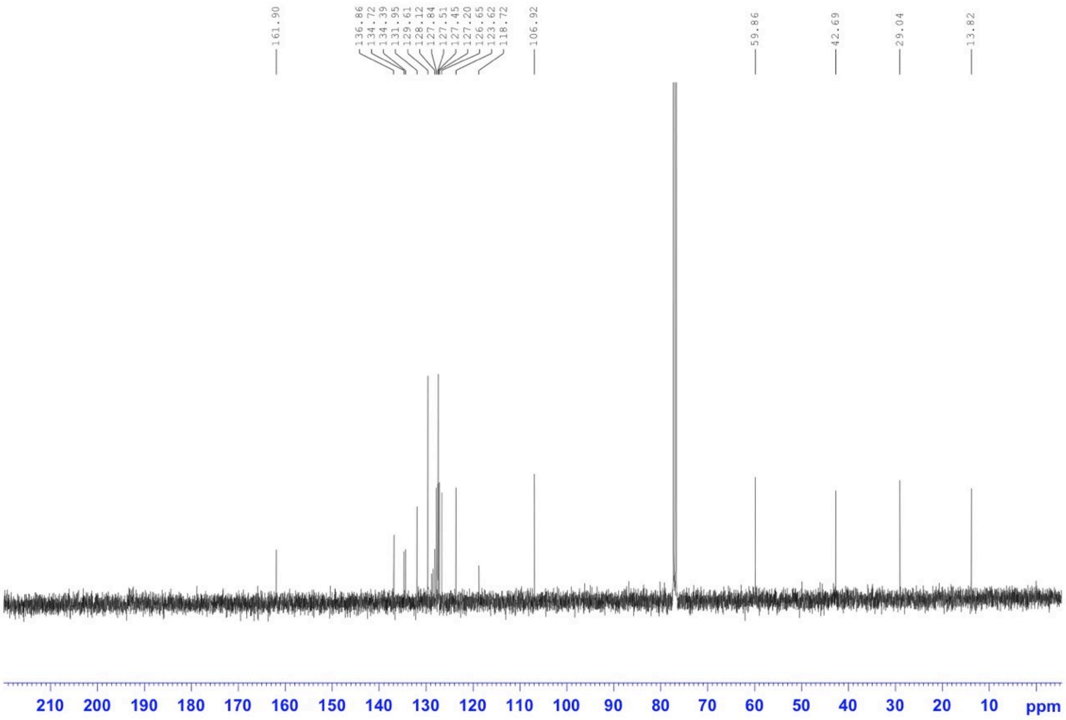

TWW-02-408x, 1H, BBFO1, CDCl3

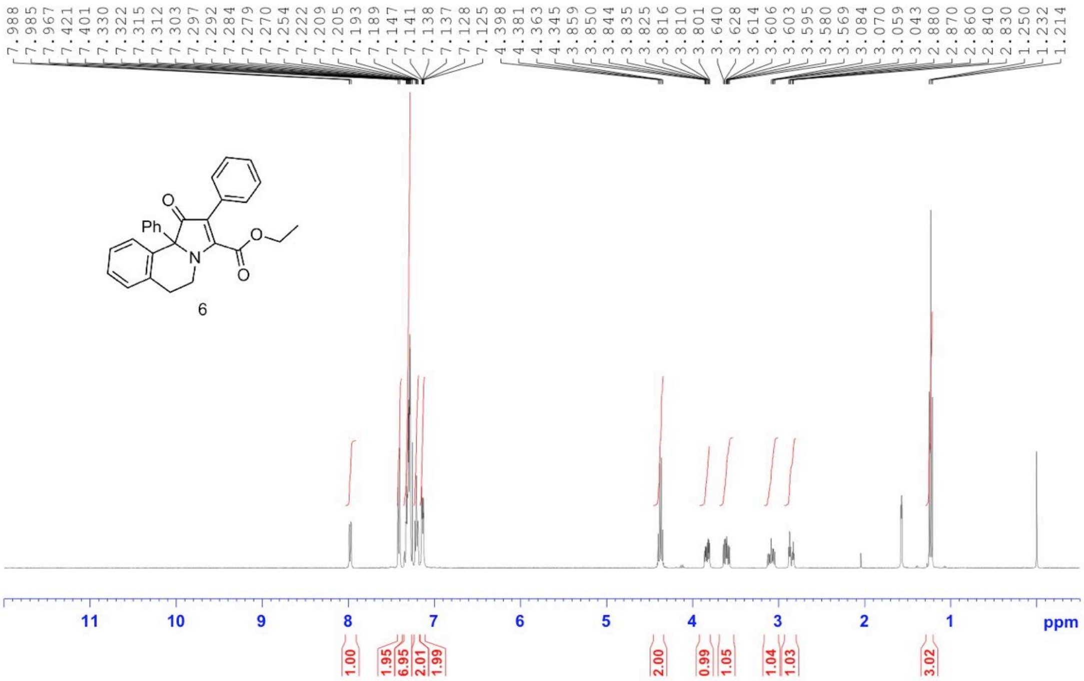

TWW-02-408x, 13C, BBFO1, CDCl3

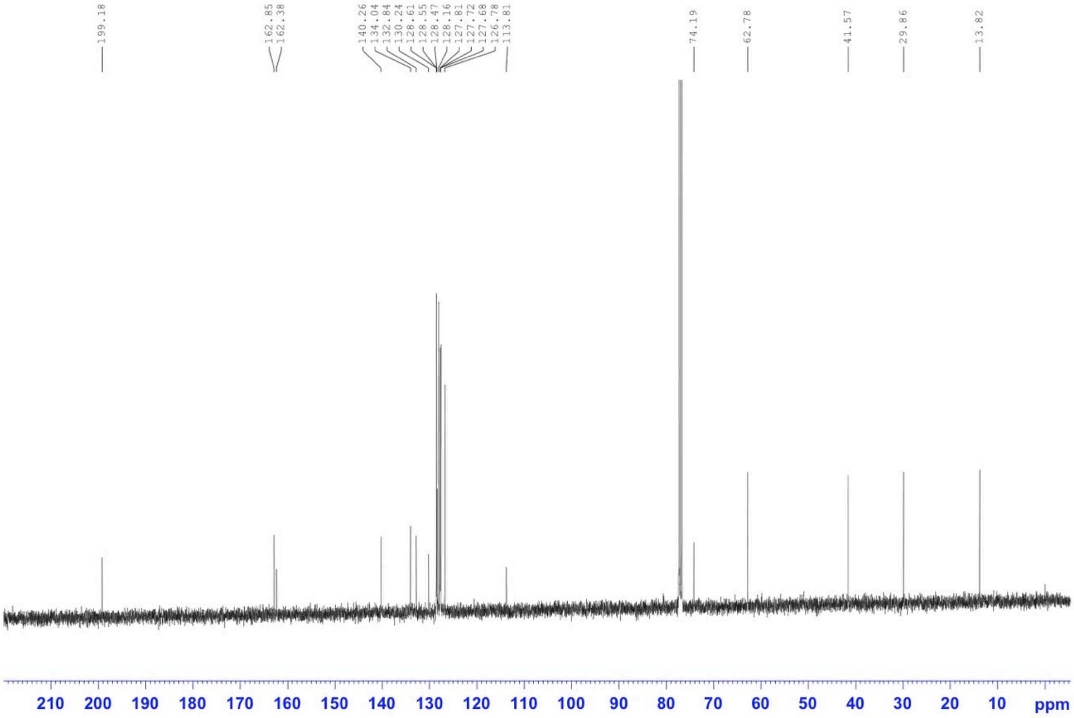

TWW-02-363 1HNMR
BBFO1 CDCl3

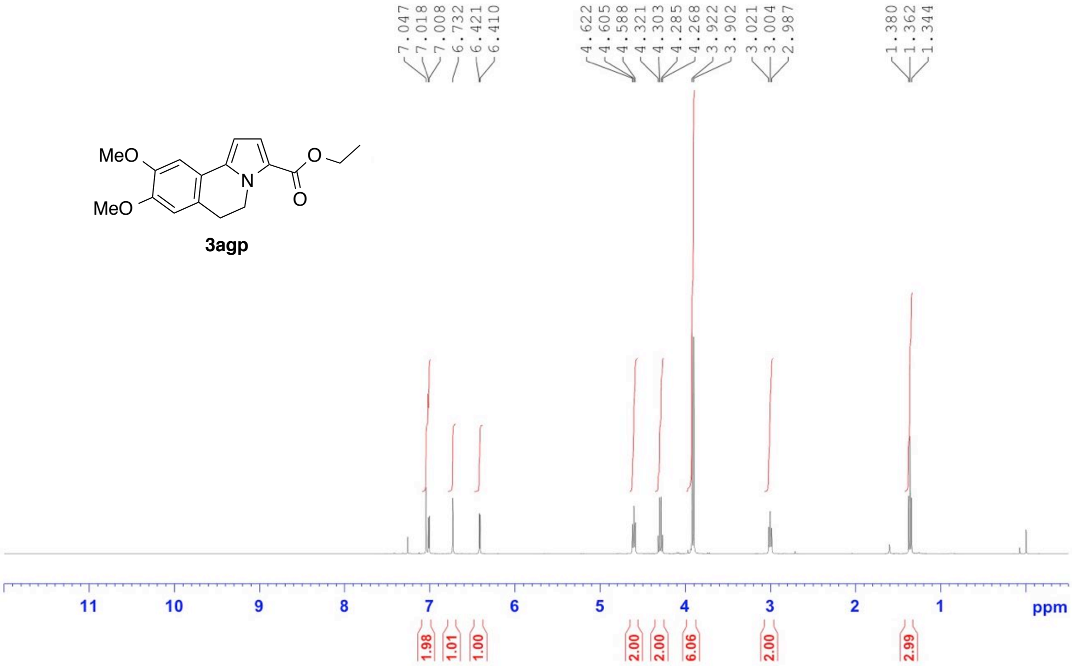

3agp

TWW-02-363 13CNMR
BBFO1 CDCl3

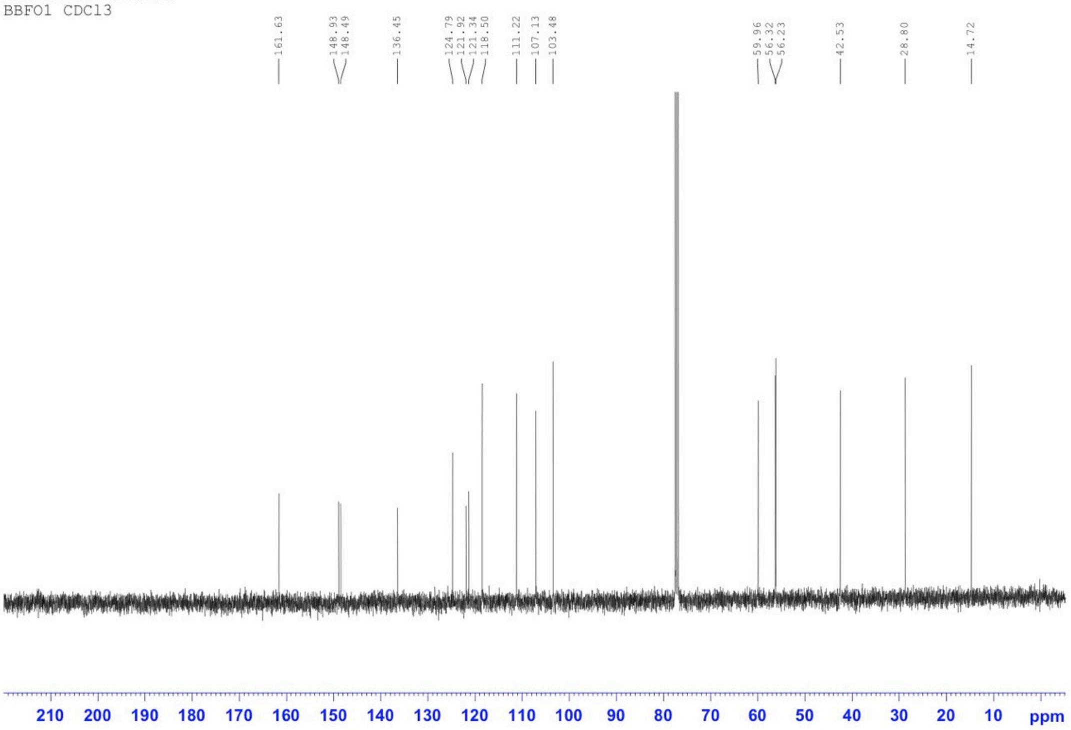

TWW-02-366 1HNMR
BBFO1 CDCl3

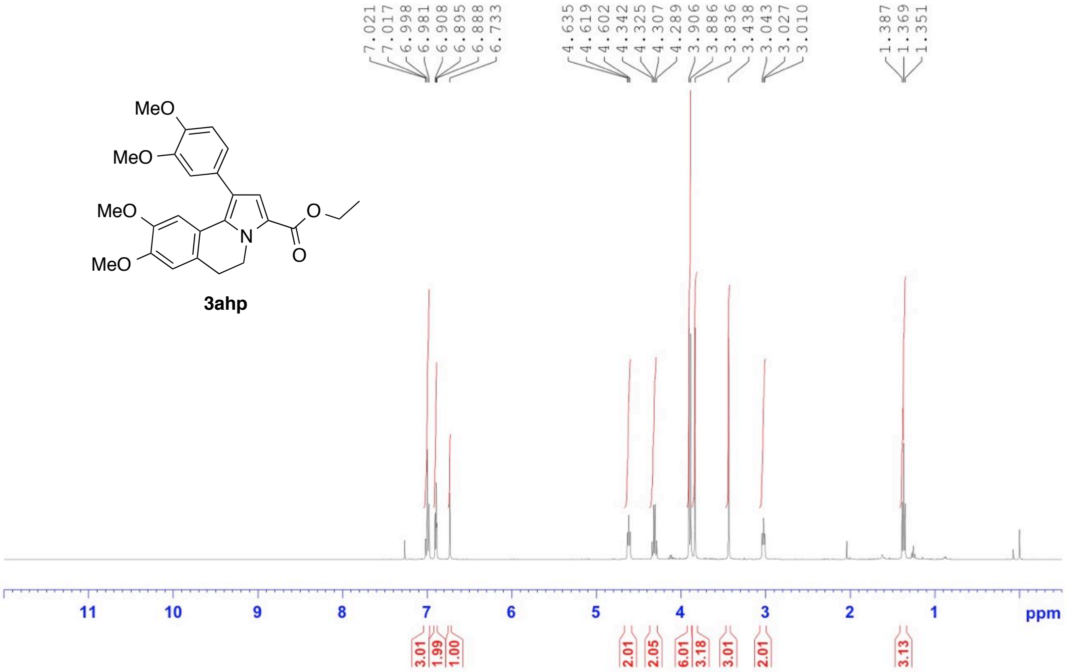

TWW-02-366 13CNMR
BBFO1 CDCl3

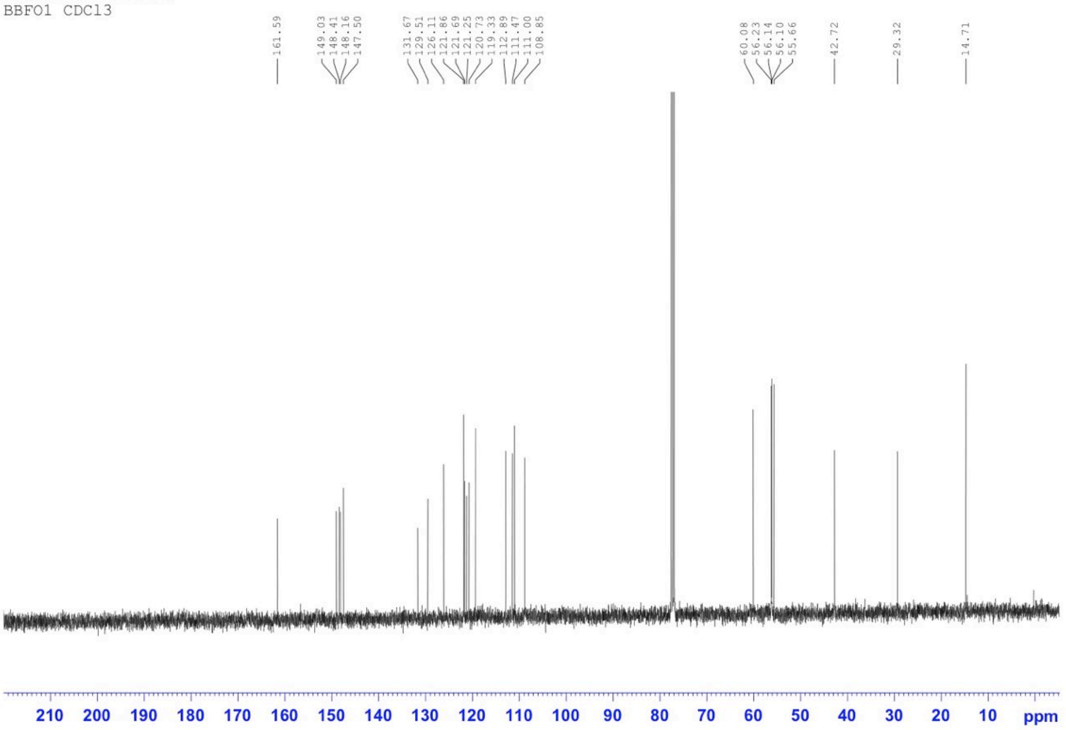

Supplement: Supplementary file 1 [file SC-006-C5SC02322J-s001.pdf]
